# Population Genomic Analysis of Strain Variation in *Leptospirillum* Group II Bacteria Involved in Acid Mine Drainage Formation 

**DOI:** 10.1371/journal.pbio.0060177

**Published:** 2008-07-22

**Authors:** Sheri L Simmons, Genevieve DiBartolo, Vincent J Denef, Daniela S. Aliaga Goltsman, Michael P Thelen, Jillian F Banfield

**Affiliations:** 1 Department of Earth and Planetary Science, University of California, Berkeley, Berkeley, California, United States of America; 2 Chemistry Directorate, Lawrence Livermore National Laboratory, Livermore, California, United States of America; University of California, Davis, United States of America

## Abstract

Deeply sampled community genomic (metagenomic) datasets enable comprehensive analysis of heterogeneity in natural microbial populations. In this study, we used sequence data obtained from the dominant member of a low-diversity natural chemoautotrophic microbial community to determine how coexisting closely related individuals differ from each other in terms of gene sequence and gene content, and to uncover evidence of evolutionary processes that occur over short timescales. DNA sequence obtained from an acid mine drainage biofilm was reconstructed, taking into account the effects of strain variation, to generate a nearly complete genome tiling path for a *Leptospirillum* group II species closely related to L. ferriphilum (sampling depth ∼20×). The population is dominated by one sequence type, yet we detected evidence for relatively abundant variants (>99.5% sequence identity to the dominant type) at multiple loci, and a few rare variants. Blocks of other *Leptospirillum* group II types (∼94% sequence identity) have recombined into one or more variants. Variant blocks of both types are more numerous near the origin of replication. Heterogeneity in genetic potential within the population arises from localized variation in gene content, typically focused in integrated plasmid/phage-like regions. Some laterally transferred gene blocks encode physiologically important genes, including quorum-sensing genes of the LuxIR system. Overall, results suggest inter- and intrapopulation genetic exchange involving distinct parental genome types and implicate gain and loss of phage and plasmid genes in recent evolution of this *Leptospirillum* group II population. Population genetic analyses of single nucleotide polymorphisms indicate variation between closely related strains is not maintained by positive selection, suggesting that these regions do not represent adaptive differences between strains. Thus, the most likely explanation for the observed patterns of polymorphism is divergence of ancestral strains due to geographic isolation, followed by mixing and subsequent recombination.

## Author Summary

Communities of microbes in nature consist of a large number of distinct individuals. The variation in DNA sequence between these individuals contains a record of the evolutionary processes that have shaped each community. In most environments, however, the high number of distinct species makes obtaining information about the nature of this variation difficult or impossible. We obtained large amounts of sequence data for a natural community in an acid mine drainage system consisting of only a few species. This enabled us to reconstruct the genome of the dominant bacterium (*Leptospirillum* group II) and obtain detailed information about sequence variation between individuals, including differences in both gene content and gene sequence. Our analysis shows extensive recombination between closely related populations, as well as fewer instances of recombination between more distantly related individuals. Additionally, viruses and plasmids account for high variability in gene content between individuals. We conclude that sequence-level variation in this population is maintained through neutral processes (migration, recombination, and genetic drift) rather than natural selection. This suggests that closely related strains of the *Leptospirillum* group II population may not be ecologically distinct.

## Abstract 3

Deep sequencing of a low-complexity microbial community revealed extensive recombination as well as polymorphic and gene content variation between individuals of the dominant organism. We show that strains defined by linked polymorphisms are not maintained by positive selection; instead, they are predominantly maintained by the forces of migration and drift.

## Introduction

A characteristic of natural populations is that they are comprised of individuals that are, in the majority of cases, not genomically identical to each other. Heterogeneity present at any time reflects the outcome of the interplay between processes that create variation (e.g., mutation and lateral gene transfer) and those that remove it (e.g., selection and genetic drift). In addition, genetic variation can be introduced into a population via migration and subsequent recombination. Variation between individuals appears both as divergence at the single nucleotide level and the presence of hypervariable gene “islands” within a more stable set of genes shared by multiple isolates [1–4]. The potential adaptive value of this variation is an important and controversial question in microbial ecology [5,6]. Through analyses of natural populations that explicitly consider genetic variability, it is possible to evaluate the basis for potential metabolic differences and infer aspects of the evolutionary processes that occur over relatively short timescales within natural communities.

Initial deductions of the processes that shape microbial lineages were based on genomic studies of single isolated strains. More recently, genomic comparisons amongst multiple isolated strains of the same species have been possible [2,7–13]. These studies defined the concept of a pangenome for a species, reflecting the observation that the gene content of a population exceeds that of any single individual member [14]. Comparative genome analyses revealed extensive gene transfer via mechanisms that include insertion of phage or plasmid DNA. However, there has been only limited analysis of the extent of heterogeneity in gene content within coexisting cells that comprise natural populations [15–17]. Such studies benefit from relatively deep genomic sampling so that orthologous regions can be compared. Despite the rapid increase in number of metagenomic studies of natural communities [18–22] and environments [16,17,23–27] over the past few years, few datasets provide the amount of sequence required for near-complete genome reconstruction and analysis of population-level heterogeneity. Relatively low-diversity microbial communities are especially tractable for this type of analysis, especially those dominated by one organism type with a tightly defined population structure.

Acid mine drainage (AMD) biofilms have proven particularly well suited to community genomic analyses because the majority of the biofilm community consists of four to six organism types, one of which is typically dominant. This organism can be relatively deeply genomically sampled by shotgun sequencing [21]. Tyson et al. [21] reported extensive reconstruction of genome fragments from the five species in one AMD biofilm using approximately 76 Mb of genomic sequence data. Most of the composite genome for the dominant bacterium, a member of *Leptospirillum* group II that is closely related to Leptospirillum ferriphilum, was sampled to a depth of approximately 12×. Binning of assembled fragments was achieved primarily based on GC content (to separate out fragments deriving from coexisting archaea) and sequence coverage (sampling depth, to separate out fragments from a *Leptospirillum* group III population). Initial studies using this dataset revealed evidence for transposon proliferation [15], genetic exchange between relatively closely related archaeal organisms via homologous recombination [21,28], and gain and loss of genes from proviral DNA [15].

Simultaneous analysis of DNA sequences derived from natural communities presents a number of challenges, the first of which is to correctly assign genome fragments to the appropriate organism. The most robust approach for binning is genome reconstruction [18,21], but this task is complicated by fragmentation of assemblies due to genomic heterogeneity [29], as confirmed at a limited number of loci in a metagenomic dataset of complex environmental systems [16]. In the current study, we resolved much of this fragmentation via manual curation of an assembly of an expanded genomic dataset (130 Mb) to capture the form and distribution of genetic variation genome-wide in a natural population.

The coexistence of multiple closely related strains is a commonly observed phenomenon in microbial communities, and is apparent from studies of cultured isolates [2,11,30], marker gene surveys [31–33], and metagenomic data [3,15–17]. Despite the existence of a robust theoretical apparatus in population genetics to determine the strength and direction of selection using polymorphism and divergence data (reviewed in [34]), these approaches have not been previously applied to the question of whether genome-wide nucleotide divergence within microbial populations reveals adaptive differences between closely related, coexisting strains. In this paper, we address the question of whether adaptive differences between closely related, coexisting strains of *Leptospirillum* group II can be detected using population genetic analysis of single nucleotide polymorphisms (SNPs). We attempt to distinguish in situ differentiation and selection from migration and recombination as the origin of the observed patterns of nucleotide variation. Through comprehensive analysis of both variation in gene sequence and gene content, we uncover details of the recent natural history of *Leptospirillum* group II.

## Results

### Core Genome Assembly and Annotation

*Leptospirillum* group II scaffolds, their functional annotations, and alignments to the earlier assembly [21] are reported in Table S1. The near-complete genome, spread over 79 contigs, representing 2.72 Mb of sequence, as well as the 2,588 ORFs with manually curated start positions and functional annotations, is deposited at the National Center for Biotechnology Information (NCBI), along with the trace files for the 54 Mb of new sequence (accession number AADL00000000). The total number of assembled reads is 42,701, indicating that each read likely derived from a single cell (there were ∼10^8^ cells in the sequenced sample [21]). Metabolic analyses and ecological profiles of the *Leptospirillum* group II species will be reported separately (D. S. A. Goltsman, V. J. Denef, S. W. Singer, N. C. VerBerkmoes, M. Lefsrud, et al., unpublished data).

**Figure 1:**
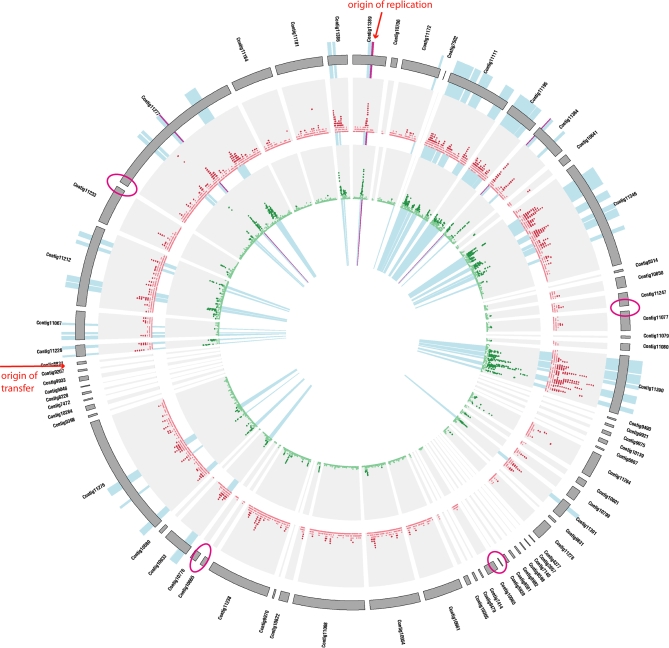
The Genome of *Leptospirillum* group II, Showing That SNP Density and Frequency Are Roughly Symmetrical around the Origin of Replication Circular diagram showing contig ordering, SNP density, minor allele frequency, and location of strains around the genome. The outer ring shows all contigs in the 5-way CG assembly, ordered by mate pairing and by reference to the UBA genome. Four locations where the join is uncertain are circled in magenta. The first inner ring shows a moving average of SNP density (1-kb windows, 50-bp slide, scale 0–1). Dark red indicates local SNP density of greater than 0.5% while pink indicates less than 0.5%. The second inner ring shows a moving average of minor allele frequency (scale 0%–0.7%). Dark-green points indicate average minor allele frequencies for a window greater than 0.05%. Light-blue highlights indicate location of substrains used to analyze variation within the 5-way CG population (>99% sequence similarity). Purple highlights indicate the location of deeply sampled reads of more divergent *Leptospirillum* group II strains incorporated into the population (∼94% sequence similarity). The image was generated with Circos (M. Krzywinski, http://mkweb.bcgsc.ca/circos/).

The core genome of the 5-way CG *Leptospirillum* group II population described here is largely syntenous with that of the UBA *Leptospirillum* group II population reported previously [18]. (“5-way CG” and “UBA” indicate different sites within the Richmond Mine from which samples were collected for genomic sequencing. The dominant *Leptospirillum* group II species derived from each of these assemblies were labeled to indicate their geographic origin.) Where lack of sequence information precluded ordering of scaffolds, scaffolds were ordered based on the UBA assembly. These junctions are evident in Table S1 as points where scaffold numbers change. Locations with uncertain ordering in the UBA assembly are circled on the outer ring of Figure 1. Although it is possible that some of these junctions correspond to *Leptospirillum* group II genome rearrangements, only one genomic rearrangement, probably associated with virus insertion, was observed within the contiguous sequences.

### Genomic Variation

**Figure 2:**
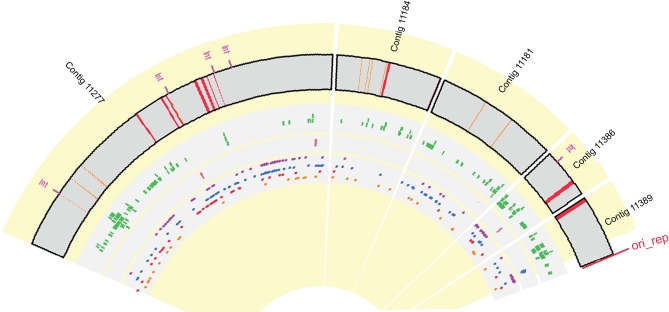
Overview of Different Source of Pangenomic Variation over a 500-kb Segment of the 5-Way CG Type *Leptospirillum* Group II Genome, Including the Origin of Replication Only the mapped segment of the genome is shown. In the outer ring, tRNAs are indicated with orange, transposons with red, and integrases with “Int.” The location and length of strain variant paths (see main text) are shown in green in the first inner ring, and the locations of recombinant reads (blue = UBA-type, and red = non–UBA-type) are shown in the second inner ring. The innermost ring shows nonsynonymous SNPs in blue, synonymous SNPs in purple, intergenic SNPs in red, and SNPs resulting in frameshifts in orange. The image was generated with Circos (M. Krzywinski, http://mkweb.bcgsc.ca/circos/).

**Figure 3:**
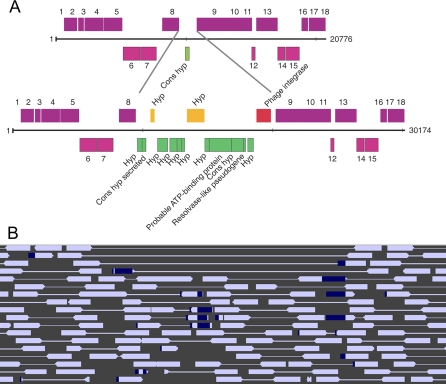
Diagram Showing Gene Content Variation Due to Integrase Insertion in a Region of Contig 11277 (A) The gene content of the variant regions are shown. One of these contains an insertion of an integrase (red) and associated genes, mostly hypotheticals or conserved hypotheticals (orange on the top strand, green on the bottom strand). Genes on opposite strands are shown in dark purple (top) or light purple (bottom). Gene annotations are given in text, or by numbers, as follows: (1) putative GTP binding protein; (2) hypothetical protein; (3) protein of unknown function; (4–5) putative peptidase M16; (6) hypothetical protein; (7) putative Na^+^/H^+^ antiporters; (8) putative metabolite transport protein; (9) leucyl-tRNA (Cons hyp); (10) protein of unknown function; (11) probable polymerase III; delta subunit; (12) ribosomal protein S20; (13) putative virulence factor, MVIN-like; (14) probable HNH endonuclease; (15) protein of unknown function; (16) hypothetical protein; (17) hypothetical protein; and (18) probable ATPase, PP-loop superfamily. (B) The associated reads are shown. Dark blue regions show inserted sequence divergent from the composite.

Although extensive reconstruction of a core genome following the dominant path during assembly was possible, variation in gene content, sequence composition, and recombination events contribute to population-level heterogeneity. We mapped the sources of variation for a 700-kb fragment (∼1/4 of the *Leptospirillum* group II genome) that includes the origin of replication (Figure 2). Although the majority of sequencing reads originating from the *Leptospirillum* group II population could be coassembled, there were numerous small contigs held in by mate-paired reads that did not coassemble due to localized high sequence variation, often due to sequence insertions or deletions. These indels can be identified by mate-paired reads separated by distances greater than expected based on the clone size and reads that transition to sequence that is highly divergent relative to the composite (Figure 3). Multiple variant paths, with shorter or longer inserts, are also evident in many of these regions.

Most of these strain variant paths consist of one or a few reads (Figure 2), and hence, full paths of most low-abundance variants could not be established. Some contigs, however, link at both ends to the core genome at locations consistent with insertions or deletion of sequence blocks that preserve synteny of flanking genomic regions. No clear correlation was observed between SNP density and the density of strain variant paths (Figure 2).

**Figure 4:**
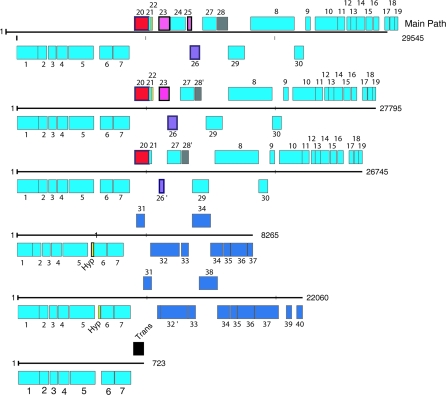
A High-Frequency Variant Region with Six Alternate Paths, One of Which Contains a Complete LuxIR Pathway The main genome path is shown on the top line with alternative paths below. Light blue indicates genes present on the main genome path, dark blue indicates genes shared between two of the variants, yellow indicates hypothetical proteins, black indicates transposases, red indicates phage integrases, and pink (top strand) and purple (bottom strand) indicate genes potentially involved in the LuxIR pathway. Hypothetical proteins in the LuxIR region are shown in grey. Genes are annotated as follows: (1) l-aspartate oxidase; (2) probable ferredoxin; (3) conserved hypothetical protein; (4) citrate synthase; (5) aconitate hydratase (same as aconitase); (6) succinyl-CoA synthetase, alpha subunit; (7) succinyl-CoA synthetase, beta subunit; (8) pyoverdine chromophore precursor synthetase; (9) hypothetical protein; (10) acetolactate synthase, large subunit; (11) acetolactate synthase, small subunit; (12) hypothetical protein; (13) conserved hypothetical protein; (14) biotin synthesis; (15) DNA binding protein; (16) hypothetical protein; (17) protein of unknown function; (18) hypothetical protein; (19) acetylornithine aminotransferase; (20) phage integrase; (21–22) hypothetical protein; (23) Lux R; (24) transposase; (25) Lux R; (26) Lux I; (26′) short Lux I; (27) cytochrome P450 family protein; (28) hypothetical protein; (28′) truncated hypothetical protein; (29) transposase; (30) transposase; (31) hypothetical protein; (32) diguanylate cyclase; (32′) diguanylate cyclase frame shifted; (33) putative protein tyrosine phosphatase; (34) UDP-glucose 4-epimerase; (35) hypothetical protein; (36) UDP 6-dehydrogenase; (37) glucosamine-fructose-6-phosphate aminotransferase; (38) putative sigma54-specific transcriptional regulator, Fis family; (39) hypothetical protein; and (40) putative sigma54-specific transcriptional regulator, Fis family.

A common cause of genome assembly fragmentation is the insertion of transposases into the genomes of some individuals and not others. The genome path for the manually curated assembly (Table S1) was reconstructed across many of these insertion points. Inserted blocks predominantly encode hypothetical proteins (e.g., Figure 3A), as well as integrases, transposases, or proteins involved in plasmid maintenance, replication, and/or transmission. For one anomalously highly variable region, six different strain paths could be reconstructed. Interestingly, three of these variants encode LuxR or LuxI, though only one variant has a potentially functional LuxIR system (Figure 4).

### Genome-Wide Patterns in Sequence Composition

We identified and classified every polymorphic site in the assembly. Different Phred quality score cutoffs were used to examine the effect of sequencing error on SNP identification (Table S2), because SNPs appearing in only one read (“singletons”) are more likely to result from sequencing error than are replicated SNPs, yet the number of true singletons is important for subsequent population genetic analysis. Raising cutoff scores reduced the number of singleton polymorphic sites identified, particularly indels. Notably, the ratio of nonsynonymous to synonymous SNPs remained relatively constant for quality cutoffs of 25 and higher (range 0.84–0.88), as did the overall SNP density (range 0.06%–0.09%), suggesting that sequencing error affected these site classes in an unbiased fashion. The proportion of all SNPs found in two or more reads (replicated) ranged from 32% for a cutoff score of 20 to 47% for a cutoff score of 50. We used a quality cutoff of 25, corresponding to a per-base error probability of 3.2 × 10^−3^, based on an analysis of the effect of increasing quality scores on the outcome of population genetic tests (Table S4 and Text S1). For this quality cutoff, the overall SNP density was 0.09%, and 38% of SNPs were replicated.

We mapped the density of replicated and nonreplicated SNPs around the entire genome using sliding windows of length 1 kb and displacement 50 bp (Figure 1). The minor allele frequency was calculated for each polymorphic site in the assembly, and is defined as the fractional abundance of the less common allele. The density of SNPs is higher in the approximately one quarter of the genome flanking the origin of replication (“ori_rep”, located in contig 11389). The distribution of SNPs is skewed slightly to the right of the origin of replication. The SNP density is not noticeably higher near the putative origin of transfer (“ori_T,” in a large integrated plasmid containing conjugal transfer proteins) located in contigs 8831–9933. The distributions of replicated and nonreplicated SNP density are not noticeably different.

**Figure 5:**
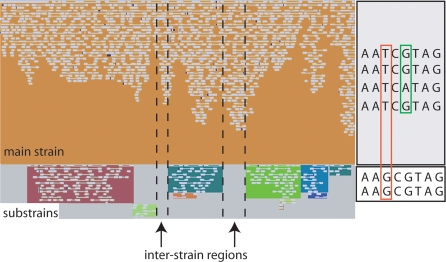
Schematic Illustrating How Strains Were Separated for Population Genetic Analyses Left: screenshot of contig 11111 (located near the origin of replication) from Strainer. Individual reads are shown as white blocks. Strains defined by shared polymorphisms are shown in distinct colors, with the main strain in orange. The vertical dashed lines indicate regions within the main strain not overlapped by any substrain and referred to as “intersubstrain regions” in the main text. Right: schematic illustrating the identification of polymorphisms within and between strains. The orange box surrounds a site classified as a fixed difference between the main strain and a substrain. The green box surrounds a site classified as polymorphic (three Gs, one A in the main strain; two Gs in the substrain).

Regions with both high SNP density and high minor allele frequency are indicative of the presence of blocks of variant sequence. We grouped closely related SNP patterns into strains genome-wide with Strainer [26] (Figure 5). Substrains were defined based on replicated, linked SNPs (light-blue highlights in Figure 1). The strains are separated from each other by regions with lower SNP density, making long-range reconstruction of substrains impossible. Nearly all the substrains are closely related (>99.5% nucleotide identity) to the dominant 5-way CG type. Although the similar coverage depth of some of these substrains suggests their linkage (Figure S1), we do not know how many distinct lineages containing these substrains occur in the present population.

### Variation Due to Recombination

**Figure 6:**
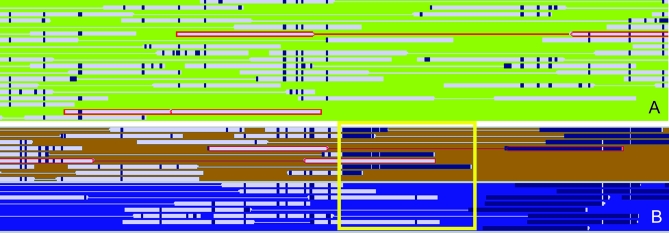
Recombination between Related Individuals Is Directly Observed in Some Sequence Reads (A) Diagram showing an area in contig 11277 with possible evidence for recombination between closely related strain variant types. Reads whose sequence type is a hybrid of a variant and the dominant strain types are outlined in red. The reads corresponding to the consensus (majority) sequence type are not shown. (B) Area of contig 11277 at approximately 140 kb where highly divergent sequence relative to the composite has inserted into three variants: the dominant strain variant (contig consensus sequence), as indicated by the upper read outlined in red, the brown strain (note that one individual sampled lacks the insert [lower read outlined in red]), and the blue strain. In the region identified by the yellow box, the contig 11277 consensus sequence appears to be a distinct *Leptospirillum* variant (dissimilar to both 5-way CG and UBA types) and the variant sequence (brown strain) is likely the pre-recombination 5-way CG-type sequence. Identity between contig 11277 and the sequence terminating the brown and blue strains is 72%. Recombinant reads are highlighted in red.

We identified a number of locations where a subset of individuals with a replicated SNP pattern transitioned into another replicated pattern within or between mate-paired reads, indicating homologous recombination (Figure 6). Recombination events between very closely related sequence types are difficult to identify, so the best examples involve events between the dominant strain type and relatively SNP-rich variants (e.g., Figure 6A).

**Figure 7:**
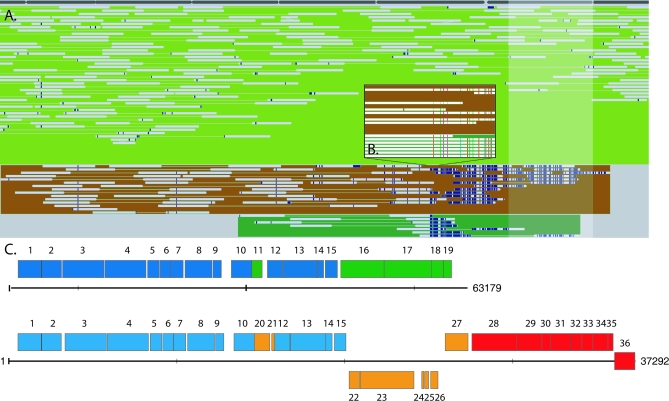
Extensive Strain Variation around the Origin of Replication Indicates Multiple Recombination Events (A) Strain variation captured within contig 11389. The white shading indicates the position of the ATPase involved in DNA replication. The composite sequence corresponds to the sequence of the olive-green strain type. Dark-blue regions on the reads indicate bases that disagree with the composite, either due to sequencing error (especially at read ends), insertions, or SNPs. Results illustrate the existence of three sequence types in this region (also see [B]). Although the brown and dark-green strain groups are highly divergent relative to the composite sequence over a region that begins shortly before the origin, it important to note that it is the dominant sequence type that becomes identical to the UBA *Leptospirillum* group II genome type due to a recombination event. (B) At higher magnification, it is evident that there were two 5-way CG strains in the population, only one of which was involved in the recombination event. The brown and dark-green strain sequence types terminate when they become too divergent to be coassembled into 11389. Most mate pairs missing from reads from the brown and dark dark-green strains at the base of the figure place at the start of scaffold 11386. (C) Diagram illustrating the two sequence variants present in the 5-way CG population, reconstructed at 11389 (top) and 11386 (bottom). Beyond this point, divergent small phage-like regions are followed by a 25-kb region in which all cells have the UBA *Leptospirillum* group II genome type. Note that the recombination block carries Cas proteins and the CRISPR locus (not shown in detail). Blue indicates genes shared between the two sequence variants, green indicates genes present only in the 11389 variant, orange indicates genes present only in the 11386 variant, and red indicates the cas proteins (100% identical between variants). Genes are annotated as follows: (1) chromosomal replication initiator protein (RepA); (2) DNA polymerase III, beta chain; (3) DNA gyrase, B subunit; (4) DNA gyrase, A subunit; (5) protein of unknown function; (6) putative radical activating enzyme; (7) ExsB protein; (8) leucyl aminopeptidase; (9) SsrA-binding protein; (10) putative integrase; (11–15) hypothetical protein; (16–17); putative Type III restriction/modification system, M and R subunits; (18–19) hypothetical protein; (20) hypothetical protein; (21) phage DNA binding protein; (22) conserved protein of unknown function; (23) DNA methyltransferase/helicase; (24–25) hypothetical protein; (26–27) probable transposase; (28) Cas3; (29) Cas1; (30) Cas2; (31) Cas4; (32) Cas5; (33) Cas3; (34) Cas1; (35) Cas2; and (36) CRISPR locus.

Recombination events also involve a distinct sequence type with approximately 94% sequence identity to the composite sequence. Some reads were approximately 100% identical to the UBA *Leptospirillum* group II sequence type (Figures 6 and 7). In the entire genome, only nine loci showed direct evidence of recombination between CG and UBA types, three of which are covered at high-read depths (Table S3). Two of these are CG-UBA recombinants (contigs 11364 and 11389), whereas one is a recombinant between CG-type reads and a distinct, divergent *Leptospirillum* group II genome type not previously observed (contig 11277, genes 147–149). All three deeply sampled recombinant regions are indicated with purple highlights in Figure 1.

One of the deeply sampled CG-UBA recombinant regions begins very close to the origin of replication in contig 11389 (Figure 6) and continues for 25 kb. This block is divided into two pieces by apparent dual phage insertion events at the end of contig 11389 and the center of contig 11386. These phages are not present in the corresponding region of the UBA genome, suggesting that the insertion events either occurred subsequent to the recombination event or that one of the phages mediated the initial recombination event. In the first half of the block (shown in Figure 7A), most cells in the 5-way CG population share 100% nucleotide identity to the UBA sequence type, whereas a subset contain a distinct sequence. Following a small region containing divergent phage-type proteins, all cells in the population contain an additional block of 100% UBA sequence encoding *cas* genes (Figure 7C). These *cas* genes are associated with clustered, regularly interspaced short palindromic repeat (CRISPR) regions, which have recently been shown to confer viral resistance [35].

The three deeply sampled recombinant regions between divergent *Leptospirillum* group II and CG-type reads were also analyzed with the McDonald-Kreitman (MK) test for selection. The MK test uses a 2 × 2 table to test the independence of the ratio of nonsynonymous to synonymous polymorphisms and the ratio of nonsynonymous to synonymous fixed differences [36]. The UBA-CG recombinant region near the origin of replication in contig 11389 (Figure 7) did not show significant evidence for positive or negative selection (dN/dS = 0.05, pN/pS = 0.13; Fisher exact test, *p* = 0.27). (pN equals the fraction of polymorphic nonsynonymous sites, pS equals the fraction of polymorphic synonymous sites, dN equals the fraction of sites with nonsynonymous fixed differences, and dS equals the fraction of sites with synonymous fixed differences.) The UBA-CG recombinant region in contig 11364 also did not show significant evidence for selection (dN/dS = 0.18, pN/pS undefined due to the lack of synonymous polymorphisms; Fisher exact test, *p* = 0.33). The region in contig 11277, which is a deeply sampled recombinant region between CG-type and unknown additional *Leptospirillum* group II variant reads (Figure 6), showed a statistically significant excess of polymorphic nonsynonymous sites relative to fixed nonsynonymous sites (dN/dS = 0.19, pN/pS = 0.90; Fisher exact test, *p* = 0.008).

### Population Genetic Tests of Strain Variation

Fourteen Phrap contigs (1.8 Mbp), totaling 66% of the 5-way CG *Leptospirillum* group II composite genome, had substrains of sufficient depth (average read depth 3.3) for analysis of polymorphism and divergence in comparison with the dominant strain group. These contigs are primarily located near the origin of replication (Figure 1) and overlap the region mapped manually in Table S3. A total of 50 separate substrains were defined, with average length 9.3 kbp and total length 464 kbp. The dominant strain in these regions had an average read depth of 9. Examples of contigs with low and absent strain variation are shown in Figure S2. We estimated θ, the product of two times the population size and mutation rate, for these contigs using methods designed specifically to work with variable-coverage metagenomic data [37]. The per-site θ under a model of stable population size was 0.049, whereas estimates of θ using a model of exponential population growth were highly variable between contigs. The likelihood of an exponentially growing population was not significantly different from that of a constant-sized population for any individual contig (likelihood ratio test). The genome-wide per-site θ calculated using Watterson′s infinite sites model and a finite sites modification [38] was 2 × 10^−4^.

Overall, the average density of polymorphisms is low in both the dominant strain and all substrains. Within regions of the dominant strain overlapping variant blocks, pN/pS = 0.43. Within all substrains, pN/pS = 0.33. dN between the dominant strain and all substrains was 0.05%, whereas for synonymous sites, dS = 0.26%, giving dN/dS = 0.18. The average SNP density within the dominant strain was calculated separately for regions overlapping defined substrains and those between substrains (“interstrain regions,” Figure 5). The mean SNP density in the dominant strain in areas overlapping substrains is 0.014%, and in interstrain regions is 0.020%. The average polymorphism density within substrains is 0.021%. Polymorphism densities within the dominant strain and in interstrain regions are not significantly different (*t*-test, *p* = 0.15). Additionally, polymorphism densities within the dominant strain and within substrain blocks are not significantly different (*t*-test, *p* = 0.12).

We calculated Tajima′s *D* [39], an estimator of whether observed polymorphism frequencies deviate from the neutral expectation (see Materials and Methods), for all main strains, substrains, and interstrain regions. No individual values of *D* were significantly different from 0. The distribution of *D* is suggestive, however. Within main strains, 43 out of 51 values of *D* were negative (binomial probability 9 × 10^−8^, probability of success 0.5) and in interstrain regions 38 of 51 values of *D* were negative (binomial probability 1 × 10^−4^, probability of success 0.5). These results suggest an excess of rare mutations in most of the genome relative to the number of segregating sites. Within substrains, there was a trend towards positive values of *D* (*D* > 0 for 16 of 31 strains with defined *D*), but this was not significant (binomial probability 0.14).

The numbers of nonsynonymous and synonymous substitutions within and between strains for a quality score cutoff of 25 are shown in Table 1. The contingency table for the MK test yielded χ^2^ = 20.932 (*p* < 0.0001), indicating that the ratios of nonsynonymous to synonymous mutations within (1.12) and between populations (0.52) are significantly divergent. The relative number of fixed nonsynonymous substitutions is smaller than the relative number of polymorphic nonsynonymous substitutions, suggesting negative (purifying) selection. Contingency tables for individual substrains with fewer than a total of ten substitutions were tested using the Fisher exact test rather than the χ^2^ test. After a sequential Bonferroni correction for multiple tests, no strain had a statistically significant difference at *p* < 0.05 between polymorphisms and fixed differences.

**Table 1:**
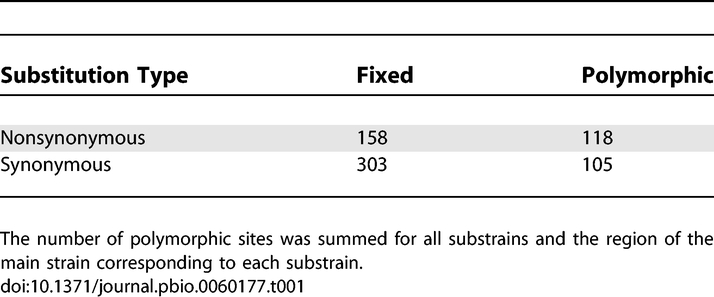
Numbers of Synonymous and Nonsynonymous Substitutions Within and Between Strains Used in the MK Test for a Sequence Quality Cutoff of 25

The Poisson random field refinement of the MK test (MKPRF) test [40] also did not find evidence that selection maintains substrains. Because this test uses segregating sites to calculate maximum likelihood estimates of shared parameters, it cannot be applied to substrains with very few segregating substitutions. Only 11 strains could be analyzed with the MKPRF test, and none of these showed statistically significant evidence for selection.

We also examined the effect of Phred quality score cutoffs and read depth on the outcome of the MK test (Table S4 and Text S1). The conclusion that purifying selection is acting on strain variant regions is robust to the effect of sequence error, read depth, and polymorphism frequency. Overall, the results indicate that genome-wide evidence of positive selection is not being masked by the presence of invalid single-copy SNPs caused by sequencing error.

## Discussion

Comprehensive analysis of diversity in complex systems has been limited by the inability to deeply sequence any of the constituent populations. The low species richness of the AMD microbial communities has allowed for nearly complete genome reconstruction for the dominant populations. In particular, *Leptospirillum* group II was sampled an average of 20 times at each genomic locus. Whereas the vast majority of sequencing reads originating from this population have been placed into one genomic path through manual curation of the assembly, we uncovered evidence for considerable population-level diversity in the form of strain variation, in part mediated by integrated elements of putative phage, plasmid, or transposon origin. Although these phenomena have been previously reported for complex environments [3,11,16], here we quantified the distribution of the variability in gene sequence and content across large regions of the genome. The high resolution of our data allowed us to test hypotheses regarding the processes that shaped this natural population.

### Recombination and Mutation

There are two possible sources of new intrapopulation sequence variation: mutation and/or immigration of new variant types, followed by recombination with members of the preexisting population. The variation may have adaptive value, may be deleterious, or may be neutral. These alternative hypotheses predict distinct patterns of variation within the *Leptospirillum* group II population.

Recombination has been widely documented in natural microbial populations [18,21,28,41–44]. We observed the signatures of recombination events among two or more closely related 5-way CG *Leptospirillum* group II strains, as well as recombination between the 5-way CG population and more distantly related *Leptospirillum* group II genome types (Figure 6). Distinct strains defined by shared SNP patterns and frequencies were apparent even when the recombination breakpoints were not directly observable (Figure 1). We were not able to manually determine recombination block length and frequency (as we did in a previous study with metagenomic data, [28]) because extremely low sequence divergence between strains prevented confident identification of transition points.

The similarity between the read depths of the observed strains (Figure S1) raises the question of how many distinct haplotypes are present in the population. It is tempting to conclude that because strains in different regions of the genome are present at similar frequencies, they must therefore belong to a single haplotype present at that frequency in the population. Short read lengths and low overall SNP density prevent us from directly linking distinct strains within the assembly, however. It will be necessary to examine whether the frequencies of these strains remain correlated in multiple samples across space and time to determine the number of distinct haplotypes present in the population.

Notably, the value of the per-site population mutation parameter θ in the 5-way *Leptospirillum* group II population is smaller than most observations of θ obtained from multi locus sequence typing (MLST) data from bacterial pathogens [45,46]. Per-site MLST estimates of θ averaged over multiple loci ranged from a low of 5 × 10^−3^ for Neisseria gonorrhoeae to 0.1 for Haemophilus influenzae, probably reflecting the more diverse populations from which alleles for MLST were drawn and the limited sampling of individual loci compared to our genome-wide estimate. The value of θ that we observe using an identical formula (2 × 10^−4^) is indicative of the overall low levels of variation in the *Leptospirillum* group II population, perhaps suggesting a relatively recent population expansion or purifying selection. We also calculated an estimate of θ using a model for metagenomic data that explicitly accounts for sequencing error in estimation, and tested whether the data fit a model of constant population size or exponential growth [37]; the fit of these models was not significantly different. Contig-based estimates of θ were consistent for a model of constant population size (0.05 ± 0.02). The only other available estimate of θ for microbial metagenomic data is for a population from an activated sludge bioreactor [37], where θ* was estimated to be 0.015 under a model of population growth. The activated sludge population was nearly clonal and had undergone recent population expansion prior to sampling. The slightly higher value of θ we observe in *Leptospirillum* group II using this method is indicative of a larger amount of standing variation than was present in the sludge population.

### Selection on Closely Related Strains

Given the clear evidence that multiple closely related strains coexist in the *Leptospirillum* group II population, the key question is whether this variation results in differential fitness between strains. If strains are maintained because each is maximally fit in a different ecological niche, we expect to see evidence of positive or balancing selection in variant regions. This expectation underlies the ecotype or periodic selection concept, which posits that distinct sequence clusters in microbial populations correspond to ecologically separate units [47]. The “Stable Ecotype Model” [5] suggests that diversity within separate clusters is periodically purged by selection, and distinct clusters persist due to the low frequency of intercluster recombination. Permanent divergence only arises if a mutation allows an individual of one ecotype to colonize a new niche [5]. This model suggests that selection maintains coexisting clusters, and that within-cluster diversity is low. Under this scenario, we expect to see an excess of fixed nonsynonymous differences between strains, as these amino acid changes would indicate niche-specific adaptations. Alternatively, if sequence variation is not maintained by positive selection, we expect to find evidence for neutral or negative selection in these regions. We tested these alternative hypotheses for the coexistence of closely related strains using the MK test for polymorphism and divergence [36]. This test posits that under a model of neutral evolution, the ratio of nonsynonymous (replacement) to synonymous substitutions within a population is the same as the ratio of replacement to synonymous fixed differences between populations. An excess of replacement fixed differences indicates positive selection, whereas a dearth indicates negative selection.

Our results from MK tests on regions of strain variation indicate strongly that positive selection on amino acid substitutions does not separate coexisting strains. We observe instead a significant deficiency of fixed replacements relative to polymorphic replacements, indicating that negative (purifying) selection is acting to remove deleterious nonsynonymous mutations differentiating closely related strains. This result is robust to the effect of sequencing error in SNP estimation and the use of low-frequency (nonreplicated) polymorphisms (Tables S2 and S4, and Text S1). These additional analyses clearly demonstrate that an excess of low-frequency polymorphisms due to sequencing error is not masking evidence of genome-wide positive selection. Recent theoretical results indicate that an abundance of slightly deleterious polymorphisms can substantially mask the presence of adaptive evolution [48]. However, in that case, one would expect the average allele frequency of nonsynonymous polymorphisms to be significantly lower than that of synonymous polymorphisms [48], which we do not observe (average synonymous site frequency in dominant strain, 0.14; average nonsynonymous site frequency, 0.16). Our results therefore indicate that this population does not fit the predictions of the stable ecotype model, and suggests that strains of 5-way CG *Leptospirillum* group II detected by sequence variation may occupy a single niche within the biofilm environment. These results are also consistent with the strong purifying selection acting on most genes in the coexisting archaeal genus *Ferroplasma*, despite a far more extensive mosaic genome structure than that of *Leptospirillum* group II [15].

Our results also rule out an alternative to the stable ecotype model, in which high recombination rates prevent periodic selective sweeps from purging diversity within strains. In this scenario, strain variant regions contain beneficial mutations decoupled from the remainder of the genome. This hypothesis—that recombination speeds adaptation by spreading beneficial variants throughout the population—again posits that these variant regions are under positive selection. Thus, it predicts a detectable signature of excess fixed amino acid replacements in recombinant regions and distinct patterns of polymorphism frequency between regions containing adaptive variants and the remainder of the genome. Results from MK tests rule out positive selection on strain variant regions. The observed patterns of polymorphism density also do not match the second prediction of the high recombination model. We do not observe a significant difference in polymorphism density between reads within substrain blocks, reads within the main strain, and reads in interstrain regions, as would be expected if the action of selection on these regions was decoupled due to high recombination rates.

Although the substrains within the 5-way CG population do not appear to be under selection themselves, it is plausible that that linkage between them is absent due to selective sweeps eliminating variation in intersubstrain regions. This hypothesis predicts a lower polymorphism density in intersubstrain regions than within substrains, since a selective sweep would carry hitchhiking neutral variants to fixation. Again, the observation that polymorphism density within substrain and interstrain regions is not significantly different is strong evidence against this hypothesis. The interblock selective sweep scenario therefore requires a higher mutation rate in intersubstrain regions, which would have allowed polymorphisms to accumulate after a selective sweep to the same levels observed in adjacent substrain regions. Such extreme spatial variation in genomic mutation rate is unlikely, and hence, we can also rule out the “interstrain” sweep model.

An alternative explanation for the observed patterns of polymorphism and divergence is that a few sites within each substrain have experienced positive or balancing selection, while all other neutral polymorphisms in the substrain are hitchhiking to higher frequency along with the selected sites. Such a signal might not be detectable using MK polymorphism-divergence tests. This scenario requires that recombination has not yet separated the adaptive site from linked neutral sites. If the mutation occurred in the distant past, the recombination rate would have to have been extremely low relative to the mutation rate to preserve the well-defined substrain blocks observed here. Because there is evidence for significant recombination, this scenario seems unlikely. If the positively selected mutation occurred recently, it would be in the process of sweeping to fixation along with linked neutral polymorphisms. Under this selective sweep scenario, we expect low levels of polymorphism in the region associated with the adaptive mutation relative to the intersubstrain region (Figure 5). This would manifest as a lower polymorphism density in substrain sequences relative to interstrain sequences, which was not observed.

Of the three deeply sampled recombinant regions between CG-type and other *Leptospirillum* group II types (Figure 1), two do not show significant evidence for either positive or negative selection. The third shows strong evidence for purifying selection, consistent with our results for recombinant 5-way CG strains. These regions do not, therefore, appear to be an exception to the general pattern of purifying selection in recombinant blocks.

The most likely explanation for the existence of blocks of closely related variant sequence is geographic separation of two or more ancestral strains, followed by subsequent mixing and recombination. The mosaic genome structure of *Ferroplasma* is also consistent with this model [15]. The high read depth and connectivity of the dominant strain compared to the smaller fragments of alternate sequence type (Figure 5) suggest that a small number of individuals from one or more strains migrated into a preexisting population. Genetic exchange likely occurred between the immigrants and the existing strain, as shown by the incorporation of 1- to 10-kb fragments, but was insufficient to erase the genomic signature of the existing strain. It is possible that this strain was better adapted to its environment than immigrant strains, and retained a selective advantage, which explains its higher frequency in the present population. For a migration/recombination model to be plausible, however, we need to determine whether the observed divergence between the dominant strain and immigrant substrain fragments is consistent with a geographic separation scenario, given the geologic history of the Richmond Mine.

Assuming that the observed divergence between strains is typical of ancestral haplotypes, and a mutation rate of 10^−9^ per site per generation [49], the ancestral strains had to be separated for at least 8 × 10^5^ generations to accumulate the observed divergence. This translates into a separation time of at least 1,400 y, given a minimum generation time of 15 h [50]. The Richmond ore body was exposed to weathering for 780,000 y prior to the onset of mining operations around 150 y ago [51]. A physical separation of the strains of order 10^3^ y is therefore possible. Assuming that the two strains were reunited 150 years ago (at most about 100,000 generations), the observed level of variation would be maintained, provided that the effective population size (*N*_e_) is sufficiently large that the original variation would not all drift to fixation in this time (*N*_e_ at least the same order of magnitude as the number of generations). If we postulate that the effective population size of *Leptospirillum* group II is at least of this order of magnitude (plausible, given 10^8^ cells per cm^3^ in the biofilm), it is certainly possible that the two strains could have comingled as the result of increased connectivity within the mountain due to mining.

In summary, the combination of assumptions required to fit alternative explanations to the data makes the simpler explanation of geographic separation and subsequent remixing more likely.

### Genome-Wide Spatial Patterning of Recombination

Substrains are distributed unevenly around the genome in a mosaic of 1–10-kb fragments (Figure 1). Both SNP density and minor allele frequency appear to be higher on both sides of the origin of replication (Figure 1) rather than the origin of transfer, suggesting the preferential incorporation of novel genetic material in this region. A similar pattern was observed in an environmental population of Ferroplasma acidarmanus, fer1 and fer2, in which 77% of interpopulation recombination events clustered within 500 genes of the origin of replication [28].

The mechanism responsible for this symmetry is not known, but would seem to indicate a higher probability of recombination events associated with newly initiated replication forks proceeding bidirectionally from the origin of replication. It is well known that recombinational intermediates can be converted into functional replication forks (reviewed in [52]). Replication forks resulting from recombination of introduced fragments could interact with replication forks initiated at *oriC*, resulting in the duplication of a chromosome containing the introduced fragment, but this process is not well understood [52]. Another example of genomic symmetry across the replication axis involves large- and small-scale inversions in closely related bacterial species [53,54]. The reason for this is thought to be selection against disruption of polarized motifs clustered symmetrically near the terminus of replication and important for chromosome replication and segregation [55]. Perhaps recombination events closer to the terminus of replication are counterselected due to interference with these motifs, resulting in an apparent clustering of events around the origin. A breakdown in synteny between related strains further from the origin is evoked as an explanation for symmetry around the origin of replication in *Ferroplasma* [28], but the lack of major genome rearrangement events observed in our assembly makes it less likely as an explanation for the 5-way CG *Leptospirillum* group II population.

The presence of a large integrated plasmid within the *Leptospirillum* group II population suggests conjugation as a possible mechanism of genetic transfer between individuals. This plasmid region contains genes coding for proteins in the *tra* and *trb* loci, which are known to be involved in formation of a bridge between two cells through which chromosomal DNA transfer can occur. In Escherichia coli Hfr strains, transfer is initiated at the *oriT* site and proceeds in the 5′ to 3′ direction. Marker genes closer to the *oriT* site are transferred more frequently due to the increased probability of breakage of conjugation bridges with time. Therefore, if conjugation were the primary mechanism of transfer, we would expect to see more incorporation of novel fragments in 5-way CG *Leptospirillum* group II close to, and on one side of, the putative *oriT* (sequence and polarity not known). Despite uncertainty about how the block containing the origin of replication is linked to the block containing the *oriT*, we can evaluate both of these considerations to some extent. The incidence of recombined substrain blocks is higher clockwise from the integrated plasmid region than counterclockwise from it, except immediately adjacent to the *oriT*. Selection against recombined regions carrying variants of conserved proteins (e.g., a large region encoding ribosomal proteins) may have obscured this pattern to some extent.

The short recombinant fragments observed both in this study and in *Ferroplasma* [28] are strikingly similar to those observed among strains of E. coli. Milkman and colleagues [56–58] showed that E. coli chromosomes typically contain distinct sequence blocks (size range around 1 kb) embedded in larger regions that have overall high similarity to other strains. A subsequent statistical comparison of six whole *E coli* genome sequences also supports a mosaic structure, albeit with shorter fragment size (50% < 1 kb, 80% < 2 kb [59]). These segments are an order of magnitude less than the average length observed in individual conjugation (48–105 kb) or transduction (10–32 kb) events [57]. The degree of fragmentation of donor DNA was determined by differences in donor–recipient restriction-modification (RM) systems [57,60].

The clustered pattern of small segments that we observe is consistent with the idea that large pieces of DNA are transferred by conjugation or transduction between closely related strains, fragmented, and then incorporated as a series of small segments [56]. The fragmentation likely occurred through the action of restriction enzymes. It is interesting that most genes annotated as part of RM pathways in *Leptospirillum* group II are found in strain variant regions, suggesting that restriction specificity varies among closely related organisms (Table S1). A similar observation was made for an environmental *Ferroplasma* population [15]. Thus, the diversity of RM systems within the 5-way CG population could explain the generation of small DNA fragments from DNA introduced from closely related strains.

### Gene Content Variation

In addition to variation in sequence composition, variation in gene content could lead to an increase in fitness, be neutral, or be deleterious. As shown from between-strain genomic comparisons of isolates [2,7–13] and population analyses [15], a high fraction of individuals in a population contain unique regions due to phage, plasmid, or transposon insertion. Community genomics identifies the laterally transferred regions that are present population-wide in addition to those present at low frequency at the time and conditions of sampling, and also provides information about the degree of gene content variation in these regions.

Our data suggest that the majority of the genes on strain variant paths are present in low copy number in the population. Most observed alternate genome paths are short (a single read length, or about 1 kb; Figure 2) but incomplete. This could be in part because the paths are undersampled and in part because the insertions are on the scale of a single gene. The shallow coverage, but high density, of these variants suggests that there is a large pool of low-frequency genotypes from which the observed variants are drawn. Given that 118 alternate paths (an average length of 1 kb spanned by approximately two reads per variant) are observed in a 500-kb segment, and that there are on the order of 42,000 reads in the main genome path, the observed frequency of variant reads is approximately 3%. The variants thus span approximately 20% of this 500-kb region. If the genome of every cell in the biofilm was 20% different from every other cell, and all were present at the same frequency, we would expect the frequency of unique reads to be 20%. Therefore, a few types are present at high frequency, while the majority of variants are present at very low frequencies. The minimum number of genome types occurs when each of these low-frequency types only occurs in one cell (combinatorial variation would greatly increase the number of possible genome types). Based on the observed frequency of variant reads, low-frequency variants comprise approximately 15% of the total population. Based on earlier estimates of approximately 10^8^ *Leptospirillum* group II cells in the sample used for sequencing [21], there are a minimum of approximately 10^7^ genome types present. The true frequency is probably somewhat higher as not all possible strain paths were counted in the assembly process. This finding of a very heterogeneous genome pool suggests that the population has not been strongly shaped by recent pervasive selective sweeps unless the genotypic variants are generated at a rate that is high compared to the frequency of such sweeps.

The high estimated number of coexisting genome types is fairly consistent with estimates based on genotyping of isolates from a complex system [11]. Importantly, however, the majority of the cells in the population share the majority of their genomes, which is what makes reconstruction of a composite genome possible. Based on their low frequency, the functional significance of these highly variable regions is unclear. Tracking of strain population structure over time may help to resolve the extent to which different genotypes represent distinct adaptations.

It is notable that many of the strain variant regions present at higher abundance in the population (reconstructed alternative genome paths listed near the end of Table S1) encode genes typical of plasmids (e.g., trwB, traA, traB, and mobD) or viruses (e.g., integrases). Of those integrated elements present at high frequency, some encode genes of potential functional significance. A case in point involves a region where most individuals carry one or more genes of the LuxIR system, involved in quorum sensing [61], whereas only a minor part of the population encodes the complete pathway (Figure 4).

High-frequency variants, which are often associated with marked variation in gene content (such as the LuxIR region), could be the result of multiple processes: (1) a mobile element encoding multiple genes inserts into one genome, and that type increases in frequency; (2) the variable loss of genes from mobile elements results in different individuals with differences in gene content; (3) a mobile element integrates into different individuals at the same site, localized by some genomic feature (e.g., tRNAs); and (4) a mobile element integrates at a single site in one individual, followed by an increase in frequency of that genome type, followed by recombination of related but distinct elements in or near the insertion site. For example, closely related viruses derived from a diverse viral population could use the initial virus insertion as a locus for homologous recombination. This could result in a high diversity of genome types at the altered locus.

High-frequency variants also result from recombination between related individuals followed by an increase in frequency of individuals carrying the recombinant block. A notable example of functionally significant genes on a recombinant block is the block of UBA genes containing a CRISPR system, including CRISPR-associated (*cas*) genes (Figure 7). Recent experimental work with Streptococcus thermophilus demonstrated that CRISPRs confer resistance to viral infection [35]. The CRISPR locus contains spacers approximately 30 bp long that match predominantly viral sequence, separated by repeats of similar length and adjacent *cas* genes inferred to be the protein machinery of the acquired resistance system (recently reviewed in [62]).

Results of the current study suggest that a block containing CRISPR genes recombined from the UBA population into the 5-way CG population in a single event (Figure 7A) and subsequently increased in frequency. The region of this UBA-type block containing the *cas* genes has risen to 100% frequency in the population (genes 28–36 in Figure 7C), whereas the first half of the UBA block co-occurs with cells containing sequence of the 5-way type (genes 1–26 in Figure 7C). A region containing phage/transposon insertions separates the two halves of the recombination block. The absence of any fixed differences between the *cas* locus in the UBA genome and the UBA-type integrated *cas* locus in the 5-way CG population suggests that this was either a very recent event and/or that strong purifying selection is acting to eliminate variation. Evidence from previous work indicating very rapid diversification of the spacer complement supports the first explanation but does not rule out the second [63]). The observation that the genome type carrying the CRISPR locus has apparently rapidly risen to high frequency in the population suggests that viral resistance conferred a selective advantage to the recipient types.

### Conclusions

Deep sequencing of the 5-way CG *Leptospirillum* group II population has given us both an unprecedented catalog of within-population variation and the opportunity to test hypotheses relating to the origin and maintenance of this variation. This opens a route for incorporating population genomic evidence into reconstruction of the natural history of the Iron Mountain microbial ecosystem. Very few metagenomic studies of microbial communities have applied the tools of population genetics to understand the origin and maintenance of sequence-level variation (e.g., [15,37]). In the present study, we translate community genomic data into a form suitable for one such test [36] by separation and comparison of genomic regions varying in their SNP composition. We show that, contrary to many models of microbial population structure, extensive strain variation in *Leptospirillum* group II is not maintained by positive selection for adaptive variants. Instead, we detected strong evidence for purifying selection in strain variant regions. The data support a model of geographic isolation, mixing, and extensive recombination, a scenario consistent with the geological history of Iron Mountain. We believe that the application of population genetics to metagenomic data, still a nascent field, has the potential for many more such insights into the processes governing microbial population structure.

In addition to variation at the nucleotide level, our data present a snapshot of ongoing genetic exchange in a natural population, including recombination and the rapid uptake and loss of plasmid and phage-derived genes. Most gene-content variants are present at low frequency, consistent with a very large number of coexisting genotypes. This suggests a dynamic process involving the continual generation and loss of gene-content variants. Very few variant regions have reached high frequency in the population, but two examples documented here involve regions of potential functional importance (LuxIR, involved in quorum sensing, and CRISPR, involved in viral resistance). Despite the high number of variants, the genome structure of the majority of population members is sufficiently similar to allow for assembly of composite genome paths. The functional significance of this extensive gene-content variation remains unclear. To provide an answer to this question, it will be important to examine the expression levels of gene variants across space and time, as well as examine signatures of selection in genes present in regions with high gene-content variation (such as the LuxIR region discussed above).

Due to the effect of strain variation on downstream postgenomic approaches such as proteomics [64], a thorough documentation of the population-level diversity in community genomic datasets is of great importance. Current technologies and methods have progressed to strain-level resolution, allowing the discrimination of closely related protein variants [18]. Databases that include population-level strain variant sequences will allow us to track the presence and activity of these variants over time and as a function of geochemical conditions. These types of studies will allow us to determine the functional importance of variation within natural populations.

## Materials and Methods

### Biofilm samples.

Genomic sequence was obtained from DNA extracted from a pink biofilm growing on a pH 0.83, 42 °C acid mine drainage stream and sampled from the 5-way location within the Richmond Mine, California, in March 2002. Library construction, sequencing, and the draft assembly methods used to analyze the first 76 Mb of data were reported previously [21]. We obtained an additional 54 Mb of sequence from a second 3-kb library constructed from the same sample and reassembled the data for the analyses reported here.

### Population genomic assembly and functional annotation.

The 130 Mb of DNA sequence (GenBank accession number AADL00000000) was assembled using phredPhrap (P. Green, http://www.phrap.org) with parameters chosen to maximize assembly extent (e.g., to allow coassembly of sequences with some SNPs) while minimizing assembly errors. Parameters used were “-minmatch 50 -minscore 50 -penalty −15 -revise_greedy.” Typically, all reads deriving from the *Leptospirillum* group II population were incorporated at a single genomic locus, except in the presence of strain-specific gene insertions or deletions. However, as noted previously, the dataset contains a very small number of reads with very high sequence identity (∼100%) to another *Leptospirillum* group II type (reconstructed from a sample from the UBA location in the Richmond Mine [18]). Reads from this organism (distantly related strain or closely related species) share approximately 94% average nucleotide-level sequence identity, and were brought into assemblies occasionally.

All contigs were manually curated using Consed [65] to eliminate assembly errors and to resolve regions of strain heterogeneity. The set of contigs assigned to *Leptospirillum* group II, initially based on GC content and depth, was augmented with new contigs that could be confidently linked into the assembly through mate pairs anchored into unique sequence. In a subset of cases, alternative paths resulting from insertion or deletion of genes or sequence divergence result in fragmentation of assemblies. However, strain-specific fragments can be linked into the main genome path via mate pairs from one or both ends of the strain-specific contig. The outcome of reassembly, new data, and manual analysis is much larger genome fragments than achieved via the JAZZ assembly [21] and some differences in gene order. The assembly order is shown in Table S1.

ORF positions were determined and refined based on previously reported gene calls [21] as well as the manually curated annotation of the closely related *Leptospirillum* group II organism assembled from the UBA community genomic data [18]. Functional annotation relied upon on the manually curated annotation of *Leptospirillum* group II from the UBA genomic dataset (D. S. Aliaga Goltsman, V. J. Denef, S. W. Singer, N. C. VerBerkmoes, M. Lefsrud, unpublished data). Contigs generated in the assembly are a composite sequence, and thus do not show SNPs present at a subset of loci.

### SNP identification and strain construction.

For analysis of sequence variation, the final Phrap contigs and aligned reads were imported from Consed into the program Strainer [66]. Substrains consisting of linked polymorphisms were defined for all large Phrap contigs. A substrain was defined heuristically to consist of two or more linked polymorphisms with Phred scores greater than 20. These were considered to be separate subpopulations from the dominant strain due to the improbability of multiple linked polymorphisms arising simultaneously due to mutation alone. For strain grouping analyses, the composite sequence was corrected to reflect the dominant polymorphism pattern so long as these changes retained SNP grouping defined by reads and their mate pairs. Sequences were grouped based on the presence of SNPs in more than one read (considering only high-quality base calls, with Phred scores > 20).

Additionally, custom Perl scripts were developed to identify and classify every polymorphic site in the assembly as synonymous, nonsynonymous, intergenic, or indel. SNPs were classified as replicated or nonreplicated. These counts are summarized in Table S2 and were used to generate Figure 1 for a Phred quality score cutoff of 25. The effect of different quality score cutoffs on the distribution of these classes was also examined (Table S2 and Text S1). In a region spanning approximately one quarter of the genome, indel SNPs were examined in detail for their effect on frameshifts and gene splits (Figure 2 and Table S3). The location of divergent UBA-type sequences was also mapped for this region.

### Population genetic analysis.

For each substrain consisting of at least four reads, we counted the synonymous and nonsynonymous (replacement) substitutions both within the substrain and between the substrain and the dominant composite sequence (Figure 5). A custom Perl pipeline was developed to extract aligned reads and a consensus sequence for every gene in the Phrap assembly. Bases with Phrap quality scores less than 25 were masked. This threshold approach treats all bases with higher quality scores as “true” and does not take into account the error probabilities associated with these scores, resulting in a somewhat inflated estimate of the true number of SNPs in the population [37]. Because substitutions at roughly two-thirds of all sites will result in nonsynonymous substitutions, we would expect a bias towards nonsynonymous SNPs due to sequencing errors. To address this question, we repeated the analysis with different levels of sequence quality score cutoffs (Table S4). Additionally, we examined the effect of read depth on all analyses (see Table S4 and Text S1). Due to common sequencing errors at read ends and the presence of inserted genes, reads diverging from the consensus by more than 2% were eliminated from the analysis. This cutoff also eliminated reads derived from the UBA-type *Leptospirillum* group II. The two short, deeply sampled regions containing multiple UBA-type reads were analyzed separately in comparison with syntenous CG-type reads.

Additional custom Perl scripts were used to calculate synonymous and nonsynonymous polymorphisms within each strain and substrain. Substitutions occurring immediately adjacent to masked bases were not counted due to the high probability of alignment or sequencing errors in these regions. The consensus sequence determined by Phrap was used to determine synonymous and nonsynonymous fixed differences between each substrain and the main strain. The number of synonymous and nonsynonymous sites in each strain was calculated using the program codeml in the PAML package [67].

The numbers of polymorphisms and fixed substitutions were used to test the hypothesis that substrains are maintained in the population through selection for adaptively significant variants. Two polymorphism-divergence tests were used: the MK test [36] and the MKPRF test [40]. For the purpose of these tests, each substrain is considered to be a distinct population (note that this differs from the terminology used elsewhere in this paper). The rationale behind the MK test is that in the absence of selection, the number of synonymous and nonsynonymous fixed differences between two populations, and the number of synonymous and nonsynonymous polymorphisms within each population, depend only on the mutation rate and time since divergence. The ratio of nonsynonymous to synonymous polymorphisms within populations should therefore be the same as the ratio of nonsynonymous to synonymous fixed differences between populations. Positive selection for nonsynonymous mutations will produce an excess of nonsynonymous fixed substitutions relative to nonsynonymous polymorphisms. The MKPRF test refines this idea, using a population genetic model to explain the distribution of within-population variation via maximum likelihood estimation of mutation rate, divergence time, and a selection parameter. Polymorphism-divergence data were combined for all genes within a strain for the MK and MKPRF tests, whereas individual genes were also analyzed using the MKPRF test. The MKPRF tests were carried out using an online implementation (Computational Biology Service Unit, Cornell University).

In addition to the MK test, we also calculated several summary statistics for each strain and substrain as well as the entire assembly. Because of variable coverage depth across the assembly, it was necessary to weight these parameters by the fraction of total sites at a given depth [68]. The parameter θ = 2*N*_e_ *u*, an estimator of the product of population size and mutation rate for a neutrally evolving population, was calculated with Watterson′s infinite sites estimator [69] and a finite sites modification [38]. Estimates for each coverage class were combined to yield a single value. The program “piim” [37], which corrects for sequencing error in the estimation of population genetic parameters from metagenomic data, was also used to calculate θ for the entire assembly. Pairwise heterozygosity (π) was calculated as described for a variable coverage assembly of Drosophila simulans [68] using custom scripts. Under a neutral model of evolution for a Wright-Fisher population, π and θ are expected to be equal. Tajima′s *D* measures the discrepancy between these statistics: *D*_T_ = (π – θ)/*C*, where *C* is a normalizing constant calculated from the data [39]. The statistical significance of *D* depends on the sample size, but roughly, for sample sizes greater than 7, *D* is considered significant at the 95% level if it is smaller than −2 or larger than 2 [39]. *D*_T_ for strains, substrains, and interstrain regions was calculated with BioPerl population genetics modules [70].

## Supporting Information

Figure S1 Frequencies of Strains Observed in the 5-Way CG *Leptospirillum* Group II Population Frequencies were calculated from the average allele frequency for each polymorphism occurring in a strain relative to the dominant population. Strains are sorted in ascending order of allele frequency. Error bars show ± 1 standard deviation. Each point represents one strain.
(158 KB DOC)

Figure S2 Diagram Showing Low Strain Variation in Two Contigs in Different Regions of the Genome Images were derived from the Strainer program [66]. White bars represent sequencing reads, linked by thin white lines to their mate-paired reads. Dark shading on reads indicates the presence of SNPs. Colored boxes indicate reads that have been grouped together based on sequence similarity using SNPs that are replicated in more than one sequencing read. Numbers indicate the number of SNPs used to distinguish different variants (grouped reads surrounded by colored boxes) for cases in which variant types are defined by more than one SNP.
(A) Diagram showing strain variation over contig 11067 (54 kb). Variant types (colored boxes surrounding reads) were defined based on SNPs replicated in more than one read. This region contains 15 replicated substitutions (mustard-colored variant: ten, green: two, blue: two, and olive: one).
(B) Contig 10961: an area distant from the origin of replication. Only three strain groups were defined, and each contains only one replicated SNP.
(153 KB DOC)

Table S1 *Leptospirillum* Group II 5-Way CG Genome Reconstruction The reconstruction includes the gene order and functional annotation, and shows relationship of the genome fragments to the previously reported draft assembly.
(37 KB PDF)

Table S2 Classification of All SNPs Observed in the 5-Way CG *Leptospirillum* Group II Assembly Increasing Phred quality score cutoffs were used to examine the effect of sequencing error on the distribution of SNPs between site classes.
(11 KB PDF)

Table S3 Click here for additional data file. List of the SNPs in Approximately One Quarter of the Genome in a Region Inferred to Span the Origin of Replication
SNP type (synonymous, nonsynonymous, frameshift/split, or intergenic) is also shown.
(56 KB PDF)

Table S4 The Results of MK Tests for Phred Quality Score Cutoffs MK tests using quality score cutoffs of 20 and higher give nearly identical results, as indicated here by the similarity in ratios of segregating nonsynonymous to silent sites and fixed nonsynonymous to fixed silent sites for cutoff scores higher than 20. The use of replicated SNPs only or sites at higher coverage levels only also does not affect the outcome. An asterisk (*) indicates the expected ratio of segregating nonsynonymous to segregating sites if two-thirds of masked sites are nonsynonymous and one-third are silent. The effect of increasing the minimum required coverage depth for a quality score cutoff of 25 is shown in the bottom three rows.
(36 KB DOC)

Text S1 Supplementary Results (1) The effect of sequencing quality and read depth on SNP identification and analysis and (2) Frameshift variation in *Leptospirillum* group II.
(37 KB DOC)

## Abbreviations

AMD: acid mine drainage
CRISPR: clustered, regularly interspaced short palindromic repeat
MK: McDonald-Kreitman
MKPRF: Poisson random field refinement of the McDonald-Kreitman test
SNP: single nucleotide polymorphism
