# Supplementary material for: Population Genomic Analysis of Strain Variation in Leptospirillum Group II Bacteria Involved in Acid Mine Drainage Formation
Source: PLoS Biol. 2008 Jul 22;6(7):e177. doi: 10.1371/journal.pbio.0060177 (PMC2475542; doi:10.1371/journal.pbio.0060177)
Supplement: Supplementary file 1 [file pbio.0060177.sd001.pdf]

Text S1

Effect of sequence quality and read depth on SNP identification and analysis

We analyzed the effect of increasing Phred quality score cutoffs (**q**) on the number and distribution of SNPs in the 5-way *Leptospirillum* group II assembly, as well as on the outcome of the McDonald-Kreitman analysis. The quality score is a reflection of the error probability associated with the base call, and is defined as **q** = -10*log(p), where p is the error probability [1]. The cutoff approach means that all sites above a given **q** are treated as true, while all sites below **q** are ignored. If sites are masked with increasing **q** in an unbiased fashion, we expect approximately 2/3 of masked sites in coding regions to be replacement and 1/3 of masked sites to be silent.

Using custom Perl scripts, we tabulated the number of replicated and nonreplicated synonymous, replacement, intergenic, and indel polymorphisms in the entire genome using **q** values of 20 to 50 (Table S2), representing error probabilities of 10^{-2} to 10^{-5}. The ratio of replacement to synonymous polymorphisms was relatively constant for this range (0.84-0.89), consistent with near-random loss of synonymous and replacement sites due to base calling errors. Indels were the polymorphism type most likely to be caused by sequence error, as shown by the decrease in their fraction of total polymorphisms with increasing **q**. The overall density of polymorphisms decreased from 0.12% for **q**=20 to 0.06% for **q**=50. As expected, the fraction of total SNPs present in more than one read (“replicated”) increased with increasing **q**, from 32% at **q**=20 to 47% at **q**=50.

We also tabulated the number of silent and replacement sites present in regions analyzed in the McDonald-Kreitman test at **q** values from 0 to 50, representing error probabilities of 1 to 10^{-5} (Table S4). We calculated the number of sites masked by each increase in **q**. We then calculated the expected number of masked replacement and silent sites relative to the previous cutoff score level, and the corresponding expected replacement/silent ratio. The observed ratio of segregating replacement to silent sites became congruent with this expected ratio at **q** = 25. We chose this cutoff score for all further analyses described in the main text, as it indicated that the loss of sites from **q**=20 to **q**=25 conformed to expectations of random sequence error. Additionally, the total number of sites masked from **q**=20 to **q**=25 was an order of magnitude

lower than the number of sites masked from **q**=10 to **q**=20. Overall, increasing the cutoff score primarily affected the total number of segregating sites and had only a minor effect on the total number of fixed sites, as most of these have high **q** values. The ratio of fixed replacement to fixed silent sites was stable for quality score cutoffs of 20 and higher, as was the ratio of segregating replacement to silent sites. Hence the outcome of the MK test (evidence for an excess of replacement segregating sites relative to replacement fixed sites) was the same in all cases.

It has been suggested that nonreplicated SNPs (present only in 1 read) are more likely to be due to sequencing error, and that only replicated SNPs should be considered in analyses of this type. Limiting MK tests to higher-frequency polymorphisms has also been suggested to decrease bias in the detection of adaptive evolution [2]. We examined the effect of using only replicated SNPs (both alleles present in 2 or more reads) on the MK test with a quality score cutoff of 25 (Table S2). The total number of sites was reduced by a factor of 0.7, consistent with the overall fraction of replicated SNPs in the community genome. Nonetheless, the ratio of segregating replacement/silent sites (1.54) was still significantly different from the ratio of fixed replacement/silent sites (0.53), consistent with the analysis including both replicated and nonreplicated SNPs. We also examined the effect of requiring minimum coverage depths of all polymorphic sites on the outcome of the MK test. Again, although the total number of sites considered was smaller, the outcome of the MK test was the same when only polymorphic sites with coverage greater than 3 or greater than 4 were considered (Table S4).

Overall, these results indicate that genome-wide evidence of positive selection is not being masked by the presence of invalid single-copy SNPs caused by sequencing error.

Complete assembly and annotation

The complete assembly of the 5-way *Leptospirillum* group II population and associated annotations for each gene are reported in Table S3. These annotations and corresponding gene sequences are also available under Genbank accession number AADL00000000. A list of high-quality SNPs (**q**>40), their type, corresponding annotation, and the location of UBA-type recombinant regions in a ~700 kb region surrounding the origin of replication are reported in Table S3.

Phase variation in *Leptospirillum* group II

The regulation of gene expression by reversible frameshifts ("phase variation") is a well-documented mechanism for heritable phenotypic alterations in bacteria, and these shifts typically occur at higher frequency than typical single nucleotide mutations [3]. The majority of documented examples involve structures on the cell surface involved in host-bacterial interactions (e.g. [4]) and virulence [5]. A common mechanism for phase variation involves regions of short sequence repeats of 2-7 nt (SSR), which can induce frameshifts via slipstrand mispairing during replication. Mispairing can cause a change in the number of unit repeats, and is observed both upstream of genes (transcriptional regulation) and within coding sequence (translational regulation) [3]. In both *Burkholderia mallei* [4] and *Salmonella enterica* serovar Typhi [5] a significant number of pseudogenes were generated by differences in SSR copy number from closely related orthologs.

The prevalence of frameshift mutations in natural populations is largely unknown, although results from comparisons of closely related strains suggest it may be an important mechanism of phenotypic variation [4-7]. We found that 13% of all high-quality SNPs documented in a 700 kb region of the 5way *Leptospirillum* group 2 population resulted in frameshifts or splits (Table S2). The majority of these (70%) arose due to single base pair alterations in SSR regions of 2-7 nt. Most were either 2 nt (20 instances) or 4 nt (9 instances) in length, and the longest SSR observed was 7 nt (2 instances). Ten of the 67 frameshift mutations occurred in more than one read, suggesting they did not arise from sequencing error. Replicated frameshift mutations occurred in genes for two proteins of unknown function, two hypothetical proteins, a probable thiamine biosynthesis protein (ThiI), glycerol 3-phosphate dehydrogenase (NADP), a thiazole biosynthesis protein (ThiG), a putative diguanylate cyclase/phosphodiesterase, and a putative cytochrome c biogenesis protein. It is notable that two of these genes (ThiI and ThiG) are involved in thiamine biosynthesis. *Leptospirillum* group II possesses a functional operon for thiamine synthesis, and thiamine biosynthesis proteins have been detected using proteomics (Goltsman et al. in prep). However, the current population genomic analyses indicate that this function may be attributed to only a subset of population members. Knockout mutants of ThiG in *Bacillus subtilis* [8] and ThiI in *Salmonella typhimurium*

[9] are thiamine auxotrophs, suggesting that *Leptospirillum* group II may regulate thiamine production by frameshifts in these genes.

Supplementary References

1. Ewing B, Green P (1998) Base-Calling of Automated Sequencer Traces Using Phred. II. Error Probabilities. Genome Res 8: 186-194.
2. Charlesworth J, Eyre-Walker A (2008) The McDonald-Kreitman Test and Slightly Deleterious Mutations. Mol Biol Evol: msn005.
3. van der Woude MW, Baümler AJ (2004) Phase and Antigenic Variation in Bacteria. Clinical Microbiology Reviews 17: 581-611.
4. Nierman WC, DeShazer D, Kim HS, Tettelin H, Nelson KE, et al. (2004) Structural flexibility in the Burkholderia mallei genome. Proc Natl Acad Sci U S A 101: 14246-14251.
5. Parkhill J, Dougan G, James KD, Thomson NR, Pickard D, et al. (2001) Complete genome sequence of a multiple drug resistant *Salmonella enterica* serovar Typhi CT18. Nature 413: 848-852.
6. Gogol EB, Cummings CA, Burns RC, Relman DA (2007) Phase variation and microevolution at homopolymeric tracts in *Bordetella pertussis*. BMC Genomics 8: 122.
7. Thomson NR, Yeats C, Bell K, Holden MTG, Bentley SD, et al. (2005) The *Chlamydophila abortus* genome sequence reveals an array of variable proteins that contribute to interspecies variation. Genome Res 15: 629-640.
8. Perkins JB, Pero J (2002) Vitamin Biosynthesis. In: Sonenshein AL, Hoch JA, Losick R, editors. *Bacillus subtilis* and its closest relatives: ASM Press. pp. 271-286.
9. Webb E, Claas K, Downs D (1997) Characterization of thiI, a new gene involved in thiazole biosynthesis in *Salmonella typhimurium*. J Bacteriol 179: 4399-4402.
10. Eppley JM, Tyson GW, Getz WM, Banfield JF (2007) Strainer: Software for analysis of population variation in community genomic datasets. BMC Bioinformatics 8.
