# Supplementary material for: Population Genomic Analysis of Strain Variation in Leptospirillum Group II Bacteria Involved in Acid Mine Drainage Formation
Source: PLoS Biol. 2008 Jul 22;6(7):e177. doi: 10.1371/journal.pbio.0060177 (PMC2475542; doi:10.1371/journal.pbio.0060177)
Supplement: Supplementary file 2 [file pbio.0060177.sg001.pdf]

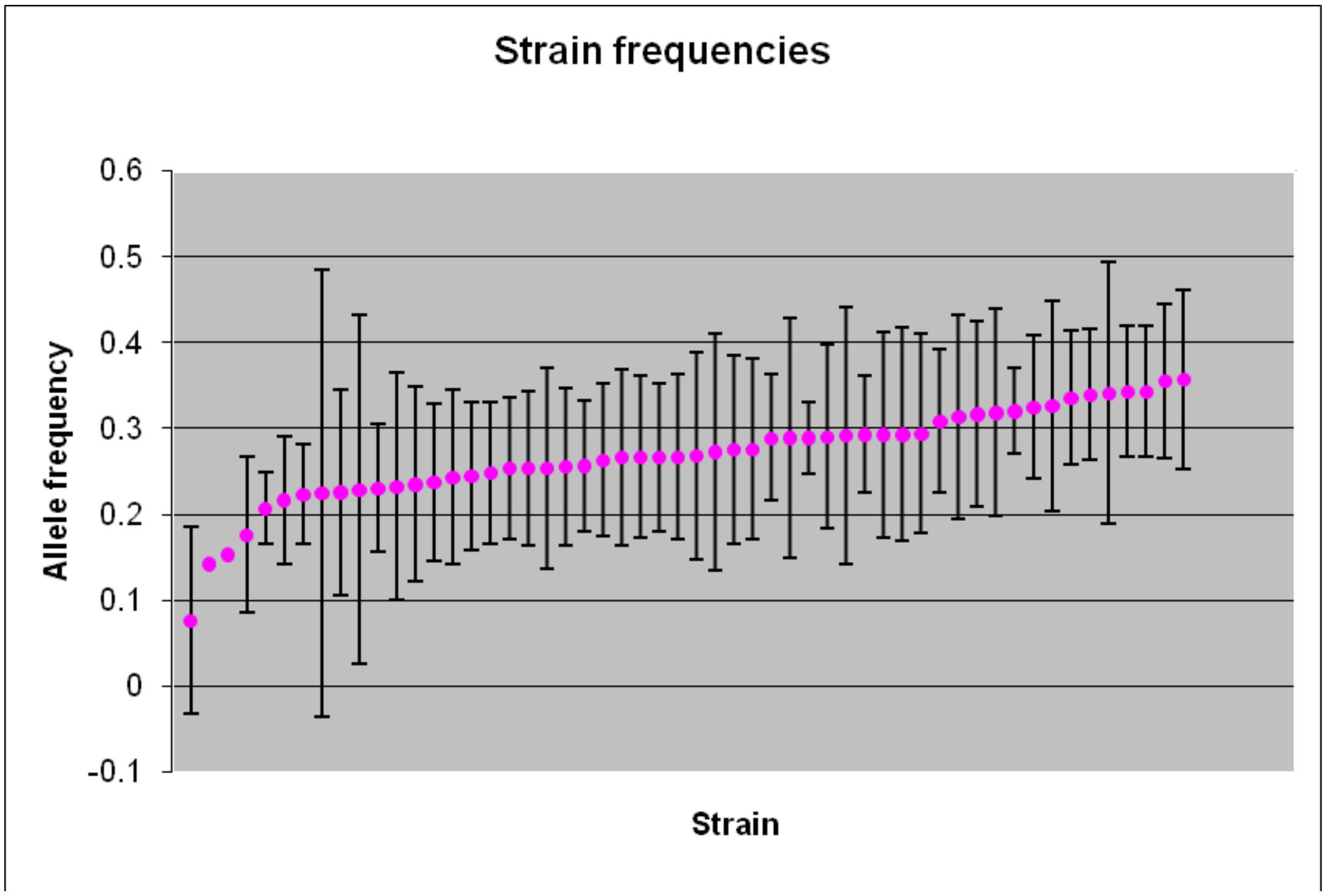

Figure S1: Frequencies of strains observed in the 5-way CG *Leptospirillum* group II population, calculated from the average allele frequency for each polymorphism occurring in a strain relative to the dominant population. Strains are sorted in ascending order of allele frequency. Error bars show ± 1 standard deviation. Each point represents one strain.
