# Supplementary material for: Population Genomic Analysis of Strain Variation in Leptospirillum Group II Bacteria Involved in Acid Mine Drainage Formation
Source: PLoS Biol. 2008 Jul 22;6(7):e177. doi: 10.1371/journal.pbio.0060177 (PMC2475542; doi:10.1371/journal.pbio.0060177)
Supplement: Supplementary file 3 [file pbio.0060177.sg002.pdf]

A

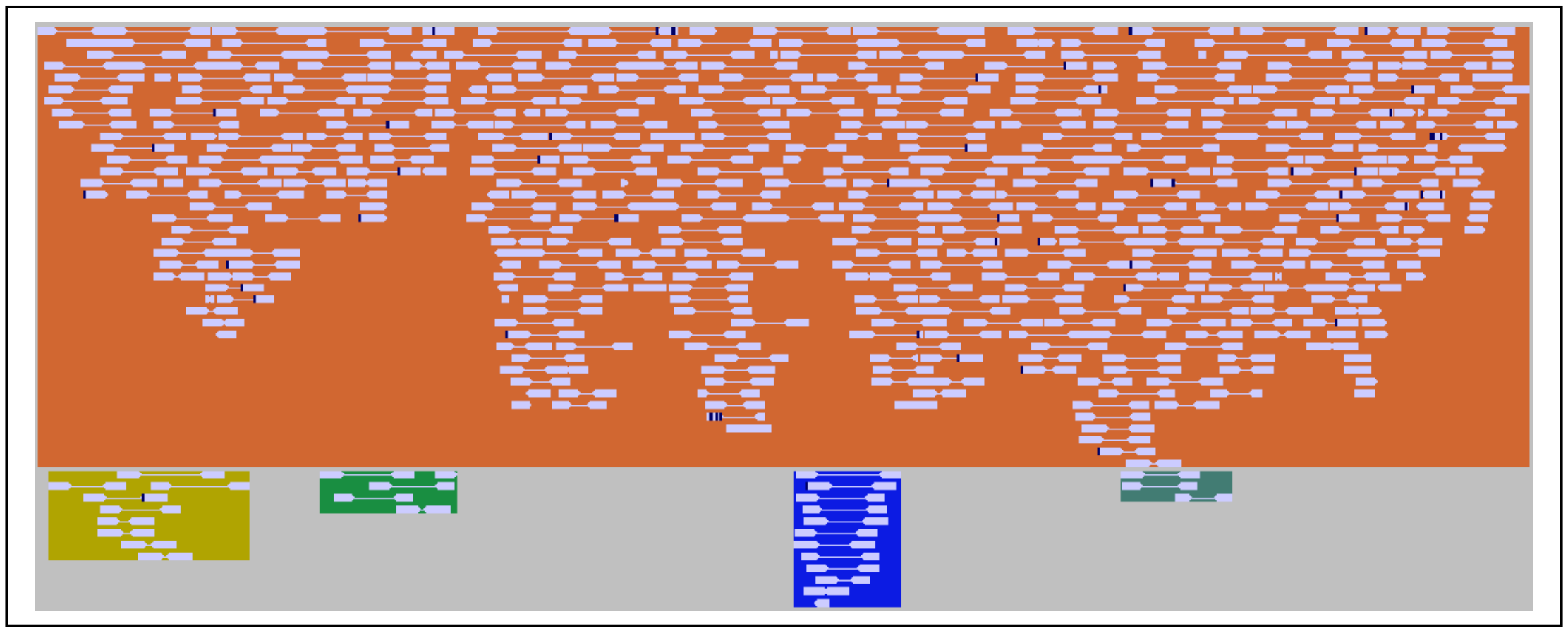

B

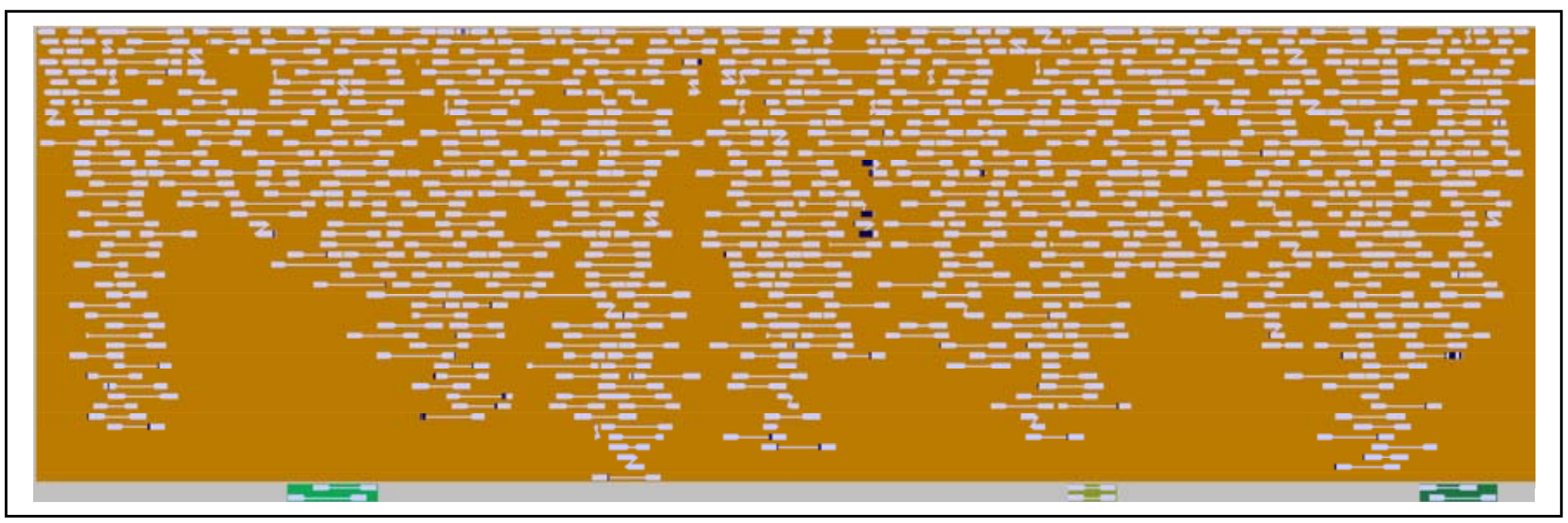

Figure S2: Diagram showing low strain variation in two contigs in different regions of the genome. Images were derived from the Strainer program [10]. White bars represent sequencing reads, linked by thin white lines to their mate paired reads. Dark shading on reads indicates the presence of SNPs. Colored boxes indicates reads that have been grouped together based on sequence similarity using SNPs that are replicated in more than one sequencing read. Numbers indicate the number of SNPs used to distinguished different variants (grouped reads surrounded by colored boxes) for cases where variant types are defined by more than one SNP.

A: Diagram showing strain variation over contig 11067 (54 kb). Variant types (colored boxes surrounding reads) were defined based on SNPs replicated in more than one read. This region contains 15 replicated substitutions (mustard colored variant: 10, green:2, blue: 2, and

olive: 1). **B:** Contig 10961: An area distant from the origin of replication. Only three strain groups were defined, and each contains only one replicated SNP.
