# Supplementary material for: Population Genomic Analysis of Strain Variation in Leptospirillum Group II Bacteria Involved in Acid Mine Drainage Formation
Source: PLoS Biol. 2008 Jul 22;6(7):e177. doi: 10.1371/journal.pbio.0060177 (PMC2475542; doi:10.1371/journal.pbio.0060177)
Supplement: Supplementary file 4 [file pbio.0060177.st001.pdf]

Table S1

Assembly of the genome of *Leptospirilum* group II from the 5-way CG location of the Richmond Mine (sample collected March 2002, as described by Tyson et al. 2004
The manually curated assembly (columns A to F) is compared with the draft assembly reported by Tyson et al. (column G). Red bars separate genome fragments for \
linkage has been established. Blue bars indicate the positions of major strain variant sequences (shown at end).

contig #	gene #	Locus_Tag	Product description	Length aa	Position on Phrap contig	Gene number (Tyson et al. 2004)
**Main genome path:**						
**9398**	**1**	**CGL2_09398001**	Putative cobyrinic acid a,c-diamide synthase	248	157..900	245_GENE_16
**9398**	**2**	**CGL2_09398002**	ParB-like partition protein	313	1047..1985	245_GENE_15
**10284**	**1**	**CGL2_10284001**	Transcriptional regulator, HxlR family	111	complement(680..1012)	245_GENE_14
**10284**	**2**	**CGL2_10284002**	NADPH:quinone reductase 1 (EC 1.6.5.5)	330	1184..2173	245_GENE_12
**10284**	**3**	**CGL2_10284003**	Hypothetical protein	69	2151..2357	245_GENE_11
**10284**	**4**	**CGL2_10284004**	Hypothetical protein	52	2483..2638	245_GENE_10
**10284**	**5**	**CGL2_10284005**	DNA methyltransferase	350	2661..3710	245_GENE_9
**10284**	**6**	**CGL2_10284006**	type II restriction endonuclease	241	3719..4441	245_GENE_8
**10284**	**7**	**CGL2_10284007**	isonitrile hydratase	268	4475..5278	245_GENE_7
**10284**	**8**	**CGL2_10284008**	Hypothetical protein	68	5385..5588	245_GENE_6
**10284**	**9**	**CGL2_10284009**	short-chain dehydrogenase	248	5576..6319	245_GENE_5
**10284**	**10**	**CGL2_10284010**	Putative phospholipid/glycerol acyltransferase	178	complement(6332..6865)	245_GENE_4
**10284**	**11**	**CGL2_10284011**	Probable conjugal transfer protein (TraB)	373	complement(7031..8149)	245_GENE_3
**7472**	**2**	**CGL2_07472002**	Probable conjugal transfer protein (TraF)	175	complement(1816..2340)	245_GENE_2
**7472**	**1**	**CGL2_07472001**	Putative diguanylate phosphodiesterase	256	complement(1040..1807)	245_GENE_1
**8228**	**1**	**CGL2_08228001**	Hypothetical protein	91	40..312	1147_GENE_3a
**8228**	**2**	**CGL2_08228002**	DNA topoisomerase III (EC 5.99.1.2)	684	320..2371	1147_GENE_3
**8228**	**3**	**CGL2_08228003**	Protein of unknown function	139	2334..2750	1147_GENE_2
**8228**	**4**	**CGL2_08228004**	Hypothetical protein	95	2807..3091	1147_GENE_1
**8228**	**5**	**CGL2_08228005**	Single-strand DNA-binding protein	133	3091..3489	
**8228**	**6**	**CGL2_08228006**	Hypothetical protein	16	3531..3578	
**6046**	**1**	**CGL2_06046001**	ssDNA binding protein	139	130..546	751_GENE_1
**6046**	**2**	**CGL2_06046002**	Hypothetical protein	78	609..842	751_GENE_2
**6046**	**3**	**CGL2_06046003**	DNA repair proteins (RadC)	146	829..1266	751_GENE_3
**9933**	**1**	**CGL2_09933001**	Conserved hypothetical protein	298	287..1180	751_GENE_4
**9933**	**2**	**CGL2_09933002**	Protein of unknown function	96	complement(1350..1637)	751_GENE_5
**9933**	**3**	**CGL2_09933003**	Conserved hypothetical protein	368	complement(1867..2970)	751_GENE_6
**9207**	**1**	**CGL2_09207001**	HD-GYP hydrolase domain protein/response regulato	206	116..733	259_GENE_12
**9207**	**3**	**CGL2_09207003**	Hypothetical protein	107	complement(1116..1436)	259_GENE_10

**9933**	**4**	**CGL2_09933004**	Protein of unknown function	154	complement(2970..3431)	259_GENE_9
**9933**	**5**	**CGL2_09933005**	Putative conjugal transfer protein (TrbI)	425	complement(3434..4708)	259_GENE_8
**9933**	**6**	**CGL2_09933006**	Putative conjugal transfer protein (TrbG)	312	complement(4708..5643)	259_GENE_7
**9933**	**7**	**CGL2_09933007**	Probable conjugal transfer protein (TrbF)	221	complement(5643..6305)	259_GENE_6
**9933**	**8**	**CGL2_09933008**	Probable conjugal transfer protein (TrbL)	454	complement(6308..7669)	259_GENE_5
**8831**	**1**	**CGL2_08831001**	Probable conjugal transfer (TrbJ)	288	complement(349..1212)	259_GENE_4
**8831**	**2**	**CGL2_08831002**	Probable conjugal transfer protein (TrbE)	812	complement(1230..3665)	259_GENE_2
**8831**	**3**	**CGL2_08831003**	Putative conjugal transfer protein (TrbD)	104	complement(3668..3979)	259_GENE_1
**8831**	**4**	**CGL2_08831004**	Hypothetical protein	102	complement(3983..4288)	
**8831**	**5**	**CGL2_08831005**	Putative conjugal transfer protein (TrbB)	168	complement(4288..4791)	440_GENE_3
**11226**	**1**	**CGL2_11226001**	Putative plasmid transfer factor (TraG)	65	complement(240..434)	440_GENE_5
**11226**	**2**	**CGL2_11226002**	Hypothetical protein	166	595..1092	582_GENE_1
**11226**	**3**	**CGL2_11226003**	Hypothetical protein	64	1103..1294	582_GENE_2
**11226**	**4**	**CGL2_11226004**	Hypothetical protein	141	1294..1716	582_GENE_3
**11226**	**5**	**CGL2_11226005**	Putative twitching motility protein (PilT)	346	1716..2753	582_GENE_4
**11226**	**6**	**CGL2_11226006**	Hypothetical protein	438	2753..4066	582_GENE_6
**11226**	**7**	**CGL2_11226007**	Protein of unknown function	196	4069..4656	582_GENE_7
**11226**	**8**	**CGL2_11226008**	Hypothetical protein	363	4656..5744	582_GENE_8
**11226**	**9**	**CGL2_11226009**	Putative type II secretion system protein D	615	5822..7666	
**11226**	**10**	**CGL2_11226010**	Putative type II secretion system protein E	574	7673..9394	
**11226**	**11**	**CGL2_11226011**	Conserved protein of unknown function	202	9397..10002	
**11226**	**12**	**CGL2_11226012**	Hypothetical protein	160	complement(10072..10551)	
**11226**	**13**	**CGL2_11226013**	Conserved hypothetical protein	101	complement(10734..11036)	784_GENE_6
**11226**	**14**	**CGL2_11226014**	Conserved hypothetical protein	136	complement(11106..11513)	784_GENE_5
**11226**	**15**	**CGL2_11226015**	Putative type II secretion system protein F	402	complement(11654..12859)	1145_GENE_1
**11226**	**16**	**CGL2_11226016**	Hypothetical protein	85	complement(12859..13113)	
**11226**	**17**	**CGL2_11226017**	Putative phage integrase	346	complement(13238..14275)	784_GENE_2
11226tRNA		**CGL2_11226R001**	TRNA Arg CCG		complement(14411..14488)	
**11226**	**18**	**CGL2_11226018**	Putative Cl-channel, voltage gated	570	complement(14549..16258)	283_GENE_8
**11226**	**19**	**CGL2_11226019**	Conserved hypothetical protein	144	complement(16258..16689)	283_GENE_7
**11226**	**20**	**CGL2_11226020**	Hypothetical protein	78	complement(16881..17114)	283_GENE_6
**11226**	**21**	**CGL2_11226021**	Protoporphyrinogen oxidase (EC 1.3.3.4)	462	17322..18707	283_GENE_5
**11226**	**22**	**CGL2_11226022**	Putative metallophosphoesterase	227	complement(18727..19407)	283_GENE_4
**11226**	**23**	**CGL2_11226023**	Zinc-containing alcohol dehydrogenase superfamily (	333	complement(19436..20434)	283_GENE_3
**11226**	**24**	**CGL2_11226024**	Putative radical SAM family protein	172	complement(20542..21057)	283_GENE_2
**11067**	**45**	**CGL2_11067045**	Protein of unknown function	108	complement(53116..53439)	53_GENE_28
**11067**	**44**	**CGL2_11067044**	Probable signal transduction protein	277	complement(52073..52903)	53_GENE_27
**11067**	**43**	**CGL2_11067043**	Conserved protein of unknown function	485	complement(50243..51697)	53_GENE_26
**11067**	**42**	**CGL2_11067042**	Hypothetical protein	496	complement(48607..50094)	53_GENE_24
**11067**	**41**	**CGL2_11067041**	Conserved protein of unknown function	185	complement(48034..48588)	53_GENE_23
**11067**	**40**	**CGL2_11067040**	Glucokinase (EC 2.7.1.2)	344	complement(46445..47476)	53_GENE_22

**11067**	**39**	**CGL2_11067039**	6-phosphogluconolactonase (EC 3.1.1.31)	276	complement(45477..46304)	53_GENE_21
**11067**	**38**	**CGL2_11067038**	Transketolase (EC 2.2.1.1)	677	complement(43407..45437)	53_GENE_20
**11067**	**37**	**CGL2_11067037**	Transaldolase	977	complement(40415..43345)	53_GENE_19
**11067**	**36**	**CGL2_11067036**	Glucose-6-phosphate 1-dehydrogenase (EC 1.1.1.49	510	complement(38831..40360)	53_GENE_18
**11067**	**35**	**CGL2_11067035**	Protein of unknown function	582	complement(37072..38817)	53_GENE_17
**11067**	**34**	**CGL2_11067034**	Putative monooxygenase	414	complement(35831..37072)	53_GENE_16
**11067**	**33**	**CGL2_11067033**	Probable trigger factor	467	complement(34231..35631)	53_GENE_15
**11067**	**32**	**CGL2_11067032**	Peptidase S14, ClpP (EC 3.4.21.92)	203	complement(33595..34203)	53_GENE_14
**11067**	**31**	**CGL2_11067031**	ClpX, ATPase regulatory subunit	426	complement(32287..33564)	53_GENE_13
**11067**	**30**	**CGL2_11067030**	Protein of unknown function	217	31634..32284	53_GENE_12
**11067**	**29**	**CGL2_11067029**	Protein of unknown function	442	complement(30241..31566)	53_GENE_11
**11067**	**28**	**CGL2_11067028**	Putative CDP-alcohol phosphatidyltransferase	183	complement(29690..30238)	53_GENE_10
**11067**	**27**	**CGL2_11067027**	Conserved protein of unknown function	462	complement(28288..29673)	53_GENE_9
**11067**	**26**	**CGL2_11067026**	4-diphosphocytidyl-2-C-methyl-D-erythritol kinase (E	293	complement(27410..28288)	53_GENE_8
11067tRNA		**CGL2_11067R006**	TRNA Gln TTG		complement(27326..27400)	
**11067**	**25**	**CGL2_11067025**	Ribose-phosphate pyrophosphokinase (EC 2.7.6.1)	314	complement(26318..27259)	53_GENE_7
**11067**	**24**	**CGL2_11067024**	Ribosomal protein L25	207	complement(25636..26256)	53_GENE_6
**11067**	**23**	**CGL2_11067023**	Peptidyl-tRNA hydrolase (EC 3.1.1.29)	187	complement(25054..25614)	53_GENE_5
**11067**	**22**	**CGL2_11067022**	GTP binding protein	364	complement(23895..24986)	53_GENE_4
**11067**	**21**	**CGL2_11067021**	Ribosomal protein S6	122	complement(23499..23864)	271_GENE_14
**11067**	**20**	**CGL2_11067020**	Single-stranded DNA-binding protein	140	complement(23080..23499)	271_GENE_13
**11067**	**19**	**CGL2_11067019**	Ribosomal protein S18	76	complement(22781..23008)	271_GENE_12
**11067**	**18**	**CGL2_11067018**	Hypothetical protein	289	complement(21810..22676)	271_GENE_11
**11067**	**17**	**CGL2_11067017**	Rare lipoprotein A family protein	263	complement(21080..21868)	271_GENE_10
**11067**	**16**	**CGL2_11067016**	Protein of unknown function	74	complement(20800..21021)	271_GENE_9
**11067**	**15**	**CGL2_11067015**	Conserved hypothetical protein	56	complement(20626..20793)	271_GENE_8
**11067**	**14**	**CGL2_11067014**	Protein of unknown function	151	20162..20614	271_GENE_7
**11067**	**13**	**CGL2_11067013**	RNA modification enzyme, MiaB family	483	complement(18599..20047)	271_GENE_6
**11067**	**12**	**CGL2_11067012**	Hypothetical protein	342	17538..18563	271_GENE_5
11067tRNA		**CGL2_11067R005**	TRNA Leu TAG		complement(17410..17495)	
**11067**	**11**	**CGL2_11067011**	Putative flavoprotein reductase	379	complement(16112..17248)	271_GENE_3
**11067**	**10**	**CGL2_11067010**	Protein of unknown function	258	complement(15336..16109)	271_GENE_2
11067tRNA		**CGL2_11067R004**	TRNA Tyr GTA		complement(15070..15155)	
11067tRNA		**CGL2_11067R003**	TRNA Gly TCC		complement(14959..15033)	
11067tRNA		**CGL2_11067R002**	TRNA Thr GGT		complement(14860..14935)	
**11067**	**9**	**CGL2_11067009**	Translation elongation factor Tu (EC 2.7.7.4)	399	complement(13549..14745)	137_GENE_1
**11067**	**8**	**CGL2_11067008**	Ribosomal protein L33	49	complement(13372..13518)	137_GENE_1a
11067tRNA		**CGL2_11067R001**	TRNA Trp CCA		complement(13285..13361)	
**11067**	**7**	**CGL2_11067007**	Preprotein translocase (SecE)	69	complement(13064..13270)	
**11067**	**6**	**CGL2_11067006**	Transcription antitermination protein (NusG)	184	complement(12489..13040)	137_GENE_2
**11067**	**5**	**CGL2_11067005**	Ribosomal protein L11	141	complement(12027..12449)	137_GENE_3

**11067**	**4**	**CGL2_11067004**	Ribosomal protein L1	232	complement(11286..11981)	137_GENE_4
**11067**	**3**	**CGL2_11067003**	Ribosomal protein L10	174	complement(10762..11283)	137_GENE_5
**11067**	**2**	**CGL2_11067002**	Ribosomal protein L7/L12	128	complement(10271..10654)	137_GENE_6
**11067**	**1**	**CGL2_11067001**	DNA-directed RNA polymerase, beta subunit (EC 2.7	1718	complement(4756..9909)	137_GENE_7
**11067**	**1a**	**CGL2_11067001a**	DNA-directed RNA polymerase, beta' subunit (EC 2.7	1574	complement(30..4751)	137_GENE_8
**11212**	**1**	**CGL2_11212001**	Ribosomal protein S12	65	133..327	713_GENE_4
**11212**	**2**	**CGL2_11212002**	Ribosomal protein S7	157	368..838	713_GENE_3
**11212**	**3**	**CGL2_11212003**	Translation elongation factor G	692	854..2929	713_GENE_2
**11212**	**4**	**CGL2_11212004**	Translation elongation factor Tu (EC 2.7.7.4)	399	2952..4148	713_GENE_1
**11212**	**5**	**CGL2_11212005**	Ribosomal protein S10	101	4185..4487	
**11212**	**6**	**CGL2_11212006**	Ribosomal protein L3	207	4502..5122	349_GENE_14
**11212**	**7**	**CGL2_11212007**	Ribosomal protein L4	218	5147..5800	349_GENE_13
**11212**	**8**	**CGL2_11212008**	Ribosomal protein L23	95	5790..6074	
**11212**	**9**	**CGL2_11212009**	Ribosomal protein L2	273	6080..6898	349_GENE_12
**11212**	**10**	**CGL2_11212010**	Ribosomal protein S19	94	6917..7198	349_GENE_11
**11212**	**11**	**CGL2_11212011**	Ribosomal protein L22	110	7223..7552	349_GENE_10
**11212**	**12**	**CGL2_11212012**	Ribosomal protein S3	226	7573..8250	349_GENE_9
**11212**	**13**	**CGL2_11212013**	Ribosomal protein L16/L10E	127	8287..8667	349_GENE_8
**11212**	**14**	**CGL2_11212014**	Ribosomal protein L29	66	8667..8864	349_GENE_7a
**11212**	**15**	**CGL2_11212015**	Ribosomal protein S17	95	8860..9144	349_GENE_7
**11212**	**16**	**CGL2_11212016**	Ribosomal protein L14	122	9182..9547	349_GENE_6
**11212**	**17**	**CGL2_11212017**	Ribosomal protein L24	117	9564..9914	349_GENE_5a
**11212**	**18**	**CGL2_11212018**	Ribosomal protein L5	226	9946..10623	349_GENE_5
**11212**	**19**	**CGL2_11212019**	Ribosomal protein S14	61	10646..10828	349_GENE_4a
**11212**	**20**	**CGL2_11212020**	Ribosomal protein S8	132	10853..11248	349_GENE_4
**11212**	**21**	**CGL2_11212021**	Ribosomal protein L6P/L9E	179	11280..11816	349_GENE_3
**11212**	**22**	**CGL2_11212022**	Ribosomal protein L18	117	11855..12205	349_GENE_2
**11212**	**23**	**CGL2_11212023**	Ribosomal protein S5	161	12234..12716	349_GENE_1
**11212**	**24**	**CGL2_11212024**	Ribosomal protein L30	63	12751..12939	88_GENE_20
**11212**	**25**	**CGL2_11212025**	Ribosomal protein L15	149	12975..13421	88_GENE_19
**11212**	**26**	**CGL2_11212026**	Preprotein translocase subunit (SecY)	437	13445..14755	88_GENE_18
**11212**	**27**	**CGL2_11212027**	Adenylate kinase (EC 2.7.4.3)	219	14761..15417	88_GENE_17
**11212**	**28**	**CGL2_11212028**	Methionine aminopeptidase (EC 3.4.11.18)	252	15423..16178	88_GENE_16
**11212**	**29**	**CGL2_11212029**	Translation initiation factor 1 (IF-1)	72	16191..16406	88_GENE_16a
**11212**	**30**	**CGL2_11212030**	Ribosomal protein L36	37	16420..16530	
**11212**	**31**	**CGL2_11212031**	Ribosomal protein S13	124	16545..16916	
**11212**	**32**	**CGL2_11212032**	Ribosomal protein S11	128	16970..17353	88_GENE_15a
**11212**	**33**	**CGL2_11212033**	Ribosomal protein S4 and related proteins	209	17410..18036	88_GENE_15
**11212**	**34**	**CGL2_11212034**	DNA-directed RNA polymerase, alpha subunit (EC 2.7	324	18083..19054	88_GENE_14
**11212**	**35**	**CGL2_11212035**	Ribosomal protein L17	154	19093..19554	88_GENE_13
**11212**	**36**	**CGL2_11212036**	Protein of unknown function	143	19579..20007	88_GENE_12

**11212**	**37**	**CGL2_11212037**	DTDP-4-dehydrorhamnose reductase (EC 1.1.1.133)	288	20079..20942	88_GENE_11
**11212**	**38**	**CGL2_11212038**	Trehalose-6-phosphate phosphatase	285	complement(20962..21816)	88_GENE_10
**11212**	**39**	**CGL2_11212039**	Alpha,alpha-trehalose-phosphate synthase (UDP-forr	500	complement(21785..23284)	88_GENE_8
**11212**	**40**	**CGL2_11212040**	Putative glycosyl transferase, group 1	361	complement(23326..24408)	88_GENE_7
**11212**	**41**	**CGL2_11212041**	Putative major facilitator superfamily transporter	428	complement(24414..25697)	88_GENE_6
**11212**	**42**	**CGL2_11212042**	Probable macrolide-efflux protein	429	complement(25851..27137)	88_GENE_5
**11212**	**43**	**CGL2_11212043**	ADP-L-glycero-D-mannoheptose-6-epimerase (EC 5.	303	27352..28260	88_GENE_4
**11212**	**44**	**CGL2_11212044**	Protein of unknown function	610	28241..30070	88_GENE_3
**11212**	**45**	**CGL2_11212045**	Amidophosphoribosyltransferase (EC 2.4.2.14)	504	complement(30090..31601)	88_GENE_2
**11212**	**46**	**CGL2_11212046**	Phosphoribosylformylglycinamidine synthase II (EC 6	737	complement(31607..33817)	88_GENE_1
**11212**	**47**	**CGL2_11212047**	Phosphoribosylformylglycinamidine synthase I	255	complement(33924..34688)	30_GENE_4
**11212**	**48**	**CGL2_11212048**	Conserved hypothetical protein	257	34948..35718	30_GENE_5
**11212**	**49**	**CGL2_11212049**	Protein of unknown function	238	35917..36630	30_GENE_6
**11212**	**50**	**CGL2_11212050**	Putative transcriptional regulator, TetR family	237	36679..37389	30_GENE_7
**11212**	**51**	**CGL2_11212051**	Putative outer membrane efflux protein	490	37389..38858	30_GENE_8
**11212**	**52**	**CGL2_11212052**	Secretion protein (HlyD)	376	38854..39981	30_GENE_9
**11212**	**53**	**CGL2_11212053**	Putative cation/multidrug efflux pump split	1070	40003..43212	30_GENE_10
**11212**	**54**	**CGL2_11212054**	Putative transcriptional regulator, LysR family	310	complement(43219..44148)	30_GENE_11
**11212**	**55**	**CGL2_11212055**	Putative outer membrane efflux protein	474	44343..45764	30_GENE_12
**11212**	**56**	**CGL2_11212056**	Protein of unknown function	243	45764..46492	30_GENE_13
**11212**	**57**	**CGL2_11212057**	Protein of unknown function	207	46534..47154	30_GENE_14
**11212**	**58**	**CGL2_11212058**	ABC transporter, ATPase subunit (EC 3.6.3.29)	256	47150..47917	30_GENE_15
**11212**	**59**	**CGL2_11212059**	Putative ABC-2 transporter	285	47913..48767	30_GENE_16
**11212**	**60**	**CGL2_11212060**	Putative oxidoreductase FAD/NAD(P)-binding	257	48821..49591	30_GENE_17
**11212**	**61**	**CGL2_11212061**	Protein of unknown function	346	49656..50693	30_GENE_18
**11212**	**62**	**CGL2_11212062**	Putative 3-hydroxybutyryl-coA dehydrogenase	378	50707..51840	30_GENE_19
**11212**	**63**	**CGL2_11212063**	Protein of unknown function	160	51915..52394	30_GENE_20
**11212**	**63**	**CGL2_11212063**	Protein of unknown function	160	51915..52394	30_GENE_20
**11212**	**65**	**CGL2_11212065**	Protein of unknown function	231	52537..53229	30_GENE_21
**11212**	**66**	**CGL2_11212066**	Putative TPR-domain containing protein	731	53229..55421	30_GENE_22
**11212**	**67**	**CGL2_11212067**	Putative DegT/DnrJ/EryC1/StrS aminotransferase	391	55443..56615	30_GENE_23
**11212**	**68**	**CGL2_11212068**	Putative OsmC-like protein (osmotic induced)	173	56615..57133	30_GENE_24
**11212**	**69**	**CGL2_11212069**	Probable dethiobiotin synthase	237	57085..57795	30_GENE_25
**11212**	**70**	**CGL2_11212070**	Protein of unknown function	110	complement(57853..58182)	30_GENE_26
**11212**	**71**	**CGL2_11212071**	Protein of unknown function	68	complement(58263..58466)	30_GENE_27
**11212**	**72**	**CGL2_11212072**	Protein of unknown function	284	complement(58472..59323)	30_GENE_28
**11212**	**73**	**CGL2_11212073**	Protein of unknown function	350	complement(59348..60397)	30_GENE_29
**11212**	**74**	**CGL2_11212074**	Protein of unknown function	107	complement(60412..60732)	30_GENE_30
**11212**	**75**	**CGL2_11212075**	Multi-sensor signal transduction histidine kinase	596	61548..63335	30_GENE_31
**11212**	**76**	**CGL2_11212076**	Putative response regulator receiver protein	148	63416..63859	30_GENE_32
**11212**	**77**	**CGL2_11212077**	Response regulator receiver modulated diguanylate c	874	63923..66544	30_GENE_33

**11212**	**78**	**CGL2_11212078**	Thiamine-phosphate pyrophosphorylase ThiE(EC 2.5.	212	complement(66551..67186)	30_GENE_34
**11212**	**79**	**CGL2_11212079**	Acetyl-CoA carboxylase, biotin carboxylase (EC 6.3.4	456	complement(67235..68602)	30_GENE_35
**11212**	**80**	**CGL2_11212080**	Acetyl-CoA biotin carboxyl carrier protein	157	complement(68611..69081)	30_GENE_36
**11212**	**81**	**CGL2_11212081**	Translation elongation factor P	191	complement(69131..69703)	30_GENE_37
**11212**	**82**	**CGL2_11212082**	3-dehydroquinate dehydratase, type II (EC 4.2.1.10	149	complement(69771..70217)	30_GENE_38
**11212**	**83**	**CGL2_11212083**	Putative TPR-domain containing protein	464	complement(70217..71608)	30_GENE_39
**11212**	**84**	**CGL2_11212084**	DNA/pantothenate metabolism flavoprotein (EC 4.1.	415	complement(71770..73014)	30_GENE_40
**11212**	**85**	**CGL2_11212085**	Putative DNA-directed RNA polymerase, omega subu	134	complement(73025..73426)	30_GENE_41
**11212**	**86**	**CGL2_11212086**	Guanylate kinase (EC 2.7.4.8)	243	complement(73426..74154)	30_GENE_42
**11212**	**87**	**CGL2_11212087**	Conserved protein of unknown function	94	complement(74222..74503)	30_GENE_43
**11212**	**88**	**CGL2_11212088**	Conserved protein of unknown function	293	complement(74496..75374)	30_GENE_44
**11212**	**89**	**CGL2_11212089**	Probable amylo-alpha-1,6-glucosidase	725	75575..77749	30_GENE_45
**11212**	**90**	**CGL2_11212090**	Putative penicillin-binding protein, transpeptidase	468	77964..79367	30_GENE_46
**11212**	**91**	**CGL2_11212091**	Putative signal-transduction protein with CBS domai	136	79504..79911	317_GENE_7
**11212**	**92**	**CGL2_11212092**	8-amino-7-oxononanoate synthase (EC 2.3.1.47)	380	complement(80007..81146)	317_GENE_6
**11212**	**93**	**CGL2_11212093**	6-carboxyhexanoate--CoA ligase (EC 6.2.1.14)	300	complement(81127..82026)	317_GENE_5
**11212**	**94**	**CGL2_11212094**	Biotin synthetase (EC 2.8.1.6)	346	complement(82026..83063)	317_GENE_4
**11212**	**95**	**CGL2_11212095**	Diguanylate cyclase	412	83322..84557	317_GENE_3
**11212**	**96**	**CGL2_11212096**	transposase	124	85643..86014	94_GENE_21
**11212**	**97**	**CGL2_11212097**	Glycyl-tRNA synthetase, alpha subunit (EC 6.1.1.14)	300	86421..87320	94_GENE_20
**11212**	**98**	**CGL2_11212098**	Glycyl-tRNA synthetase, beta subunit (EC 6.1.1.14)	739	87320..89536	94_GENE_19
**11212**	**99**	**CGL2_11212099**	Riboflavin biosynthesis protein RibD (EC 3.5.4.26) (E	388	89539..90702	94_GENE_18
**11212**	**100**	**CGL2_11212100**	Riboflavin synthase, alpha subunit	210	90709..91338	94_GENE_17
**11212**	**101**	**CGL2_11212101**	Hypothetical protein	67	91354..91554	94_GENE_16
**11212**	**102**	**CGL2_11212102**	Cobalt-precorrin-2 C(20)-methyltransferase (EC 2.1.	251	91554..92306	94_GENE_15
**11212**	**103**	**CGL2_11212103**	Hypothetical protein	179	92306..92842	94_GENE_14
**11212**	**104**	**CGL2_11212104**	Precorrin-4 C11-methyltransferase (EC 2.1.1.133)	279	92829..93665	94_GENE_13
**11212**	**105**	**CGL2_11212105**	Protein of unknown function (transcrip reg?)	179	complement(93823..94359)	94_GENE_12
**11212**	**106**	**CGL2_11212106**	Protein of unknown function	221	complement(94513..95175)	94_GENE_11
**11212**	**107**	**CGL2_11212107**	Putative radical SAM family protein	476	complement(95203..96630)	94_GENE_10
**11212**	**108**	**CGL2_11212108**	Cation efflux family protein	315	96844..97788	94_GENE_9
**11212**	**109**	**CGL2_11212109**	Conserved hypothetical protein	112	complement(97802..98137)	94_GENE_8
**11212**	**110**	**CGL2_11212110**	Putative DNA recombination protein, RmuC family	477	complement(98137..99567)	94_GENE_7
**11212**	**111**	**CGL2_11212111**	Dihydroxy-acid dehydratase (EC 4.2.1.9)	557	complement(99607..101277)	94_GENE_6
**11212**	**112**	**CGL2_11212112**	Protein of unknown function	183	complement(101476..102024)	94_GENE_5
**11212**	**113**	**CGL2_11212113**	Hypothetical protein	59	complement(102073..102249)	94_GENE_4
**11212**	**114**	**CGL2_11212114**	Cobyric acid synthase (CobQ)	500	102394..103893	94_GENE_3
**11212**	**115**	**CGL2_11212115**	Cobalamin biosynthesis protein (CbiB)	338	103893..104906	94_GENE_2
**11212**	**116**	**CGL2_11212116**	Putative L-threonine-O-3-phosphate decarboxylase	369	104913..106019	94_GENE_1
**11212**	**117**	**CGL2_11212117**	Fumarate hydratase class II (EC 4.2.1.2)	471	106493..107905	57_GENE_26

11212tRNA		**CGL2_11212R001**	TRNA Pro CGG		108074..108150	
**11212**	**118**	**CGL2_11212118**	Putative acylphosphatase	99	108264..108560	57_GENE_24
**11212**	**119**	**CGL2_11212119**	RNA polymerase, RpoD-like sigma subunit	499	108623..110119	57_GENE_23
**11212**	**120**	**CGL2_11212120**	Conserved protein of unknown function	110	110125..110454	57_GENE_22
**11212**	**121**	**CGL2_11212121**	Glutamate synthase [NADPH] large subunit (EC 1.4.1	1525	complement(110573..115147)	57_GENE_21
**11212**	**122**	**CGL2_11212122**	Putative metal dependent phosphohydrolase	332	115413..116408	57_GENE_20
**11212**	**123**	**CGL2_11212123**	D-lactate dehydrogenase (Cytochrome) (EC 1.1.2.4)	945	116408..119242	57_GENE_19
**11212**	**124**	**CGL2_11212124**	Putative methyl-accepting chemotaxis sensory transd	372	complement(119251..120366)	57_GENE_18
**11212**	**125**	**CGL2_11212125**	Probable acyl-[acyl-carrier protein] desaturase	286	complement(120700..121557)	57_GENE_17
**11212**	**126**	**CGL2_11212126**	Exodeoxyribonuclease III (EC 3.1.11.2)	265	complement(121793..122587)	57_GENE_16
**11212**	**127**	**CGL2_11212127**	Probable cation transport protein	181	complement(122580..123122)	57_GENE_15
**11212**	**128**	**CGL2_11212128**	3-methyl-2-oxobutanoate hydroxymethyltransferase	287	complement(123127..123987)	57_GENE_14
**11212**	**129**	**CGL2_11212129**	Putative methylenetetrahydrofolate reductase	289	complement(123993..124859)	57_GENE_13
**11212**	**130**	**CGL2_11212130**	Methenyltetrahydrofolate cyclohydrolase (EC 3.5.4.9	294	complement(124859..125740)	57_GENE_12
**11212**	**131**	**CGL2_11212131**	GTP cyclohydrolase I (EC 3.5.4.16)	191	complement(125824..126396)	57_GENE_11
**11212**	**132**	**CGL2_11212132**	Putative 5-formyltetrahydrofolate cyclo-ligase	206	complement(126377..126994)	57_GENE_10
**11212**	**133**	**CGL2_11212133**	Probable citrate transporter	633	127016..128914	57_GENE_9
**11212**	**134**	**CGL2_11212134**	Putative acyltransferase	232	complement(128922..129617)	57_GENE_8
**11212**	**135**	**CGL2_11212135**	Cytidylate kinase (EC 2.7.4.14)	221	complement(129593..130255)	57_GENE_7
**11212**	**136**	**CGL2_11212136**	3-phosphoshikimate 1-carboxyvinyltransferase (EC 2	454	complement(130252..131613)	57_GENE_6
**11212**	**138**	**CGL2_11212138**	Prephenate dehydrogenase (EC 1.3.1.12)	302	complement(131600..132505)	57_GENE_5
**11212**	**139**	**CGL2_11212139**	Phospho-2-dehydro-3-deoxyheptonate aldolase, sub	340	complement(132505..133524)	57_GENE_4
**11212**	**140**	**CGL2_11212140**	Prephenate dehydratase (EC 4.2.1.51)	365	complement(133548..134642)	57_GENE_3
**11212**	**141**	**CGL2_11212141**	Conserved protein of unknown function	252	complement(134731..135486)	57_GENE_2
**11212**	**142**	**CGL2_11212142**	Probable cytochrome C oxidase mono-heme subunit	176	135909..136436	120_GENE_14
**11212**	**143**	**CGL2_11212143**	Conserved hypothetical protein	429	136596..137882	120_GENE_13
**11212**	**144**	**CGL2_11212144**	Glycine oxidase (ThiO)	375	complement(137898..139022)	120_GENE_12
**11212**	**145**	**CGL2_11212145**	UDP-glucose 6-dehydrogenase (EC 1.1.1.22)	443	139252..140580	120_GENE_11
**11212**	**146**	**CGL2_11212146**	Sugar fermentation stimulation protein	239	140562..141278	120_GENE_10
**11212**	**147**	**CGL2_11212147**	Putative NAD-dependent epimerase/dehydratase fam	308	141307..142230	120_GENE_9
**11212**	**148**	**CGL2_11212148**	Conserved hypothetical protein	349	142223..143269	120_GENE_8
**11212**	**149**	**CGL2_11212149**	Nucleoside-diphosphate kinase (EC 2.7.4.6)	138	143276..143689	120_GENE_7
**11212**	**150**	**CGL2_11212150**	Protein of unknown function	397	143746..144936	120_GENE_6
**11212**	**151**	**CGL2_11212151**	Tyrosyl-tRNA synthetase (EC 6.1.1.1)	403	145018..146226	120_GENE_5
**11212**	**152**	**CGL2_11212152**	Secretion protein HlyD	374	146440..147561	120_GENE_4
**11212**	**153**	**CGL2_11212153**	Heavy metal efflux pump, CzcA family	1027	147561..150641	120_GENE_3
**11212**	**154**	**CGL2_11212154**	Putative glutamine amidotransferase class-I	235	150735..151439	120_GENE_2
**11212**	**155**	**CGL2_11212155**	Transposase	446	151619..152956	120_GENE_1
11233rRNA		**CGL2_11233R001**	16S rRNA		219..1736	
11233tRNA		**CGL2_11233R001**	TRNA Ile GAT		1862..1938	
11233tRNA		**CGL2_11233R002**	TRNA Ala TGC		1944..2019	

11233rRNA		**CGL2_11233R002**	23S rRNA		2347..4992	
11233rRNA		**CGL2_11233R003**	5S rRNA		5220..5283	
**11233**	**1**	**CGL2_11233001**	Hypothetical protein	49	5616..5762	
**11233**	**3**	**CGL2_11233003**	NADH dehydrogenase, subunit F (EC 1.6.99.5)	1048	5797..8940	67_GENE_27
**11233**	**4**	**CGL2_11233004**	Putative endonuclease III	210	complement(8905..9534)	67_GENE_26
**11233**	**5**	**CGL2_11233005**	Putative transcriptional regulators, TraR/DksA family	136	9653..10060	67_GENE_25
**11233**	**6**	**CGL2_11233006**	Prolipoprotein diacylglyceryl transferase	272	10091..10906	67_GENE_24
**11233**	**7**	**CGL2_11233007**	Putative methyl-accepting chemotaxis sensory transd	484	10919..12370	67_GENE_23
**11233**	**8**	**CGL2_11233008**	Transcriptional regulator, BadM/Rrf2 family	141	12409..12831	67_GENE_22
**11233**	**9**	**CGL2_11233009**	Hypothetical protein	109	complement(12830..13156)	67_GENE_21
**11233**	**10**	**CGL2_11233010**	Conserved hypothetical protein	90	13304..13573	67_GENE_20
**11233**	**11**	**CGL2_11233011**	Conserved protein of unknown function	380	complement(13576..14715)	67_GENE_19
**11233**	**12**	**CGL2_11233012**	Putative permease	852	complement(14711..17266)	67_GENE_18
**11233**	**13**	**CGL2_11233013**	ABC transporter, ATP-binding protein (3.6.3.25)	241	complement(17273..17995)	67_GENE_17
**11233**	**14**	**CGL2_11233014**	3-hydroxyisobutyrate dehydrogenase (EC 1.1.1.31)	292	complement(17995..18870)	67_GENE_16
**11233**	**15**	**CGL2_11233015**	Hypothetical protein	87	19172..19432	67_GENE_14
**11233**	**16**	**CGL2_11233016**	Conserved hypothetical protein	97	19435..19725	67_GENE_13
**11233**	**17**	**CGL2_11233017**	Conserved hypothetical protein	834	19721..22222	67_GENE_12
**11233**	**18**	**CGL2_11233018**	Hypothetical protein	204	22222..22833	67_GENE_11
**11233**	**19**	**CGL2_11233019**	Hypothetical protein	410	22845..24074	67_GENE_10
**11233**	**20**	**CGL2_11233020**	Hypothetical protein	229	24074..24760	67_GENE_9
**11233**	**21**	**CGL2_11233021**	DNA-dependent RNA polymerase	385	24760..25914	67_GENE_8
**11233**	**22**	**CGL2_11233022**	Hypothetical protein	316	25919..26866	67_GENE_7
**11233**	**23**	**CGL2_11233023**	Putative MoxR-like ATPases	282	26866..27711	67_GENE_6
**11233**	**24**	**CGL2_11233024**	Hypothetical protein	151	complement(27892..28344)	67_GENE_5
**11233**	**25**	**CGL2_11233025**	Putative oxygen-independent coproporphyrinogen II	389	28602..29768	67_GENE_4
**11233**	**26**	**CGL2_11233026**	Putative phage integrase family protein	314	complement(29784..30725)	67_GENE_3
**11233**	**27**	**CGL2_11233027**	Hypothetical protein	390	complement(30742..31911)	67_GENE_2
**11233**	**28**	**CGL2_11233028**	Protein of unknown function	455	complement(31935..33299)	62_GENE_28
**11233**	**29**	**CGL2_11233029**	Ferredoxin-nitrite reductase (EC 1.7.7.1)	547	33572..35212	62_GENE_27
**11233**	**30**	**CGL2_11233030**	Hypothetical protein	63	complement(35224..35412)	62_GENE_26
**11233**	**31**	**CGL2_11233031**	Conserved protein of unknown function	282	complement(35489..36334)	62_GENE_25
**11233**	**32**	**CGL2_11233032**	Glycogen synthase (EC 2.4.1.21)	493	36597..38075	62_GENE_24
**11233**	**33**	**CGL2_11233033**	transposase	133	complement(38375..38773)	62_GENE_23
**11233**	**34**	**CGL2_11233034**	Hypothetical protein	45	complement(39064..39198)	62_GENE_22
**11233**	**35**	**CGL2_11233035**	Putative transcriptional regulator, Crp/Fnr family	237	complement(39216..39926)	62_GENE_21
**11233**	**36**	**CGL2_11233036**	Putative peptidoglycan-binding lytic transglycosylase	473	40318..41736	62_GENE_20
**11233**	**37**	**CGL2_11233037**	Protein of unknown function	264	complement(41888..42679)	62_GENE_19
**11233**	**38**	**CGL2_11233038**	Probable ferredoxin	86	complement(42687..42944)	62_GENE_18
**11233**	**39**	**CGL2_11233039**	NADH dehydrogenase (Quinone) (EC 1.6.99.5)	453	complement(43053..44411)	62_GENE_17
**11233**	**40**	**CGL2_11233040**	Phosphoribosylaminoimidazole carboxylase, catalytic	175	44665..45189	62_GENE_16

**11233**	**41**	**CGL2_11233041**	Phosphoribosylaminoimidazole carboxylase, ATPase s	382	45189..46334	62_GENE_15
**11233**	**42**	**CGL2_11233042**	Conserved protein of unknown function	221	complement(46352..47014)	62_GENE_14
**11233**	**43**	**CGL2_11233043**	Protein of unknown function	136	complement(47165..47572)	62_GENE_13
**11233**	**44**	**CGL2_11233044**	Putative adenylate/guanylate cyclase	454	complement(48132..49493)	62_GENE_12
**11233**	**45**	**CGL2_11233045**	Hypothetical protein	483	49618..51066	62_GENE_11
**11233**	**46**	**CGL2_11233046**	Conserved protein of unknown function	374	51339..52460	62_GENE_10
**11233**	**47**	**CGL2_11233047**	Conserved hypothetical protein	338	52613..53626	62_GENE_9
**11233**	**48**	**CGL2_11233048**	Conserved hypothetical protein	132	53633..54028	62_GENE_8
**11233**	**49**	**CGL2_11233049**	Protein of unknown function	213	54121..54759	62_GENE_7
**11233**	**50**	**CGL2_11233050**	oxidoreductase/K+-channel protein?	58	54944..55117	62_GENE_6
**11233**	**51**	**CGL2_11233051**	Protein of unknown function	313	complement(55300..56238)	62_GENE_5
**11233**	**52**	**CGL2_11233052**	Putative ABC transporter	304	complement(56238..57149)	62_GENE_4
**11233**	**53**	**CGL2_11233053**	Putative ABC transporter	257	complement(57188..57958)	62_GENE_3
**11233**	**54**	**CGL2_11233054**	Probable ABC transporter, permease protein	261	complement(57981..58763)	62_GENE_2
**11233**	**55**	**CGL2_11233055**	Protein of unknown function	243	complement(58813..59541)	62_GENE_1
**11233**	**57**	**CGL2_11233057**	Cold shock proteins	68	complement(60195..60398)	140_GENE_1
**11233**	**58**	**CGL2_11233058**	Hypothetical protein	205	60718..61332	140_GENE_2
**11233**	**59**	**CGL2_11233059**	Probable general secretion pathway protein I	127	61332..61712	140_GENE_3
**11233**	**60**	**CGL2_11233060**	Probable general secretion pathway protein J	235	61712..62416	140_GENE_4
**11233**	**61**	**CGL2_11233061**	DnaB helicase	463	complement(62454..63842)	140_GENE_5
**11233**	**62**	**CGL2_11233062**	Histidyl-tRNA lygase (EC 6.1.1.21)	437	complement(63849..65159)	140_GENE_6
**11233**	**63**	**CGL2_11233063**	D-3-phosphoglycerate dehydrogenase (EC 1.1.1.95)	535	complement(65159..66763)	140_GENE_7
**11233**	**64**	**CGL2_11233064**	Serine--glyoxylate transaminase (EC 2.6.1.45)	381	complement(66785..67927)	140_GENE_8
**11233**	**65**	**CGL2_11233065**	Putative oligopeptide ABC transporter	533	complement(68134..69732)	140_GENE_9
11233rRNA		**CGL2_11233R004**	5S rRNA		complement(69908..69971)	
11233rRNA		**CGL2_11233R005**	23S rRNA		complement(70199..72844)	
11233tRNA		**CGL2_11233R003**	TRNA Ala TGC		complement(73172..73247)	
11233tRNA		**CGL2_11233R004**	TRNA Ile GAT		complement(73253..73329)	
**11277**	**2**	**CGL2_11277002**	Protein of unknown function	40	complement(239..358)	42_GENE_2
**11277**	**3**	**CGL2_11277003**	Protein of unknown function (probable hemolysin?)	455	562..1926	42_GENE_3
**11277**	**4**	**CGL2_11277004**	Protein of unknown function	189	1957..2523	42_GENE_4
**11277**	**5**	**CGL2_11277005**	Protein of unknown function	355	2548..3612	42_GENE_5
**11277**	**6**	**CGL2_11277006**	Transcriptional regulator, BadM/Rrf2 family	156	3698..4165	42_GENE_6
**11277**	**7**	**CGL2_11277007**	Cysteine desulfurase (EC 2.8.1.7)	404	4128..5339	42_GENE_7
**11277**	**8**	**CGL2_11277008**	FeS cluster assembly scaffold IscU	137	5380..5790	42_GENE_8
**11277**	**9**	**CGL2_11277009**	Iron-sulfur cluster assembly protein	109	5805..6131	42_GENE_9
**11277**	**10**	**CGL2_11277010**	Putative co-chaperone HscB	225	6228..6902	42_GENE_10
**11277**	**11**	**CGL2_11277011**	Chaperone protein HscA	603	6926..8734	42_GENE_11
**11277**	**12**	**CGL2_11277012**	Putative ferredoxin	103	8754..9062	42_GENE_13
**11277**	**13**	**CGL2_11277013**	Protein of unknown function	67	9098..9298	42_GENE_14

**11277**	**14**	**CGL2_11277014**	Conserved hypothetical protein	155	9298..9762	42_GENE_15
**11277**	**15**	**CGL2_11277015**	Putative ATP binding protein, Mrp like	358	9855..10928	42_GENE_16
**11277**	**16**	**CGL2_11277016**	Protein of unknown function	85	10928..11182	42_GENE_17
**11277**	**17**	**CGL2_11277017**	Pseudouridylate synthase (4.2.1.70)	268	11243..12046	42_GENE_18
**11277**	**18**	**CGL2_11277018**	Protein of unknown function	103	complement(12064..12372)	42_GENE_19
**11277**	**19**	**CGL2_11277019**	Glycerol 3-phosphate dehydrogenase (NADP) (1.1.1.	353	complement(12441..13499)	42_GENE_20
**11277**	**20**	**CGL2_11277020**	Probable phosphoribosyltransferase	223	13734..14402	42_GENE_21
**11277**	**21**	**CGL2_11277021**	Protein of unknown function	403	14518..15726	42_GENE_22
**11277**	**22**	**CGL2_11277022**	Putative diguanylate cyclase/phosphodiesterase with	884	complement(15720..18371)	42_GENE_23
**11277**	**23**	**CGL2_11277023**	Putative phosphatidylethanolamine binding protein	188	complement(18424..18987)	42_GENE_24
**11277**	**24**	**CGL2_11277024**	Seryl-tRNA synthetase (6.1.1.11)	442	19309..20634	42_GENE_25
**11277**	**25**	**CGL2_11277025**	Protein of unknown function	123	20634..21002	42_GENE_26
**11277**	**26**	**CGL2_11277026**	Protein of unknown function	241	20989..21711	42_GENE_27
**11277**	**27**	**CGL2_11277027**	Hypothetical protein	84	21749..22000	42_GENE_27a
**11277**	**28**	**CGL2_11277028**	Putative GTP binding protein	518	22111..23664	42_GENE_29
**11277**	**29**	**CGL2_11277029**	Hypothetical protein	163	23669..24157	42_GENE_30
**11277**	**30**	**CGL2_11277030**	Hypothetical protein	333	24160..25158	42_GENE_31
**11277**	**31**	**CGL2_11277031**	Protein of unknown function	140	25253..25672	42_GENE_32
**11277**	**32**	**CGL2_11277032**	Putative peptidase M16	476	25738..27165	42_GENE_33
**11277**	**33**	**CGL2_11277033**	Putative peptidase M16	481	27196..28638	42_GENE_34
**11277**	**34**	**CGL2_11277034**	Hypothetical protein	430	complement(28705..29994)	42_GENE_35
**11277**	**35**	**CGL2_11277035**	Putative Na+/H+ antiporters	406	complement(30044..31261)	42_GENE_36
**11277**	**36**	**CGL2_11277036**	Putative metabolite transport protein	429	31715..33001	42_GENE_37
**11277**	**37**	**CGL2_11277037**	Conserved hypothetical protein	101	complement(33490..33792)	42_GENE_38
**11277**	**38**	**CGL2_11277038**	Hypothetical protein	102	34138..34443	42_GENE_39
**11277**	**39**	**CGL2_11277039**	Hypothetical protein	93	34446..34724	42_GENE_40
**11277**	**40**	**CGL2_11277040**	Hypothetical protein	261	complement(34681..35463)	42_GENE_41
**11277**	**41**	**CGL2_11277041**	Hypothetical protein	187	complement(35623..36183)	42_GENE_44
**11277**	**42**	**CGL2_11277042**	Hypothetical protein	111	complement(36183..36515)	42_GENE_45
**11277**	**43**	**CGL2_11277043**	Conserved hypothetical protein	83	complement(36515..36763)	42_GENE_47
**11277**	**44**	**CGL2_11277044**	Conserved hypothetical protein	588	complement(36892..38655)	21_GENE_2
**11277**	**45**	**CGL2_11277045**	Conserved hypothetical protein	307	complement(38648..39568)	21_GENE_3
**11277**	**46**	**CGL2_11277046**	Hypothetical protein	270	39766..40575	21_GENE_5
**11277**	**47**	**CGL2_11277047**	Integrase	367	40580..41680	21_GENE_6
11277tRNA		**CGL2_11277R001**	TRNA Arg ACG		complement(41698..41774)	
**11277**	**48**	**CGL2_11277048**	Leucyl-tRNA synthetase (6.1.1.4)	828	42055..44538	21_GENE_8
**11277**	**49**	**CGL2_11277049**	Protein of unknown function	239	44538..45254	21_GENE_9
**11277**	**50**	**CGL2_11277050**	Probable DNA polymerase III, delta subunit	348	45254..46297	21_GENE_10
**11277**	**51**	**CGL2_11277051**	Ribosomal protein S20	87	complement(46287..46547)	21_GENE_11
**11277**	**52**	**CGL2_11277052**	Putative virulence factor MVN like	547	46631..48271	21_GENE_12
**11277**	**53**	**CGL2_11277053**	Probable HNH endonuclease	186	complement(48285..48842)	21_GENE_13

**11277**	**54**	**CGL2_11277054**	Protein of unknown function	347	complement(48906..49946)	21_GENE_14
**11277**	**55**	**CGL2_11277055**	Protein of unknown function	163	50116..50604	21_GENE_15
**11277**	**56**	**CGL2_11277056**	Protein of unknown function	116	50686..51036	21_GENE_16
**11277**	**57**	**CGL2_11277057**	Probable ATPase, PP-loop superfamily implicat	307	51036..51956	21_GENE_17
**11277**	**58**	**CGL2_11277058**	Conserved protein of unknown function	324	52008..52979	21_GENE_18
**11277**	**59**	**CGL2_11277059**	Probable metal-dependent phosphohydrolase	424	complement(52982..54253)	21_GENE_19
**11277**	**60**	**CGL2_11277060**	Protein of unknown funciton, but transposase (delete	105	54888..55202	21_GENE_20
11277tRNA		**CGL2_11277R002**	TRNA His GTG		complement(55579..55653)	
**11277**	**62**	**CGL2_11277062**	Anthranilate synthases component I (EC 4.1.3.27)	441	55759..57080	21_GENE_21
**11277**	**64**	**CGL2_11277064**	Putative branched chain amino acid aminotransferas	295	57072..57956	21_GENE_22
**11277**	**65**	**CGL2_11277065**	Rubrerythrin	139	58187..58603	21_GENE_23
**11277**	**66**	**CGL2_11277066**	Ribonucleotide reductase (EC 1.17.4.1)	732	complement(58849..61044)	21_GENE_24
**11277**	**67**	**CGL2_11277067**	Protein of unknown function	484	complement(61262..62713)	21_GENE_25
**11277**	**68**	**CGL2_11277068**	Protein of unknown function	205	complement(62854..63468)	21_GENE_26
**11277**	**69**	**CGL2_11277069**	Putative acriflavin resistance protein	386	complement(63783..64940)	21_GENE_27
**11277**	**70**	**CGL2_11277070**	Putative acriflavin resistance protein	664	complement(64959..66950)	21_GENE_28
11277tRNA		**CGL2_11277R003**	TRNA Arg TCG		complement(67254..67330)	
**11277**	**71**	**CGL2_11277071**	Protein of unknown function	152	67577..68032	21_GENE_31
11277tRNA		**CGL2_11277R004**	TRNA Cys GCA		68110..68184	
**11277**	**72**	**CGL2_11277072**	Putative major facilitator superfamily transporter	487	complement(68293..69753)	21_GENE_32
**11277**	**73**	**CGL2_11277073**	L-2,4-diaminobutyric acid acetyltransferase	187	70464..71024	21_GENE_34
**11277**	**74**	**CGL2_11277074**	Diaminobutyrate--pyruvate aminotransferase (EC 2.6	425	71048..72322	21_GENE_35
**11277**	**75**	**CGL2_11277075**	Ectoine synthase	131	72349..72741	21_GENE_36
**11277**	**76**	**CGL2_11277076**	Ectoine hydroxylase	305	72819..73733	21_GENE_37
**11277**	**77**	**CGL2_11277077**	Protein of unknown function	147	complement(73776..74216)	21_GENE_38
**11277**	**78**	**CGL2_11277078**	Hypothetical protein	153	complement(74253..74711)	21_GENE_39
**11277**	**79**	**CGL2_11277079**	Hypothetical protein	67	74818..75018	21_GENE_40
**11277**	**80**	**CGL2_11277080**	Probable sodium/calcium exchanger membrane regic	303	75018..75926	21_GENE_41
**11277**	**81**	**CGL2_11277081**	Probable transporter protein	406	75926..77143	21_GENE_42
**11277**	**82**	**CGL2_11277082**	Hypothetical protein	214	complement(77134..77775)	21_GENE_43
**11277**	**83**	**CGL2_11277083**	Putative proteasome component	448	complement(77823..79166)	21_GENE_44
**11277**	**84**	**CGL2_11277084**	Probable 20S proteasome alpha-subunit	229	complement(79193..79879)	21_GENE_45
**11277**	**85**	**CGL2_11277085**	Putative 20S proteasome beta-subunit	279	complement(79879..80715)	21_GENE_46
**11277**	**86**	**CGL2_11277086**	Conserved protein of unknown function	70	complement(80734..80943)	21_GENE_47
**11277**	**87**	**CGL2_11277087**	Putative proteasome component	505	complement(80969..82483)	21_GENE_48
**11277**	**88**	**CGL2_11277088**	Vesicle-fusing ATPase (EC 3.6.4.6)	579	complement(82504..84240)	21_GENE_49
**11277**	**89**	**CGL2_11277089**	Peroxiredoxin	158	complement(84432..84905)	21_GENE_51
**11277**	**90**	**CGL2_11277090**	Putative band 7 family protein	286	complement(84982..85839)	21_GENE_52
**11277**	**91**	**CGL2_11277091**	Protein of unknown function	202	complement(85874..86479)	21_GENE_53
**11277**	**92**	**CGL2_11277092**	Protein of unknown function	93	complement(86487..86765)	21_GENE_54
**11277**	**93**	**CGL2_11277093**	Protein of unknown function	115	87064..87408	21_GENE_56

**11277**	**94**	**CGL2_11277094**	Alcohol dehydrogenase (EC 1.1.1.1)	334	complement(87473..88474)	21_GENE_57
**11277**	**95**	**CGL2_11277095**	Protein of unknown function	72	complement(88519..88734)	21_GENE_58
**11277**	**96**	**CGL2_11277096**	Putative acriflavin resistance protein	1087	complement(88826..92086)	21_GENE_59
**11277**	**97**	**CGL2_11277097**	Efflux transporter, RND family, MFP subunit	349	complement(92086..93132)	32_GENE_53
**11277**	**98**	**CGL2_11277098**	Putative outer membrane efflux protein	472	complement(93193..94608)	32_GENE_52
**11277**	**99**	**CGL2_11277099**	Putative transcriptional regulator, ArsR family	112	complement(94608..94943)	32_GENE_51
**11277**	**100**	**CGL2_11277100**	Hypothetical protein	89	95205..95471	32_GENE_50
**11277**	**101**	**CGL2_11277101**	Exinuclease ABC, B subunit (UvrB)	701	complement(95482..97584)	32_GENE_49
**11277**	**102**	**CGL2_11277102**	Conserved hypothetical protein	117	complement(97584..97934)	32_GENE_48
**11277**	**103**	**CGL2_11277103**	Protein of unknown function	239	97991..98707	32_GENE_47
**11277**	**104**	**CGL2_11277104**	Aminopeptidase N (EC 3.4.11.2)	863	complement(98735..101323)	32_GENE_46
**11277**	**105**	**CGL2_11277105**	Putative thioredoxin	108	complement(101372..101695)	32_GENE_45
**11277**	**106**	**CGL2_11277106**	Conserved protein of unknown function	253	101887..102645	32_GENE_44
**11277**	**107**	**CGL2_11277107**	Protein of unknown function	234	102658..103359	32_GENE_43
**11277**	**108**	**CGL2_11277108**	Probable cytochrome b561 family	253	103465..104223	32_GENE_42
**11277**	**109**	**CGL2_11277109**	Putative diguanylate phosphodiesterase	416	complement(104254..105501)	32_GENE_41
**11277**	**110**	**CGL2_11277110**	Copper translocating P-type ATPase (EC 3.6.3.4)	843	105624..108152	32_GENE_40
**11277**	**111**	**CGL2_11277111**	Ammonium transporter	435	complement(108233..109537)	32_GENE_39
**11277**	**112**	**CGL2_11277112**	Nitrogen regulatory protein P-II	112	complement(109608..109943)	32_GENE_38
**11277**	**113**	**CGL2_11277113**	Ammonium transporter	490	complement(110013..111482)	32_GENE_37
**11277**	**114**	**CGL2_11277114**	Protein of unknown function	514	complement(111609..113150)	32_GENE_36
**11277**	**115**	**CGL2_11277115**	Hypothetical protein	154	113548..114009	32_GENE_35
**11277**	**116**	**CGL2_11277116**	Cystathionine gamma-synthase (EC 2.5.1.48)	401	complement(114028..115230)	32_GENE_34
**11277**	**117**	**CGL2_11277117**	Protein of unknown function	365	115366..116460	32_GENE_33
**11277**	**118**	**CGL2_11277118**	Putative oligopeptide ABC transporter	303	complement(116491..117399)	32_GENE_32
**11277**	**119**	**CGL2_11277119**	Putative oligopeptide ABC transporter	283	complement(117399..118247)	32_GENE_31
**11277**	**120**	**CGL2_11277120**	Hypothetical protein	90	complement(118250..118519)	32_GENE_29
**11277**	**121**	**CGL2_11277121**	Protein of unknown function	126	complement(118522..118899)	32_GENE_28
**11277**	**122**	**CGL2_11277122**	Protein of unknown function	116	complement(118957..119304)	32_GENE_27
**11277**	**123**	**CGL2_11277123**	Protein of unknown function	208	complement(119294..119917)	32_GENE_26
**11277**	**124**	**CGL2_11277124**	Putative glycosyl hydrolase, BNR repeat	352	complement(120032..121087)	32_GENE_25
**11277**	**125**	**CGL2_11277125**	Hypothetical protein	42	complement(121087..121212)	
**11277**	**126**	**CGL2_11277126**	Hypothetical protein	18	complement(121209..121262)	
**11277**	**126a**	**CGL2_11277126a**	Transposase	446	complement(121576..122913)	
**11277**	**127**	**CGL2_11277127**	Protein of unknown function	223	complement(123460..124128)	32_GENE_24
**11277**	**128**	**CGL2_11277128**	Putative haloacid dehalogenase-like hydrolase	232	complement(124353..125048)	32_GENE_23
**11277**	**129**	**CGL2_11277129**	Probable hexapeptide transferase family protein	177	125210..125740	32_GENE_22
**11277**	**130**	**CGL2_11277130**	Putative periplasmic sensor signal transduction histid	546	125800..127437	32_GENE_21
**11277**	**131**	**CGL2_11277131**	Putative response regulator receiver protein	148	127459..127902	32_GENE_20
**11277**	**132**	**CGL2_11277132**	Peptidase S1C, Do	500	127946..129445	32_GENE_19
**11277**	**133**	**CGL2_11277133**	Probable crossover junction endodeoxyribonuclease (	158	129486..129959	32_GENE_18

**11277**	**134**	**CGL2_11277134**	Probable holliday junction DNA helicase (RuvA)	208	129959..130582	32_GENE_17
**11277**	**135**	**CGL2_11277135**	Holliday junction DNA helicase (RuvB)	332	130572..131567	32_GENE_16
**11277**	**136**	**CGL2_11277136**	Protein of unknown function	241	complement(131580..132302)	32_GENE_15
**11277**	**137**	**CGL2_11277137**	Protein of unknown function	277	132450..133280	32_GENE_14
**11277**	**138**	**CGL2_11277138**	Protein of unknown function	308	133280..134203	32_GENE_13
**11277**	**139**	**CGL2_11277139**	Conserved hypothetical protein	51	complement(134216..134368)	32_GENE_12
**11277**	**140**	**CGL2_11277140**	Conserved hypothetical protein	100	complement(134323..134622)	32_GENE_11
**11277**	**141**	**CGL2_11277141**	Probable prevent-host-death protein	64	complement(134673..134864)	32_GENE_10
**11277**	**142**	**CGL2_11277142**	Hypothetical protein	105	135141..135455	32_GENE_9
**11277**	**143**	**CGL2_11277143**	Putative antibiotic biosynthesis monooxygenase	104	136077..136388	32_GENE_8
**11277**	**144**	**CGL2_11277144**	Probable thiamine biosynthesis protein	328	136388..137371	32_GENE_7
**11277**	**145**	**CGL2_11277145**	Probable radical SAM family protein	311	137362..138294	32_GENE_6
**11277**	**146**	**CGL2_11277146**	Conserved protein of unknown function	109	complement(138672..138998)	32_GENE_5
**11277**	**147**	**CGL2_11277147**	Probable oxidoreductase FAD/NAD(P)-binding protein	229	complement(139091..139777)	32_GENE_4
**11277**	**148**	**CGL2_11277148**	Putative major facilitator superfamily transporter	418	140056..141309	32_GENE_1
**11277**	**149**	**CGL2_11277149**	Protein of unknown function	244	complement(141338..142069)	
**11277**	**150**	**CGL2_11277150**	Protein of unknown function	255	142172..142936	630_GENE_3
**11277**	**151**	**CGL2_11277151**	Transposase	411	143224..144456	630_GENE_4
**11277**	**153**	**CGL2_11277153**	Protein of unknown function (cyt 572, see full length	576	complement(144648..146375)	630_GENE_6
		**Strain variant path**	**Strain variant path**			**Strain variant path**
**11277**	**154**	**CGL2_11277154**	Transposase	446	complement(147058..148395)	815_GENE_1
11277tRNA		**CGL2_11277R005**	TRNA Leu CAA		148692..148778	
**11277**	**155**	**CGL2_11277155**	Integrase	185	149319..149873	815_GENE_2
**11277**	**156**	**CGL2_11277156**	Hypothetical protein	63	150116..150304	815_GENE_3
**11277**	**157**	**CGL2_11277157**	Hypothetical protein	60	complement(150474..150653)	815_GENE_4
**11277**	**158**	**CGL2_11277158**	Protein of unknown function	1140	complement(150680..154099)	815_GENE_5
**11277**	**159**	**CGL2_11277159**	Hypothetical protein	63	complement(154104..154292)	600_GENE_2
**11277**	**160**	**CGL2_11277160**	Hypothetical protein	36	complement(154300..154407)	600_GENE_3
**11277**	**161**	**CGL2_11277161**	Hypothetical protein	150	complement(154407..154856)	600_GENE_4
**11277**	**162**	**CGL2_11277162**	conjugation TrwB	456	complement(154980..156347)	600_GENE_5
**11277**	**163**	**CGL2_11277163**	plasmid coupling TrwB	425	complement(156353..157627)	185_GENE_1
**11277**	**164**	**CGL2_11277164**	Hypothetical protein	138	complement(157645..158058)	185_GENE_2
**11277**	**165**	**CGL2_11277165**	Hypothetical protein	180	complement(158058..158597)	185_GENE_3
**11277**	**166**	**CGL2_11277166**	Hypothetical protein	85	complement(158751..159005)	185_GENE_4
**11277**	**167**	**CGL2_11277167**	Hypothetical protein	75	complement(159005..159229)	185_GENE_5
**11277**	**168**	**CGL2_11277168**	Hypothetical protein	82	complement(159229..159474)	185_GENE_6
**11277**	**169**	**CGL2_11277169**	Hypothetical protein	43	complement(159518..159646)	185_GENE_7
**11277**	**170**	**CGL2_11277170**	Hypothetical protein	104	complement(159661..159972)	185_GENE_8
**11277**	**171**	**CGL2_11277171**	Protein of unknown function	45	160002..160136	185_GENE_9
**11277**	**172**	**CGL2_11277172**	Hypothetical protein	43	160097..160225	185_GENE_10
**11277**	**173**	**CGL2_11277173**	Hypothetical protein	63	160422..160610	185_GENE_12

**11277**	**174**	**CGL2_11277174**	Protein of unknown function	155	160628..161092	185_GENE_13
**11277**	**174a**	**CGL2_11277174a**	Hypothetical protein	113	complement(161100..161438)	
**11277**	**175**	**CGL2_11277175**	Hypothetical protein	302	complement(161562..162467)	185_GENE_14
**11277**	**176**	**CGL2_11277176**	biopolymer transport, ExbD	147	complement(162562..163002)	185_GENE_15
**11277**	**177**	**CGL2_11277177**	TonB	509	complement(163575..165101)	185_GENE_16
**11277**	**178**	**CGL2_11277178**	TolQ	236	complement(165245..165952)	185_GENE_18
**11277**	**179**	**CGL2_11277179**	Protein of unknown function	500	complement(166046..167545)	185_GENE_19
**11277**	**180**	**CGL2_11277180**	Hypothetical protein	54	complement(167638..167799)	185_GENE_20
**11277**	**181**	**CGL2_11277181**	Hypothetical protein	146	complement(167795..168232)	185_GENE_21
**11277**	**182**	**CGL2_11277182**	Conserved protein of unknown function	577	complement(168204..169934)	185_GENE_22
**11277**	**183**	**CGL2_11277183**	Probable transposase	213	complement(170344..170982)	173_GENE_16
**11277**	**184**	**CGL2_11277184**	Transposase	431	complement(170975..172267)	173_GENE_15
**11277**	**185**	**CGL2_11277185**	Protein of unknown function	620	complement(172511..174370)	173_GENE_14
**11277**	**186**	**CGL2_11277186**	Conserved hypothetical protein	161	complement(174395..174877)	173_GENE_13
**11277**	**187**	**CGL2_11277187**	Hypothetical protein	82	complement(174885..175130)	173_GENE_12
**11277**	**188**	**CGL2_11277188**	IstB helper protein/transposase	259	complement(175186..175962)	173_GENE_11
**11277**	**189**	**CGL2_11277189**	transposase	440	complement(175965..177284)	173_GENE_10
**11277**	**189a**	**CGL2_11277189a**	Hypothetical protein	126	complement(177336..177713)	
**11277**	**190**	**CGL2_11277190**	Conserved protein of unknown function	221	complement(178159..178821)	173_GENE_9
**11277**	**191**	**CGL2_11277191**	Hypothetical protein	151	complement(179031..179483)	173_GENE_7
**11277**	**191a**	**CGL2_11277191a**	Hypothetical protein	138	complement(179587..180000)	
**11277**	**192**	**CGL2_11277192**	transposase	102	complement(180152..180457)	173_GENE_5
**11277**	**193**	**CGL2_11277193**	transposase	135	complement(180442..180846)	173_GENE_4
**11277**	**194**	**CGL2_11277194**	Hypothetical protein	225	complement(180864..181538)	173_GENE_3
**11277**	**195**	**CGL2_11277195**	Hypothetical protein	73	complement(181573..181791)	173_GENE_2
**11277**	**196**	**CGL2_11277196**	Putative replicative helicase	448	complement(181769..183112)	173_GENE_1
**11277**	**196a**	**CGL2_11277196a**	Hypothetical protein	75	complement(183112..183336)	
**11277**	**197a**	**CGL2_11277197a**	Hypothetical protein	110	complement(183336..183665)	
**11277**	**197**	**CGL2_11277197**	Hypothetical protein	80	complement(183777..184016)	374_GENE_1
**11277**	**198**	**CGL2_11277198**	Protein of unknown function	231	184124..184816	374_GENE_2
**11277**	**199**	**CGL2_11277199**	Transposase	146	complement(185024..185461)	374_GENE_3
**11277**	**200**	**CGL2_11277200**	Probable phage integrase, pseudo	123	185866..186234	374_GENE_4a
**11277**	**201**	**CGL2_11277201**	Protein of unknown function	113	complement(186368..186706)	374_GENE_5
**11277**	**202**	**CGL2_11277202**	Protein of unknown function	217	complement(186712..187362)	374_GENE_6
**11277**	**203**	**CGL2_11277203**	Protein of unknown function	163	complement(187774..188262)	374_GENE_7
**11277**	**204**	**CGL2_11277204**	Protein of unknown function	371	complement(188410..189522)	374_GENE_8
**11277**	**205**	**CGL2_11277205**	Conserved hypothetical protein	326	complement(189531..190508)	374_GENE_9
**11277**	**206**	**CGL2_11277206**	Probable TPR-domain containing protein	176	complement(190718..191245)	374_GENE_10
**11277**	**207**	**CGL2_11277207**	Conserved protein of unknown function	95	complement(191933..192217)	4_GENE_100
**11277**	**208**	**CGL2_11277208**	Putative carbon-nitrogen hydrolase	273	complement(192294..193112)	4_GENE_99
**11277**	**209**	**CGL2_11277209**	Pantoate-beta-alanine ligase (EC 6.3.2.1)	286	193191..194048	4_GENE_98

**11277**	**210**	**CGL2_11277210**	Putative tetrapyrrole methylase family protein	282	194044..194889	4_GENE_97
**11277**	**211**	**CGL2_11277211**	Deoxyuridine-triphosphatase (EC 3.6.1.23)	157	complement(194931..195401)	4_GENE_96
**11277**	**212**	**CGL2_11277212**	Protein of unknown function	156	complement(195401..195868)	4_GENE_95
**11277**	**213**	**CGL2_11277213**	Acetylglutamate kinase (EC 2.7.2.8)	290	complement(195858..196727)	4_GENE_94
**11277**	**214**	**CGL2_11277214**	Heat shock protein HslU	444	complement(196784..198115)	4_GENE_93
**11277**	**215**	**CGL2_11277215**	ATP-dependent protease HslVU (ClpYQ), peptidase su	180	complement(198115..198654)	4_GENE_92
**11277**	**216**	**CGL2_11277216**	Putative phage integrase family protein	321	complement(198695..199657)	4_GENE_91
**11277**	**218**	**CGL2_11277218**	Putaive glucose-inhibited division protein A	427	complement(199650..200930)	4_GENE_90
**11277**	**219**	**CGL2_11277219**	DNA topoisomerase (EC 5.99.1.2)	850	complement(201028..203577)	4_GENE_89
**11277**	**220**	**CGL2_11277220**	DNA processing protein DprA	306	complement(203549..204466)	4_GENE_88
**11277**	**221**	**CGL2_11277221**	Protein of unknown function	90	204560..204829	4_GENE_87
**11277**	**222**	**CGL2_11277222**	Putative ABC transporter ATP-binding protein	160	complement(204832..205311)	4_GENE_86
**11277**	**223**	**CGL2_11277223**	Sulfate-transporting ATPase (EC 3.6.3.25)	252	complement(205379..206134)	4_GENE_85
**11277**	**224**	**CGL2_11277224**	Putative transmembrane transporter	577	complement(206130..207860)	4_GENE_84
**11277**	**225**	**CGL2_11277225**	Protein of unknown function	137	207968..208378	4_GENE_83
**11277**	**226**	**CGL2_11277226**	Protein of unknown function	107	208414..208734	4_GENE_82
**11277**	**227**	**CGL2_11277227**	Protein of unknown function	175	complement(208761..209285)	4_GENE_81
**11277**	**228**	**CGL2_11277228**	Hypothetical protein	362	complement(209466..210551)	4_GENE_80
**11277**	**229**	**CGL2_11277229**	Putative A/G-specific DNA glycosylase	355	complement(210551..211615)	4_GENE_79
**11277**	**230**	**CGL2_11277230**	Hypothetical protein	245	complement(211605..212339)	4_GENE_78
**11277**	**231**	**CGL2_11277231**	Putative 2-amino-4-hydroxy-6-hydroxymethyldihydr	175	complement(212384..212908)	4_GENE_77
**11277**	**232**	**CGL2_11277232**	Aspartate aminotransferase (EC 2.6.1.1)	394	complement(212911..214092)	4_GENE_76
**11277**	**233**	**CGL2_11277233**	Hypothetical protein	237	complement(214273..214983)	4_GENE_75
**11277**	**234**	**CGL2_11277234**	Hypothetical protein	358	complement(215023..216096)	4_GENE_74
**11277**	**235**	**CGL2_11277235**	Hypothetical protein	149	complement(216216..216662)	4_GENE_73
**11277**	**236**	**CGL2_11277236**	Hypothetical protein	152	complement(216662..217117)	4_GENE_72
**11277**	**237**	**CGL2_11277237**	Hypothetical protein	165	complement(217193..217687)	4_GENE_70a
**11277**	**238**	**CGL2_11277238**	3-oxoacyl-[acyl-carrier-protein] reductase (1.1.1.10(	235	217903..218607	4_GENE_70
**11277**	**239**	**CGL2_11277239**	Probable flagellar biosynthesis protein	100	complement(218585..218884)	4_GENE_69
**11277**	**240**	**CGL2_11277240**	Hypothetical protein	271	complement(218884..219696)	4_GENE_68
**11277**	**241**	**CGL2_11277241**	Protein of unknown function	186	complement(219715..220272)	4_GENE_67
**11277**	**242**	**CGL2_11277242**	Hypothetical protein	96	complement(220305..220592)	4_GENE_66
**11277**	**243**	**CGL2_11277243**	Putative flagellar motor protein (OmpA/MotB)	261	complement(220616..221398)	4_GENE_65
**11277**	**244**	**CGL2_11277244**	Putative flagellar motor component (MotA)	260	complement(221410..222189)	4_GENE_64
**11277**	**245**	**CGL2_11277245**	Putative chemotaxis protein (CheW)	171	complement(222221..222733)	4_GENE_63
**11277**	**246**	**CGL2_11277246**	Putative methyl-accepting chemotaxis sensory trans(	557	complement(222768..224438)	4_GENE_62
**11277**	**247**	**CGL2_11277247**	Protein-glutamate methylesterase (CheB) (EC 3.1.1.	344	complement(224494..225525)	4_GENE_61
**11277**	**248**	**CGL2_11277248**	Putative signal transduction histidine kinase (CheA)	646	complement(225538..227475)	4_GENE_60
**11277**	**249**	**CGL2_11277249**	Putative response regulator receiver protein (CheY)	126	complement(227520..227897)	4_GENE_59
**11277**	**250**	**CGL2_11277250**	Protein-glutamate O-methyltransferase (CheR) (EC 2	264	complement(228021..228812)	4_GENE_58
**11277**	**251**	**CGL2_11277251**	Putative RNA polymerase sigma 28 subunit	251	complement(228855..229607)	4_GENE_57

**11277**	**252**	**CGL2_11277252**	Protein of unknown function	292	complement(229668..230543)	4_GENE_56
**11277**	**253**	**CGL2_11277253**	flagellar biosynthesis protein FlhF	393	complement(230547..231725)	4_GENE_55
**11277**	**254**	**CGL2_11277254**	possible flagellar biosynthetic protein FlhA	702	complement(231732..233837)	4_GENE_54
**11277**	**255**	**CGL2_11277255**	flagellar biosynthesis protein, FlhB	362	complement(233821..234906)	4_GENE_53
**11277**	**256**	**CGL2_11277256**	flagellar biosynthesis protein, FliR	262	complement(234899..235684)	4_GENE_52
**11277**	**257**	**CGL2_11277257**	Chemotaxis/flagella (novel lepto genera)	112	complement(235684..236019)	4_GENE_51
**11277**	**258**	**CGL2_11277258**	Flagellar biosynthesis protein FliP	265	complement(236083..236877)	4_GENE_50
**11277**	**259**	**CGL2_11277259**	Protein of unknown function	173	complement(236868..237386)	4_GENE_49
**11277**	**260**	**CGL2_11277260**	flagellar motor switch protein FliN	125	complement(237419..237793)	4_GENE_48
**11277**	**261**	**CGL2_11277261**	Putative flagellar basal body-associated protein FliL	168	complement(237833..238336)	4_GENE_47
**11277**	**262**	**CGL2_11277262**	Probable flagellar hook protein FlgE	457	complement(238500..239870)	4_GENE_46
**11277**	**263**	**CGL2_11277263**	Probable flagellar hook capping protein FlgD	276	complement(239875..240702)	4_GENE_45
**11277**	**264**	**CGL2_11277264**	Conserved protein of unknown function	494	complement(240733..242214)	4_GENE_44
**11277**	**265**	**CGL2_11277265**	Protein of unknown function	169	complement(242242..242748)	4_GENE_43
**11277**	**266**	**CGL2_11277266**	Protein of unknown function	147	complement(242748..243188)	4_GENE_42
**11277**	**267**	**CGL2_11277267**	Flagellum-specific ATP synthase FliI (EC 3.6.3.14)	462	complement(243191..244576)	4_GENE_41
**11277**	**268**	**CGL2_11277268**	Protein of unknown function	219	complement(244576..245232)	4_GENE_40
**11277**	**269**	**CGL2_11277269**	Flagellar motor switch protein FliG	334	complement(245263..246264)	4_GENE_39
**11277**	**270**	**CGL2_11277270**	Flagellar M-ring protein FliF	531	complement(246292..247884)	4_GENE_38
**11277**	**271**	**CGL2_11277271**	Flagellar hook-basal body complex protein FliE	109	complement(247936..248262)	4_GENE_37
**11277**	**272**	**CGL2_11277272**	Flagellar basal-body rod protein FlgC	145	complement(248308..248742)	4_GENE_36
**11277**	**273**	**CGL2_11277273**	Putative flagellar basal-body rod protein FlgB	144	complement(248748..249179)	4_GENE_35
**11277**	**274**	**CGL2_11277274**	Putative two component, sigma54 specific, transcript	489	complement(249198..250664)	4_GENE_34
**11277**	**275**	**CGL2_11277275**	Putative PAS/PAC sensor signal transduction histidine	406	complement(250664..251881)	4_GENE_33
**11277**	**276**	**CGL2_11277276**	Protein of unknown function	719	complement(251900..254056)	4_GENE_32
**11277**	**277**	**CGL2_11277277**	Putative sigma54 specific transcriptional regulator, F	344	complement(254233..255264)	4_GENE_31
**11277**	**278**	**CGL2_11277278**	Probable iron-sulfur cluster binding protein	342	complement(255269..256294)	4_GENE_30
**11277**	**279**	**CGL2_11277279**	Probable metal dependent phosphohydrolase	281	complement(256294..257136)	4_GENE_29
**11277**	**280**	**CGL2_11277280**	Probable metal dependent phosphohydrolase	280	complement(257190..258029)	4_GENE_28
**11277**	**281**	**CGL2_11277281**	Protein of unknown function	110	complement(258070..258399)	4_GENE_27
**11277**	**282**	**CGL2_11277282**	Probable lipoate protein ligase	209	complement(258399..259025)	4_GENE_26
**11277**	**283**	**CGL2_11277283**	Glycine dehydrogenase (Decarboxylating) subunit 2	496	complement(259038..260525)	4_GENE_25
**11277**	**284**	**CGL2_11277284**	Glycine dehydrogenase (Decarboxylating) subunit 1	453	complement(260525..261883)	4_GENE_24
**11277**	**285**	**CGL2_11277285**	Glycine cleavage system H protein	134	complement(261879..262280)	4_GENE_23
**11277**	**286**	**CGL2_11277286**	Glycine cleavage system T protein (EC 2.1.2.10)	377	complement(262394..263524)	4_GENE_22
**11277**	**287**	**CGL2_11277287**	Protein of unknown function	234	264038..264739	4_GENE_21
**11277**	**288**	**CGL2_11277288**	Putative methyl-accepting chemotaxis sensory transd	454	complement(264821..266182)	4_GENE_20
**11277**	**289**	**CGL2_11277289**	Putative methyl-accepting chemotaxis sensory transd	158	complement(266088..266561)	4_GENE_19
**11277**	**290**	**CGL2_11277290**	Protein of unknown function	184	complement(266571..267122)	4_GENE_18
**11277**	**291**	**CGL2_11277291**	Probable ABC transporter, periplasmic component	339	267338..268354	4_GENE_17
**11277**	**292**	**CGL2_11277292**	Protein of unknown function	467	268367..269767	4_GENE_16

**11277**	**293**	**CGL2_11277293**	Putative rhodanese-like domain protein	114	269829..270170	4_GENE_15
**11277**	**294**	**CGL2_11277294**	Protein of unknown function	101	complement(270246..270548)	4_GENE_14
**11277**	**295**	**CGL2_11277295**	Putative TRNA (1-methyladenosine) methyltransfera:	276	270705..271532	4_GENE_13
**11184**	**1**	**CGL2_11184001**	Putative methyltransferase	214	complement(73..714)	4_GENE_12
**11184**	**2**	**CGL2_11184002**	NHL repeat domain protein	494	847..2328	4_GENE_11
**11184**	**3**	**CGL2_11184003**	Protein of unknown function	436	2404..3711	4_GENE_10
**11184**	**4**	**CGL2_11184004**	Hypothetical protein	67	3820..4020	4_GENE_9
**11184**	**5**	**CGL2_11184005**	Diaminopimelate epimerase (EC 5.1.1.7)	290	complement(4043..4912)	4_GENE_8
**11184**	**6**	**CGL2_11184006**	Protein of unknown function	284	complement(4951..5802)	4_GENE_7
**11184**	**7**	**CGL2_11184007**	Probable short-chain dehydrogenase/reductase SDR	247	complement(5798..6538)	4_GENE_6
**11184**	**8**	**CGL2_11184008**	Putative transcriptional regulators, TraR/DksA family	127	complement(6538..6918)	4_GENE_5
**11184**	**9**	**CGL2_11184009**	Putative catalytic LigB subunit of aromatic ring-openi	291	7054..7926	4_GENE_4
**11184**	**10**	**CGL2_11184010**	Heavy metal efflux pump (CzcA)	1036	complement(7845..10952)	4_GENE_3
**11184**	**11**	**CGL2_11184011**	Secretion protein (HlyD)	380	complement(10955..12094)	4_GENE_2
**11184**	**12**	**CGL2_11184012**	Probable outer membrane efflux protein	458	complement(12109..13482)	59_GENE_29
**11184**	**13**	**CGL2_11184013**	Protein of unknown function	206	complement(13836..14453)	59_GENE_28
**11184**	**14**	**CGL2_11184014**	Conserved protein of unknown function	281	complement(14453..15295)	59_GENE_27
**11184**	**15**	**CGL2_11184015**	Protein of unknown function	402	15515..16720	59_GENE_26
**11184**	**16**	**CGL2_11184016**	Protein of unknown function	82	16894..17139	59_GENE_24a
**11184**	**17**	**CGL2_11184017**	Protein of unknown function	100	17174..17473	59_GENE_24
**11184**	**18**	**CGL2_11184018**	Protein of unknown function	122	17491..17856	59_GENE_23
**11184**	**19**	**CGL2_11184019**	Protein of unknown function	83	17920..18168	59_GENE_22
11184tRNA		**CGL2_11184R001**	TRNA Ser GGA		18280..18372	
**11184**	**20**	**CGL2_11184020**	DNA polymerase III subunit gamma/tau (EC 2.7.7.7)	540	18561..20180	59_GENE_21
**11184**	**21**	**CGL2_11184021**	Protein of unknown function	104	20180..20491	59_GENE_20
**11184**	**22**	**CGL2_11184022**	Recombination protein RecR	214	20507..21148	59_GENE_19
**11184**	**23**	**CGL2_11184023**	Peptide chain release factor 3	551	21206..22858	59_GENE_18
**11184**	**24**	**CGL2_11184024**	Putative mechanosensitive ion channel	313	complement(22805..23743)	59_GENE_17
11184tRNA		**CGL2_11184R002**	TRNA Ser GCT		complement(23816..23908)	
11184tRNA		**CGL2_11184R003**	TRNA Ser TGA		complement(23939..24024)	
**11184**	**25**	**CGL2_11184025**	Putative TPR-domain containing protein	272	complement(24581..25396)	59_GENE_16
**11184**	**26**	**CGL2_11184026**	Putative zinc-binding cytidine/deoxycytidylate deami	162	25430..25915	59_GENE_15
11184tRNA		**CGL2_11184R004**	TRNA Ser CGA		25991..26080	
**11184**	**27**	**CGL2_11184027**	Putative sigma-54 specific transcriptional regulator, I	453	26104..27462	59_GENE_14
**11184**	**28**	**CGL2_11184028**	Protein of unknown function	45	27707..27841	59_GENE_13
**11184**	**29**	**CGL2_11184029**	MazG family protein (nucleotide pyrophosphohydrola	301	27947..28849	59_GENE_12
**11184**	**30**	**CGL2_11184030**	Probable glycosyl transferase family protein	334	28849..29850	59_GENE_11
**11184**	**31**	**CGL2_11184031**	Probable metal-dependent phosphohydrolase	403	29865..31073	59_GENE_10
**11184**	**32**	**CGL2_11184032**	Protein of unknown function	80	complement(31072..31311)	59_GENE_9
**11184**	**33**	**CGL2_11184033**	Protein of unknown function	136	complement(31376..31783)	59_GENE_8
**11184**	**34**	**CGL2_11184034**	Hypothetical protein	72	31989..32204	59_GENE_7

**11184**	**35**	**CGL2_11184035**	Probable acid phosphatase	329	complement(32217..33203)	59_GENE_6
**11184**	**36**	**CGL2_11184036**	Protein of unknown function	464	complement(33203..34594)	59_GENE_5
**11184**	**37**	**CGL2_11184037**	Dihydrolipoamide dehydrogenase (EC 1.8.1.4)	462	complement(34642..36027)	59_GENE_4
11184tRNA		**CGL2_11184R005**	TRNA Met CAT		36112..36188	
**11184**	**38**	**CGL2_11184038**	transposase	479	complement(37465..38901)	333_GENE_1
**11184**	**39**	**CGL2_11184039**	Hypothetical protein	554	complement(38909..40570)	59_GENE_3
**11184**	**40**	**CGL2_11184040**	Hypothetical protein	213	complement(40761..41399)	333_GENE_4
**11184**	**41**	**CGL2_11184041**	Probable transcriptional regulator, LuxR family	358	42029..43102	333_GENE_5
**11184**	**42**	**CGL2_11184042**	Putative uracil-DNA glycosylase	237	complement(43132..43842)	333_GENE_6
**11184**	**43**	**CGL2_11184043**	Hypothetical protein	66	complement(43833..44030)	333_GENE_7
**11184**	**44**	**CGL2_11184044**	Protein of unknown function	404	complement(44030..45241)	333_GENE_8
**11184**	**45**	**CGL2_11184045**	Protein of unknown function (membrane?)	124	45372..45743	333_GENE_9
**11184**	**46**	**CGL2_11184046**	Exoribonuclease II (EC 3.1.13.1)	744	complement(45739..47970)	3_GENE_1
**11184**	**47**	**CGL2_11184047**	Protein of unknown function	160	48741..49220	3_GENE_4
**11184**	**48**	**CGL2_11184048**	Putative outer membrane protein, OmpA/MotB famil	179	49362..49898	3_GENE_5
**11184**	**49**	**CGL2_11184049**	Probable OmpA family protein	232	50257..50952	3_GENE_6
**11184**	**50**	**CGL2_11184050**	Hypothetical protein	161	complement(50891..51373)	3_GENE_7
**11184**	**51**	**CGL2_11184051**	Thiamine-monophosphate kinase (EC 2.7.4.16)	335	complement(51360..52364)	3_GENE_8
**11184**	**52**	**CGL2_11184052**	ATP-dependent protease La (EC 3.4.21.53)	816	complement(52357..54804)	3_GENE_9
**11184**	**53**	**CGL2_11184053**	UTP-glucose-1-phosphate uridylyltransferase (GalU)	297	complement(54811..55701)	3_GENE_10
**11184**	**54**	**CGL2_11184054**	Dephospho-CoA kinase (EC 2.7.1.24)	204	complement(55723..56334)	3_GENE_11
**11184**	**55**	**CGL2_11184055**	Hypothetical protein	159	complement(56334..56810)	3_GENE_12
**11184**	**56**	**CGL2_11184056**	Protein of unknown function	229	complement(56810..57496)	3_GENE_13
**11184**	**57**	**CGL2_11184057**	DNA polymerase I (EC 2.7.7.7)	872	complement(57530..60145)	3_GENE_14
**11184**	**58**	**CGL2_11184058**	Branched-chain amino acid aminotransferase (EC 2.6	310	complement(60155..61084)	3_GENE_15
**11184**	**59**	**CGL2_11184059**	Probable membrane protein	234	complement(61126..61827)	3_GENE_16
**11184**	**60**	**CGL2_11184060**	Hypothetical protein	191	complement(61920..62492)	3_GENE_17
**11184**	**61**	**CGL2_11184061**	Hypothetical protein	333	complement(62483..63481)	3_GENE_18
**11184**	**62**	**CGL2_11184062**	Putative transcriptional regulator, LysR family	303	complement(63481..64389)	3_GENE_19
**11184**	**63**	**CGL2_11184063**	Probable ATP-dependent DNA helicase	737	complement(64411..66621)	3_GENE_20
**11184**	**64**	**CGL2_11184064**	Conserved hypothetical protein	240	complement(66625..67344)	3_GENE_21
**11184**	**65**	**CGL2_11184065**	Protein of unknown function	468	complement(67319..68722)	3_GENE_22
**11184**	**66**	**CGL2_11184066**	Conserved protein of unknown function	185	complement(68725..69279)	3_GENE_23
**11184**	**67**	**CGL2_11184067**	Nicotinate-nucleotide adenylyltransferase (EC 2.7.7.1	230	complement(69263..69952)	3_GENE_25
**11184**	**68**	**CGL2_11184068**	Gamma-glutamyl phosphate reductase (EC 1.2.1.41)	421	complement(69974..71236)	3_GENE_26
**11184**	**69**	**CGL2_11184069**	Glutamate 5-kinase (EC 2.7.2.11)	380	complement(71246..72385)	3_GENE_27
**11184**	**70**	**CGL2_11184070**	Putative GTP binding protein	360	complement(72385..73464)	3_GENE_28
**11184**	**71**	**CGL2_11184071**	Ribosomal protein L27	92	complement(73492..73767)	3_GENE_29
**11184**	**72**	**CGL2_11184072**	Ribosomal protein L21	106	complement(73849..74166)	3_GENE_30
**11184**	**73**	**CGL2_11184073**	Putative ferric uptake regulator, Fur family	153	74477..74935	3_GENE_31
**11184**	**74**	**CGL2_11184074**	Probable glycosyl hydrolase, BNR repeat	352	complement(74979..76034)	3_GENE_32

**11184**	**75**	**CGL2_11184075**	transposase	115	complement(76434..76778)	3_GENE_33
**11181**	**1**	**CGL2_11181001**	Protein of unknown function	332	complement(801..1796)	3_GENE_34
**11181**	**2**	**CGL2_11181002**	Probable 6-pyruvoyl tetrahydrobiopterin synthase fa	128	2118..2501	3_GENE_36
**11181**	**3**	**CGL2_11181003**	Oligopeptide/dipeptide ABC transporter, ATP-binding	336	2501..3508	3_GENE_37
**11181**	**4**	**CGL2_11181004**	ABC phosphate transporter (EC 3.6.3.27)	347	3513..4553	3_GENE_38
**11181**	**5**	**CGL2_11181005**	Phenylalanyl-tRNA synthetase alpha chain (EC 6.1.1.	519	4581..6137	3_GENE_39
**11181**	**6**	**CGL2_11181006**	Phenylalanyl-tRNA synthetase beta chain	569	6174..7880	3_GENE_40
**11181**	**7**	**CGL2_11181007**	Ribosomal protein L20	118	complement(7952..8305)	3_GENE_41
**11181**	**8**	**CGL2_11181008**	Ribosomal protein L35	65	complement(8390..8584)	3_GENE_42
**11181**	**9**	**CGL2_11181009**	Translation initiation factor 3 (IF-3)	175	complement(8629..9153)	3_GENE_43
**11181**	**10**	**CGL2_11181010**	Threonyl-tRNA synthetase (EC 6.1.1.3)	617	complement(9229..11079)	3_GENE_44
**11181**	**10a**	**CGL2_11181010a**	Putative transpoase	446	complement(11438..12775)	
**11181**	**11**	**CGL2_11181011**	Putative diguanylate cyclase/phosphodiesterase	1196	complement(12923..16510)	3_GENE_45
**11181**	**12**	**CGL2_11181012**	Phosphomethylpyrimidine kinase (EC 2.7.4.7)	283	complement(16529..17377)	3_GENE_46
**11181**	**13**	**CGL2_11181013**	Putative peptidase M23B family protein	296	complement(17383..18270)	3_GENE_47
**11181**	**14**	**CGL2_11181014**	Thiamine monophosphate synthase (EC 2.5.1.3)	215	complement(18270..18914)	3_GENE_48
**11181**	**15**	**CGL2_11181015**	Thiazole biosynthesis protein (ThiG)	274	complement(18920..19741)	3_GENE_49
**11181**	**17**	**CGL2_11181017**	Acetyl-CoA synthetase (EC 6.2.1.1)	641	19983..21905	3_GENE_50
**11181**	**18**	**CGL2_11181018**	Putative glyoxalase (GloA)	291	21932..22804	3_GENE_51
**11181**	**19**	**CGL2_11181019**	Hypothetical protein	270	complement(22776..23585)	3_GENE_52
**11181**	**20**	**CGL2_11181020**	Putative monogalactosyldiacylglycerol synthase	376	23777..24904	3_GENE_53
**11181**	**21**	**CGL2_11181021**	Putative metallophosphoesterase	311	24904..25836	3_GENE_54
**11181**	**22**	**CGL2_11181022**	Probable major facilitator superfamily transporter	405	complement(25751..26965)	3_GENE_55
**11181**	**23**	**CGL2_11181023**	Carboxymethylenebutenolidase (EC 3.1.1.45)	259	complement(27021..27797)	3_GENE_56
**11181**	**24**	**CGL2_11181024**	Putative outer membrane protein	496	complement(28208..29695)	3_GENE_57
**11181**	**25**	**CGL2_11181025**	Protein of unknown function	165	complement(29796..30290)	3_GENE_58
**11181**	**26**	**CGL2_11181026**	Putative periplasmic sensor signal transduction histid	474	complement(30414..31835)	3_GENE_59
**11181**	**27**	**CGL2_11181027**	Putative two component transcriptional regulator, wi	222	complement(31850..32515)	3_GENE_60
**11181**	**28**	**CGL2_11181028**	Protein of unknown function	151	complement(32571..33023)	3_GENE_61
11181tRNA		**CGL2_11181R001**	TRNA Pro TGG		complement(33263..33339)	
**11181**	**29**	**CGL2_11181029**	Protein of unknown function	318	33507..34460	3_GENE_62
**11181**	**30**	**CGL2_11181030**	Putative beta-lactamase family protein	293	34468..35346	3_GENE_63
**11181**	**31**	**CGL2_11181031**	ATP synthase F1, epsilon subunit	143	complement(35424..35852)	3_GENE_64
**11181**	**32**	**CGL2_11181032**	ATP synthase F1, beta subunit (EC 3.6.3.15)	463	complement(35861..37249)	3_GENE_65
**11181**	**33**	**CGL2_11181033**	ATP synthase F1, gamma subunit (EC 3.6.3.15)	291	complement(37297..38169)	3_GENE_66
**11181**	**34**	**CGL2_11181034**	ATP synthase F1, alpha subunit (EC 3.6.3.15)	512	complement(38176..39711)	3_GENE_67
**11181**	**35**	**CGL2_11181035**	Putative ATP synthase F1, delta subunit	189	complement(39711..40277)	3_GENE_68
**11181**	**36**	**CGL2_11181036**	ATP synthase F0, subunit B	175	complement(40277..40801)	3_GENE_69
**11181**	**37**	**CGL2_11181037**	ATP synthase F0, subunit C	76	complement(40850..41077)	3_GENE_70
**11181**	**38**	**CGL2_11181038**	ATP synthase F0, subunit A	244	complement(41111..41842)	3_GENE_71
**11181**	**39**	**CGL2_11181039**	Hypothetical protein	89	complement(41849..42115)	3_GENE_72

**11181**	**40**	**CGL2_11181040**	Probable membrane protein	164	42457..42948	3_GENE_73
**11181**	**41**	**CGL2_11181041**	Phosphoglycolate phosphatase (EC 3.1.3.18)	239	complement(42869..43585)	3_GENE_74
**11181**	**42**	**CGL2_11181042**	Putative oligopeptide ABC transporter, permease pro	340	complement(43651..44670)	3_GENE_75
**11181**	**43**	**CGL2_11181043**	Putative oligopeptide ABC transporter, permease pro	324	complement(44670..45641)	3_GENE_76
**11181**	**44**	**CGL2_11181044**	Transporter (Extracellular solute binding protein fam	534	complement(45641..47242)	3_GENE_77
**11181**	**45**	**CGL2_11181045**	preprotein translocase, SecG subunit	101	complement(47380..47682)	3_GENE_78
**11181**	**46**	**CGL2_11181046**	Triosephosphate isomerase (EC 5.3.1.1)	258	complement(47737..48510)	3_GENE_79
**11181**	**47**	**CGL2_11181047**	Phosphoglycerate kinase (EC 2.7.2.3)	399	complement(48527..49723)	3_GENE_80
**11181**	**48**	**CGL2_11181048**	Glyceraldehyde-3-phosphate dehydrogenase (EC 1.2	342	complement(49723..50748)	3_GENE_81
**11181**	**49**	**CGL2_11181049**	Peptidoglycan glycosyltransferase (EC 2.4.1.129)	790	complement(50867..53236)	3_GENE_82
**11181**	**50**	**CGL2_11181050**	Hypothetical protein	70	complement(53463..53672)	3_GENE_84
**11181**	**51**	**CGL2_11181051**	Orotate phosphoribosyl transferase (EC 2.4.2.10)	212	complement(53820..54455)	3_GENE_85
**11181**	**52**	**CGL2_11181052**	Putative methyl-accepting chemotaxis sensory transd	368	complement(54660..55763)	3_GENE_86
11181tRNA		**CGL2_11181R002**	TRNA Leu GAG		complement(56130..56213)	
**11181**	**53**	**CGL2_11181053**	Siroheme synthase, N-terminal	217	complement(56502..57152)	3_GENE_89
**11181**	**54**	**CGL2_11181054**	Probable DNA uptake lipoprotein	243	complement(57152..57880)	3_GENE_90
**11181**	**55**	**CGL2_11181055**	3-oxoacyl-[acyl-carrier-protein] synthase 3 (EC 2.3.1	329	57974..58960	3_GENE_91
**11181**	**56**	**CGL2_11181056**	Putative two component, sigma54 specific, transcript	457	complement(58963..60333)	3_GENE_92
**11181**	**57**	**CGL2_11181057**	Multi-sensor signal transduction histidine kinase	751	complement(60337..62589)	3_GENE_93
**11181**	**58**	**CGL2_11181058**	Protein of unknown function	408	complement(62648..63871)	3_GENE_94
**11181**	**59**	**CGL2_11181059**	Protein of unknown function	222	complement(63878..64543)	3_GENE_95
**11181**	**60**	**CGL2_11181060**	Probable multiple antibiotic resistance protein (MarC)	201	64582..65184	3_GENE_96
**11181**	**61**	**CGL2_11181061**	Protein of unknown function	144	65289..65720	3_GENE_97
**11181**	**62**	**CGL2_11181062**	Putative UbiE/COQ5 methyltransferase	279	complement(65732..66568)	3_GENE_98
**11181**	**63**	**CGL2_11181063**	Putative queuosine biosynthesis protein	385	complement(66568..67722)	3_GENE_99
**11181**	**64**	**CGL2_11181064**	Putative diguanylate phosphodiesterase	347	67758..68798	3_GENE_100
**11181**	**65**	**CGL2_11181065**	Protein of unknown function	168	68937..69440	3_GENE_101
**11181**	**66**	**CGL2_11181066**	Probable lipoprotein	396	69456..70643	3_GENE_102
**11181**	**67**	**CGL2_11181067**	Putative sulfide-quinone reductase	375	complement(70746..71870)	3_GENE_103
**11181**	**68**	**CGL2_11181068**	Carbonic anhydrase	193	complement(71985..72563)	147_GENE_15
**11181**	**69**	**CGL2_11181069**	Diguanylate cyclase	368	72947..74050	147_GENE_14
**11181**	**70**	**CGL2_11181070**	Protein of unknown function	161	74106..74588	147_GENE_13
**11181**	**71**	**CGL2_11181071**	Probable helicase	62	74581..74766	147_GENE_11a
**11181**	**72**	**CGL2_11181072**	Putative helicase	1055	complement(74776..77940)	147_GENE_11
**11181**	**73**	**CGL2_11181073**	Putative NUDIX hydrolase	134	complement(77930..78331)	147_GENE_10
**11181**	**74**	**CGL2_11181074**	Putative metal-dependent phosphohydrolase	476	78515..79942	147_GENE_9
**11181**	**75**	**CGL2_11181075**	Patatin-like phospholipase	330	79948..80937	147_GENE_8
**11181**	**76**	**CGL2_11181076**	Alcohol dehydrogenase (EC 1.1.1.1)	341	complement(80947..81969)	147_GENE_7
**11181**	**77**	**CGL2_11181077**	Probable anti-ECFsigma factor (ChrR)	223	complement(81969..82637)	147_GENE_6
**11181**	**78**	**CGL2_11181078**	Protein of unknown function	268	82811..83614	147_GENE_5
**11181**	**79**	**CGL2_11181079**	Conserved hypothetical protein	318	83614..84567	147_GENE_4

**11181**	**80**	**CGL2_11181080**	Protein of unknown function	100	84741..85040	147_GENE_3
**11181**	**81**	**CGL2_11181081**	Probable transcriptional regulator	97	85040..85330	147_GENE_2
		**CGL2_11181CRISPR**			85518..89905	
		**CGL2_11386CRISPR**			36056..37265	
**11386**	**30**	**CGL2_11386030**	CRISPR-ssociated protein, Cas2	102	35660..35965	26_GENE_2
**11386**	**29**	**CGL2_11386029**	CRISPR-ssociated protein, Cas1	306	34759..35676	26_GENE_3
**11386**	**28**	**CGL2_11386028**	CRISPR-ssociated protein, Cas3	218	34110..34763	26_GENE_4
**11386**	**27**	**CGL2_11386027**	CRISPR-ssociated protein, Cas5	227	33440..34120	26_GENE_5
**11386**	**26**	**CGL2_11386026**	CRISPR-ssociated protein, Cas4	398	32239..33432	26_GENE_6
**11386**	**25**	**CGL2_11386025**	CRISPR-ssociated protein, Cas2	171	31727..32239	26_GENE_7
**11386**	**24**	**CGL2_11386024**	CRISPR-ssociated protein, Cas1	508	30204..31727	26_GENE_8
**11386**	**23**	**CGL2_11386023**	CRISPR-associated helicase Cas3	880	27565..30204	26_GENE_9
**11386**	**22**	**CGL2_11386022**	Transposase	455	25968..27332	26_GENE_10
**11386**	**21**	**CGL2_11386021**	Probable transposase	143	complement(25114..25542)	26_GENE_12
**11386**	**20**	**CGL2_11386020**	Hypothetical protein	74	complement(24765..24986)	26_GENE_13
**11386**	**19**	**CGL2_11386019**	Hypothetical protein	43	complement(24580..24708)	26_GENE_14
**11386**	**18**	**CGL2_11386018**	DNA methyltransferase/helicase	1067	complement(20914..24114)	26_GENE_15
**11386**	**17**	**CGL2_11386017**	Conserved protein of unknown function	209	complement(20254..20880)	26_GENE_16
**11386**	**16**	**CGL2_11386016**	Protein of unknown function	229	19380..20066	26_GENE_17
**11386**	**15**	**CGL2_11386015**	Hypothetical protein	125	18864..19238	26_GENE_18
**11386**	**14**	**CGL2_11386014**	Conserved hypothetical protein	703	16756..18864	26_GENE_19
		Strain variant path	Strain variant path			Strain variant path
**11386**	**13**	**CGL2_11386013**	Conserved hypothetical protein	300	15833..16732	26_GENE_20
**11386**	**12**	**CGL2_11386012**	phage DNA binding protein	60	15654..15833	26_GENE_21
**11386**	**11**	**CGL2_11386011**	Hypothetical protein	309	14611..15537	26_GENE_22
**11386**	**10**	**CGL2_11386010**	integrase INT P4	403	13403..14611	26_GENE_23
**11386**	**9**	**CGL2_11386009**	SsrA-binding protein	163	12311..12799	26_GENE_24
**11386**	**8**	**CGL2_11386008**	Leucyl aminopeptidase (EC 3.4.11.1)	532	10649..12244	183_GENE_9
**11386**	**7**	**CGL2_11386007**	ExsB protein (succinoglycan synthase?)	241	9812..10534	183_GENE_8
**11386**	**6**	**CGL2_11386006**	Putative radical activating enzyme	212	9171..9806	183_GENE_7
**11386**	**5**	**CGL2_11386005**	Protein of unknown function	231	8411..9103	183_GENE_6
**11386**	**4**	**CGL2_11386004**	DNA gyrase, A subunit	816	5860..8307	183_GENE_5
**11386**	**3**	**CGL2_11386003**	DNA gyrase, B subunit (EC 5.99.1.3)	818	3348..5801	183_GENE_4
**11386**	**2**	**CGL2_11386002**	DNA polymerase III, beta chain (EC 2.7.7.7)	387	1959..3119	183_GENE_3
**11386**	**1**	**CGL2_11386001**	Chromosomal replication initiator protein (DnaA) ORI	465	543..1937	183_GENE_2
		Strain variant path	Strain variant path			Strain variant path
**11389**	**40**	**CGL2_11389040**	Acetolactate synthase, large subunit (EC 2.2.1.6)	581	complement(34161..35903)	26_GENE_33
**11389**	**39**	**CGL2_11389039**	Acetolactate synthase, small subunit	184	complement(33592..34143)	26_GENE_34
**11389**	**38**	**CGL2_11389038**	Acetohydroxy acid isomeroreductase (EC 1.1.1.86)	339	complement(32507..33523)	26_GENE_35
**11389**	**37**	**CGL2_11389037**	Phosphatidylserine decarboxylase (EC 4.1.1.65)	217	complement(31841..32491)	26_GENE_36
**11389**	**36**	**CGL2_11389036**	CDP-diacylglycerol-serine O-phosphatidyltransferase	278	complement(31036..31869)	26_GENE_37

**11389**	**35**	**CGL2_11389035**	2-isopropylmalate synthase (EC 2.3.3.13) AA and py	532	complement(29395..30990)	26_GENE_38
**11389**	**34**	**CGL2_11389034**	3-isopropylmalate dehydrogenase (EC 1.1.1.85) AA I	370	complement(28228..29337)	26_GENE_39
**11389**	**33**	**CGL2_11389033**	Aspartate-semialdehyde dehydrogenase (EC 1.2.1.1:	349	complement(27182..28228)	26_GENE_40
**11389**	**32**	**CGL2_11389032**	Quinolinate synthetase complex, subunit A	316	complement(26215..27162)	26_GENE_41
**11389**	**31**	**CGL2_11389031**	Conserved hypothetical protein	79	complement(25979..26215)	26_GENE_42
**11389**	**30**	**CGL2_11389030**	Protein of unknown function	170	25497..26006	26_GENE_43
**11389**	**29**	**CGL2_11389029**	Putative ferredoxin oxidoreductase	193	24828..25406	26_GENE_44
**11389**	**28**	**CGL2_11389028**	Putative glyoxalase/bleomycin resistance protein	141	complement(24161..24583)	26_GENE_45
**11389**	**27**	**CGL2_11389027**	Protein of unknown function	351	23091..24143	26_GENE_46
**11389**	**26**	**CGL2_11389026**	Conserved hypothetical protein	375	21909..23033	26_GENE_47
**11389**	**25**	**CGL2_11389025**	Conserved hypothetical protein	191	21350..21922	26_GENE_48
**11389**	**24**	**CGL2_11389024**	Conserved protein of unknown function	190	20605..21174	80_GENE_26
**11389**	**23**	**CGL2_11389023**	Cobalamin B12-binding/Radical SAM family protein	517	19008..20558	80_GENE_25
**11389**	**22**	**CGL2_11389022**	Ribulose-bisphosphate carboxylase (EC 4.1.1.39)	392	complement(17625..18800)	80_GENE_24
**11389**	**21**	**CGL2_11389021**	Protein of unknown function	283	16774..17622	80_GENE_23
**11389**	**20**	**CGL2_11389020**	Periplasmic phosphate binding protein	364	15661..16752	80_GENE_22
**11389**	**19**	**CGL2_11389019**	Protein of unknown function	321	14642..15604	80_GENE_21
**11389**	**18**	**CGL2_11389018**	Probable 2'-5' RNA ligase	204	14041..14652	80_GENE_20
**11389**	**17**	**CGL2_11389017**	Protein of unknown function	348	complement(12885..13928)	80_GENE_19
**11389**	**16**	**CGL2_11389016**	Protein of unknown function	138	complement(12452..12865)	80_GENE_18
**11389**	**15**	**CGL2_11389015**	Putative ribosomal protein L11 methyltransferase	291	complement(11580..12452)	80_GENE_17
**11389**	**14**	**CGL2_11389014**	Orotidine 5'-phosphate decarboxylase (EC 4.1.1.23)	251	complement(10828..11580)	80_GENE_16
**11389**	**13**	**CGL2_11389013**	Peptide deformylase (EC 3.5.1.88)	184	10274..10825	80_GENE_15
**11389**	**12**	**CGL2_11389012**	Cob(I)alamin adenosyltransferase (EC 2.5.1.17)	179	9735..10271	80_GENE_14
**11389**	**11**	**CGL2_11389011**	Precorrin-3B methylase (EC 2.1.1.131)	273	8917..9735	80_GENE_13
**11389**	**10**	**CGL2_11389010**	Putative cobalamin biosynthesis protein (CbiG)	378	7740..8873	80_GENE_12
**11389**	**9**	**CGL2_11389009**	Precorrin-6B methyltransferase	205	7126..7740	80_GENE_11
**11389**	**8**	**CGL2_11389008**	Precorrin 6y methyltransferase	210	6466..7095	80_GENE_10
**11389**	**7**	**CGL2_11389007**	Cobalamin biosynthesis protein (CbiD)	375	5313..6437	80_GENE_9
**11389**	**6**	**CGL2_11389006**	Conserved hypothetical protein	176	4676..5203	80_GENE_8
**11389**	**5**	**CGL2_11389005**	Large-conductance mechanosensitive channel	151	complement(3535..3987)	80_GENE_7
**11389**	**4**	**CGL2_11389004**	transposase	122	2331..2696	80_GENE_6
**11389**	**3**	**CGL2_11389003**	transposase	79	2107..2343	80_GENE_5
**11389**	**2**	**CGL2_11389002**	transposase	200	1474..2073	80_GENE_4
**11389**	**1**	**CGL2_11389001**	transposase	334	complement(405..1406)	80_GENE_3
**10706**	**8**	**CGL2_10706008**	Hypothetical protein	264	complement(10384..11175)	80_GENE_1
**10706**	**7**	**CGL2_10706007**	Hypothetical protein	454	8466..9827	
**10706**	**6**	**CGL2_10706006**	DNA helicase	1573	complement(3543..8261)	214_GENE_4
**10706**	**5**	**CGL2_10706005**	transposase	78	3207..3440	214_GENE_5
**10706**	**4**	**CGL2_10706004**	integrase fragment or transposase (Tn domain)	218	2594..3247	214_GENE_6
**10706**	**3**	**CGL2_10706003**	transposase?	49	complement(1994..2140)	214_GENE_7

**10706**	**2**	**CGL2_10706002**	Hypothetical protein	81	complement(1752..1994)	214_GENE_8
**10706**	**1**	**CGL2_10706001**	Hypothetical protein	507	complement(114..1634)	
**11172**	**1**	**CGL2_11172001**	Ribosomal protein L9	150	873..1322	150_GENE_15
**11172**	**2**	**CGL2_11172002**	Ribosomal protein L34	44	1415..1546	150_GENE_15a
**11172**	**3**	**CGL2_11172003**	Protein of unknown function	111	1567..1899	150_GENE_14a
**11172**	**4**	**CGL2_11172004**	Protein of unknown function	68	1878..2081	150_GENE_13a
**11172**	**5**	**CGL2_11172005**	Putative inner-membrane protein	511	2128..3660	150_GENE_13
**11172**	**6**	**CGL2_11172006**	TRNA modification GTPase TrmE	445	3677..5011	150_GENE_12
**11172**	**7**	**CGL2_11172007**	Glucose-inhibited division protein A	605	5030..6844	150_GENE_11
**11172**	**8**	**CGL2_11172008**	Putative glucose-inhibited division protein B	210	6852..7481	150_GENE_10
**11172**	**9**	**CGL2_11172009**	Putative cobyrinic acid a,c-diamide synthase (CbiA)	254	7532..8293	150_GENE_9
**11172**	**10**	**CGL2_11172010**	ParB-like partition protein	284	8289..9140	150_GENE_8
**11172**	**11**	**CGL2_11172011**	Protein of unknown function	132	9157..9552	150_GENE_7
**11172**	**12**	**CGL2_11172012**	tRNA (5-methylaminomethyl-2-thiouridylate)-methyl	361	9570..10652	150_GENE_6
**11172**	**13**	**CGL2_11172013**	DNA polymerase III, epsilon subunit	265	10652..11446	150_GENE_5
**11172**	**14**	**CGL2_11172014**	Ribonuclease III (EC 3.1.26.3)	375	11930..13054	150_GENE_4
**11172**	**15**	**CGL2_11172015**	Transposase	479	13375..14811	150_GENE_1
**11172**	**16**	**CGL2_11172016**	Protein of unknown function	77	15149..15379	
**11172**	**17**	**CGL2_11172017**	Ferredoxin	109	complement(15419..15745)	
**11172**	**18**	**CGL2_11172018**	Protein of unknown function	148	15949..16392	6_GENE_88a
**11172**	**19**	**CGL2_11172019**	Hypothetical protein	161	16544..17026	6_GENE_88
**11172**	**20**	**CGL2_11172020**	UDP-3-O-acyl-N-acetylglucosamine deacetylase	298	complement(17057..17950)	6_GENE_87
**11172**	**21**	**CGL2_11172021**	Hypothetical protein	101	complement(18201..18503)	6_GENE_85
**11172**	**22**	**CGL2_11172022**	Protein of unknown function	520	complement(18632..20191)	6_GENE_84
**11172**	**23**	**CGL2_11172023**	Hypothetical protein	180	complement(20252..20791)	6_GENE_83
**11172**	**24**	**CGL2_11172024**	Inositol monophosphatase (EC 3.1.3.25)	265	21032..21826	6_GENE_82
**11172**	**25**	**CGL2_11172025**	Putative peptidase M50 family	264	complement(21819..22610)	6_GENE_81
**11172**	**26**	**CGL2_11172026**	Hypothetical protein	106	complement(22673..22990)	6_GENE_80
**11172**	**27**	**CGL2_11172027**	Transposase	446	complement(23400..24737)	
**11172**	**28**	**CGL2_11172028**	Trans-hexaprenyltranstransferase (EC 2.5.1.30)	330	24939..25928	6_GENE_79
**11172**	**29**	**CGL2_11172029**	Protein of unknown function	426	25980..27257	6_GENE_78
**11172**	**31**	**CGL2_11172031**	Pseudouridylate synthases, 23S RNA-specific	340	complement(27247..28266)	6_GENE_77
**11172**	**32**	**CGL2_11172032**	Putative TPR-domain containing protein	327	complement(28279..29259)	6_GENE_76
**11172**	**33**	**CGL2_11172033**	Signal peptidase II (EC 3.4.23.36)	179	complement(29262..29798)	6_GENE_75
**11172**	**34**	**CGL2_11172034**	Isoleucyl-tRNA synthetase (EC 6.1.1.5)	976	complement(29798..32725)	6_GENE_74
11172tRNA		**CGL2_11172R001**	TRNA Val CAC		32816..32890	
**11172**	**35**	**CGL2_11172035**	Transposase	396	complement(33467..34654)	
**11172**	**36**	**CGL2_11172036**	Hypothetical protein	88	35171..35434	6_GENE_73
**11172**	**37**	**CGL2_11172037**	Transposase	416	35505..36752	
**11172**	**38**	**CGL2_11172038**	Conserved hypothetical protein	192	36808..37383	
**11172**	**39**	**CGL2_11172039**	Hypothetical protein	96	37465..37752	6_GENE_73

**11172**	**40**	**CGL2_11172040**	Formamidopyrimidine-DNA glycosylase (EC 3.2.2.23)	291	complement(37655..38527)	6_GENE_72a
**11172**	**41**	**CGL2_11172041**	Conserved hypothetical protein	343	complement(38650..39678)	48_GENE_37
**11172**	**42**	**CGL2_11172042**	Putative aminoglycoside phosphotransferase	355	39815..40879	48_GENE_36
**11172**	**43**	**CGL2_11172043**	Nucleoside-diphosphate-sugar pyrophosphorylase inv	240	40914..41633	48_GENE_35
**11172**	**44**	**CGL2_11172044**	Protein of unknown function	384	complement(41479..42630)	48_GENE_34
**11172**	**45**	**CGL2_11172045**	GTP-binding protein	212	42767..43402	48_GENE_33
**11172**	**46**	**CGL2_11172046**	Putative peptidyl-prolyl cis-trans isomerase	340	complement(43411..44430)	48_GENE_32
**11172**	**47**	**CGL2_11172047**	Putative peptidyl-prolyl cis-trans isomerase	324	complement(44470..45441)	48_GENE_31
**11172**	**48**	**CGL2_11172048**	Probable peptidil-prolyl cis-trans isomerase	289	complement(45451..46317)	48_GENE_30
**11172**	**49**	**CGL2_11172049**	Transcription-repair coupling factor	1154	complement(46332..49793)	48_GENE_29
**11172**	**50**	**CGL2_11172050**	Cytidylyltransferase family protein	179	complement(49793..50329)	48_GENE_28
**11172**	**51**	**CGL2_11172051**	Phosphoheptose isomerase	203	complement(50322..50930)	48_GENE_27
**11172**	**52**	**CGL2_11172052**	Glutamate-1-semialdehyde 2,1-aminomutase (EC 5.4	437	complement(50991..52301)	48_GENE_26
**11172**	**53**	**CGL2_11172053**	Protein of unknown function	70	52765..52974	48_GENE_24
**11172**	**54**	**CGL2_11172054**	Protein of unknown function	195	53079..53663	48_GENE_23
**11172**	**55**	**CGL2_11172055**	Pyruvate synthase alpha subunit (EC 1.2.7.1)	412	53966..55201	48_GENE_21
**11172**	**56**	**CGL2_11172056**	Pyruvate synthase beta subunit	288	55283..56146	48_GENE_20
**11172**	**57**	**CGL2_11172057**	Putative ferredoxin oxidoreductase gamma subunit	232	56192..56887	48_GENE_19
**11172**	**58**	**CGL2_11172058**	Probable ferredoxin oxidoreductase epsilon subunit	101	56918..57220	48_GENE_18
**11172**	**59**	**CGL2_11172059**	Protein of unknown function	94	57305..57586	48_GENE_17
**11172**	**60**	**CGL2_11172060**	Protein of unknown function	140	57595..58014	48_GENE_16a
**11172**	**61**	**CGL2_11172061**	Pyruvate synthase alpha chain (EC 1.2.7.1)	406	58212..59429	48_GENE_16
**11172**	**62**	**CGL2_11172062**	Putative ferredoxin oxidoreductase beta subunit	296	59425..60312	48_GENE_15
**11172**	**63**	**CGL2_11172063**	Ferredoxin oxidoreductase gamma subunit	232	60338..61033	48_GENE_13
**11172**	**64**	**CGL2_11172064**	Protein of unknown function	76	61285..61512	48_GENE_12
**11172**	**65**	**CGL2_11172065**	Probable ferredoxin oxidoreductase	193	61593..62171	48_GENE_11
**11172**	**66**	**CGL2_11172066**	Protein of unknown function	133	complement(62234..62632)	48_GENE_10
**11172**	**67**	**CGL2_11172067**	Conserved hypothetical protein	175	complement(62787..63311)	48_GENE_9
**11172**	**68**	**CGL2_11172068**	Glutamyl-tRNA synthetase (EC 6.1.1.17)	474	complement(63311..64732)	48_GENE_8
**11172**	**69**	**CGL2_11172069**	Protein of unknown function	181	65060..65602	48_GENE_7
**11172**	**70**	**CGL2_11172070**	L-aspartate oxidase (EC 1.4.3.16)	513	65610..67148	48_GENE_6
**11172**	**71**	**CGL2_11172071**	Putative heat shock protein DnaJ, N-terminal	284	67148..67999	48_GENE_5
**11172**	**72**	**CGL2_11172072**	Probable heat shock transcription regulator	108	68017..68340	48_GENE_4
**11172**	**73**	**CGL2_11172073**	Chaperone clpB	866	68430..71027	48_GENE_3
**11172**	**74**	**CGL2_11172074**	Probable bacterial regulatory protein, Fis family	178	71027..71560	48_GENE_2
**11172**	**75**	**CGL2_11172075**	Transposase	446	71731..73068	
07502tRNA		CGL2_7502R001	TRNA Val GAC		64..138	
**11111**	**114**	**CGL2_11111114**	Putative heavy metal efflux pump, CzcA family	1003	complement(116616..119624)	43_GENE_1
**11111**	**113**	**CGL2_11111113**	Putative secretion protein HlyD	358	complement(115543..116616)	43_GENE_2
**11111**	**112**	**CGL2_11111112**	Protein of unknown function	230	114799..115488	43_GENE_3
**11111**	**111**	**CGL2_11111111**	Putative Xaa-Pro aminopeptidase	381	113650..114792	43_GENE_4

**11111**	**110**	**CGL2_11111110**	Probable O-methyltransferase family protein	220	112988..113647	43_GENE_5
**11111**	**109**	**CGL2_11111109**	Putative nitrite reductase small subunit	102	complement(112445..112750)	43_GENE_6
**11111**	**108**	**CGL2_11111108**	Hypothetical protein	75	complement(112137..112361)	43_GENE_7
**11111**	**107**	**CGL2_11111107**	Glucose-1-phosphate adenylyltransferase (EC 2.7.7.:	419	complement(110833..112089)	43_GENE_8
**11111**	**106**	**CGL2_11111106**	Putative glycoside hydrolase, family 57	717	complement(108683..110833)	43_GENE_9
**11111**	**105**	**CGL2_11111105**	4-alpha-glucanotransferase (EC 2.4.1.25)	697	complement(106585..108675)	43_GENE_10
**11111**	**104**	**CGL2_11111104**	Galactose-1-phosphate uridylyltransferase (EC 2.7.7	334	complement(105563..106564)	43_GENE_11
**11111**	**103**	**CGL2_11111103**	Probable aldolase	460	complement(104127..105506)	43_GENE_12
**11111**	**102**	**CGL2_11111102**	Putative glycoside hydrolase, family 57	529	complement(102538..104124)	43_GENE_13
**11111**	**101**	**CGL2_11111101**	Secretion protein HlyD	363	complement(100547..101635)	43_GENE_14
**11111**	**100**	**CGL2_11111100**	Putative acriflavin resistance protein	1057	complement(97266..100436)	43_GENE_15
**11111**	**99**	**CGL2_11111099**	Putative aminomethyltransferase	334	96234..97235	43_GENE_16
**11111**	**98**	**CGL2_11111098**	Putative two component, sigma54 specific, transcript	455	complement(94731..96095)	43_GENE_17
**11111**	**97**	**CGL2_11111097**	Probable secretion protein HlyD	403	complement(93516..94724)	43_GENE_18
**11111**	**96**	**CGL2_11111096**	Putative outer membrane efflux protein	490	complement(91979..93448)	43_GENE_19
**11111**	**95**	**CGL2_11111095**	Thioredoxin reductase (EC 1.8.1.9)	334	complement(90807..91808)	43_GENE_20
**11111**	**94**	**CGL2_11111094**	Protein of unknown function	168	complement(90304..90807)	43_GENE_21
**11111**	**93**	**CGL2_11111093**	Protein of unknown function	408	complement(89081..90304)	43_GENE_22
**11111**	**92**	**CGL2_11111092**	Protein of unknown function	689	complement(86764..88830)	43_GENE_23
**11111**	**91**	**CGL2_11111091**	Putative carboxylesterase	230	complement(86072..86761)	43_GENE_24
**11111**	**90**	**CGL2_11111090**	Protein of unknown function	143	complement(85668..86096)	43_GENE_25
**11111**	**89**	**CGL2_11111089**	Polypeptide deformylase (EC 3.5.1.31)	177	complement(85138..85668)	43_GENE_26
**11111**	**88**	**CGL2_11111088**	Methionyl-tRNA formyltransferase (EC 2.1.2.9)	319	complement(84189..85145)	43_GENE_27
**11111**	**87**	**CGL2_11111087**	Putative peptidase, M48 family	295	complement(83251..84135)	43_GENE_28
**11111**	**86**	**CGL2_11111086**	Ribulose-phosphate 3-epimerase (EC 5.1.3.1)	231	complement(82559..83251)	43_GENE_29
**11111**	**85**	**CGL2_11111085**	Protein of unknown function	149	complement(81973..82419)	43_GENE_30
**11111**	**84**	**CGL2_11111084**	Putative cell shape determining protein (MreB/Mrl)	343	complement(80891..81919)	277_GENE_2
**11111**	**83**	**CGL2_11111083**	Putative rod shape-determining protein MreC	281	complement(80012..80854)	277_GENE_3
**11111**	**82**	**CGL2_11111082**	Hypothetical protein	157	complement(79521..79991)	277_GENE_4
**11111**	**81**	**CGL2_11111081**	Peptidoglycan glycosyltransferase (EC 2.4.1.129)	644	complement(77527..79458)	277_GENE_5
**11111**	**80**	**CGL2_11111080**	Putative rod shape-determining protein (RodA)	363	complement(76439..77527)	277_GENE_6
**11111**	**79**	**CGL2_11111079**	Ribonucleases G and E	526	complement(74756..76333)	277_GENE_7
**11111**	**78**	**CGL2_11111078**	Hypothetical protein	280	73889..74728	277_GENE_8
**11111**	**77**	**CGL2_11111077**	Putative dihydroneopterin aldolase	132	73494..73889	277_GENE_9
**11111**	**76**	**CGL2_11111076**	Protein of unknown function	24	72894..72966	277_GENE_10
**11111**	**75**	**CGL2_11111075**	Protein of unknown function	181	72952..73494	277_GENE_10
**11111**	**74**	**CGL2_11111074**	Putative ferredoxin	84	complement(72362..72613)	426_GENE_1
**11111**	**73**	**CGL2_11111073**	Putative cobalamin B12-binding/Radical SAM family p	626	complement(70444..72321)	426_GENE_2
**11111**	**72**	**CGL2_11111072**	Ribosomal protein L13	151	complement(69959..70411)	426_GENE_3
**11111**	**71**	**CGL2_11111071**	Ribosomal protein S9	135	complement(69562..69966)	426_GENE_4
**11111**	**70**	**CGL2_11111070**	N-acetyl-gamma-glutamyl-phosphate reductase (EC	343	complement(68451..69479)	426_GENE_5

**11111**	**69**	**CGL2_11111069**	Arginine biosynthesis protein (ArgJ) (EC 2.3.1.35)	404	complement(67201..68412)	426_GENE_6
**11111**	**68**	**CGL2_11111068**	Ribosomal protein S2	274	complement(66212..67033)	13_GENE_68
**11111**	**67**	**CGL2_11111067**	Elongation factor Ts (EF-Ts)	198	complement(65550..66143)	13_GENE_67
**11111**	**66**	**CGL2_11111066**	Uridylate kinase	236	complement(64840..65547)	13_GENE_66
**11111**	**65**	**CGL2_11111065**	Ribosome recycling factor	184	complement(64252..64803)	13_GENE_65
**11111**	**64**	**CGL2_11111064**	Alanine racemase (EC 5.1.1.1)	406	complement(63015..64232)	13_GENE_64
**11111**	**63**	**CGL2_11111063**	Putative integral membrane protein	255	complement(62248..63012)	13_GENE_63
**11111**	**62**	**CGL2_11111062**	ABC transporter ATP-binding protein (EC 3.6.3.25)	264	complement(61466..62257)	13_GENE_62
**11111**	**61**	**CGL2_11111061**	Putative ABC transporter	518	complement(59841..61394)	13_GENE_61
**11111**	**60**	**CGL2_11111060**	ResB-like family protein (poss cyt biosyn)	513	complement(58137..59675)	13_GENE_60
**11111**	**59**	**CGL2_11111059**	Putative cytochrome c biogenesis protein	283	complement(57130..57978)	13_GENE_59
**11111**	**58**	**CGL2_11111058**	Putative cytochrome c biogenesis protein	199	complement(56521..57117)	13_GENE_58
**11111**	**57**	**CGL2_11111057**	Putative thioredoxin family protein	171	complement(55998..56510)	13_GENE_57
**11111**	**56**	**CGL2_11111056**	Putative cytochrome c biogenesis protein (CcdA)	249	complement(55204..55950)	13_GENE_56
**11111**	**55**	**CGL2_11111055**	Geranylgeranyl reductase family protein	416	complement(53919..55166)	13_GENE_55
**11111**	**54**	**CGL2_11111054**	Probable protease family protein	402	complement(52714..53919)	13_GENE_54
**11111**	**53**	**CGL2_11111053**	Primosomal protein N' (PriA)	661	50781..52763	13_GENE_53
**11111**	**52**	**CGL2_11111052**	Regulatory protein	155	50317..50781	13_GENE_52
**11111**	**51**	**CGL2_11111051**	Glycine hydroxymethyltransferase (EC 2.1.2.1)	414	49048..50289	13_GENE_51
**11111**	**50**	**CGL2_11111050**	Ribose/galactose isomerase (EC 5.3.1.26)	158	48544..49017	13_GENE_50
**11111**	**49**	**CGL2_11111049**	Protein of unknown function	181	complement(47916..48458)	13_GENE_49
**11111**	**48**	**CGL2_11111048**	Thymidylate kinase	229	complement(47107..47793)	13_GENE_48
**11111**	**47**	**CGL2_11111047**	Thymidylate kinase (EC 2.7.4.9)	217	complement(46457..47107)	13_GENE_47
**11111**	**46**	**CGL2_11111046**	Putative Ppx/GppA phosphatase family protein	518	44901..46454	13_GENE_46
**11111**	**45**	**CGL2_11111045**	Protein of unknown function	431	complement(43510..44802)	13_GENE_45
**11111**	**44**	**CGL2_11111044**	Ubiquinone/menaquinone biosynthesis methyltransfe	259	complement(42687..43463)	13_GENE_44
**11111**	**43**	**CGL2_11111043**	Putative radical SAM family protein	530	complement(41093..42682)	13_GENE_43
**11111**	**42**	**CGL2_11111042**	Protein of unknown function	170	complement(40580..41089)	13_GENE_42
**11111**	**41**	**CGL2_11111041**	Putative 4-hydroxybenzoate polyprenyl transferase	287	complement(39720..40580)	13_GENE_41
**11111**	**40**	**CGL2_11111040**	Hypothetical protein	38	complement(39348..39461)	13_GENE_39
**11111**	**39**	**CGL2_11111039**	Hypothetical protein	85	39100..39354	13_GENE_39a
**11111**	**38**	**CGL2_11111038**	Putative major facilitator superfamily transporter	459	complement(37599..38975)	13_GENE_37
**11111**	**37**	**CGL2_11111037**	Putative potassium channel protein	347	complement(36548..37588)	13_GENE_36
**11111**	**36**	**CGL2_11111036**	Acetylornithine aminotransferase (EC 2.6.1.11)	390	complement(35308..36477)	13_GENE_35
**11111**	**35**	**CGL2_11111035**	Ornithine carbamoyltransferase (EC 2.1.3.3)	309	complement(34379..35305)	13_GENE_34
**11111**	**34**	**CGL2_11111034**	Argininosuccinate synthase (EC 6.3.4.5)	408	complement(33146..34369)	13_GENE_33
**11111**	**33**	**CGL2_11111033**	Argininosuccinate lyase (EC 4.3.2.1)	467	complement(31743..33143)	13_GENE_32
**11111**	**32**	**CGL2_11111032**	Diaminopimelate decarboxylase (EC 4.1.1.20)	414	complement(30307..31548)	13_GENE_31
**11111**	**31**	**CGL2_11111031**	Protein of unknown function	165	complement(29817..30311)	13_GENE_30
**11111**	**30**	**CGL2_11111030**	Dihydrodipicolinate synthase (EC 4.2.1.52)	295	complement(28886..29770)	13_GENE_29
**11111**	**29**	**CGL2_11111029**	Dihydrodipicolinate reductase (EC 1.3.1.26)	265	complement(28083..28877)	13_GENE_28

**11111**	**28**	**CGL2_11111028**	5-carboxymethyl-2-hydroxymuconate delta-isomeras	267	complement(27283..28083)	13_GENE_27
**11111**	**27**	**CGL2_11111027**	Putative flagellar basal body rod protein	267	complement(26200..27000)	13_GENE_26
**11111**	**26**	**CGL2_11111026**	Flagellar basal body rod protein	261	complement(25392..26174)	13_GENE_25
**11111**	**25**	**CGL2_11111025**	Hypothetical protein	212	complement(24742..25377)	13_GENE_24
**11111**	**24**	**CGL2_11111024**	Protein of unknown function	208	complement(24129..24752)	13_GENE_23
**11111**	**23**	**CGL2_11111023**	Putative flagellar P-ring protein	354	complement(23068..24129)	13_GENE_22
**11111**	**22**	**CGL2_11111022**	Conserved hypothetical protein	121	complement(22706..23068)	13_GENE_21
**11111**	**21**	**CGL2_11111021**	Putative flagellin	286	complement(21816..22673)	13_GENE_20
**11111**	**20**	**CGL2_11111020**	Protein of unknown function	119	complement(21336..21692)	13_GENE_19
**11111**	**19**	**CGL2_11111019**	Protein of unknown function	164	complement(20802..21293)	13_GENE_18
**11111**	**18**	**CGL2_11111018**	Flagellar hook-associated protein (FlgK)	561	complement(19114..20796)	13_GENE_17
**11111**	**17**	**CGL2_11111017**	Flagellar hook-associated protein (FlgL)	312	complement(18148..19083)	13_GENE_16
**11111**	**16**	**CGL2_11111016**	Putative carbon storage regulator (CsrA)	80	complement(17841..18080)	13_GENE_15
**11111**	**15**	**CGL2_11111015**	Protein of unknown function	143	complement(17413..17841)	13_GENE_14
**11111**	**14**	**CGL2_11111014**	Putative flagellin	275	complement(16416..17240)	13_GENE_13
**11111**	**13**	**CGL2_11111013**	Putative flagellar hook-associated protein (FliD)	479	complement(14878..16314)	13_GENE_12
**11111**	**12**	**CGL2_11111012**	Flagellar protein (FliS)	152	complement(14398..14853)	13_GENE_11
**11111**	**11**	**CGL2_11111011**	Hypothetical protein	114	complement(14049..14390)	13_GENE_10
**11111**	**10**	**CGL2_11111010**	Conserved protein of unknown function	326	complement(12893..13870)	13_GENE_9
**11111**	**9**	**CGL2_11111009**	Putative SAM-dependent methyltransferases	780	complement(10573..12912)	13_GENE_8
**11111**	**8**	**CGL2_11111008**	Putative heptosyltransferase family protein	519	complement(9002..10558)	13_GENE_7
**11111**	**6**	**CGL2_11111006**	Conserved hypothetical protein	599	complement(7213..9009)	13_GENE_6
**11111**	**5**	**CGL2_11111005**	Probable heptosyltransferase family protein	595	complement(5429..7213)	13_GENE_5
**11111**	**4**	**CGL2_11111004**	Hypothetical protein	217	complement(4779..5429)	13_GENE_4
**11111**	**2**	**CGL2_11111002**	Probable TonB dependent receptor	861	complement(1953..4535)	13_GENE_3
**11111**	**2**	**CGL2_11111002**	Probable TonB dependent receptor	861	complement(1953..4535)	13_GENE_3
**11111**	**1**	**CGL2_11111001**	transposase	360	complement(734..1813)	13_GENE_1
**11195**	**47**	**CGL2_11195047**	Protein of unknown function	139	complement(54662..55078)	22_GENE_48
**11195**	**46**	**CGL2_11195046**	Protein of unknown function	206	complement(53810..54427)	22_GENE_46
**11195**	**45**	**CGL2_11195045**	Inorganic diphosphatase (EC 3.6.1.1)	182	complement(53169..53714)	22_GENE_45
**11195**	**44**	**CGL2_11195044**	ABC transporter ATP-binding protein (EC 3.6.3.25)	234	complement(52439..53140)	22_GENE_44
**11195**	**43**	**CGL2_11195043**	Putative ABC transporter, permease protein	402	complement(51234..52439)	22_GENE_43
**11195**	**42**	**CGL2_11195042**	Secretion protein HlyD	380	complement(50072..51211)	22_GENE_42
**11195**	**41**	**CGL2_11195041**	Flagellar motor switch protein FliM)	338	complement(48917..49930)	22_GENE_41
**11195**	**40**	**CGL2_11195040**	Transcriptional regulator, NifA, Fis Family	550	complement(47082..48731)	22_GENE_39
**11195**	**39**	**CGL2_11195039**	NADH dehydrogenase (Quinone) precursor (EC 1.6.9	584	complement(45113..46864)	22_GENE_38
**11195**	**38**	**CGL2_11195038**	Probable NADH dehydrogenase I, M subunit	437	complement(43807..45117)	22_GENE_37
**11195**	**37**	**CGL2_11195037**	Protein of unknown function	1139	complement(40391..43807)	22_GENE_36
**11195**	**36**	**CGL2_11195036**	Probable nitrogen regulatory protein P-II	114	complement(40059..40400)	22_GENE_35
**11195**	**35**	**CGL2_11195035**	Putative major facilitator superfamily transporter	265	39251..40045	22_GENE_34
**11195**	**34**	**CGL2_11195034**	transposase	420	37992..39251	

**11195**	**33**	**CGL2_11195033**	Putative major facilitator superfamily transporter	159	37448..37924	22_GENE_34
**11195**	**32**	**CGL2_11195032**	Probable cellulose synthase operon protein C	964	complement(34438..37329)	22_GENE_33
**11195**	**31**	**CGL2_11195031**	glycosyltransferase	714	complement(32307..34448)	22_GENE_32
**11195**	**30**	**CGL2_11195030**	cyclic di-GMP binding protein	737	complement(30097..32307)	22_GENE_31
**11195**	**29**	**CGL2_11195029**	Endoglucanase (EC 3.2.1.4)	366	complement(29000..30097)	22_GENE_30
11195tRNA		**CGL2_11195R001**	TRNA Gln CTG		28853..28926	
**11195**	**27**	**CGL2_11195027**	Peptidoglycan glycosyltransferase (EC 2.4.1.129)	756	26508..28775	22_GENE_29
**11195**	**26**	**CGL2_11195026**	Protein of unknown function	125	complement(26027..26401)	22_GENE_28
**11195**	**25**	**CGL2_11195025**	Putative outer membrane protein	512	complement(24378..25913)	22_GENE_27
**11195**	**24**	**CGL2_11195024**	Protein of unknown function	247	complement(23602..24342)	22_GENE_26
**11195**	**23**	**CGL2_11195023**	Hypothetical protein	227	complement(22935..23615)	22_GENE_25
**11195**	**22**	**CGL2_11195022**	DNA repair protein (RadC)	237	complement(22096..22806)	22_GENE_24
**11195**	**21**	**CGL2_11195021**	ABC1 family transporter	557	20394..22064	22_GENE_23
**11195**	**20**	**CGL2_11195020**	Protein of unknown function	523	18783..20351	22_GENE_22
**11195**	**19**	**CGL2_11195019**	Protein of unknown function	212	complement(17924..18559)	22_GENE_21
**11195**	**18**	**CGL2_11195018**	Adenylosuccinate synthase (EC 6.3.4.4)	432	16650..17945	22_GENE_20
**11195**	**17**	**CGL2_11195017**	Protein of unknown function	104	16198..16509	22_GENE_19
**11195**	**16**	**CGL2_11195016**	Protein of unknown function	547	complement(14171..15811)	22_GENE_18
**11195**	**15**	**CGL2_11195015**	Putative two component transcriptional regulator, wi	259	complement(13236..14012)	22_GENE_17
**11195**	**14**	**CGL2_11195014**	Hypothetical protein	112	complement(12901..13236)	22_GENE_15
**11195**	**13**	**CGL2_11195013**	Periplasmic phosphate binding protein	372	complement(11721..12836)	22_GENE_14
**11195**	**12**	**CGL2_11195012**	Phosphate ABC transporter, permease protein (PstC)	340	complement(10497..11516)	22_GENE_12
**11195**	**11**	**CGL2_11195011**	Phosphate transport system permease protein 2	303	complement(9581..10489)	22_GENE_11
**11195**	**10**	**CGL2_11195010**	Phosphate import ATP-binding protein (PstB) (EC 3.6	250	complement(8801..9550)	22_GENE_10
**11195**	**9**	**CGL2_11195009**	Phosphate uptake regulator (PhoU)	217	complement(8122..8772)	22_GENE_9
**11195**	**8**	**CGL2_11195008**	Thymidylate synthase (ThyX)	272	6933..7748	22_GENE_8
**11195**	**7**	**CGL2_11195007**	Protein of unknown function	419	5429..6685	22_GENE_7
**11195**	**6**	**CGL2_11195006**	Aldehyde dehydrogenase (EC 1.2.1.3)	502	complement(3768..5273)	22_GENE_6
**11195**	**5**	**CGL2_11195005**	Hypothetical protein	52	3603..3758	22_GENE_5
**11195**	**4**	**CGL2_11195004**	Fructose-1,6-bisphosphatase (EC 3.1.3.11)	335	2480..3484	22_GENE_4
**11195**	**3**	**CGL2_11195003**	Hypothetical protein	139	1993..2409	22_GENE_3a
**11195**	**2**	**CGL2_11195002**	Protein of unknown function	150	1544..1993	22_GENE_3
**11195**	**1**	**CGL2_11195001**	Probable histone deacetylase family protein	322	541..1506	22_GENE_2
**11364**	**1**	**CGL2_11364001**	Transcription termination factor Rho	416	455..1702	174_GENE_2
**11364**	**2**	**CGL2_11364002**	Ribosomal protein L31	68	1766..1969	174_GENE_3
**11364**	**3**	**CGL2_11364003**	Peptide chain release factor 1	361	1982..3064	174_GENE_4
**11364**	**4**	**CGL2_11364004**	Modification methylase (HemK)	293	3064..3942	174_GENE_5
**11364**	**5**	**CGL2_11364005**	UDP-N-acetylglucosamine 1-carboxyvinyltransferase	442	3951..5276	174_GENE_6
**11364**	**6**	**CGL2_11364006**	ATP phosphoribosyltransferase (EC 2.4.2.17)	205	5324..5938	174_GENE_7
**11364**	**7**	**CGL2_11364007**	Histidinol dehydrogenase (EC 1.1.1.23) (HDH)	445	5975..7309	174_GENE_8
**11364**	**8**	**CGL2_11364008**	Histidinol-phosphate aminotransferase (EC 2.6.1.9)	359	7309..8385	174_GENE_9

**11364**	**9**	**CGL2_11364009**	Imidazoleglycerol-phosphate dehydratase (EC 4.2.1.	210	8385..9014	174_GENE_10
**11364**	**10**	**CGL2_11364010**	Imidazole glycerol phosphate synthase, glutamine ar	211	9014..9646	174_GENE_11
**11364**	**11**	**CGL2_11364011**	1-(5-phosphoribosyl)-5-[(5-phosphoribosylamino)me	247	9627..10367	174_GENE_12
**11364**	**12**	**CGL2_11364012**	Histidine biosynthesis protein (HisF) (EC 5.3.1.16)	255	10360..11124	174_GENE_13
**11364**	**13**	**CGL2_11364013**	Putative histidine biosynthesis bifunctional protein (H	233	11124..11822	174_GENE_14
**11364**	**14**	**CGL2_11364014**	Putative histidine triad (HIT) protein	118	11822..12175	174_GENE_15
**11364**	**15**	**CGL2_11364015**	Putative histone-like DNA-binding protein	102	complement(12204..12509)	174_GENE_16
**11364**	**16**	**CGL2_11364016**	Ribosomal protein S21	65	12847..13041	
**11364**	**17**	**CGL2_11364017**	Conserved protein of unknown function	139	13104..13520	224_GENE_1
**11364**	**18**	**CGL2_11364018**	DNA primase	461	13789..15171	224_GENE_3
**11364**	**19**	**CGL2_11364019**	RNA polymerase, sigma 38 subunit, RpoS	598	15232..17025	224_GENE_4
11364tRNA		**CGL2_11364R001**	TRNA Met CAT		17030..17104	
**11364**	**20**	**CGL2_11364020**	Conserved protein of unknown function	262	17153..17938	224_GENE_5
**11364**	**21**	**CGL2_11364021**	Putative ribonuclease H	134	17938..18339	224_GENE_6
**11364**	**22**	**CGL2_11364022**	Putative metal dependent phosphohydrolase	467	18765..20165	224_GENE_7
**11364**	**23**	**CGL2_11364023**	Protein of unknown function	110	20322..20651	224_GENE_8
**11364**	**24**	**CGL2_11364024**	Putative transglycosylase	220	complement(20568..21227)	224_GENE_9
**11364**	**25**	**CGL2_11364025**	Protein of unknown function	298	21408..22301	224_GENE_10
**11364**	**26**	**CGL2_11364026**	Putative heat shock protein Hsp20	152	22401..22856	288_GENE_9
**11364**	**27**	**CGL2_11364027**	ATPase components of ABC transporters wi	643	23133..25061	288_GENE_8
**11364**	**28**	**CGL2_11364028**	Probable phosphohydrolase	298	25169..26062	288_GENE_7
**11364**	**29**	**CGL2_11364029**	Ribonuclease PH (EC 2.7.7.56)	245	26163..26897	288_GENE_6
**11364**	**30**	**CGL2_11364030**	Putative Ham1-like protein	198	26897..27490	288_GENE_5
**11364**	**31**	**CGL2_11364031**	Beta-N-acetylhexosaminidase (EC 3.2.1.52)	342	27490..28515	288_GENE_4
**11364**	**32**	**CGL2_11364032**	Glutamate racemase (EC 5.1.1.3)	314	28515..29456	288_GENE_3
**11364**	**33**	**CGL2_11364033**	Putative peptidase C26	239	complement(29329..30045)	288_GENE_2
**11364**	**34**	**CGL2_11364034**	Putative ATP-dependent RNA helicase	444	complement(30035..31366)	288_GENE_1
**11364**	**35**	**CGL2_11364035**	Hypothetical protein	32	31851..31946	175_GENE_1
**11364**	**36**	**CGL2_11364036**	Conserved protein of unknown function	165	32123..32617	175_GENE_2
**11364**	**37**	**CGL2_11364037**	Hypothetical protein	53	complement(32842..33000)	175_GENE_3
**11364**	**38**	**CGL2_11364038**	Phosphoglycerate mutase (EC 5.4.2.1)	223	complement(33000..33668)	175_GENE_4
**11364**	**39**	**CGL2_11364039**	Lipoate synthase	316	complement(33777..34724)	175_GENE_5
**11364**	**40**	**CGL2_11364040**	Mutative metal dependent phosphohydrolase	413	complement(34808..36046)	175_GENE_6
**11364**	**41**	**CGL2_11364041**	Glycogen debranching enzyme GlgX (EC 3.2.1.68)	714	36540..38681	175_GENE_8
**11364**	**42**	**CGL2_11364042**	Protein of unknown function	143	38744..39172	175_GENE_9
**11364**	**43**	**CGL2_11364043**	Putative major facilitator superfamily transporter	420	complement(39227..40486)	175_GENE_10
**11364**	**44**	**CGL2_11364044**	Putative beta-lactamase family protein	290	complement(40595..41464)	175_GENE_11
**11364**	**45**	**CGL2_11364045**	Hypothetical protein	135	41533..41937	175_GENE_12
**11364**	**46**	**CGL2_11364046**	Protein of unknown function	398	42122..43315	175_GENE_13
**11364**	**47**	**CGL2_11364047**	Conserved protein of unknown function	657	complement(43598..45568)	294_GENE_2
**11364**	**48**	**CGL2_11364048**	Protein of unknown function	281	complement(45602..46444)	294_GENE_3

**11364**	**49**	**CGL2_11364049**	Hypothetical protein	108	46617..46940	294_GENE_4
**11364**	**50**	**CGL2_11364050**	Hypothetical protein	81	46928..47170	294_GENE_5
**11364**	**51**	**CGL2_11364051**	Probable DNA helicase	455	47170..48534	294_GENE_6
**11364**	**52**	**CGL2_11364052**	Hypothetical protein	316	48494..49441	170_GENE_15
**11364**	**53**	**CGL2_11364053**	Conserved hypothetical protein	466	complement(49749..51146)	170_GENE_14
**11364**	**54**	**CGL2_11364054**	Hypothetical protein	106	51487..51804	170_GENE_13
**11364**	**55**	**CGL2_11364055**	Hypothetical protein	162	51855..52340	170_GENE_12
**11364**	**56**	**CGL2_11364056**	Protein of unknown function	204	52363..52974	170_GENE_10
**11364**	**57**	**CGL2_11364057**	Hypothetical protein	73	52974..53192	170_GENE_9
**11364**	**58**	**CGL2_11364058**	Hypothetical protein	197	53384..53974	170_GENE_8
**11364**	**59**	**CGL2_11364059**	Probable TrwB protein	672	53980..55995	170_GENE_7
**11364**	**60**	**CGL2_11364060**	Hypothetical protein	59	55995..56171	170_GENE_6
**11364**	**61**	**CGL2_11364061**	Probable TrwC protein	1128	56174..59557	170_GENE_5
**11364**	**62**	**CGL2_11364062**	Hypothetical protein	60	59578..59757	170_GENE_4
**10641**	**21**	**CGL2_10641021**	Putative transcriptional activator	80	13143..13382	5-way_CG_LeptoII_sca
**10641**	**20**	**CGL2_10641020**	Probable transposase	162	complement(13425..13910)	5-way_CG_LeptoII_sca
**10641**	**19**	**CGL2_10641019**	Hypothetical protein	85	complement(12759..13013)	170_GENE_1a
**10641**	**17**	**CGL2_10641017**	Probable conujugal transfer protein TraB	434	complement(11387..12688)	170_GENE_1
**10641**	**17**	**CGL2_10641017**	Probable conujugal transfer protein TraB	434	complement(11387..12688)	170_GENE_1
**10641**	**16**	**CGL2_10641016**	Hypothetical protein	204	complement(10760..11371)	5-way_CG_LeptoII_sca
**10641**	**15**	**CGL2_10641015**	Conserved protein of unknown function	153	complement(10284..10742)	5-way_CG_LeptoII_sca
**10641**	**14**	**CGL2_10641014**	Putative phage integrase	325	9275..10249	5-way_CG_LeptoII_sca
10641tRNA		**CGL2_10641R001**	TRNA Leu TAA		9088..9146	
**10641**	**13**	**CGL2_10641013**	Putative metallophosphoesterase	251	8064..8816	212_GENE_14
**10641**	**12**	**CGL2_10641012**	N-6 DNA methylase	524	complement(6361..7932)	212_GENE_12
**10641**	**11**	**CGL2_10641011**	Hypothetical protein	95	complement(6084..6368)	212_GENE_11
**10641**	**10**	**CGL2_10641010**	Hypothetical protein	74	complement(5863..6084)	212_GENE_10
**10641**	**9**	**CGL2_10641009**	Hypothetical protein	77	complement(5626..5856)	212_GENE_9
**10641**	**8**	**CGL2_10641008**	Hypothetical protein	133	complement(4951..5349)	212_GENE_8
**10641**	**7**	**CGL2_10641007**	Hypothetical protein	116	complement(4448..4795)	212_GENE_7
**10641**	**6**	**CGL2_10641006a**	Hypothetical protein	98	complement(3943..4236)	
**10641**	**6**	**CGL2_10641006**	Putative nucletidyltransferase	132	complement(3495..3890)	212_GENE_6
**10641**	**5**	**CGL2_10641005**	Putative Type I restriction modification system, speci	533	complement(1904..3502)	212_GENE_5
**10641**	**4**	**CGL2_10641004**	Hypothetical protein	73	complement(1547..1765)	212_GENE_4
**10641**	**3**	**CGL2_10641003**	Putative DNA damage inducible protein J	78	complement(1263..1496)	212_GENE_3
**10641**	**2**	**CGL2_10641002**	Hypothetical protein	130	complement(827..1216)	212_GENE_2
**10641**	**1**	**CGL2_10641001**	Hypothetical protein	106	complement(532..849)	212_GENE_1
**11346**	**1**	**CGL2_11346001**	ABC transporter, ATP-binding subunit	239	complement(594..1310)	232_GENE_1
**11346**	**2**	**CGL2_11346002**	Conserved hypothetical protein	222	complement(1332..1997)	232_GENE_2
**11346**	**3**	**CGL2_11346003**	Hypothetical protein	188	complement(3384..3947)	232_GENE_3
**11346**	**4**	**CGL2_11346004**	ABC transporter, ATP-binding subunit	389	complement(3957..5123)	232_GENE_4

**11346**	**5**	**CGL2_11346005**	Hypothetical protein	94	complement(5303..5584)	232_GENE_5
**11346**	**6**	**CGL2_11346006**	Hypothetical protein	344	5713..6744	232_GENE_6
**11346**	**7**	**CGL2_11346007**	Hypothetical protein	233	6957..7655	232_GENE_6a
**11346**	**8**	**CGL2_11346008**	Conserved hypothetical protein	93	complement(7652..7930)	232_GENE_8
**11346**	**9**	**CGL2_11346009**	Conserved hypothetical protein	98	complement(7937..8230)	232_GENE_9
**11346**	**10**	**CGL2_11346010**	transposase	313	8499..9437	579_GENE_7
**11346**	**11**	**CGL2_11346011**	Hypothetical protein	148	9684..10127	232_GENE_12
**11346**	**12**	**CGL2_11346012**	Hypothetical protein	223	complement(10375..11043)	
**11346**	**13**	**CGL2_11346013**	transposase	374	complement(11114..12235)	579_GENE_3
**11346**	**14**	**CGL2_11346014**	transposase	315	12265..13209	579_GENE_2
**11346**	**15**	**CGL2_11346015**	transposase	153	12951..13409	579_GENE_2
**11346**	**16**	**CGL2_11346016**	Hypothetical protein	138	13409..13822	579_GENE_1
**11346**	**16a**	**CGL2_11346016a**	Hypothetical protein	118	complement(13811..14164)	
**11346**	**17**	**CGL2_11346017**	Protein of unknown function	146	complement(14167..14604)	27_GENE_52
**11346**	**18**	**CGL2_11346018**	Protein of unknown function	84	complement(14607..14858)	27_GENE_51
**11346**	**19**	**CGL2_11346019**	Hypothetical protein	92	complement(14976..15251)	27_GENE_50
**11346**	**20**	**CGL2_11346020**	Conserved protein of unknown function	156	complement(15301..15768)	27_GENE_49
**11346**	**21**	**CGL2_11346021**	Conserved hypothetical protein	127	complement(16120..16500)	27_GENE_48
**11346**	**22**	**CGL2_11346022**	Putative helicase	1625	complement(16487..21361)	27_GENE_47
**11346**	**22**	**CGL2_11346022**	Putative helicase	1625	complement(16487..21361)	27_GENE_47
**11346**	**24**	**CGL2_11346024**	Protein of unknown function	268	complement(21476..22279)	27_GENE_46
**11346**	**25**	**CGL2_11346025**	Probable phage integrase	105	complement(22282..22596)	27_GENE_45a
**11346**	**26**	**CGL2_11346026**	Probable phage integrase CG confirms was misssemt	146	complement(22394..22831)	27_GENE_45
**11346**	**27**	**CGL2_11346027**	integrase	127	22955..23335	27_GENE_44
**11346**	**28**	**CGL2_11346028**	Protein of unknown function	193	complement(23537..24115)	27_GENE_43
**11346**	**29**	**CGL2_11346029**	Conserved hypothetical protein	359	complement(24115..25191)	27_GENE_42
**11346**	**30**	**CGL2_11346030**	Conserved protein of unknown function	423	complement(25194..26462)	27_GENE_41
**11346**	**31**	**CGL2_11346031**	Putative phage shock protein A (PspA)	225	complement(26465..27139)	27_GENE_40
**11346**	**32**	**CGL2_11346032**	Protein of unknown function	218	complement(27185..27838)	27_GENE_39
**11346**	**33**	**CGL2_11346033**	Conserved protein of unknown function	188	28016..28579	27_GENE_38
**11346**	**34**	**CGL2_11346034**	HAD-superfamily hydrolase, subfamily IA	230	complement(28558..29247)	27_GENE_37
11346tRNA		**CGL2_11346R001**	TRNA Glu CTC		complement(29317..29392)	
**11346**	**35**	**CGL2_11346035**	Protein of unknown function	148	29468..29911	27_GENE_36
**11346**	**36**	**CGL2_11346036**	Protein of unknown function	151	complement(29907..30359)	27_GENE_35
**11346**	**37**	**CGL2_11346037**	Protein of unknown function	536	30492..32099	27_GENE_34
**11346**	**38**	**CGL2_11346038**	Putative LD-carboxypeptidase family protein	319	complement(32110..33066)	27_GENE_33
**11346**	**39**	**CGL2_11346039**	Protein of unknown function	89	complement(33036..33302)	27_GENE_32
**11346**	**40**	**CGL2_11346040**	Putative cobyrinic acid a,c-diamide synthase	208	complement(33308..33931)	27_GENE_31
**11346**	**41**	**CGL2_11346041**	Putative creatininase	251	complement(33951..34703)	27_GENE_30
**11346**	**42**	**CGL2_11346042**	Putative phytoene synthase	320	34825..35784	27_GENE_29
**11346**	**43**	**CGL2_11346043**	Putative amine oxidase	491	35780..37252	27_GENE_28

**11346**	**44**	**CGL2_11346044**	Putative phytoene synthase	317	37252..38202	27_GENE_27
**11346**	**45**	**CGL2_11346045**	Putative beta-lactamase family protein	352	complement(38166..39221)	27_GENE_26
**11346**	**46**	**CGL2_11346046**	Putative peptidase U62, modulator of DNA gyrase	485	39408..40862	27_GENE_25
**11346**	**47**	**CGL2_11346047**	Putative peptidase U62, modulator of DNA gyrase	452	40862..42217	27_GENE_24
**11346**	**48**	**CGL2_11346048**	Putative decarboxylase family protein	214	42217..42858	27_GENE_23
**11346**	**49**	**CGL2_11346049**	Protein of unknown function	247	complement(42877..43617)	27_GENE_22
**11346**	**50**	**CGL2_11346050**	DNA repair protein RadA	461	43753..45135	27_GENE_21
**11346**	**51**	**CGL2_11346051**	Probable glycoprotein endopeptidase	215	45135..45779	27_GENE_20
**11346**	**52**	**CGL2_11346052**	Ribosomal-protein-alanine acetyltransferase	204	45763..46374	27_GENE_19
**11346**	**53**	**CGL2_11346053**	Protein of unknown function	78	46477..46710	27_GENE_18
**11346**	**54**	**CGL2_11346054**	Probable twin-arginine translocation protein (TatA/E)	99	46716..47012	27_GENE_17
**11346**	**55**	**CGL2_11346055**	Sec-independent protein translocase	252	47005..47760	27_GENE_16
**11346**	**56**	**CGL2_11346056**	Putative undecaprenol kinase	285	47857..48711	27_GENE_15
**11346**	**57**	**CGL2_11346057**	Formate--tetrahydrofolate ligase (EC 6.3.4.3)	557	48859..50529	27_GENE_14
**11346**	**58**	**CGL2_11346058**	Protein of unknown function	642	50529..52454	27_GENE_13
**11346**	**59**	**CGL2_11346059**	Di-trans-poly-cis-decaprenylcistransferase (EC 2.5.1.	264	52460..53251	27_GENE_12
**11346**	**60**	**CGL2_11346060**	Putative phosphatidate cytidylyltransferase	279	53251..54087	27_GENE_11
**11346**	**61**	**CGL2_11346061**	1-deoxy-D-xylulose 5-phosphate reductoisomerase (	393	54112..55290	27_GENE_10
**11346**	**62**	**CGL2_11346062**	Peptidase M50, putative membrane-associated zinc r	354	55317..56378	27_GENE_9
**11346**	**63**	**CGL2_11346063**	4-hydroxy-3-methylbut-2-en-1-yl diphosphate synth	377	56444..57574	27_GENE_8
**11346**	**64**	**CGL2_11346064**	Prolyl-tRNA synthetase	422	57591..58856	27_GENE_7
**11346**	**65**	**CGL2_11346065**	Conserved hypothetical protein	164	58863..59354	27_GENE_6
**11346**	**66**	**CGL2_11346066**	Ribosomal protein S32	37	59409..59519	27_GENE_5
**11346**	**67**	**CGL2_11346067**	Ribosomal protein S32	23	59519..59587	27_GENE_5
**11346**	**68**	**CGL2_11346068**	Fatty acid/phospholipid synthesis protein (PlsX)	331	59603..60595	27_GENE_4
**11346**	**69**	**CGL2_11346069**	3-oxoacyl-(Acyl-carrier-protein) synthase III (EC 2.3	341	60595..61617	27_GENE_3
**11346**	**70**	**CGL2_11346070**	Malonyl CoA-acyl carrier protein transacylase (EC 2.3	314	61617..62558	27_GENE_2
**11346**	**71**	**CGL2_11346071**	Malonyl CoA-acyl carrier protein transacylase (EC 2.3	251	62558..63310	27_GENE_1
**11346**	**72**	**CGL2_11346072**	Acyl carrier protein (ACP)	76	63392..63619	295_GENE_10
**11346**	**73**	**CGL2_11346073**	3-oxoacyl-(Acyl-carrier-protein) synthase II (EC 2.3.	419	63622..64878	295_GENE_9
**11346**	**74**	**CGL2_11346074**	Ribonuclease III (EC 3.1.26.3)	247	64979..65719	295_GENE_8
**11346**	**75**	**CGL2_11346075**	hypothetical protein predicted by Glimmer/Critic	382	complement(65748..66893)	295_GENE_7
**11346**	**76**	**CGL2_11346076**	Hypothetical protein	354	complement(66911..67972)	295_GENE_6
**11346**	**77**	**CGL2_11346077**	Phosphoribosylformylglycinamidine cyclo-ligase (EC (	350	68302..69351	295_GENE_5
**11346**	**78**	**CGL2_11346078**	Phosphoribosylglycinamide formyltransferase (EC 2.1	207	69338..69958	295_GENE_4
11346tRNA		**CGL2_11346R002**	TRNA Ala CGC		70039..70114	
**11346**	**79**	**CGL2_11346079**	Hypothetical protein	69	complement(70138..70344)	295_GENE_3
**11346**	**80**	**CGL2_11346080**	6-phosphogluconate dehydrogenase (decarboxylating	339	70440..71456	135_GENE_12
**11346**	**81**	**CGL2_11346081**	Glucose-6-phosphate dehydrogenase (EC 1.1.1.49)	468	71461..72864	135_GENE_11
**11346**	**82**	**CGL2_11346082**	Serine 3-dehydrogenase (EC 1.1.1.276)	247	72942..73682	135_GENE_10
**11346**	**83**	**CGL2_11346083**	Conserved protein of unknown function	344	73748..74779	135_GENE_9

**11346**	**84**	**CGL2_11346084**	Methyl-accepting chemotaxis sensory transducer	577	complement(74814..76544)	135_GENE_8
**11346**	**85**	**CGL2_11346085**	Amino-acid N-acetyltransferase (EC 2.3.1.1)	448	76933..78276	135_GENE_6
**11346**	**86**	**CGL2_11346086**	Protein of unknown function	207	78770..79390	135_GENE_5
**11346**	**87**	**CGL2_11346087**	Putative response regulator receiver protein	322	79589..80554	135_GENE_4
**11346**	**88**	**CGL2_11346088**	Phosphoenolpyruvate synthase (EC 2.7.9.2)	799	80931..83327	135_GENE_3
**11346**	**89**	**CGL2_11346089**	GTP-binding protein (EngA)	469	complement(83398..84804)	135_GENE_2
**11346**	**90**	**CGL2_11346090**	Protein of unknown function	230	complement(84804..85493)	135_GENE_1
**11346**	**92**	**CGL2_11346092**	Probable purine-cytosine permease	477	85875..87305	14_GENE_68
**11346**	**93**	**CGL2_11346093**	NADH dehydrogenase (EC 1.6.99.3)	452	87301..88656	14_GENE_67
**11346**	**94**	**CGL2_11346094**	Putative amino acid permease	651	complement(88659..90611)	14_GENE_66
**11346**	**95**	**CGL2_11346095**	Hypothetical protein	519	complement(90800..92356)	14_GENE_65
**11346**	**96**	**CGL2_11346096**	Hypothetical protein	175	complement(92416..92940)	14_GENE_64
**11346**	**97**	**CGL2_11346097**	Hypothetical protein	54	complement(93068..93229)	14_GENE_63
**11346**	**98**	**CGL2_11346098**	Hypothetical protein	98	complement(93300..93593)	14_GENE_62
**11346**	**99**	**CGL2_11346099**	Hypothetical protein	332	complement(93714..94709)	14_GENE_61
**11346**	**100**	**CGL2_11346100**	Putative amino acid permease	476	complement(94788..96215)	14_GENE_60
**11346**	**101**	**CGL2_11346101**	Conserved protein of unknown function	176	complement(96288..96815)	14_GENE_59
**11346**	**102**	**CGL2_11346102**	Putative acetyltransferase	164	complement(96867..97358)	14_GENE_58
**11346**	**103**	**CGL2_11346103**	Deoxyribonuclease, TatD family	263	complement(97361..98149)	14_GENE_57
**11346**	**104**	**CGL2_11346104**	Methionyl-tRNA synthetase (EC 6.1.1.10)	673	complement(98157..100175)	14_GENE_56
**11346**	**105**	**CGL2_11346105**	Conserved protein of unknown function	240	complement(100214..100933)	14_GENE_55
**11346**	**106**	**CGL2_11346106**	DNA polymerase III (EC 2.7.7.7)	343	complement(101001..102029)	14_GENE_54
**11346**	**107**	**CGL2_11346107**	Thymidylate kinase (EC 2.7.4.9)	220	complement(102029..102688)	14_GENE_53
**11346**	**108**	**CGL2_11346108**	Hypothetical protein	43	complement(102857..102985)	14_GENE_52
**11346**	**109**	**CGL2_11346109**	Putative ferric uptake regulator, Fur family	155	103267..103731	14_GENE_51
**11346**	**110**	**CGL2_11346110**	Cytochrome c peroxidase (EC 1.11.1.5)	338	103757..104770	14_GENE_50
**11346**	**111**	**CGL2_11346111**	Putative alkyl hydroperoxide reducatase	200	104922..105521	14_GENE_49
**11346**	**112**	**CGL2_11346112**	Putative acireductone dioxygenase	186	105580..106137	14_GENE_48
**11346**	**113**	**CGL2_11346113**	Ribulose-bisphosphate carboxylase (EC 4.1.1.39)	389	106140..107306	14_GENE_47
**11346**	**114**	**CGL2_11346114**	Putative hydrolase, haloacid dehalogenase-like famil	216	107306..107953	14_GENE_46
**11346**	**115**	**CGL2_11346115**	Putative aldolase class II	200	107949..108548	14_GENE_45
**11346**	**116**	**CGL2_11346116**	Transcriptional regulator, MarR family	159	108707..109183	14_GENE_44
**11346**	**117**	**CGL2_11346117**	Conserved hypothetical protein	150	109215..109664	14_GENE_43
**11346**	**118**	**CGL2_11346118**	Conserved protein of unknow function	76	109766..109993	14_GENE_42
**11346**	**119**	**CGL2_11346119**	Putative silent information regulator protein, Sir2 fan	279	complement(110082..110918)	14_GENE_41
**11346**	**120**	**CGL2_11346120**	Hypothetical protein	125	111491..111865	14_GENE_39
**11346**	**121**	**CGL2_11346121**	Putative transcriptional regulator, ArsR family	117	112055..112405	14_GENE_38
**11346**	**122**	**CGL2_11346122**	Glyoxalase family protein	146	112405..112842	14_GENE_37
**11346**	**123**	**CGL2_11346123**	Hypothetical protein	285	complement(112871..113725)	14_GENE_36
**11346**	**124**	**CGL2_11346124**	Conserved protein of unknown function	222	113943..114608	14_GENE_35
**11346**	**125**	**CGL2_11346125**	Glucosamine-1-phosphate n-acetyltransferase] (EC 2	469	114601..116007	14_GENE_34

**11346**	**126**	**CGL2_11346126**	Glucosamine--fructose-6-phosphate aminotransferas	605	116012..117826	14_GENE_33
**11346**	**127**	**CGL2_11346127**	Protein of unknown function	439	117865..119181	14_GENE_32
**11346**	**128**	**CGL2_11346128**	Putative ATP-dependent DNA helicase, UvrD/REP fam	746	119181..121418	14_GENE_31
**11346**	**129**	**CGL2_11346129**	Glutamyl-tRNA(Gln) amidotransferase, C subunit	99	121565..121861	14_GENE_30
**11346**	**130**	**CGL2_11346130**	Aspartate 1-decarboxylase (EC 4.1.1.11	121	121869..122231	14_GENE_29
**11346**	**131**	**CGL2_11346131**	Glutamyl-tRNA(Gln) amidotransferase A subunit / An	492	122231..123706	14_GENE_28
**11346**	**132**	**CGL2_11346132**	Glutamyl-tRNA(Gln) amidotransferase B subunit	490	123706..125175	14_GENE_27
**11346**	**133**	**CGL2_11346133**	Protein of unknown function	227	125175..125855	14_GENE_26
**11346**	**134**	**CGL2_11346134**	Probable Cytochrome c, NapC/NirT family	166	125871..126368	14_GENE_25
**11346**	**135**	**CGL2_11346135**	Enolase (EC 4.2.1.11)	427	126381..127661	14_GENE_24
**11346**	**136**	**CGL2_11346136**	Hypothetical protein	113	127672..128010	14_GENE_23
**11346**	**137**	**CGL2_11346137**	Protein of unknown function	146	complement(128038..128475)	14_GENE_22
**11346**	**138**	**CGL2_11346138**	Probable cytochrome c, class I	185	129079..129633	14_GENE_20
**11346**	**139**	**CGL2_11346139**	Putative sigma54 specific transcriptional regulator, F	463	130191..131579	14_GENE_19
**11346**	**140**	**CGL2_11346140**	Hypothetical protein	168	132055..132558	14_GENE_18
**11346**	**141**	**CGL2_11346141**	Hypothetical protein	121	132641..133003	14_GENE_17
**11346**	**142**	**CGL2_11346142**	Putative peptidase, C39 family	279	133087..133923	14_GENE_16
**11346**	**143**	**CGL2_11346143**	Hypothetical protein	246	133957..134694	14_GENE_15
**11346**	**144**	**CGL2_11346144**	Conserved hypothetical protein	375	135338..136462	14_GENE_14
**11346**	**145**	**CGL2_11346145**	Putative Outer membrane protein, OmpAfamily	521	136474..138036	14_GENE_13
**11346**	**146**	**CGL2_11346146**	Protein of unknown function	292	complement(138057..138932)	14_GENE_12
**11346**	**147**	**CGL2_11346147**	Flavin oxidoreductase/NADH oxidase (EC 1.6.99.1)	367	complement(139106..140206)	14_GENE_11
**11346**	**148**	**CGL2_11346148**	Putative transcriptional regulator, BadM/Rrf2 family	159	complement(140289..140765)	14_GENE_10
**11346**	**149**	**CGL2_11346149**	Phosphoglucosamine mutase (EC 5.4.2.10)	457	140920..142290	14_GENE_8
**11346**	**150**	**CGL2_11346150**	Competence protein (ComEC)	482	142566..144011	14_GENE_7
**11346**	**151**	**CGL2_11346151**	Putative DNA methylase N-4/N-6	453	complement(143987..145345)	14_GENE_6
**11346**	**152**	**CGL2_11346152**	Hypothetical protein	46	145522..145659	14_GENE_5
**11346**	**153**	**CGL2_11346153**	Protein of unknown function	207	complement(145672..146292)	14_GENE_4
**11346**	**154**	**CGL2_11346154**	NADH dehydrogenase (Quinone) (EC 1.6.99.5)	634	146431..148332	14_GENE_3
**11346**	**154a**	**CGL2_11346154a**	transposase	446	148469..149806	
11346tRNA		**CGL2_11346R003**	TRNA Lys CTT		150149..150224	
**11346**	**155**	**CGL2_11346155**	Diguanylate cyclase with GAF sensor	322	150471..151436	14_GENE_2
**11346**	**156**	**CGL2_11346156**	Conserved hypothetical protein	262	151662..152447	51_GENE_7
**11346**	**157**	**CGL2_11346157**	Protein of unknown function	409	152512..153738	51_GENE_8
**11346**	**158**	**CGL2_11346158**	Secretion protein (HlyD)	385	complement(153802..154956)	51_GENE_9
**11346**	**159**	**CGL2_11346159**	Hydrophobe/amphiphile efflux family protein	1042	complement(155012..158137)	51_GENE_10
**11346**	**160**	**CGL2_11346160**	RND efflux system, outer membrane lipoprotein (Noc	500	complement(158198..159697)	51_GENE_11
**11346**	**161**	**CGL2_11346161**	Hypothetical protein	63	complement(159841..160029)	51_GENE_12
**11346**	**162**	**CGL2_11346162**	Putative transcriptional regulator, MarR family	181	160165..160707	51_GENE_13
**11346**	**163**	**CGL2_11346163**	Cinnamyl-alcohol dehydrogenase (EC 1.1.1.195)	334	160786..161787	51_GENE_14
**11346**	**164**	**CGL2_11346164**	Putative exodeoxyribonuclease V, gamma subunit	1095	161852..165136	51_GENE_15

**11346**	**165**	**CGL2_11346165**	Putative exodeoxyribonuclease V, beta subunit	1095	165136..168420	51_GENE_16
**11346**	**166**	**CGL2_11346166**	Exodeoxyribonuclease V, alpha subunit (EC 3.1.11.5)	575	168420..170144	51_GENE_17
**11346**	**167**	**CGL2_11346167**	Conserved protein of unknow function	364	complement(170172..171263)	51_GENE_18
**11346**	**168**	**CGL2_11346168**	Putative two component transcriptional regulator, wi	253	complement(171263..172021)	51_GENE_19
**11346**	**169**	**CGL2_11346169**	Protein of unknown function	144	complement(172379..172810)	51_GENE_20
**11346**	**170**	**CGL2_11346170**	Probable TonB-like protein	216	complement(172824..173471)	51_GENE_21
**11346**	**171**	**CGL2_11346171**	Putative biopolymer transport protein	139	complement(173471..173887)	51_GENE_22
**11346**	**172**	**CGL2_11346172**	Putative biopolymer transport protein	234	complement(173954..174655)	51_GENE_23
**11346**	**173**	**CGL2_11346173**	Probable TonB-dependent receptor	974	complement(174823..177744)	51_GENE_24
**11346**	**174**	**CGL2_11346174**	Conserved protein of unknown function	238	178026..178739	51_GENE_26
**11346**	**175**	**CGL2_11346175**	Protein of unknown function	147	179281..179721	51_GENE_27
**11346**	**175a**	**CGL2_11346175a**	Conserved hypothetical protein	213	complement(179805..180443)	
**11346**	**176**	**CGL2_11346176**	Transposase	416	complement(180436..181683)	
**11346**	**177**	**CGL2_11346177**	Hypothetical protein	2132	complement(181919..188314)	
**11346**	**178**	**CGL2_11346178**	Putative Type I Restriction modification system, S su	360	complement(188806..189885)	
**11346**	**179**	**CGL2_11346179**	Putative Type I Restriction modification system, M su	549	complement(189885..191531)	
**11346**	**180**	**CGL2_11346180**	Putative DEAD/DEAH box helicase	756	complement(191575..193842)	
**11346**	**181**	**CGL2_11346181**	Conserved hypothetical protein	317	complement(193870..194820)	
**11346**	**182**	**CGL2_11346182**	Hypothetical protein	144	complement(194866..195297)	
**11346**	**183**	**CGL2_11346183**	Hypothetical protein	164	complement(195704..196195)	265_GENE_7
**11346**	**184**	**CGL2_11346184**	Probable XRE family transcriptional regulator	175	complement(196188..196712)	265_GENE_6
**11346**	**185**	**CGL2_11346185**	Conserved hypothetical protein	119	complement(196728..197084)	
**11346**	**186**	**CGL2_11346186**	Putative mobilization protein TraA	899	complement(197283..199979)	219_GENE_18
**11346**	**8**	**CGL2_11346008**	Conserved protein of unknow function	93	complement(7652..7930)	232_GENE_8
**11346**	**9**	**CGL2_11346009**	Conserved protein of unknow function	98	complement(7937..8230)	232_GENE_9
**9314**	**5**	**CGL2_09314005**	Putative phage integrase	330	complement(2687..3676)	219_GENE_5
**9314**	**4**	**CGL2_09314004**	Conserved protein of unknown function	73	complement(2385..2603)	219_GENE_4
**9314**	**3**	**CGL2_09314003**	Probable plasmid stabilization system protein	100	complement(2083..2382)	219_GENE_3
**9314**	**2**	**CGL2_09314002**	Hypothetical protein	145	complement(1424..1858)	
**9314**	**1**	**CGL2_09314001**	Hypothetical protein	226	complement(736..1413)	
**11185**	**11**	**CGL2_11185011**	Protein of unknown function	240	8393..9112	
**11346**	**187**	**CGL2_11346187**	Hypothetical protein	110	complement(199930..200259)	219_GENE_17
**11346**	**188**	**CGL2_11346188**	mobilization protein, MobE	210	complement(200263..200892)	219_GENE_16
**11346**	**189**	**CGL2_11346189**	mobilization protein, MobD	132	complement(200892..201287)	219_GENE_15
**11346**	**190**	**CGL2_11346190**	Conserved hypothetical protein	111	complement(201218..201550)	219_GENE_14
**11346**	**191**	**CGL2_11346191**	Protein of unknown function	163	complement(201578..202066)	219_GENE_13
**11346**	**192**	**CGL2_11346192**	Hypothetical protein	138	complement(202318..202731)	219_GENE_12
**11346**	**193**	**CGL2_11346193**	Hypothetical protein	430	complement(202860..204149)	219_GENE_10
**11346**	**194**	**CGL2_11346194**	Hypothetical protein	62	complement(204154..204339)	219_GENE_9
**11346**	**195**	**CGL2_11346195**	Hypothetical protein	236	204449..205156	219_GENE_8
**11346**	**196**	**CGL2_11346196**	Integrase	388	205199..206362	219_GENE_7

11346tRNA		**CGL2_11346R004**	TRNA Gly GCC		complement(206302..206376)	
**11346**	**197**	**CGL2_11346197**	Probable outer membrane lipoprotein	173	complement(206523..207041)	219_GENE_6
		Strain variant path	Strain variant path			Strain variant path
**10850**	**18**	**CGL2_10850018**	Putative pirin protein	301	complement(19392..20294)	98_GENE_19
**10850**	**17**	**CGL2_10850017**	Putative potassium channel protein	421	complement(17862..19124)	98_GENE_18
**10850**	**16**	**CGL2_10850016**	Deoxyribodipyrimidine photolyase (EC 4.1.99.3)	534	16247..17848	98_GENE_17
**10850**	**15**	**CGL2_10850015**	Protein of unknown function	276	complement(15086..15913)	98_GENE_16
**10850**	**14**	**CGL2_10850014**	Putative secretion protein (HlyD)	299	14139..15035	98_GENE_15
**10850**	**13**	**CGL2_10850013**	Conserved hypothetical protein	68	13936..14139	98_GENE_14
**10850**	**12**	**CGL2_10850012**	Putative fusaric acid resistance protein	682	11891..13936	98_GENE_13
**10850**	**11**	**CGL2_10850011**	Protein of unknown function	240	10986..11705	98_GENE_12
**10850**	**10**	**CGL2_10850010**	Protein of unknown function	386	9698..10855	98_GENE_11
**10850**	**9**	**CGL2_10850009**	Protein of unknown function	214	9028..9669	98_GENE_10
**10850**	**8**	**CGL2_10850008**	a+/H+ antiporter	535	7213..8817	98_GENE_9
**10850**	**7**	**CGL2_10850007**	Putative transcriptional regulator, TetR family	219	6352..7008	98_GENE_8
**10850**	**6**	**CGL2_10850006**	Putative isochorismatase hydrolase	188	complement(5681..6244)	98_GENE_7
**10850**	**5**	**CGL2_10850005**	Putative ammonia monooxygenase	345	complement(4543..5577)	98_GENE_6
10850tRNA		**CGL2_10850R001**	TRNA Asn GTT		4384..4459	
**10850**	**4**	**CGL2_10850004**	Conserved protein of unknown function	235	3574..4278	98_GENE_4
**10850**	**3**	**CGL2_10850003**	Conserved protein of unknown function	293	2696..3574	98_GENE_3
**10850**	**2**	**CGL2_10850002**	Dihydropteroate synthase (EC 2.5.1.15)	283	1843..2691	98_GENE_2
**10850**	**1**	**CGL2_10850001**	Peptidase M41, FtsH (EC 3.6.4.6)	592	78..1853	98_GENE_1
**11247**	**23**	**CGL2_11247023**	Hypoxanthine phosphoribosyl transferase (EC 2.4.2.8	176	24189..24716	76_GENE_1
**11247**	**22**	**CGL2_11247022**	TRNA(Ile)-lysidine synthetase	504	22702..24213	76_GENE_2
**11247**	**21**	**CGL2_11247021**	Riboflavin kinase / FAD synthase (RibC) (EC 2.7.7.2)	319	21771..22727	76_GENE_3
**11247**	**20**	**CGL2_11247020**	Porphobilinogen synthase (EC 4.2.1.24)	326	20743..21720	76_GENE_4
**11247**	**19**	**CGL2_11247019**	Uroporphyrin-III C-methyltransferase / synthase (EC	513	19166..20704	76_GENE_5
**11247**	**18**	**CGL2_11247018**	Porphobilinogen deaminase (EC 2.5.1.61)	314	17662..18603	76_GENE_6
**11247**	**17**	**CGL2_11247017**	Glutamyl-tRNA reductase	452	16304..17659	76_GENE_7
**11247**	**16**	**CGL2_11247016**	Putative Cytochrome c assembly protein	269	15425..16231	76_GENE_8
**11247**	**15**	**CGL2_11247015**	Protein of unknown function	515	complement(13749..15293)	76_GENE_9
**11247**	**14**	**CGL2_11247014**	Lysine 2,3-aminomutase	383	complement(12597..13745)	76_GENE_10
**11247**	**13**	**CGL2_11247013**	Nitroreductase	249	complement(11824..12570)	76_GENE_11
**11247**	**12**	**CGL2_11247012**	Putative 2-deoxycytidine 5-triphosphate deaminase	366	complement(10736..11833)	76_GENE_12
**11247**	**11**	**CGL2_11247011**	Putative AAA-family ATPase	575	complement(8846..10570)	76_GENE_13
**11247**	**10**	**CGL2_11247010**	Putative proteasome component	511	complement(7314..8846)	76_GENE_14
**11247**	**9**	**CGL2_11247009**	Protein of unknown function	64	complement(7103..7294)	76_GENE_15
**11247**	**8**	**CGL2_11247008**	Putative 20S proteasome beta-subunit	265	complement(6301..7095)	76_GENE_16
**11247**	**7**	**CGL2_11247007**	Probable 20S proteasome alpha-subunit	251	complement(5553..6305)	76_GENE_17
**11247**	**6**	**CGL2_11247006**	Conserved protein of unknown function	502	complement(4120..5625)	76_GENE_18
**11247**	**5**	**CGL2_11247005**	Dihydroorotate dehydrogenase (EC 1.3.3.1)	368	3014..4117	76_GENE_19

**11247**	**4**	**CGL2_11247004**	Homoserine kinase (EC 2.7.1.39)	331	2041..3033	76_GENE_20
**11247**	**3**	**CGL2_11247003**	Probable ferredoxin	112	1710..2045	76_GENE_21
**11247**	**1**	**CGL2_11247001**	Conserved hypothetical protein	239	791..1507	76_GENE_22
**11077**	**1**	**CGL2_11077001**	Hypothetical protein	37	42..152	
**11077**	**2**	**CGL2_11077002**	Hypothetical protein	66	215..412	25_GENE_1
**11077**	**3**	**CGL2_11077003**	Probable cytochrome C oxidase mono-heme subunit	176	477..1004	25_GENE_2
**11077**	**4**	**CGL2_11077004**	Protein of unknown function	110	1117..1446	25_GENE_3
**11077**	**5**	**CGL2_11077005**	Hypothetical protein	68	1716..1919	25_GENE_4
**11077**	**6**	**CGL2_11077006**	Protein of unknown function	143	1934..2362	25_GENE_5
**11077**	**7**	**CGL2_11077007**	Protein of unknown function	68	2402..2605	25_GENE_6
**11077**	**8**	**CGL2_11077008**	Protein of unknown function	155	2628..3092	25_GENE_7
**11077**	**9**	**CGL2_11077009**	Probable cytochrome-c oxidase	185	3092..3646	25_GENE_8
**11077**	**10**	**CGL2_11077010**	Conserved protein of unknown function	362	3646..4731	25_GENE_9
**11077**	**11**	**CGL2_11077011**	Probable cytochrome C oxidase	188	4770..5333	25_GENE_10
**11077**	**12**	**CGL2_11077012**	Hypothetical protein	89	5338..5604	25_GENE_11
**11077**	**13**	**CGL2_11077013**	Probable iron-sulfur protein	442	5692..7017	25_GENE_12
**11077**	**14**	**CGL2_11077014**	Protein of unknown function	155	7096..7560	25_GENE_13
**11077**	**15**	**CGL2_11077015**	Hypothetical protein	108	7544..7867	25_GENE_13a
**11077**	**16**	**CGL2_11077016**	Conserved hypothetical protein	202	7887..8492	25_GENE_15
**11077**	**17**	**CGL2_11077017**	Putative ADP-ribose pyrophosphatase	198	8482..9075	25_GENE_16
**11077**	**18**	**CGL2_11077018**	Probable ferredoxin	63	9106..9294	25_GENE_17
**11077**	**19**	**CGL2_11077019**	Sigma54 specific transcriptional regulator, Fis family	473	9301..10719	25_GENE_18
**11077**	**20**	**CGL2_11077020**	Anthranilate synthase component I (EC 4.1.3.27)	501	10719..12221	25_GENE_19
**11077**	**21**	**CGL2_11077021**	Anthranilate synthase component II (EC 4.1.3.27)	198	12212..12805	25_GENE_20
**11077**	**22**	**CGL2_11077022**	Anthranilate phosphoribosyltransferase (EC 2.4.2.18	341	12805..13827	25_GENE_21
**11077**	**23**	**CGL2_11077023**	Indole-3-glycerol phosphate synthase (EC 4.1.1.48)	274	13797..14618	25_GENE_22
**11077**	**24**	**CGL2_11077024**	Phosphoribosylanthranilate isomerase (EC 5.3.1.24)	212	14611..15246	25_GENE_23
**11077**	**25**	**CGL2_11077025**	Tryptophan synthase, beta subunit (EC 4.2.1.20)	412	15236..16471	25_GENE_24
**11077**	**26**	**CGL2_11077026**	Tryptophan synthase, alpha chain (EC 4.2.1.20)	264	16471..17262	25_GENE_25
**11077**	**27**	**CGL2_11077027**	Acetyl-CoA carboxylase carboxyltransferase, beta su	304	17318..18229	25_GENE_26
**11077**	**28**	**CGL2_11077028**	Folylpolyglutamate synthase (EC 6.3.2.17)	444	18233..19564	25_GENE_27
**11077**	**29**	**CGL2_11077029**	Probable organic solvent tolerance protein (OstA)	767	19564..21864	25_GENE_28
**11077**	**30**	**CGL2_11077030**	Conserved protein of unknown function	266	complement(21891..22688)	25_GENE_29
**11077**	**31**	**CGL2_11077031**	NAD(+) kinase (EC 2.7.1.23)	305	complement(22753..23667)	25_GENE_30
**11077**	**32**	**CGL2_11077032**	Glutamate synthase, small subunit (EC 1.4.1.13)	613	24161..25999	25_GENE_31
**11077**	**33**	**CGL2_11077033**	Putative rieske iron-sulfur family protein	206	25999..26616	25_GENE_32
**11077**	**34**	**CGL2_11077034**	Putative cytochrome b/b6, N-terminal	253	26795..27553	25_GENE_33
**11077**	**35**	**CGL2_11077035**	Probable cytochrome b/b6, C-terminal	276	27611..28438	25_GENE_34
**11077**	**36**	**CGL2_11077036**	Protein of unknown function	72	28540..28755	25_GENE_35
**11077**	**37**	**CGL2_11077037**	Probable cytochrome c, class I	321	28755..29717	25_GENE_36

**11077**	**38**	**CGL2_11077038**	Daunorubicin resistance ABC transporter ATP-binding	327	29802..30782	25_GENE_37
**11077**	**39**	**CGL2_11077039**	Putative ABC-2 type transporter	273	30782..31600	25_GENE_38
**11077**	**40**	**CGL2_11077040**	Conserved protein of unknown function	164	31704..32195	25_GENE_39
**11077**	**40**	**CGL2_11077040**	Conserved protein of unknown function	164	31704..32195	25_GENE_39
**11077**	**42**	**CGL2_11077042**	Signal peptide peptidase, SppA	349	32210..33256	25_GENE_40
**11077**	**43**	**CGL2_11077043**	Protein of unknown function	119	complement(33223..33579)	25_GENE_41
**11077**	**44**	**CGL2_11077044**	Putative hydroxypyruvate reductase/glycerate kinase	431	complement(33579..34871)	25_GENE_42
**11077**	**45**	**CGL2_11077045**	Hypothetical protein	87	complement(34879..35139)	25_GENE_43
**11077**	**46**	**CGL2_11077046**	Conserved protein of unknown function	359	35184..36260	25_GENE_44
**11077**	**47**	**CGL2_11077047**	Protein of unknown function	115	complement(36277..36621)	25_GENE_45
**11079**	**1**	**CGL2_11079001**	Protein of unknown function	220	complement(739..1398)	25_GENE_46
**11079**	**2**	**CGL2_11079002**	Protein of unknown function	115	complement(1478..1822)	25_GENE_47
**11079**	**3**	**CGL2_11079003**	Conserved hypothetical protein	168	1908..2411	25_GENE_48
**11079**	**4**	**CGL2_11079004**	Putative major facilitator superfamily transporter	404	2386..3597	25_GENE_49
**11080**	**1**	**CGL2_11080001**	Putative CBS domain containing membrane protein	152	1065..1520	25_GENE_50
**11080**	**2**	**CGL2_11080002**	Conserved protein of unknown function	175	1510..2034	25_GENE_51
**11080**	**3**	**CGL2_11080003**	Putative histidine triad (HIT) protein	179	2048..2584	25_GENE_52
**11080**	**4**	**CGL2_11080004**	Protein of unknown function	231	2602..3294	25_GENE_53
**11080**	**5**	**CGL2_11080005**	Uracil phosphoribosyltransferase (EC 2.4.2.9)	199	3354..3950	25_GENE_54
**11080**	**6**	**CGL2_11080006**	Aspartate carbamoyltransferase (EC 2.1.3.2)	308	3950..4873	25_GENE_55
**11080**	**7**	**CGL2_11080007**	Dihydroorotase (EC 3.5.2.3)	438	4906..6219	25_GENE_56
**11080**	**8**	**CGL2_11080008**	Carbamoyl-phosphate synthase, small subunit (EC 6	365	6219..7313	25_GENE_57
**11080**	**9**	**CGL2_11080009**	Putative radical SAM family protein	332	7306..8301	25_GENE_58
**11080**	**10**	**CGL2_11080010**	Carbamoyl-phosphate synthase, large subunit (EC 6.	1080	8297..11536	25_GENE_59
**11080**	**11**	**CGL2_11080011**	Putative transcription elongation factor GreA	160	11616..12095	25_GENE_60
**11080**	**12**	**CGL2_11080012**	Hypothetical protein	41	12133..12255	25_GENE_61
**11080**	**13**	**CGL2_11080013**	Hypothetical protein	25	12437..12511	
**11390**	**1**	**CGL2_11390001**	Putative lytic transglycosylase	185	163..717	1108_GENE_3
**11390**	**2**	**CGL2_11390002**	Hypothetical protein	298	840..1733	483_GENE_1
**11390**	**3**	**CGL2_11390003**	Dihydrolipoamide dehydrogenase (EC 1.8.1.4)	461	complement(1764..3146)	483_GENE_2
**11390**	**4**	**CGL2_11390004**	Putative diguanylate cyclase/phosphodiesterase with	1157	complement(3277..6747)	483_GENE_3
**11390**	**5**	**CGL2_11390005**	Glutamine synthetase, type I (EC 6.3.1.2)	470	7377..8786	23_GENE_3
**11390**	**6**	**CGL2_11390006**	Aspartyl-tRNA synthetase (EC 6.1.1.12)	584	complement(8907..10658)	23_GENE_4
**11390**	**7**	**CGL2_11390007**	Pseudouridine synthase, Rsu	583	complement(10673..12421)	23_GENE_4a
**11390**	**8**	**CGL2_11390008**	GMP synthase [glutamine-hydrolyzing] (EC 6.3.5.2)	516	complement(12427..13974)	23_GENE_6
**11390**	**9**	**CGL2_11390009**	Inosine-5'-monophosphate dehydrogenase (EC 1.1.1	489	complement(14017..15483)	23_GENE_7
**11390**	**10**	**CGL2_11390010**	Conserved protein of unknown function	173	complement(15583..16101)	23_GENE_8
**11390**	**11**	**CGL2_11390011**	Probable AAA ATPase superfamily	300	complement(16097..16996)	23_GENE_9
**11390**	**12**	**CGL2_11390012**	Putative Lon family ATP-dependent protease	218	complement(17020..17673)	23_GENE_10
**11390**	**13**	**CGL2_11390013**	Protein of unknown function	133	complement(17703..18101)	23_GENE_11
**11390**	**14**	**CGL2_11390014**	Probable N-acetyltransferase family protein	167	18242..18742	23_GENE_12

**11390**	**15**	**CGL2_11390015**	DNA ligase (EC 6.5.1.1)	603	complement(18784..20592)	23_GENE_13
**11390**	**16**	**CGL2_11390016**	Hypothetical protein	155	20772..21236	23_GENE_14
**11390**	**17**	**CGL2_11390017**	Protein of unknown function	179	complement(21286..21822)	23_GENE_15
**11390**	**18**	**CGL2_11390018**	Apolipoprotein N-acyltransferase	491	22155..23627	23_GENE_16
**11390**	**19**	**CGL2_11390019**	Peptide chain release factor 2	370	23667..24776	23_GENE_17
**11390**	**20**	**CGL2_11390020**	Putative heat shock protein (Hsp20)	147	24833..25273	23_GENE_18
**11390**	**21**	**CGL2_11390021**	ATP-dependent protease La (EC 3.4.21.53)	813	25269..27707	23_GENE_19
**11390**	**22**	**CGL2_11390022**	Endonuclease III/Nth	241	27652..28374	23_GENE_20
**11390**	**23**	**CGL2_11390023**	Conserved hypothetical protein	69	28518..28724	23_GENE_21
**11390**	**24**	**CGL2_11390024**	Aldo/keto reductase (EC 1.1.1.65)	335	complement(28761..29765)	23_GENE_22
**11390**	**25**	**CGL2_11390025**	Putative transcriptional regulator, AraC family	315	29953..30897	23_GENE_23
**11390**	**26**	**CGL2_11390026**	Putative glycoside hydrolase, family 15	684	complement(30969..33020)	23_GENE_24
**11390**	**28**	**CGL2_11390028**	Histidyl-tRNA synthetase (EC 6.1.1.21)	437	complement(33041..34351)	23_GENE_25
**11390**	**29**	**CGL2_11390029**	Putative glycoside hydrolase, family 15	659	34579..36555	23_GENE_27
**11390**	**31**	**CGL2_11390031**	GTP-binding protein (lepA)	609	37020..38846	23_GENE_28
**11390**	**32**	**CGL2_11390032**	Signal peptidase I (EC 3.4.21.89)	223	38891..39559	23_GENE_29
**11390**	**33**	**CGL2_11390033**	Carbohydrate kinase, PfkB	352	39587..40642	23_GENE_30
**11390**	**34**	**CGL2_11390034**	3-deoxy-D-manno-octulosonate cytidylyltransferase	277	40642..41472	23_GENE_31
**11390**	**35**	**CGL2_11390035**	CTP synthase (EC 6.3.4.2)	545	41463..43097	23_GENE_32
**11390**	**36**	**CGL2_11390036**	2-Dehydro-3-deoxyphosphooctonate aldolase (EC 4.:	289	43097..43963	23_GENE_33
**11390**	**37**	**CGL2_11390037**	Sugar isomerase, KpsF/GutQ family (EC 5.3.1.13)	332	43963..44958	23_GENE_34
**11390**	**38**	**CGL2_11390038**	Phosphatase kdsC	182	44961..45506	23_GENE_35
**11390**	**39**	**CGL2_11390039**	CDP-diacylglycerol--glycerol-3-phosphate 3-phospha	189	45506..46072	23_GENE_36
**11390**	**40**	**CGL2_11390040**	Conserved hypothetical protein	206	46092..46709	23_GENE_37
**11390**	**41**	**CGL2_11390041**	Putative peptidase, M23B family	327	46855..47835	23_GENE_39
**11390**	**42**	**CGL2_11390042**	Putative periplasmic sensor signal transduction histid	506	47955..49472	23_GENE_40
**11390**	**43**	**CGL2_11390043**	Protein of unknown function	176	49472..49999	23_GENE_41
**11390**	**44**	**CGL2_11390044**	Putative two component, sigma54 specific, transcript	458	49999..51372	23_GENE_42
**11390**	**45**	**CGL2_11390045**	Conserved hypothetical protein	256	complement(51412..52179)	23_GENE_43
**11390**	**46**	**CGL2_11390046**	Hypothetical protein	359	complement(52217..53293)	23_GENE_44
**11390**	**47**	**CGL2_11390047**	Conserved protein of unknown function	327	complement(53375..54355)	23_GENE_45
**11390**	**48**	**CGL2_11390048**	Protein of unknown function	102	54439..54744	23_GENE_46
**11390**	**49**	**CGL2_11390049**	Putative phosphoesterase, PAP2 family	211	54832..55464	23_GENE_47
**11390**	**50**	**CGL2_11390050**	Putative radical SAM family protein	379	55442..56578	23_GENE_48
**11390**	**51**	**CGL2_11390051**	Hypothetical protein	105	56548..56862	23_GENE_49
**11390**	**52**	**CGL2_11390052**	Putative catalytic LigB subunit of aromatic ring-openi	264	complement(56888..57679)	23_GENE_50
**11390**	**53**	**CGL2_11390053**	Probable methyltransferase	388	complement(57735..58898)	23_GENE_51
**11390**	**54**	**CGL2_11390054**	Hypothetical protein	77	complement(59023..59253)	23_GENE_52
**11390**	**55**	**CGL2_11390055**	Putative cytochrome c oxidase, subunit I	479	59439..60875	33_GENE_1
**11390**	**56**	**CGL2_11390056**	Twin-arginine translocation protein TatA/E	73	61085..61303	33_GENE_4
**11390**	**57**	**CGL2_11390057**	Protein of unknown function	143	complement(61360..61788)	33_GENE_5

**11390**	**58**	**CGL2_11390058**	Putative FAD dependent oxidoreductase	413	complement(61788..63026)	33_GENE_6
**11390**	**60**	**CGL2_11390060**	Conserved hypothetical protein	303	complement(63128..64036)	33_GENE_7
**11390**	**61**	**CGL2_11390061**	NADH-flavin oxidoreductase/NADH oxidase	354	complement(64103..65164)	33_GENE_8
**11390**	**62**	**CGL2_11390062**	Putative major facilitator superfamily transporter	496	complement(65312..66799)	33_GENE_9
**11390**	**63**	**CGL2_11390063**	Hypothetical protein	85	complement(66799..67053)	33_GENE_10
**11390**	**64**	**CGL2_11390064**	Protein of unknown function	420	complement(67237..68496)	33_GENE_11
**11390**	**65**	**CGL2_11390065**	Conserved hypothetical protein	195	68836..69420	33_GENE_12
**11390**	**66**	**CGL2_11390066**	Protein of unknown function	273	69648..70466	33_GENE_14
**11390**	**67**	**CGL2_11390067**	Probable PAS/PAC sensor protein	218	70493..71146	33_GENE_15
**11390**	**68**	**CGL2_11390068**	Diguanylate cyclase	426	71221..72498	33_GENE_16
**11390**	**70**	**CGL2_11390070**	Hypothetical protein	258	72781..73554	33_GENE_17
**11390**	**71**	**CGL2_11390071**	Hypothetical protein	147	complement(73698..74138)	33_GENE_18
**11390**	**72**	**CGL2_11390072**	Protein of unknown function	345	74395..75429	33_GENE_19
**11390**	**73**	**CGL2_11390073**	Protein of unknown function	493	75429..76907	33_GENE_20
**11390**	**74**	**CGL2_11390074**	Putative nodulation efficiency protein D	465	77161..78555	33_GENE_21
**11390**	**75**	**CGL2_11390075**	Band 7 family protein	252	78555..79310	33_GENE_22
**11390**	**76**	**CGL2_11390076**	Putative carboxymuconolactone decarboxylase	146	complement(79331..79768)	33_GENE_23
**11390**	**77**	**CGL2_11390077**	Protein of unknown function	175	complement(79768..80292)	33_GENE_24
**11390**	**78**	**CGL2_11390078**	Putative 3-hydroxyisobutyrate dehydrogenas	308	80505..81428	33_GENE_25
**11390**	**79**	**CGL2_11390079**	Probable hydrolase	277	81424..82254	33_GENE_26
**11390**	**79**	**CGL2_11390079**	Probable hydrolase	277	81424..82254	33_GENE_26
**11390**	**81**	**CGL2_11390081**	Protein of unknown function	150	complement(82423..82872)	33_GENE_27
**11390**	**82**	**CGL2_11390082**	Hypothetical protein	126	complement(82968..83345)	33_GENE_28
**11390**	**83**	**CGL2_11390083**	Hypothetical protein	101	83398..83700	33_GENE_29
**11390**	**84**	**CGL2_11390084**	Putative two component, sigma54 specific, transcript	489	complement(83935..85401)	33_GENE_30
**11390**	**85**	**CGL2_11390085**	Multi-sensor signal transduction histidine kinase	660	complement(85401..87380)	33_GENE_31
**11390**	**86**	**CGL2_11390086**	Conserved protein of unknown function	826	complement(87548..90025)	33_GENE_32
**11390**	**87**	**CGL2_11390087**	NADH dehydrogenase (Quinone) (EC 1.6.99.5)	555	complement(90025..91689)	33_GENE_33
**11390**	**88**	**CGL2_11390088**	Hypothetical protein	112	91972..92307	33_GENE_33a
**11390**	**89**	**CGL2_11390089**	Dihydroxy-acid dehydratase (EC 4.2.1.9)	561	92432..94114	33_GENE_36
**11390**	**90**	**CGL2_11390090**	SUA5/yciO/yrdC family	339	complement(94206..95222)	33_GENE_37
**11390**	**91**	**CGL2_11390091**	Dihydroxy-acid dehydratase (EC 4.2.1.9)	558	complement(95296..96969)	33_GENE_38
**11390**	**92**	**CGL2_11390092**	Conserved protein of unknown function	288	complement(97064..97927)	33_GENE_39
**11390**	**93**	**CGL2_11390093**	Conserved protein of unknown function	823	complement(98122..100590)	33_GENE_41
**11390**	**94**	**CGL2_11390094**	NADH dehydrogenase (Quinone) (EC 1.6.99.5)	553	complement(100590..102248)	33_GENE_42
**11390**	**95**	**CGL2_11390095**	Hypothetical protein	99	102681..102977	33_GENE_42a
**11390**	**96**	**CGL2_11390096**	Probable PilT protein-like	105	complement(103189..103503)	33_GENE_44
**11390**	**97**	**CGL2_11390097**	Probable transcriptional regulator (AbrB)	88	complement(103503..103766)	33_GENE_45
**11390**	**98**	**CGL2_11390098**	Putative glycosyltransferase	362	104057..105142	33_GENE_47
**11390**	**98**	**CGL2_11390098**	Putative glycosyltransferase	362	104057..105142	33_GENE_47
**11390**	**100**	**CGL2_11390100**	Putative glycosyl transferase, group 1	401	105224..106426	33_GENE_48

**11390**	**101**	**CGL2_11390101**	Undecaprenyl-phosphate galactose phosphotransfera	503	106419..107927	369_GENE_7
**11390**	**102**	**CGL2_11390102**	ABC transporter permease protein	264	107969..108760	369_GENE_8
**11390**	**103**	**CGL2_11390103**	ABC transporter ATP-binding protein	181	108773..109315	369_GENE_9
**11390**	**104**	**CGL2_11390104**	Putative glycosyltransferase	380	110083..111222	
**9490**	**1**	**CGL2_09490001**	Hypothetical protein	116	674..1021	318_GENE_1
**9490**	**2**	**CGL2_09490002**	Conserved hypothetical protein	87	1283..1543	318_GENE_2
**9490**	**3**	**CGL2_09490003**	Probable CopG family protein	79	1536..1772	318_GENE_3
**9490**	**3**	**CGL2_09490003**	Probable CopG family protein	79	1536..1772	318_GENE_3
**9490**	**5**	**CGL2_09490005**	Probable transposase	220	complement(2157..2816)	318_GENE_4
**9490**	**5**	**CGL2_09490005**	Probable transposase	220	complement(2157..2816)	318_GENE_4
**9921**	**4**	**CGL2_09921004**	Methylated-DNA-[protein]-cysteine S-methyltransfer	164	3045..3536	318_GENE_5
**9921**	**3**	**CGL2_09921003**	Putative Ada DNA repair protein and transcriptional r	475	1598..3022	318_GENE_6
**9921**	**2**	**CGL2_09921002**	Protein of unknown function	108	1087..1410	318_GENE_7
**9921**	**1**	**CGL2_09921001**	Hypothetical protein	142	412..837	318_GENE_8
**11284**	**2**	**CGL2_11284002**	Putative transposase	182	complement(598..1143)	318_GENE_9
**11284**	**3**	**CGL2_11284003**	Probable monooxygenase	420	complement(1470..2729)	191_GENE_2
**11284**	**4**	**CGL2_11284004**	Protein of unknown function	585	complement(2729..4483)	191_GENE_3
**11284**	**5**	**CGL2_11284005**	Glucose-6-phosphate 1-dehydrogenase (EC 1.1.1.49	495	complement(4498..5982)	191_GENE_4
**11284**	**6**	**CGL2_11284006**	Transaldolase	974	complement(6319..9240)	191_GENE_5
**11284**	**7**	**CGL2_11284007**	Transketolase (EC 2.2.1.1)	475	complement(9296..10720)	191_GENE_6
**9975**	**1**	**CGL2_09975001**	6-phosphogluconolactonase (EC 3.1.1.31)	19	complement(41..97)	511_GENE_9
**9975**	**2**	**CGL2_09975002**	Glucokinase (EC 2.7.1.2)	347	complement(348..1388)	511_GENE_8
**9975**	**3**	**CGL2_09975003**	Hypothetical protein	92	complement(1500..1775)	511_GENE_7
**9975**	**4**	**CGL2_09975004**	Hypothetical protein	61	complement(1796..1978)	511_GENE_5a
**9975**	**5**	**CGL2_09975005**	Protein of unknown function	185	complement(2110..2664)	511_GENE_5
**9975**	**6**	**CGL2_09975006**	Protein of unknown function	492	complement(2682..4157)	511_GENE_4
**9975**	**7**	**CGL2_09975007**	Protein of unknown function	487	complement(4360..5820)	511_GENE_2
**10139**	**1**	**CGL2_10139001**	Conserved protein of unknown function	241	complement(44..766)	736_GENE_4
**10139**	**2**	**CGL2_10139002**	DNA/RNA helicase, SNF2 family	263	complement(766..1554)	736_GENE_2
**10139**	**3**	**CGL2_10139003**	DNA/RNA helicase, SNF2 family	631	complement(1520..3412)	736_GENE_2
**10139**	**4**	**CGL2_10139004**	Probable transposase	458	3592..4965	412_GENE_6
**10139**	**5**	**CGL2_10139005**	Transposase	352	complement(5307..6362)	412_GENE_5
**10139**	**7**	**CGL2_10139007**	Diguanylate cyclase/phosphodiesterase with PAS/PA(	250	6459..7208	412_GENE_4
**10139**	**7**	**CGL2_10139007**	Diguanylate cyclase/phosphodiesterase with PAS/PA(	250	6459..7208	412_GENE_4
**6867**	**4**	**CGL2_06867004**	RNA-directed DNA polymerase (EC 2.7.7.49)	195	complement(2032..2616)	NOTE: see end of file-
**6867**	**3**	**CGL2_06867003**	Hypothetical protein	155	complement(1166..1630)	
**6867**	**2**	**CGL2_06867002**	Hypothetical protein	213	complement(116..754)	
**11284**	**8**	**CGL2_11284008**	Putative transposase	146	10780..11217	191_GENE_7
**11284**	**9**	**CGL2_11284009**	RNA-directed DNA polymerase (EC 2.7.7.49)	501	11597..13099	73_GENE_33
**11284**	**10**	**CGL2_11284010**	Probable nucleotidyltransferase superfamily protein	99	complement(13299..13595)	gap between gene 73_
**11284**	**10**	**CGL2_11284010**	Probable nucleotidyltransferase superfamily protein	99	complement(13299..13595)	73_GENE_31

**11284**	**12**	**CGL2_11284012**	Hypothetical protein	129	complement(13774..14160)	73_GENE_29a
**11284**	**13**	**CGL2_11284013**	Conserved protein of unknown function	502	14459..15964	73_GENE_29
**11284**	**14**	**CGL2_11284014**	3-hydroxyisobutyrate dehydrogenase (EC 1.1.1.31)	311	16469..17401	73_GENE_27
**11284**	**15**	**CGL2_11284015**	Probable alpha/beta fold family protein	268	17401..18204	73_GENE_26
**11284**	**16**	**CGL2_11284016**	Conserved protein of unknown function	157	18365..18835	73_GENE_25
**11284**	**17**	**CGL2_11284017**	Protein of unknown function	85	complement(18949..19203)	73_GENE_23a
**11284**	**18**	**CGL2_11284018**	Hypothetical protein	85	complement(19362..19616)	73_GENE_23
**11284**	**19**	**CGL2_11284019**	Putative transposase	290	complement(19609..20478)	73_GENE_22
**11284**	**20**	**CGL2_11284020**	Putative transposase	96	complement(20451..20738)	73_GENE_21
**11284**	**21**	**CGL2_11284021**	Hypothetical protein	66	20895..21092	73_GENE_19
**11284**	**22**	**CGL2_11284022**	Probable PilT protein, N-terminal	128	complement(21183..21566)	73_GENE_18
**11284**	**23**	**CGL2_11284023**	Prevent-host-death family protein	79	complement(21624..21860)	73_GENE_17
**11284**	**24**	**CGL2_11284024**	Hypothetical protein	96	complement(21878..22165)	73_GENE_16
**11284**	**25**	**CGL2_11284025**	Plasmid maintenance system antidote protein, XRE fa	96	complement(22443..22730)	73_GENE_14
**11284**	**26**	**CGL2_11284026**	Plasmid maintenance system killer	93	complement(22749..23027)	73_GENE_13
**11284**	**27**	**CGL2_11284027**	Probable transposase	160	23491..23970	73_GENE_12
**11284**	**28**	**CGL2_11284028**	Probable transposase	170	24030..24539	73_GENE_11
**11284**	**29**	**CGL2_11284029**	Probable ATPase protein	159	24945..25421	73_GENE_9
**11284**	**30**	**CGL2_11284030**	Transposase	380	25456..26595	73_GENE_8
**11284**	**31**	**CGL2_11284031**	ATPase	135	26674..27078	73_GENE_7
**11284**	**32**	**CGL2_11284032**	Hypothetical protein	82	complement(27392..27637)	73_GENE_6
**11284**	**33**	**CGL2_11284033**	Probable transposase	335	complement(27738..28742)	73_GENE_5
**11284**	**34**	**CGL2_11284034**	Putative glycosyl transferase, group 1	357	29579..30649	73_GENE_4
**11284**	**35**	**CGL2_11284035**	Putative glycosyl transferase, group 1	431	30699..31991	627_GENE_2
**11284**	**36**	**CGL2_11284036**	Undecaprenyl-phosphate galactose phosphotransfera	502	31991..33496	73_GENE_1
**11284**	**37**	**CGL2_11284037**	Putative ABC transporter permease protein	229	33526..34212	627_GENE_4
**11284**	**40**	**CGL2_11284040**	Teichoic-acid-transporting ATPase (EC 3.6.3.40)	201	34442..35044	955_GENE_4
**11284**	**42**	**CGL2_11284042**	Probable transposase	133	35177..35575	659_GENE_7
**11284**	**43**	**CGL2_11284043**	Transposase	314	complement(35582..36523)	659_GENE_6
**11284**	**44**	**CGL2_11284044**	Probable transposase	180	36657..37196	659_GENE_5
**11284**	**46**	**CGL2_11284046**	Conserved hypothetical protein	189	complement(37491..38057)	659_GENE_3
**11284**	**47**	**CGL2_11284047**	Hypothetical protein	109	complement(38060..38386)	659_GENE_2
**11284**	**48**	**CGL2_11284048**	Putative glycosyl transferase, family 2	568	38669..40372	659_GENE_1
**11284**	**49**	**CGL2_11284049**	Putative glycosyl transferase, family 2	1023	40459..43527	
**11284**	**50**	**CGL2_11284050**	Conserved hypothetical protein	441	complement(43627..44949)	
**10601**	**1**	**CGL2_10601001**	Conserved hypothetical protein	50	complement(312..462)	985_GENE_3
**10601**	**2**	**CGL2_10601002**	Hypothetical protein	86	787..1044	985_GENE_4
**10601**	**3**	**CGL2_10601003**	Putative metallo-beta-lactamase family protein	243	1234..1962	178_GENE_1
**10601**	**4**	**CGL2_10601004**	Probable transcriptional regulator, ArsR family	119	1988..2344	178_GENE_2
**10601**	**5**	**CGL2_10601005**	Putative major facilitator superfamily transporter	442	2344..3669	178_GENE_3
**10601**	**6**	**CGL2_10601006**	Hypothetical protein	53	complement(3900..4058)	178_GENE_4

**10601**	**7**	**CGL2_10601007**	Hypothetical protein	76	4109..4336	178_GENE_4a
**10601**	**8**	**CGL2_10601008**	Hypothetical protein	152	4463..4918	178_GENE_5
**10601**	**9**	**CGL2_10601009**	Probable outer membrane efflux protein	481	4994..6436	178_GENE_6
**10601**	**10**	**CGL2_10601010**	Secretion protein (HlyD)	419	6429..7685	178_GENE_7
**10601**	**11**	**CGL2_10601011**	Heavy metal efflux pump, CzcA family	1061	7685..10867	178_GENE_8
**10601**	**12**	**CGL2_10601012**	Conserved protein of unknown function	134	10885..11286	178_GENE_9
**10799**	**26**	**CGL2_10799026**	Hypothetical protein	479	complement(18611..20047)	178_GENE_10
**10799**	**25**	**CGL2_10799025**	Putative IstB-like ATP-binding protein	260	17590..18369	321_GENE_1
**10799**	**24**	**CGL2_10799024**	Probable transposase	284	16742..17593	321_GENE_2
**10799**	**23**	**CGL2_10799023**	Putative transposase	351	15580..16632	321_GENE_3
**10799**	**22**	**CGL2_10799022**	Probable transposase	175	complement(14810..15334)	321_GENE_4
**10799**	**21**	**CGL2_10799021**	Probable transposase	489	complement(13150..14616)	321_GENE_5
**10799**	**20**	**CGL2_10799020**	Putative IstB-like ATP-binding protein	252	complement(12395..13150)	321_GENE_6
**10799**	**19**	**CGL2_10799019**	Hypothetical protein	70	complement(12159..12368)	321_GENE_7
**10799**	**18**	**CGL2_10799018**	Putative transposase	384	complement(10981..12132)	321_GENE_8
**10799**	**17**	**CGL2_10799017**	Putative IstB-like ATP-binding protein	292	complement(10133..11008)	581_GENE_8
**10799**	**16**	**CGL2_10799016**	Probable transposase	161	9670..10152	
**10799**	**15**	**CGL2_10799015**	Conserved protein of unknown function	360	8366..9445	45_GENE_46
**10799**	**14**	**CGL2_10799014**	Protein of unknown function	120	7891..8250	45_GENE_45a
**10799**	**13**	**CGL2_10799013**	Putative transcriptional regulator, TetR family	219	7222..7878	45_GENE_45
**10799**	**12**	**CGL2_10799012**	Conserved hypothetical protein	117	complement(6469..6819)	45_GENE_43
**10799**	**11**	**CGL2_10799011**	Putative metal dependent phosphohydrolase	254	complement(5091..5852)	45_GENE_41
**10799**	**10**	**CGL2_10799010**	Conserved protein of unknown function	188	3936..4499	45_GENE_40
**10799**	**9**	**CGL2_10799009**	Hypothetical protein	79	3570..3806	45_GENE_39
**10799**	**8**	**CGL2_10799008**	Probable transposase	105	complement(3021..3335)	45_GENE_38
**10799**	**7**	**CGL2_10799007**	Probable transposase	115	complement(2658..3002)	45_GENE_37
**10799**	**6**	**CGL2_10799006**	Conserved hypothetical protein	62	complement(2394..2579)	45_GENE_36
**10799**	**5**	**CGL2_10799005**	Putative PilT protein, N-terminal	140	1946..2365	45_GENE_35
**10799**	**4**	**CGL2_10799004**	Probable plasmid stability protein	84	1695..1946	45_GENE_34
**10799**	**3**	**CGL2_10799003**	Conserved protein of unknown function	165	948..1442	45_GENE_33
**10799**	**2**	**CGL2_10799002**	Conserved hypothetical protein	137	578..988	45_GENE_32
**10799**	**1**	**CGL2_10799001**	Probable PilT protein, N-terminal	133	172..570	45_GENE_31
**11391**	**30**	**CGL2_11391030**	Prevent-host-death protein	81	26362..26604	45_GENE_30
**11391**	**29**	**CGL2_11391029**	Hypothetical protein	49	complement(26133..26279)	45_GENE_29
**11391**	**28**	**CGL2_11391028**	Conserved hypothetical protein	92	complement(25809..26084)	45_GENE_28
**11391**	**27**	**CGL2_11391027**	Conserved hypothetical protein	129	complement(25445..25831)	
**11391**	**26**	**CGL2_11391026**	Hypothetical protein	77	25000..25230	45_GENE_27
**11391**	**25**	**CGL2_11391025**	Protein of unknown function	119	24550..24906	45_GENE_26
**11391**	**24**	**CGL2_11391024**	Probable cyclic nucleotide-binding protein	249	23259..24005	45_GENE_25
**11391**	**23**	**CGL2_11391023**	Conserved hypothetical protein	428	complement(21577..22860)	45_GENE_24
**11391**	**22**	**CGL2_11391022**	Putative NADH ubiquinone oxidoreductase, 20 kDa su	169	21041..21547	45_GENE_23

**11391**	**21**	**CGL2_11391021**	Putative NADH-ubiquinone oxidoreductase, 49 kDa s	499	19523..21019	45_GENE_22
**11391**	**20**	**CGL2_11391020**	NADH dehydrogenase (Quinone) (EC 1.6.99.5)	485	17998..19452	45_GENE_21
**11391**	**19**	**CGL2_11391019**	Probable hydrogenase 4 membrane component	220	17331..17990	45_GENE_20
**11391**	**18**	**CGL2_11391018**	Putative formate hydrogenlyase subunit 4	320	16368..17327	45_GENE_19
**11391**	**17**	**CGL2_11391017**	NADH dehydrogenase (Quinone) (EC 1.6.99.5)	686	14300..16357	45_GENE_18
**11391**	**16**	**CGL2_11391016**	Plasmid maintenance system antidote protein, XRE fa	116	13943..14290	45_GENE_17
**11391**	**15**	**CGL2_11391015**	Hypothetical protein	297	12918..13808	45_GENE_16
**11391**	**14**	**CGL2_11391014**	Conserved protein of unknown function	218	12108..12761	45_GENE_15
**11391**	**13**	**CGL2_11391013**	Conserved protein of unknown function	483	complement(10047..11495)	45_GENE_13
**11391**	**12**	**CGL2_11391012**	Conserved protein of unknown function	358	complement(8989..10062)	45_GENE_12
**11391**	**11**	**CGL2_11391011**	Conserved protein of unknown function	251	complement(8014..8766)	45_GENE_11
**11391**	**10**	**CGL2_11391010**	DNA-3-methyladenine glycosylase (EC 3.2.2.21)	211	complement(7382..8014)	45_GENE_10
**11391**	**9**	**CGL2_11391009**	Protein of unknown function	137	complement(6852..7262)	45_GENE_9
**11391**	**8**	**CGL2_11391008**	Conserved hypothetical protein	211	complement(5782..6414)	45_GENE_8
**11391**	**7**	**CGL2_11391007**	Conserved protein of unknown function	173	complement(5140..5658)	45_GENE_7
**11391**	**6**	**CGL2_11391006**	Conserved hypothetical protein	363	complement(3967..5055)	45_GENE_6
**11391**	**5**	**CGL2_11391005**	Methylated-DNA-[protein]-cysteine S-methyltransfer	164	3485..3976	45_GENE_5
**11391**	**4**	**CGL2_11391004**	Putative Ada DNA repair protein and transcriptional r	477	2055..3485	45_GENE_4
**11391**	**3**	**CGL2_11391003**	Aldo/keto reductase (EC 1.1.1.91)	357	complement(824..1894)	45_GENE_3
**11391**	**2**	**CGL2_11391002**	Hypothetical protein	97	496..786	45_GENE_2
**11391**	**1**	**CGL2_11391001**	Protein of unknown function	112	44..379	45_GENE_1
**9831**	**1**	**CGL2_09831001**	Conserved hypothetical protein	333	370..1368	454_GENE_3
**9831**	**2**	**CGL2_09831002**	Hypothetical protein	44	1721..1852	454_GENE_4
**9831**	**3**	**CGL2_09831003**	Hypothetical protein	53	complement(2219..2377)	454_GENE_5
**9831**	**4**	**CGL2_09831004**	Methyl-accepting chemotaxis sensory transducer witl	231	complement(2591..3283)	454_GENE_6
**9831**	**5**	**CGL2_09831005**	transposage	140	3291..3710	454_GENE_8
**11278**	**42**	**CGL2_11278042**	Putative methyl-accepting chemotaxis protein	247	29709..30449	81_GENE_5
**11278**	**41**	**CGL2_11278041**	Hypothetical protein	77	complement(29322..29552)	81_GENE_6
**11278**	**40**	**CGL2_11278040**	Hypothetical protein	142	28412..28837	81_GENE_7
**11278**	**39**	**CGL2_11278039**	Hypothetical protein	152	27954..28409	81_GENE_8
**11278**	**38**	**CGL2_11278038**	Hypothetical protein	187	complement(26885..27445)	81_GENE_9
**11278**	**37**	**CGL2_11278037**	Hypothetical protein	50	26513..26662	81_GENE_10
**11278**	**36**	**CGL2_11278036**	Hypothetical protein	71	complement(26146..26358)	81_GENE_11
**11278**	**35**	**CGL2_11278035**	Hypothetical protein	86	25771..26028	81_GENE_12
**11278**	**33**	**CGL2_11278033**	Putative phage integrase	106	25429..25746	81_GENE_14
**11278**	**32**	**CGL2_11278032**	Putative phage integrase	248	24690..25433	81_GENE_14
**11278**	**30**	**CGL2_11278030**	Hypothetical protein	108	24045..24368	75_GENE_30
**11278**	**29**	**CGL2_11278029**	Hypothetical protein	72	complement(23675..23890)	75_GENE_28a
**11278**	**28**	**CGL2_11278028**	Putative phage protein Gp37/Gp68	240	22928..23647	75_GENE_28
**11278**	**27**	**CGL2_11278027**	Conserved hypothetical protein	371	21809..22921	75_GENE_27
**11278**	**26**	**CGL2_11278026**	Protein of unknown function	149	21363..21809	75_GENE_26

**11278**	**25**	**CGL2_11278025**	Hypothetical protein	291	20467..21339	75_GENE_25
**11278**	**24**	**CGL2_11278024**	Hypothetical protein	133	20069..20467	75_GENE_24
**11278**	**23**	**CGL2_11278023**	Probable O-methyltransferase	199	19442..20038	75_GENE_23
**11278**	**22**	**CGL2_11278022**	Conserved hypothetical protein	224	18465..19136	75_GENE_22
**11278**	**21**	**CGL2_11278021**	Conserved hypothetical protein	162	complement(17717..18202)	75_GENE_21
**11278**	**20**	**CGL2_11278020**	Putative metallo-beta-lactamase family protein	259	complement(16938..17714)	75_GENE_20
**11278**	**19**	**CGL2_11278019**	Diguanylate cyclase/phosphodiesterase	720	complement(14778..16937)	75_GENE_19
**11278**	**17**	**CGL2_11278017**	Probable methyl-accepting chemotaxis sensory trans	275	complement(13712..14536)	75_GENE_18
**11278**	**16**	**CGL2_11278016**	Probable methyl-accepting chemotaxis sensory trans	119	complement(13340..13696)	75_GENE_17
**11278**	**15**	**CGL2_11278015**	Putative metallo-beta-lactamase family protein	248	complement(12560..13303)	75_GENE_16
**11278**	**14**	**CGL2_11278014**	Probable methyl-accepting chemotaxis sensory trans	232	complement(11849..12544)	75_GENE_15
**11278**	**13**	**CGL2_11278013**	Hypothetical protein	47	complement(11102..11242)	75_GENE_13
**11278**	**12**	**CGL2_11278012**	Probable transposase	87	complement(10666..10926)	75_GENE_12
**11278**	**11**	**CGL2_11278011**	Conserved hypothetical protein	139	10163..10579	75_GENE_11
**11278**	**10**	**CGL2_11278010**	Hypothetical protein	210	9465..10094	75_GENE_10
**11278**	**9**	**CGL2_11278009**	Probable transposase	56	complement(9086..9253)	75_GENE_9
**11278**	**8**	**CGL2_11278008**	Putative O-methyltransferase, family 3	236	8314..9021	75_GENE_8
**11278**	**7**	**CGL2_11278007**	Conserved hypothetical protein	231	7529..8221	75_GENE_7
**11278**	**6**	**CGL2_11278006**	Conserved hypothetical protein	104	7166..7477	75_GENE_6
**11278**	**5**	**CGL2_11278005**	Hypothetical protein	57	6719..6889	
**11278**	**4**	**CGL2_11278004**	Protein of unknown function	176	6107..6634	75_GENE_5
**11278**	**3**	**CGL2_11278003**	DNA helicase	1602	1228..6033	75_GENE_4
**11278**	**2**	**CGL2_11278002**	Putative putative phage repressor	224	320..991	75_GENE_2
**11278**	**1**	**CGL2_11278001**	Hypothetical protein	72	complement(18..233)	75_GENE_1
**4377**	**1**	**CGL2_04377001**	Hypothetical protein	204	557..1168	
**4377**	**2**	**CGL2_04377002**	Conserved hypothetical protein	60	1175..1354	
**2657**	**1**	**CGL2_02657001**	Protein of unknown function	104	88..399	
02657tRNA		**CGL2_02657R001**	TRNA Ser CGA		587..677	
**2657**	**2**	**CGL2_02657002**	Hypothetical protein	60	732..911	376_GENE_1a
**7140**	**1**	**CGL2_07140001**	Hypothetical protein	79	70..306	376_GENE_1
**7140**	**2**	**CGL2_07140002**	Conserved hypothetical protein	98	773..1066	376_GENE_3
**8598**	**2**	**CGL2_08598002**	Hypothetical protein	192	complement(1181..1756)	376_GENE_5
**8598**	**1**	**CGL2_08598001**	Hypothetical protein	123	907..1275	376_GENE_6
**8982**	**7**	**CGL2_08982007**	Hypothetical protein	34	complement(3294..3395)	376_GENE_7
**8982**	**6**	**CGL2_08982006**	Hypothetical protein	61	complement(3112..3294)	376_GENE_8
**8982**	**5**	**CGL2_08982005**	Hypothetical protein	64	complement(2928..3119)	376_GENE_9
**8982**	**4a**	**CGL2_08982004a**	Hypothetical protein	84	complement(2680..2928)	
**8982**	**4**	**CGL2_08982004**	Hypothetical protein	120	complement(2330..2689)	376_GENE_10
**8982**	**3**	**CGL2_08982003**	Putative phage replisome organizer, N-terminal regic	328	complement(1279..2262)	376_GENE_11
**8982**	**2**	**CGL2_08982002**	Hypothetical protein	181	complement(731..1273)	376_GENE_12
**8982**	**1**	**CGL2_08982001**	Hypothetical protein	62	complement(395..580)	376_GENE_12a

**8581**	**3**	**CGL2_08581003**	Hypothetical protein	541	complement(2408..4030)	376_GENE_14
**8581**	**2**	**CGL2_08581002**	Hypothetical protein	427	complement(1092..2372)	376_GENE_15
**8581**	**1**	**CGL2_08581001**	Hypothetical protein	53	complement(931..1089)	
**5939**	**1**	**CGL2_05939001**	Hypothetical protein	88	complement(458..721)	
**5939**	**2**	**CGL2_05939002**	Hypothetical protein	175	complement(736..1260)	
**10965**	**24**	**CGL2_10965024**	Putative adenylylsulfate reductase, subunit A	524	complement(18938..20509)	303_GENE_1
**10965**	**23**	**CGL2_10965023**	Probable adenylylsulfate reductase, subunit B	114	complement(18610..18951)	303_GENE_2
**10965**	**22**	**CGL2_10965022**	Sulfate adenylyltransferase (EC 2.7.7.4)	393	complement(17359..18537)	303_GENE_3
**10965**	**21**	**CGL2_10965021**	Putative transcriptional regulator, XRE family	121	16859..17221	303_GENE_4
**10965**	**20**	**CGL2_10965020**	Conserved hypothetical protein	64	complement(16608..16799)	303_GENE_5
**10965**	**19**	**CGL2_10965019**	Putative radical SAM superfamily transporter	445	complement(14911..16245)	303_GENE_6
**10965**	**18**	**CGL2_10965018**	Putative transport protein	506	complement(13352..14869)	303_GENE_7
**10965**	**17**	**CGL2_10965017**	Hypothetical protein	65	complement(13158..13352)	303_GENE_9
10965tRNA		**CGL2_10965R001**	TRNA Met CAT		complement(13089..13164)	
**10965**	**16**	**CGL2_10965016**	transposase	476	11276..12703	303_GENE_10
**7414**	**1**	**CGL2_07414001**	Hypothetical protein	130	262..651	539_GENE_9
07414tRNA		**CGL2_07414R001**	TRNA Met CAT		55..130	
**7414**	**2**	**CGL2_07414002**	Hypothetical protein	149	complement(730..1176)	539_GENE_8
**7414**	**3**	**CGL2_07414003**	Hypothetical protein	91	complement(1179..1451)	539_GENE_7
**9479**	**2**	**CGL2_09479002**	Hypothetical protein	134	2537..2938	539_GENE_4
**9479**	**1**	**CGL2_09479001**	Phage/plasmid primase P4, C-terminal	730	366..2555	539_GENE_3
**10305**	**1**	**CGL2_10305001**	Hypothetical protein	149	complement(1119..1565)	
**10305**	**2**	**CGL2_10305002**	Hypothetical protein	226	complement(1568..2245)	
**10305**	**3**	**CGL2_10305003**	Conserved hypothetical protein	229	complement(2921..3607)	
**10305**	**4**	**CGL2_10305004**	DNA primase	717	complement(3607..5757)	
**10305**	**5**	**CGL2_10305005**	Hypothetical protein	64	complement(5772..5963)	51_GENE_2
**10305**	**6**	**CGL2_10305006**	Hypothetical protein	259	complement(6104..6880)	798_GENE_3
**10305**	**7**	**CGL2_10305007**	Conserved protein of unknown function	69	complement(6912..7118)	798_GENE_4
**10305**	**8**	**CGL2_10305008**	Conserved hypothetical protein	67	complement(7118..7318)	798_GENE_5
**10305**	**9**	**CGL2_10305009**	Putative phage integrase	161	complement(7353..7835)	
**10965**	**15**	**CGL2_10965015**	transposition helper protein, IstB	97	complement(10987..11277)	443_GENE_2
**10965**	**14**	**CGL2_10965014**	integrase Rci fragment (partial domain)	155	10438..10902	443_GENE_3
**10965**	**13**	**CGL2_10965013**	Hypothetical protein	72	complement(10199..10414)	443_GENE_4
**10965**	**12**	**CGL2_10965012**	Hypothetical protein	38	complement(10086..10199)	443_GENE_5
**10965**	**11**	**CGL2_10965011**	Hypothetical protein	151	9541..9993	443_GENE_6
**10965**	**10**	**CGL2_10965010**	Hypothetical protein	71	8861..9073	443_GENE_7
**10965**	**9**	**CGL2_10965009**	transposase	209	complement(8174..8800)	443_GENE_8

**10965**	**8**	**CGL2_10965008**	transposase	175	complement(7650..8174)	443_GENE_9
**10965**	**7**	**CGL2_10965007**	Hypothetical protein	224	6745..7416	443_GENE_10
**10965**	**6**	**CGL2_10965006**	Hypothetical protein	126	6368..6745	
**10965**	**5**	**CGL2_10965005**	Protein of unknown function	238	complement(5135..5848)	41_GENE_1
**10965**	**4**	**CGL2_10965004**	Putative sigma54 specific transcriptional regulator, F	454	3651..5012	41_GENE_2
**10965**	**3**	**CGL2_10965003**	Cytochrome bd ubiquinol oxidase, subunit I	534	complement(1859..3460)	41_GENE_4
**10965**	**2**	**CGL2_10965002**	Cytochrome bd ubiquinol oxidase, subunit II	379	complement(727..1863)	41_GENE_5
**10965**	**1**	**CGL2_10965001**	Cyd operon protein (YbgT)	43	complement(556..684)	41_GENE_6
**10961**	**1**	**CGL2_10961001**	Putative sulfide-quinone reductase	424	457..1728	41_GENE_7
**10961**	**2**	**CGL2_10961002**	Putative radical SAM superfamily protein	366	complement(1768..2865)	41_GENE_8
**10961**	**3**	**CGL2_10961003**	Conserved protein of unknown function	150	complement(2902..3351)	41_GENE_9
**10961**	**4**	**CGL2_10961004**	Conserved hypothetical protein	103	complement(3441..3749)	41_GENE_10
**10961**	**5**	**CGL2_10961005**	Probable major facilitator superfamily transporter	430	complement(3733..5022)	41_GENE_11
**10961**	**6**	**CGL2_10961006**	Putative glycosyl transferase, family 9	423	complement(5022..6290)	41_GENE_12
**10961**	**7**	**CGL2_10961007**	Putative two component transcriptional regulator, Lu	211	complement(6413..7045)	41_GENE_13
**10961**	**8**	**CGL2_10961008**	Putative periplasmic sensor signal transduction histid	532	complement(7045..8640)	41_GENE_14
**10961**	**9**	**CGL2_10961009**	Putative amino acid permease	665	complement(8612..10606)	41_GENE_15
**10961**	**10**	**CGL2_10961010**	Conserved protein of unknown function	482	complement(10712..12157)	41_GENE_16
**10961**	**11**	**CGL2_10961011**	Potassium-transporting ATPase, C subunit (EC 3.6.3.	205	complement(12266..12880)	41_GENE_17
**10961**	**12**	**CGL2_10961012**	Potassium-transporting ATPase, A subunit (EC 3.6.3.	567	complement(12939..14639)	41_GENE_18
**10961**	**13**	**CGL2_10961013**	Potassium-translocating P-type ATPase, B subunit (E	670	complement(14860..16869)	41_GENE_19
**10961**	**14**	**CGL2_10961014**	Protein of unknown function	202	complement(17129..17734)	41_GENE_20
**10961**	**15**	**CGL2_10961015**	Hypothetical protein	212	complement(17883..18518)	41_GENE_21
**10961**	**16**	**CGL2_10961016**	Hypothetical protein	199	complement(18784..19380)	41_GENE_22
**10961**	**17**	**CGL2_10961017**	Protein of unknown function	214	complement(19725..20366)	41_GENE_24
**10961**	**18**	**CGL2_10961018**	Putative osmosensitive K+ channel signal transductic	741	20561..22783	41_GENE_25
**10961**	**19**	**CGL2_10961019**	Hypothetical protein	192	22925..23500	41_GENE_26
**10961**	**20**	**CGL2_10961020**	Protein of unknown function	309	23535..24461	41_GENE_27
**10961**	**21**	**CGL2_10961021**	Putative NAD-binding 6-phosphogluconate dehydrog	307	complement(24476..25396)	41_GENE_28
**10961**	**22**	**CGL2_10961022**	Putative peroxiredoxin	191	complement(25396..25968)	41_GENE_29
**10961**	**23**	**CGL2_10961023**	Probable Dyp-type peroxidase	295	complement(26137..27021)	41_GENE_30
**10961**	**24**	**CGL2_10961024**	Putative CoA-binding protein	148	complement(27114..27557)	41_GENE_31
**10961**	**25**	**CGL2_10961025**	Probable ferredoxin	420	27772..29031	41_GENE_33
**10961**	**27**	**CGL2_10961027**	Conserved protein of unknown function	197	29119..29709	41_GENE_34
**10961**	**28**	**CGL2_10961028**	Cysteine desulfurase (EC 2.8.1.7)	390	29705..30874	41_GENE_35
**10961**	**29**	**CGL2_10961029**	Putative NAD-dependent epimerase/dehydratase	298	30915..31808	41_GENE_36
10961tRNA		**CGL2_10961R001**	TRNA Leu CAG		complement(32176..32261)	
10961tRNA		**CGL2_10961R002**	TRNA Arg TCT		complement(32500..32576)	
**10961**	**30**	**CGL2_10961030**	Hypothetical protein	68	complement(32609..32812)	287_GENE_8
**10961**	**31**	**CGL2_10961031**	Putative glutaredoxin	129	complement(32898..33284)	287_GENE_7
**10961**	**32**	**CGL2_10961032**	Protein of unknown function	450	33491..34840	287_GENE_6

**10961**	**33**	**CGL2_10961033**	Hypothetical protein	551	34846..36498	287_GENE_5
**10961**	**34**	**CGL2_10961034**	Protein of unknown function	206	36552..37169	287_GENE_4
**10961**	**35**	**CGL2_10961035**	Peptide methionine sulfoxide reductase (EC 1.8.4.6)	185	complement(37167..37721)	287_GENE_3
**10961**	**36**	**CGL2_10961036**	Diguanylate cyclase/phosphodiesterase	702	38235..40340	287_GENE_2
**10961**	**37**	**CGL2_10961037**	Protein of unknown function	206	40754..41371	86_GENE_19
**10961**	**38**	**CGL2_10961038**	Conserved hypothetical protein	63	41554..41742	86_GENE_18
**10961**	**39**	**CGL2_10961039**	Protein of unknown function	356	41864..42931	86_GENE_17
**10961**	**40**	**CGL2_10961040**	Protein of unknown function	215	42988..43632	86_GENE_16
10961tRNA		**CGL2_10961R003**	TRNA Lys TTT		complement(44332..44407)	
**10961**	**41**	**CGL2_10961041**	Conserved hypothetical protein	361	complement(44444..45526)	86_GENE_14
**10961**	**42**	**CGL2_10961042**	3-isopropylmalate dehydratase small subunit (EC 4.2	208	complement(45591..46214)	86_GENE_13
**10961**	**43**	**CGL2_10961043**	3-isopropylmalate dehydratase large subunit (EC 4.2	465	complement(46251..47645)	86_GENE_12
**10961**	**44**	**CGL2_10961044**	DTDP-glucose 4,6-dehydratase (EC 4.2.1.46)	357	47836..48906	86_GENE_11
**10961**	**45**	**CGL2_10961045**	Glucose-1-phosphate thymidyltransferase (EC 2.7.7.	299	48906..49802	86_GENE_10
**10961**	**47**	**CGL2_10961047**	DTDP-4-dehydrorhamnose 3,5-epimerase (EC 5.1.3.	183	49802..50350	86_GENE_9
**10961**	**48**	**CGL2_10961048**	Putative major facilitator superfamily transporter	425	complement(50382..51656)	86_GENE_8
**10961**	**49**	**CGL2_10961049**	Conserved protein of unknown function	316	51803..52750	86_GENE_7
**10961**	**50**	**CGL2_10961050**	Diguanylate cyclase/phosphodiesterase with PAS/PA(	878	52830..55463	86_GENE_6
**10961**	**51**	**CGL2_10961051**	Putative phosphoglycerate mutase	156	55498..55965	86_GENE_5
**10961**	**52**	**CGL2_10961052**	Glutamate decarboxylase (EC 4.1.1.15)	457	complement(56012..57382)	86_GENE_4
**10961**	**53**	**CGL2_10961053**	Malto-oligosyltrehalose trehalohydrolase (EC 3.2.1.1	637	57614..59524	86_GENE_3
**10961**	**54**	**CGL2_10961054**	Malto-oligosyltrehalose synthase (EC 5.4.99.15)	965	59524..62418	86_GENE_2
**10961**	**55**	**CGL2_10961055**	Trehalose synthase (EC 5.4.99.16)	556	62418..64085	213_GENE_8
**10961**	**56**	**CGL2_10961056**	Trehalose synthase (EC 5.4.99.16)	559	64085..65761	213_GENE_7
**10961**	**57**	**CGL2_10961057**	Putative fatty acid desaturase	338	complement(65872..66885)	213_GENE_6
**10961**	**58**	**CGL2_10961058**	Diguanylate cyclase	386	complement(67097..68254)	213_GENE_5
**10961**	**59**	**CGL2_10961059**	Hypothetical protein	280	69037..69876	213_GENE_4
**10961**	**60**	**CGL2_10961060**	Diguanylate cyclase	380	complement(69911..71050)	213_GENE_3
**10961**	**61**	**CGL2_10961061**	Protein of unknown function	211	complement(71244..71876)	206_GENE_8
**10961**	**62**	**CGL2_10961062**	Hypothetical protein	67	complement(71932..72132)	206_GENE_7
**10954**	**1**	**CGL2_10954001**	Putative alpha amylase, catalytic region	663	1250..3238	206_GENE_6
**10954**	**2**	**CGL2_10954002**	Probable alpha amylase, catalytic region	523	3238..4806	206_GENE_5
**10954**	**3**	**CGL2_10954003**	Zinc-containing alcohol dehydrogenase superfamily (	347	5003..6043	206_GENE_4
**10954**	**4**	**CGL2_10954004**	Hypothetical protein	107	6396..6716	206_GENE_3
**10954**	**5**	**CGL2_10954005**	Diguanylate cyclase with PAS/PAC sensor	1029	6892..9978	206_GENE_2
**10954**	**6**	**CGL2_10954006**	Putative enoyl-CoA hydratase/isomerase	288	complement(10723..11586)	206_GENE_1
**10954**	**7**	**CGL2_10954007**	Putative multi-sensor hybrid histidine kinase	849	complement(11980..14526)	7_GENE_62
**10954**	**8**	**CGL2_10954008**	Putative response regulator receiver modulated meta	376	complement(14619..15746)	7_GENE_61
**10954**	**9**	**CGL2_10954009**	Hypothetical protein	231	complement(16126..16818)	7_GENE_59
**10954**	**10**	**CGL2_10954010**	Hypothetical protein	295	complement(17224..18108)	7_GENE_58
**10954**	**11**	**CGL2_10954011**	Hypothetical protein	505	complement(18543..20057)	7_GENE_57

**10954**	**12**	**CGL2_10954012**	Probable TPR-domain containing protein	1076	complement(20057..23284)	7_GENE_56
**10954**	**13**	**CGL2_10954013**	Methyltransferase, FkbM family	262	complement(23911..24696)	7_GENE_54
**10954**	**14**	**CGL2_10954014**	Putative ABC transporter, ATP-binding protein	609	complement(24824..26650)	7_GENE_53
**10954**	**15**	**CGL2_10954015**	Hypothetical protein	299	complement(26650..27546)	7_GENE_52
**10954**	**16**	**CGL2_10954016**	SAM-dependent methyltransferase	286	complement(27894..28751)	7_GENE_51
**10954**	**17**	**CGL2_10954017**	Probable glycosyl transferase	439	complement(28918..30234)	7_GENE_50
**10954**	**18**	**CGL2_10954018**	Putative glycosyl transferase, family 2	364	complement(30285..31376)	7_GENE_49
**10954**	**19**	**CGL2_10954019**	Protein of unknown function	351	complement(31418..32470)	7_GENE_48
**10954**	**20**	**CGL2_10954020**	Putative glycosyl transferase, family 2	367	complement(32530..33630)	7_GENE_47
**10954**	**21**	**CGL2_10954021**	Probable glycosyl transferase, family 2	625	complement(33764..35638)	7_GENE_46
**10954**	**22**	**CGL2_10954022**	Protein of unknown function	681	complement(35644..37686)	7_GENE_45
**10954**	**23**	**CGL2_10954023**	Putative two component, sigma54 specific, transcript	471	complement(37766..39178)	7_GENE_44
**10954**	**24**	**CGL2_10954024**	Conserved hypothetical protein	237	complement(39178..39888)	7_GENE_43
**10954**	**25**	**CGL2_10954025**	Eight transmembrane protein (EpsH)	276	complement(39881..40708)	7_GENE_42
**10954**	**26**	**CGL2_10954026**	UTP-glucose-1-phosphate uridylyltransferase (EC 2.7	307	complement(40698..41618)	7_GENE_41
**10954**	**27**	**CGL2_10954027**	Putative periplasmic sensor signal transduction histid	704	complement(42142..44253)	7_GENE_39
**10954**	**28**	**CGL2_10954028**	Putative glycosyl transferase, family 2	329	44593..45579	7_GENE_38
**10954**	**29**	**CGL2_10954029**	Putative glycosyl transferase, family 2	336	complement(45689..46696)	7_GENE_37
**10954**	**30**	**CGL2_10954030**	Hypothetical protein	621	47061..48923	7_GENE_36
**10954**	**31**	**CGL2_10954031**	O-antigen polymerase	503	48994..50502	7_GENE_35
**10954**	**32**	**CGL2_10954032**	Putative glycosyl transferase, group 1	374	50502..51623	7_GENE_34
**10954**	**33**	**CGL2_10954033**	Putative polysaccharide deacetylase	653	51619..53577	7_GENE_33
**10954**	**34**	**CGL2_10954034**	Putative glycosyl transferase, group 1	394	53577..54758	7_GENE_32
**10954**	**35**	**CGL2_10954035**	Probable glycosyltransferase	381	54752..55894	7_GENE_31
**10954**	**36**	**CGL2_10954036**	Putative polysaccharide deacetylase	269	55882..56688	7_GENE_30
**10954**	**37**	**CGL2_10954037**	Conserved hypothetical protein	504	56688..58199	7_GENE_29
**10954**	**38**	**CGL2_10954038**	Putative polysaccharide export protein	285	58366..59220	7_GENE_28
**10954**	**39**	**CGL2_10954039**	Putative glycosyltransferase	447	59257..60597	7_GENE_27
**10954**	**40**	**CGL2_10954040**	Probable lipopolysaccharide biosynthesis protein	660	60620..62599	7_GENE_26
**10954**	**41**	**CGL2_10954041**	Conserved hypothetical protein	128	complement(62821..63204)	7_GENE_25
**10954**	**42**	**CGL2_10954042**	Conserved hypothetical protein	75	complement(63204..63428)	7_GENE_24
**10954**	**43**	**CGL2_10954043**	Hypothetical protein	99	complement(63468..63764)	7_GENE_23
**10954**	**44**	**CGL2_10954044**	Hypothetical protein	170	complement(63772..64281)	7_GENE_21a
**10954**	**45**	**CGL2_10954045**	Conserved hypothetical protein	85	64627..64881	7_GENE_21
**10954**	**46**	**CGL2_10954046**	RNA polymerase sigma factor	193	64881..65459	7_GENE_20
**10954**	**47**	**CGL2_10954047**	Hypothetical protein	153	65590..66048	7_GENE_19
**10954**	**48**	**CGL2_10954048**	Mercuric reductase (EC 1.16.1.1)	557	66148..67818	7_GENE_18
**10954**	**48**	**CGL2_10954048**	Mercuric reductase (EC 1.16.1.1)	557	66148..67818	7_GENE_18
**10954**	**50**	**CGL2_10954050**	Putative transcriptional regulator, MerR family	135	67864..68268	7_GENE_17
**10954**	**51**	**CGL2_10954051**	Putative mechanosensitive ion channel (MscS)	365	complement(68413..69507)	7_GENE_16
**10954**	**52**	**CGL2_10954052**	Diguanylate cyclase	371	complement(69689..70801)	7_GENE_15

**10954**	**53**	**CGL2_10954053**	Hypothetical protein	108	71327..71650	7_GENE_14
**10954**	**54**	**CGL2_10954054**	DNA adenine methylase (EC 2.1.1.72)	634	71980..73881	7_GENE_13
**10954**	**55**	**CGL2_10954055**	UDP-galactopyranose mutase (EC 5.4.99.9)	367	complement(74009..75109)	7_GENE_12
**10954**	**56**	**CGL2_10954056**	UDP-glucuronate 5'-epimerase (EC 5.1.3.12)	341	complement(75173..76195)	7_GENE_11
**10954**	**58**	**CGL2_10954058**	Probable adenylate cyclase	1202	complement(76310..79915)	7_GENE_10
**10954**	**59**	**CGL2_10954059**	Putative sigma54 specific transcriptional regulator, F	518	80349..81902	7_GENE_9
**10954**	**60**	**CGL2_10954060**	Glucosamine--fructose-6-phosphate aminotransferas	615	83321..85165	7_GENE_8
**10954**	**61**	**CGL2_10954061**	UDP-glucose 6-dehydrogenase (EC 1.1.1.22)	433	85205..86503	7_GENE_7
**10954**	**62**	**CGL2_10954062**	Protein of unknown function	170	86533..87042	7_GENE_6
**10954**	**63**	**CGL2_10954063**	UDP-glucose 4-epimerase (EC 5.1.3.2)	316	87101..88048	7_GENE_5
**10954**	**64**	**CGL2_10954064**	Putative sigma54 specific transcriptional regulator, F	475	complement(88049..89473)	7_GENE_4
**10954**	**65**	**CGL2_10954065**	Putative protein-tyrosine-phosphatase	189	89750..90316	7_GENE_3
**10954**	**66**	**CGL2_10954066**	Diguanylate cyclase/phosphodiesterase with PAS/PA(	747	90463..92703	7_GENE_2
**10954**	**67**	**CGL2_10954067**	Hypothetical protein	213	complement(93158..93796)	7_GENE_1
**10954**	**68**	**CGL2_10954068**	transposase	117	complement(94809..95159)	2_GENE_128
		Strain variant path	Strain variant path			Strain variant path
11068tRNA		**CGL2_11068R002**	TRNA Glu TTC		135482..135559	
**11068**	**123**	**CGL2_11068123**	Succinyl-CoA synthetase, beta subunit	416	complement(133987..135234)	2_GENE_126
**11068**	**122**	**CGL2_11068122**	Succinyl-CoA synthetase, alpha subunit (EC 6.2.1.5)	328	complement(132956..133939)	2_GENE_123
**11068**	**121**	**CGL2_11068121**	Hypothetical protein	50	complement(132772..132921)	2_GENE_122
**11068**	**120**	**CGL2_11068120**	Aconitate hydratase (EC 4.2.1.3)	641	complement(130542..132464)	2_GENE_121
**11068**	**119**	**CGL2_11068119**	Probable citrate synthase	269	complement(129639..130445)	2_GENE_120
**11068**	**118**	**CGL2_11068118**	Conserved hypothetical protein	200	complement(128965..129564)	2_GENE_119
**11068**	**117**	**CGL2_11068117**	Probable ferredoxin	228	complement(128170..128853)	2_GENE_118
**11068**	**116**	**CGL2_11068116**	L-aspartate oxidase (EC 1.4.3.16)	540	complement(126551..128170)	2_GENE_117
**11068**	**115**	**CGL2_11068115**	Succinyl-CoA synthetase, beta subunit (EC 6.2.1.5)	396	complement(125285..126472)	2_GENE_116
**11068**	**114**	**CGL2_11068114**	Succinyl-CoA synthetase, alpha subunit (EC 6.2.1.5)	305	complement(124371..125285)	2_GENE_115
**11068**	**113**	**CGL2_11068113**	Putative thioredoxin peroxidase	153	complement(123798..124256)	2_GENE_114
**11068**	**112**	**CGL2_11068112**	Protein of unknown function	180	123103..123642	2_GENE_113
**11068**	**111**	**CGL2_11068111**	Conserved hypothetical protein	80	122758..122997	2_GENE_111a
**11068**	**110**	**CGL2_11068110**	Conserved protein of unknown function	148	complement(122091..122534)	2_GENE_111
**11068**	**109**	**CGL2_11068109**	S-adenosyl-methyltransferase (MraW)	307	complement(121153..122073)	2_GENE_110
**11068**	**108**	**CGL2_11068108**	Hypothetical protein	91	complement(120888..121160)	2_GENE_109
**11068**	**107**	**CGL2_11068107**	Peptidoglycan glycosyltransferase (EC 2.4.1.129)	578	complement(119060..120793)	2_GENE_108
**11068**	**106**	**CGL2_11068106**	UDP-N-acetylmuramoylalanyl-D-glutamate--2, 6-dia	509	complement(117484..119010)	2_GENE_107
**11068**	**105**	**CGL2_11068105**	UDP-N-acetylmuramoyl-tripeptide--D-alanyl-D-alanir	495	complement(116000..117484)	2_GENE_106
**11068**	**104**	**CGL2_11068104**	Phospho-N-acetylmuramoyl-pentapeptide-transferas	357	complement(114927..115997)	2_GENE_105
**11068**	**103**	**CGL2_11068103**	UDP-N-acetylmuramoylalanine--D-glutamate ligase (	487	complement(113429..114889)	2_GENE_104
**11068**	**102**	**CGL2_11068102**	Putative cell division protein (FtsW)	396	complement(112242..113429)	2_GENE_103
**11068**	**101**	**CGL2_11068101**	N-acetylglucosaminyltransferase, MurG (EC 2.4.1.22	371	complement(111130..112242)	2_GENE_102
**11068**	**100**	**CGL2_11068100**	UDP-N-acetylmuramate--L-alanine ligase (EC 6.3.2.8	464	complement(109615..111006)	2_GENE_101

**11068**	**99**	**CGL2_11068099**	UDP-N-acetylenolpyruvoylglucosamine reductase (EC	304	complement(108704..109615)	2_GENE_100
**11068**	**98**	**CGL2_11068098**	D-alanine--D-alanine ligase (EC 6.3.2.4)	303	complement(107742..108650)	2_GENE_99
**11068**	**97**	**CGL2_11068097**	Protein of unknown function	286	complement(106879..107736)	2_GENE_98
**11068**	**96**	**CGL2_11068096**	Cell division protein (FtsA)	442	complement(105459..106784)	2_GENE_97
**11068**	**95**	**CGL2_11068095**	Cell division protein (FtsZ)	390	complement(104238..105407)	2_GENE_96
**11068**	**94**	**CGL2_11068094**	Conserved hypothetical protein	216	complement(103587..104234)	2_GENE_95
**11068**	**93**	**CGL2_11068093**	Conserved protein of unknown function	230	complement(102887..103576)	2_GENE_94
**11068**	**92**	**CGL2_11068092**	Pyrroline-5-carboxylate reductase (EC 1.5.1.2)	288	complement(102019..102882)	2_GENE_93
**11068**	**91**	**CGL2_11068091**	Conserved hypothetical protein	72	complement(101796..102011)	2_GENE_92
**11068**	**90**	**CGL2_11068090**	Probable cell-division initiation protein	175	complement(101221..101745)	2_GENE_91
**11068**	**89**	**CGL2_11068089**	Conserved hypothetical protein	102	complement(100897..101202)	2_GENE_90
**11068**	**88**	**CGL2_11068088**	Protein of unknown function	199	100298..100894	2_GENE_89
**11068**	**87a**	**CGL2_11068087a**	Probable transposase	197	99506..100096	
**11068**	**87**	**CGL2_11068087**	Squalene-hopene cyclase (EC 5.4.99.17)	683	complement(96980..99028)	2_GENE_87
**11068**	**86**	**CGL2_11068086**	Protein of unknown function	262	complement(96195..96980)	2_GENE_86
**11068**	**85**	**CGL2_11068085**	Conserved protein of unknown function	446	complement(94858..96195)	2_GENE_85
**11068**	**84**	**CGL2_11068084**	Protein of unknown function	232	94211..94906	2_GENE_84
**11068**	**83**	**CGL2_11068083**	Dihydroflavonol 4-reductase (EC 1.1.1.219)	336	complement(93047..94054)	2_GENE_83
**11068**	**82**	**CGL2_11068082**	4-hydroxy-3-methylbut-2-enyl diphosphate reductas	302	complement(92054..92959)	2_GENE_82
**11068**	**81**	**CGL2_11068081**	Putative radical SAM family protein	479	complement(90578..92014)	2_GENE_81
**11068**	**80**	**CGL2_11068080**	Ceramide glucosyltransferase (EC 2.4.1.80)	412	complement(89327..90562)	2_GENE_80
**11068**	**79**	**CGL2_11068079**	Conserved hypothetical protein	97	complement(88941..89231)	2_GENE_79
**11068**	**78**	**CGL2_11068078**	Conserved hypothetical protein	257	complement(88143..88913)	2_GENE_78
**11068**	**77**	**CGL2_11068077**	Putative cobalamin (Vitamin B12) biosynthesis (CbiX	276	complement(87206..88033)	2_GENE_77
**11068**	**76**	**CGL2_11068076**	Uroporphyrinogen decarboxylase (EC 4.1.1.37)	356	complement(86136..87203)	2_GENE_76
**11068**	**75**	**CGL2_11068075**	Oxygen-independent coproporphyrinogen III oxidase	459	complement(84684..86060)	2_GENE_75
**11068**	**74**	**CGL2_11068074**	Protein of unknown function	126	84204..84581	2_GENE_74
11068tRNA		**CGL2_11068R001**	TRNA Ala GGC		complement(83757..83832)	
**11068**	**73**	**CGL2_11068073**	S-adenosylmethionine synthetase (EC 2.5.1.6)	404	complement(82156..83367)	2_GENE_72
**11068**	**72**	**CGL2_11068072**	Adenosylhomocysteinase (EC 3.3.1.1)	418	complement(80813..82066)	2_GENE_71
**11068**	**71**	**CGL2_11068071**	Thiamine biosynthesis protein (ThiS)	76	complement(80579..80806)	2_GENE_70
**11068**	**70**	**CGL2_11068070**	Cysteine synthase (EC 2.5.1.47)	312	complement(79663..80598)	2_GENE_69
**11068**	**69**	**CGL2_11068069**	Putative ThiF family protein	270	complement(78814..79623)	2_GENE_68
**11068**	**68**	**CGL2_11068068**	Threonine synthase (EC 4.2.3.1)	407	complement(77571..78791)	2_GENE_67
**11068**	**67**	**CGL2_11068067**	Putative ThiS family protein	93	complement(77268..77546)	2_GENE_66
**11068**	**66**	**CGL2_11068066**	Conserved protein of unknown function	80	complement(77029..77268)	2_GENE_65
**11068**	**65**	**CGL2_11068065**	Putative molybdopterin biosynthesis protein	267	complement(76201..77001)	2_GENE_64
**11068**	**64**	**CGL2_11068064**	Conserved protein of unknown function	142	complement(75773..76198)	2_GENE_63
**11068**	**63**	**CGL2_11068063**	Putative two component transcriptional regulator, wi	231	complement(75078..75770)	2_GENE_62
**11068**	**62**	**CGL2_11068062**	Putative multi-sensor signal transduction histidine ki	453	complement(73706..75064)	2_GENE_61
**11068**	**61**	**CGL2_11068061**	UDP-glucose 4-epimerase (EC 5.1.3.2)	323	72739..73707	2_GENE_60

**11068**	**60**	**CGL2_11068060**	Apurinic endonuclease (APN1) (EC 3.1.21.2)	318	complement(71466..72419)	2_GENE_59
**11068**	**59**	**CGL2_11068059**	Probable ABC transporter, permease	251	complement(70688..71440)	2_GENE_58
**11068**	**58**	**CGL2_11068058**	ABC transporter, ATP-binding protein (EC 3.6.3.25)	240	complement(69969..70688)	2_GENE_57
**11068**	**57**	**CGL2_11068057**	Conserved protein of unknown function	325	complement(68948..69922)	2_GENE_56
**11068**	**56**	**CGL2_11068056**	Putative metallophosphoesterase	385	complement(67767..68921)	2_GENE_55
**11068**	**55**	**CGL2_11068055**	Protein of unknown function	226	66933..67610	2_GENE_54
**11068**	**54**	**CGL2_11068054**	Diguanylate cyclase/phosphodiesterase with PAS/PA(	1139	63393..66809	2_GENE_53
**11068**	**53**	**CGL2_11068053**	4-hydroxythreonine-4-phosphate dehydrogenase (EC	344	61971..63002	2_GENE_52
**11068**	**52**	**CGL2_11068052**	Cation efflux system protein	302	61091..61996	2_GENE_51
**11068**	**51**	**CGL2_11068051**	Excinuclease ABC, A subunit (UvrA)	935	58287..61091	2_GENE_50
**11068**	**50**	**CGL2_11068050**	Probable outer membrane lipoprotein carrier protein	238	57571..58284	2_GENE_49
**11068**	**49**	**CGL2_11068049**	Putative cell division protein (FtsK)	758	55308..57581	2_GENE_48
**11068**	**48**	**CGL2_11068048**	Metallo-beta-lactamase family protein	562	53608..55293	2_GENE_47
**11068**	**47**	**CGL2_11068047**	Putative dimethyladenosine transferase	256	52841..53608	2_GENE_46
**11068**	**46**	**CGL2_11068046**	Putative TonB protein	367	complement(51509..52609)	2_GENE_45
**11068**	**45**	**CGL2_11068045**	Putative lytic transglycosylase	686	complement(49462..51519)	2_GENE_44
**11068**	**44**	**CGL2_11068044**	Protein of unknown function	91	complement(49181..49453)	2_GENE_43
**11068**	**43**	**CGL2_11068043**	Protein of unknown function	111	complement(48865..49197)	2_GENE_42
**11068**	**42**	**CGL2_11068042**	Probable ATPase, AAA family	444	complement(47299..48630)	2_GENE_41
**11068**	**41**	**CGL2_11068041**	Metal dependent phosphohydrolase	521	complement(45531..47093)	2_GENE_40
**11068**	**40**	**CGL2_11068040**	Conserved protein of unknown function	284	complement(44696..45547)	2_GENE_39
**11068**	**39**	**CGL2_11068039**	Exodeoxyribonuclease VII large subunit (EC 3.1.11.6	503	complement(43181..44689)	2_GENE_38
**11068**	**38**	**CGL2_11068038**	Exodeoxyribonuclease VII small subunit	97	complement(42885..43175)	2_GENE_37
**11068**	**37**	**CGL2_11068037**	Geranyltranstransferase (EC 2.5.1.10)	303	complement(41932..42840)	2_GENE_36
**11068**	**36**	**CGL2_11068036**	Deoxyxylulose-5-phosphate synthase (EC 2.2.1.7)	630	complement(40040..41929)	2_GENE_35
**11068**	**35**	**CGL2_11068035**	Hemolysin A	251	complement(39265..40017)	2_GENE_34
**11068**	**34**	**CGL2_11068034**	DTDP-glucose 4,6-dehydratase (EC 4.2.1.46)	313	complement(38317..39255)	2_GENE_33
**11068**	**33**	**CGL2_11068033**	Putative divalent-cation tolerance protein (CutA)	113	complement(37979..38317)	2_GENE_32
**11068**	**32**	**CGL2_11068032**	Putative peptidase, M23B family	316	complement(37032..37979)	2_GENE_31
**11068**	**31**	**CGL2_11068031**	Preprotein translocase SecA subunit	908	complement(34290..37013)	2_GENE_30
**11068**	**30**	**CGL2_11068030**	Putative NADH dehydrogenase (ubiquinone), subunit	123	complement(33727..34095)	2_GENE_29
**11068**	**29**	**CGL2_11068029**	NADH dehydrogenase (ubiquinone), B subunit (EC 1.	178	complement(33200..33733)	2_GENE_28
**11068**	**28**	**CGL2_11068028**	NADH dehydrogenase (quinone), chain C (EC 1.6.99.	184	complement(32639..33190)	2_GENE_27
**11068**	**27**	**CGL2_11068027**	NADH dehydrogenase (ubiquinone), D subunit (EC 1.	423	complement(31307..32575)	2_GENE_26
**11068**	**26**	**CGL2_11068026**	Putative NADH dehydrogenase (ubiquinone), E subur	168	complement(30653..31156)	2_GENE_25
**11068**	**25**	**CGL2_11068025**	NADH dehydrogenase (quinone) (EC 1.6.99.5)	617	complement(28689..30539)	2_GENE_24
**11068**	**24**	**CGL2_11068024**	NADH dehydrogenase (quinone), chain G (EC 1.6.99	903	complement(25957..28665)	2_GENE_23
**11068**	**23**	**CGL2_11068023**	NADH dehydrogenase (ubiquinone), H subunit	337	complement(24865..25875)	2_GENE_22
**11068**	**22**	**CGL2_11068022**	NADH dehydrogenase, chain I (EC 1.6.99.3)	186	complement(24279..24836)	2_GENE_21
**11068**	**21**	**CGL2_11068021**	Putative NADH dehydrogenase (ubiquinone), chain J	169	complement(23761..24267)	2_GENE_20
**11068**	**20**	**CGL2_11068020**	Putative NADH dehydrogenase (ubiquinone), chain 4	100	complement(23399..23698)	2_GENE_19

**11068**	**19**	**CGL2_11068019**	NADH dehydrogenase (quinone), chain L (EC 1.6.99.	639	complement(21455..23371)	2_GENE_18
**11068**	**18**	**CGL2_11068018**	NADH dehydrogenase (quinone), chain M (EC 1.6.99	500	complement(19910..21409)	2_GENE_17
**11068**	**17**	**CGL2_11068017**	NADH dehydrogenase (quinone), chain N (EC 1.6.99	481	complement(18424..19866)	2_GENE_16
**11068**	**16**	**CGL2_11068016**	LexA repressor (EC 3.4.21.88)	222	complement(17729..18394)	2_GENE_15
**11068**	**15**	**CGL2_11068015**	Conserved protein of unknown function	156	complement(17268..17735)	2_GENE_14
**11068**	**14**	**CGL2_11068014**	Ammonium transporter	505	complement(15508..17022)	2_GENE_13
**11068**	**13**	**CGL2_11068013**	Putative nitrogen regulatory protein P-II	112	complement(15173..15508)	2_GENE_12
**11068**	**12**	**CGL2_11068012**	Putative helicase, Snf2 family	1049	complement(11956..15102)	2_GENE_11
**11068**	**11**	**CGL2_11068011**	Probable proline dehydrogenase	270	complement(11116..11925)	2_GENE_10
**11068**	**10**	**CGL2_11068010**	Conserved protein of unknown function	80	10838..11077	2_GENE_9
**11068**	**9**	**CGL2_11068009**	Transposase	479	complement(9122..10558)	2_GENE_8
**11068**	**8**	**CGL2_11068008**	Hypothetical protein	157	complement(8533..9003)	2_GENE_7
**11068**	**7**	**CGL2_11068007**	Diguanylate cyclase/phosphodiesterase with PAS/PA(	1035	5408..8512	2_GENE_6
**11068**	**5**	**CGL2_11068005**	Conserved protein of unknown function	346	4239..5276	2_GENE_5
**11068**	**4**	**CGL2_11068004**	Protein of unknown function	215	3508..4152	2_GENE_4
**11068**	**3**	**CGL2_11068003**	Conserved hypothetical protein	499	1968..3464	2_GENE_3
**11068**	**2**	**CGL2_11068002**	Putative TPR domain containing protein	125	1594..1968	2_GENE_2
**11068**	**1**	**CGL2_11068001**	Diguanylate cyclase	393	complement(266..1444)	2_GENE_1
**10822**	**1**	**CGL2_10822001**	Putative TPR domain containing protein	204	complement(826..1437)	186_GENE_13
**10822**	**2**	**CGL2_10822002**	Putative general secretion pathway protein K	346	complement(1542..2579)	186_GENE_11
**10822**	**3**	**CGL2_10822003**	Hypothetical protein	196	complement(2557..3144)	186_GENE_10
**10822**	**4**	**CGL2_10822004**	Protein of unknown function	452	complement(3140..4495)	186_GENE_9
**10822**	**5**	**CGL2_10822005**	Protein of unknown function	290	complement(4506..5375)	186_GENE_8
**10822**	**6**	**CGL2_10822006**	General secretion pathway protein G	159	complement(5553..6029)	186_GENE_7
**10822**	**7**	**CGL2_10822007**	Putative general secretion pathway protein F	400	complement(6067..7266)	186_GENE_6
**10822**	**8**	**CGL2_10822008**	General secretion pathway protein E	571	complement(7290..9002)	186_GENE_5
**10822**	**9**	**CGL2_10822009**	Putative general secretion pathway protein D	555	complement(9010..10674)	186_GENE_4
**10822**	**10**	**CGL2_10822010**	Probable general secretion pathway protein C	323	complement(10674..11642)	186_GENE_3
**10822**	**11**	**CGL2_10822011**	Thiamine biosynthesis protein (ThiS)	70	11796..12005	186_GENE_2
10822tRNA		**CGL2_10822R001**	TRNA Gly CCC		12087..12161	
**10822**	**14**	**CGL2_10822014**	Hypothetical protein	97	complement(12331..12621)	710_GENE_6
**10822**	**17**	**CGL2_10822017**	replication protein A	455	12952..14316	710_GENE_3
**10822**	**18**	**CGL2_10822018**	replication protein C	335	14270..15274	710_GENE_2
**10822**	**19**	**CGL2_10822019**	Hypothetical protein	44	15252..15383	710_GENE_1
**8970**	**2**	**CGL2_08970002**	Probable cytochrome C, class I	269	1992..2798	46_GENE_28
**8970**	**1**	**CGL2_08970001**	Probable cytochrome C, class I	269	1118..1924	46_GENE_27
**11238**	**109**	**CGL2_11238109**	Probable cytochrome C, class I	225	118679..119353	46_GENE_26
**11238**	**108**	**CGL2_11238108**	Diguanylate cyclase/phosphodiesterase with GAF ser	988	115484..118447	46_GENE_25
**11238**	**107**	**CGL2_11238107**	Conserved hypothetical protein	173	complement(114772..115290)	46_GENE_24
**11238**	**106**	**CGL2_11238106**	Probable phytoene dehydrogenase	496	113294..114781	46_GENE_23
**11238**	**105**	**CGL2_11238105**	Hypothetical protein	351	112205..113257	46_GENE_22

**11238**	**104**	**CGL2_11238104**	Protein of unknown function	556	110490..112157	46_GENE_21
**11238**	**103**	**CGL2_11238103**	Probable cellulose synthase subunit C	335	109270..110274	46_GENE_20
**11238**	**102**	**CGL2_11238102**	Probable cellulose synthase regulatory subunit	755	107006..109270	46_GENE_19
**11238**	**101**	**CGL2_11238101**	Cellulose synthase catalytic subunit (UDP-forming) (I	714	104865..107006	46_GENE_18
**11238**	**100**	**CGL2_11238100**	Putative cytochrome C, class I	223	complement(103674..104342)	46_GENE_17
**11238**	**99**	**CGL2_11238099**	Putative cytochrome C, class I	272	complement(102794..103609)	46_GENE_16
**11238**	**98**	**CGL2_11238098**	DNA polymerase family B	736	100584..102791	46_GENE_15
**11238**	**97**	**CGL2_11238097**	Conserved hypothetical protein	87	100346..100606	46_GENE_14
**11238**	**96**	**CGL2_11238096**	Hypothetical protein	179	99807..100343	46_GENE_13
**11238**	**95**	**CGL2_11238095**	LexA repressor (EC 3.4.21.88)	205	99187..99801	46_GENE_12
**11238**	**94**	**CGL2_11238094**	Glucose-6-phosphate isomerase (EC 5.3.1.9)	548	97426..99069	46_GENE_11
**11238**	**93**	**CGL2_11238093**	Conserved protein of unknown function	238	96657..97370	46_GENE_10
**11238**	**92**	**CGL2_11238092**	Hypothetical protein	125	96254..96628	46_GENE_7a
**11238**	**91**	**CGL2_11238091**	Putative acriflavin resistance protein	1036	92842..95949	46_GENE_7
**11238**	**90**	**CGL2_11238090**	Protein of unknown function	211	92089..92721	46_GENE_6
**11238**	**89**	**CGL2_11238089**	Protein of unknown function	200	91251..91850	46_GENE_5
**11238**	**88**	**CGL2_11238088**	Protein of unknown function	103	90729..91037	46_GENE_4
**11238**	**87**	**CGL2_11238087**	Arsenical pump membrane protein	439	89215..90531	46_GENE_3
**11238**	**86**	**CGL2_11238086**	Putative transcriptional regulator, ArsR family	269	88402..89208	46_GENE_2
**11238**	**84**	**CGL2_11238084**	transposase	479	86625..88061	297_GENE_3
**11238**	**83**	**CGL2_11238083**	Protein of unknown function	168	86040..86543	297_GENE_4
**11238**	**82**	**CGL2_11238082**	Protein of unknown function	231	complement(85079..85771)	297_GENE_5
**11238**	**81**	**CGL2_11238081**	Putative Kelch domain-containing protein	392	83931..85106	297_GENE_6
**11238**	**80**	**CGL2_11238080**	Conserved hypothetical protein	125	83020..83394	297_GENE_7
**11238**	**79**	**CGL2_11238079**	Putative iron permease, FTR1 family	292	82145..83020	297_GENE_8
**11238**	**78**	**CGL2_11238078**	Putative metallo-beta-lactamase family protein	479	complement(80493..81929)	66_GENE_28
**11238**	**77**	**CGL2_11238077**	Long-chain-fatty-acid--CoA ligase (EC 6.2.1.3)	900	complement(77771..80470)	66_GENE_27
**11238**	**76**	**CGL2_11238076**	Hypothetical protein	209	77156..77782	66_GENE_26
**11238**	**75**	**CGL2_11238075**	Transcriptional regulator, BadM/Rrf2 family	169	complement(76403..76909)	66_GENE_24
**11238**	**74**	**CGL2_11238074**	Conserved hypothetical protein	76	complement(76132..76359)	66_GENE_23
**11238**	**73**	**CGL2_11238073**	Probable oxidoreductase FAD/NAD(P)-binding	248	75285..76028	66_GENE_22
**11238**	**72**	**CGL2_11238072**	Putative cytochrome c	120	complement(74647..75006)	66_GENE_21
**11238**	**71**	**CGL2_11238071**	Conserved protein of unknown function	64	complement(74338..74529)	66_GENE_20
**11238**	**70**	**CGL2_11238070**	Putative flavohemoprotein	133	73794..74192	66_GENE_19
**11238**	**69**	**CGL2_11238069**	Phosphomannomutase (EC 5.4.2.8)	467	72203..73603	66_GENE_18
**11238**	**68**	**CGL2_11238068**	Mannose-1-phosphate guanylyltransferase (EC 2.7.7	491	70731..72203	66_GENE_17
**11238**	**67**	**CGL2_11238067**	Hypothetical protein	90	complement(69993..70262)	66_GENE_15
**11238**	**66**	**CGL2_11238066**	Protein of unknown function	245	complement(69190..69924)	66_GENE_14
**11238**	**65**	**CGL2_11238065**	Putative short-chain dehydrogenase	277	68334..69164	66_GENE_13
**11238**	**64**	**CGL2_11238064**	GTP-binding protein (Era)	312	complement(67283..68218)	66_GENE_12
**11238**	**63**	**CGL2_11238063**	Recombination protein O (RecO)	246	complement(66530..67267)	66_GENE_11

**11238**	**62**	**CGL2_11238062**	Hypothetical protein	191	65903..66475	66_GENE_10
**11238**	**61**	**CGL2_11238061**	Protein of unknown function	332	64620..65615	66_GENE_9
**11238**	**60**	**CGL2_11238060**	Conserved protein of unknown function	391	complement(63115..64287)	66_GENE_8
**11238**	**59**	**CGL2_11238059**	GTP-binding protein (TypA)	623	complement(61090..62958)	66_GENE_7
**11238**	**58**	**CGL2_11238058**	Peptidyl-prolyl cis-trans isomerase (EC 5.2.1.8)	218	complement(60423..61076)	66_GENE_6
**11238**	**57**	**CGL2_11238057**	Putative metallo-beta-lactamase family protein	262	complement(59570..60355)	66_GENE_5
**11238**	**56**	**CGL2_11238056**	5-methyltetrahydrofolate--homocysteine methyltrans	815	57123..59567	66_GENE_4
**11238**	**55**	**CGL2_11238055**	Conserved protein of unknown function	282	56278..57123	66_GENE_3
**11238**	**54**	**CGL2_11238054**	Thiamine biosynthesis protein (ThiC)	468	54838..56241	134_GENE_2
**11238**	**53**	**CGL2_11238053**	Probable ATP-dependent Clp protease	1026	51541..54618	134_GENE_3
**11238**	**52**	**CGL2_11238052**	Excinuclease ABC, C subunit (UvrC)	632	49646..51541	134_GENE_4
**11238**	**51**	**CGL2_11238051**	Hypothetical protein	84	complement(49298..49549)	134_GENE_5
**11238**	**50**	**CGL2_11238050**	Shikimate 5-dehydrogenase (EC 1.1.1.25)	290	complement(48387..49256)	134_GENE_6
**11238**	**49**	**CGL2_11238049**	Cell division protein (FtsY)	309	47434..48360	134_GENE_7
**11238**	**48**	**CGL2_11238048**	Conserved protein of unknown function	169	46893..47399	134_GENE_8
**11238**	**47**	**CGL2_11238047**	Metal dependent phosphohydrolase	501	45398..46900	134_GENE_9
**11238**	**46**	**CGL2_11238046**	Putative phosphate starvation-inducible protein (Pho	331	44374..45366	134_GENE_10
**11238**	**45**	**CGL2_11238045**	Probable universal stress protein (UspA)	178	43838..44371	134_GENE_11
**11238**	**44**	**CGL2_11238044**	Putative type IV prepilin peptidase	263	42887..43675	410_GENE_2
**11238**	**43**	**CGL2_11238043**	Protein of unknown function	105	42412..42726	410_GENE_3
**11238**	**42**	**CGL2_11238042**	Putative polyphosphate kinase (PPK2)	275	41539..42363	410_GENE_4
**11238**	**41**	**CGL2_11238041**	Probable oxidoreductase	254	40731..41492	410_GENE_5
11238tRNA		**CGL2_11238R001**	TRNA Thr TGT		complement(40186..40261)	
**11238**	**40**	**CGL2_11238040**	Putative response regulator receiver protein	326	complement(38975..39952)	410_GENE_6
**11238**	**39**	**CGL2_11238039**	Conserved protein of unknown function	200	complement(38170..38769)	410_GENE_7
**11238**	**38**	**CGL2_11238038**	Protein of unknown function	74	complement(37914..38135)	410_GENE_8
**11238**	**37**	**CGL2_11238037**	Conserved protein of unknown function	167	complement(37308..37808)	467_GENE_5a
**11238**	**36**	**CGL2_11238036**	Transcription termination factor (NusA)	452	complement(35915..37270)	467_GENE_5
**11238**	**35**	**CGL2_11238035**	Translation initiation factor IF-2	874	complement(33257..35878)	467_GENE_4
**11238**	**34**	**CGL2_11238034**	Conserved protein of unknown function	99	complement(32938..33234)	467_GENE_3
**11238**	**33**	**CGL2_11238033**	Probable ribosome-binding factor A	131	complement(32543..32935)	467_GENE_2
**11238**	**32**	**CGL2_11238032**	TRNA pseudouridine synthase B (EC 4.2.1.70)	325	complement(31569..32543)	467_GENE_1
**11238**	**31**	**CGL2_11238031**	Ribosomal protein S15	89	complement(31242..31508)	71_GENE_25
**11238**	**30**	**CGL2_11238030**	Polyribonucleotide nucleotidyltransferase (EC 2.7.7.8	713	complement(28969..31107)	71_GENE_24
**11238**	**29**	**CGL2_11238029**	Hypothetical protein	39	complement(28811..28927)	71_GENE_23
**11238**	**28**	**CGL2_11238028**	Processing peptidase (EC 3.4.24.64)	411	complement(27357..28589)	71_GENE_22
**11238**	**27**	**CGL2_11238027**	Putative pyridoxal phosphate biosynthesis protein	255	complement(26593..27357)	71_GENE_21
**11238**	**26**	**CGL2_11238026**	Holo-[acyl-carrier-protein] synthase (EC 2.7.8.7)	133	complement(26195..26593)	71_GENE_20
**11238**	**25**	**CGL2_11238025**	Carbohydrate kinase family protein	518	complement(24642..26195)	71_GENE_19
**11238**	**24**	**CGL2_11238024**	Uracil-DNA glycosylase, family 4	245	complement(23835..24569)	71_GENE_18
**11238**	**23**	**CGL2_11238023**	Hypothetical protein	85	complement(23581..23835)	71_GENE_17

**11238**	**22**	**CGL2_11238022**	DNA mismatch repair protein (MutS)	843	complement(21057..23585)	71_GENE_16
**11238**	**21**	**CGL2_11238021**	Protein of unknown function	381	complement(19796..20938)	71_GENE_15
**11238**	**20**	**CGL2_11238020**	Protein of unknown function	390	complement(18605..19774)	71_GENE_14
**11238**	**19**	**CGL2_11238019**	Enoyl-(Acyl-carrier-protein) reductase (EC 1.3.1.9)	265	complement(17770..18564)	71_GENE_13
**11238**	**18**	**CGL2_11238018**	Probable rieske iron-sulfur family protein	309	complement(16788..17714)	71_GENE_12
**11238**	**17**	**CGL2_11238017**	Putative cytochrome b/b6, N-terminal	459	complement(15390..16766)	71_GENE_11
**11238**	**16**	**CGL2_11238016**	Probable thioredoxin	83	15106..15354	71_GENE_10
**11238**	**15**	**CGL2_11238015**	Hypothetical protein	62	complement(14770..14955)	71_GENE_8a
**11238**	**14**	**CGL2_11238014**	Endoribonuclease L-PSP	128	complement(14394..14777)	71_GENE_8
**11238**	**13**	**CGL2_11238013**	Ribosomal protein L28	63	14158..14346	71_GENE_7a
**11238**	**12**	**CGL2_11238012**	Putative ATP-dependent DNA helicase (RecG)	713	complement(11869..14007)	71_GENE_7
**11238**	**11**	**CGL2_11238011**	Protein of unknown function	119	complement(11528..11884)	71_GENE_6
**11238**	**10**	**CGL2_11238010**	Ppx/GppA phosphatase (EC 3.6.1.11)	315	complement(10578..11522)	71_GENE_5
**11238**	**9**	**CGL2_11238009**	Protein of unknown function	587	8839..10599	71_GENE_4
**11238**	**8**	**CGL2_11238008**	Putative polyA polymerase family protein	419	complement(7472..8728)	71_GENE_3
**11238**	**7**	**CGL2_11238007**	Protein of unknown function	166	6882..7379	71_GENE_2
**11238**	**6**	**CGL2_11238006**	Putative acriflavin resistance protein	1027	complement(3665..6745)	428_GENE_7
**11238**	**5**	**CGL2_11238005**	Protein of unknown function	206	complement(2986..3603)	428_GENE_6
**11238**	**4**	**CGL2_11238004**	Putative ferric uptake regulator, Fur family	146	complement(2468..2905)	428_GENE_5
**11238**	**3**	**CGL2_11238003**	Protein of unknown function	154	complement(1923..2384)	428_GENE_3
**11238**	**2**	**CGL2_11238002**	Conserved protein of unknown function	261	1114..1896	428_GENE_2
**11238**	**1**	**CGL2_11238001**	Protein of unknown function	305	186..1100	428_GENE_1
**10665**	**1**	**CGL2_10665001**	Ribosomal protein S1	591	23..1795	274_GENE_1
**10665**	**2**	**CGL2_10665002**	Peptidase S49 (SppA)	295	1859..2743	274_GENE_2
**10665**	**3**	**CGL2_10665003**	Putative integration host factor, beta-subunit	98	2772..3065	274_GENE_2a
**10665**	**4**	**CGL2_10665004**	Signal recognition particle protein	444	3068..4399	274_GENE_3
**10665**	**5**	**CGL2_10665005**	Ribosomal protein S16	81	4448..4690	274_GENE_4
**10665**	**6**	**CGL2_10665006**	Conserved protein of unknown function	76	4741..4968	274_GENE_5
**10665**	**7**	**CGL2_10665007**	Hypothetical protein	108	5199..5522	274_GENE_5a
**10665**	**8**	**CGL2_10665008**	tRNA (guanine-N(1)-)-methyltransferase (EC 2.1.1.3	241	5495..6217	274_GENE_6
**10665**	**9**	**CGL2_10665009**	Ribosomal protein L19	118	6286..6639	274_GENE_7
**10665**	**10**	**CGL2_10665010**	Ribonuclease HII (EC 3.1.26.4)	228	6656..7339	274_GENE_8
**10665**	**11**	**CGL2_10665011**	Hypothetical protein	301	7352..8254	274_GENE_9
**10665**	**12**	**CGL2_10665012**	Putative peptidase M23B family protein	398	8227..9420	274_GENE_10
**10776**	**10**	**CGL2_10776010**	Chaperonin GroEL	204	complement(11391..12002)	
**10776**	**9**	**CGL2_10776009**	Thioredoxin	110	complement(10919..11248)	222_GENE_9
**10776**	**8**	**CGL2_10776008**	Putative DNA repair protein (RecN)	564	complement(9089..10780)	222_GENE_7
**10776**	**7**	**CGL2_10776007**	Conserved protein of unknown function	915	6268..9012	222_GENE_6
**10776**	**6**	**CGL2_10776006**	Arginine decarboxylase (EC 4.1.1.19)	489	4753..6219	222_GENE_5
**10776**	**5**	**CGL2_10776005**	Spermidine synthase (EC 2.5.1.16)	306	3821..4738	222_GENE_4

**10776**	**4**	**CGL2_10776004**	Putative S-adenosylmethionine decarboxylase	154	3391..3852	222_GENE_3
**10776**	**3**	**CGL2_10776003**	Diguanylate cyclase	740	830..3049	222_GENE_2
**10776**	**2**	**CGL2_10776002**	Protein of unknown function	183	81..629	222_GENE_1
**10933**	**1**	**CGL2_10933001**	Protein of unknown function	247	456..1196	44_GENE_1
**10933**	**2**	**CGL2_10933002**	Putative phosphatidylglycerophosphatase A	180	1186..1725	44_GENE_2
**10933**	**3**	**CGL2_10933003**	RecA protein	360	1837..2916	44_GENE_3
**10933**	**4**	**CGL2_10933004**	Alanyl-tRNA synthetase (EC 6.1.1.7)	891	3000..5672	44_GENE_4
**10933**	**5**	**CGL2_10933005**	Protein of unknown function	94	5672..5953	44_GENE_5
**10933**	**6**	**CGL2_10933006**	Putative holliday junction resolvase YqgF	171	5973..6485	44_GENE_6
**10933**	**7**	**CGL2_10933007**	Aminodeoxychorismate lyase	341	6517..7539	44_GENE_7
**10933**	**8**	**CGL2_10933008**	Valyl-tRNA synthetase (EC 6.1.1.9)	904	7571..10282	44_GENE_8
**10933**	**9**	**CGL2_10933009**	Nicotinate-nucleotide pyrophosphorylase (EC 2.4.2.1	292	10282..11157	44_GENE_9
**10933**	**10**	**CGL2_10933010**	Biotin--acetyl-CoA-carboxylase ligase	255	11157..11921	44_GENE_10
**10933**	**11**	**CGL2_10933011**	Transcriptional acitvator, Baf family	259	11921..12697	44_GENE_11
**10933**	**12**	**CGL2_10933012**	Hypothetical protein	60	complement(12739..12918)	44_GENE_12
**10933**	**13**	**CGL2_10933013**	Diguanylate cyclase/phosphodiesterase with PAS/PA(	1449	complement(12985..17331)	44_GENE_13
**10933**	**13**	**CGL2_10933013**	PAS/PAC sensor protein	1449	complement(12985..17331)	44_GENE_13
**10933**	**15**	**CGL2_10933015**	check gene	91	complement(18072..18344)	44_GENE_14
**10933**	**16**	**CGL2_10933016**	Probable secretion protein (HlyD)	329	18642..19628	44_GENE_15
**10933**	**17**	**CGL2_10933017**	Putative outer membrane efflux protein	417	19748..20998	44_GENE_16
**10933**	**18**	**CGL2_10933018**	Malate dehydrogenase (EC 1.1.1.27)	320	21209..22168	44_GENE_17
**10933**	**19**	**CGL2_10933019**	Protein of unknown function	195	22249..22833	44_GENE_18
**10933**	**20**	**CGL2_10933020**	Protein of unknown function	209	22826..23452	44_GENE_19
**10933**	**21**	**CGL2_10933021**	ABC transporter, ATP-binding protein (EC 3.6.3.25)	248	23460..24203	44_GENE_20
**10933**	**22**	**CGL2_10933022**	RNA polymerase, sigma 54 subunit, RpoN	486	24203..25660	44_GENE_21
**10933**	**23**	**CGL2_10933023**	Sigma 54 modulation protein/ribosomal protein S30E	176	25707..26234	44_GENE_22
**10933**	**24**	**CGL2_10933024**	Putative P-loop ATPase protein family	288	26262..27125	44_GENE_23
**10933**	**25**	**CGL2_10933025**	Protein of unknown function	195	27128..27712	44_GENE_24
**10933**	**26**	**CGL2_10933026**	Chorismate synthase (EC 4.2.3.5)	401	27752..28954	44_GENE_25
**10933**	**27**	**CGL2_10933027**	Shikimate kinase (EC 2.7.1.71)	183	28947..29495	44_GENE_26
**10933**	**28**	**CGL2_10933028**	3-dehydroquinate synthetase (EC 4.2.3.4)	364	29535..30626	44_GENE_27
**10933**	**29**	**CGL2_10933029**	Putative GAF sensor protein	421	30658..31920	44_GENE_28
**10933**	**30**	**CGL2_10933030**	Hypothetical protein	123	31966..32334	44_GENE_29
**10933**	**31**	**CGL2_10933031**	Conserved protein of unknown function	260	32340..33119	44_GENE_30
**10933**	**32**	**CGL2_10933032**	Probable acetyltransferase	134	33160..33561	44_GENE_31
**10933**	**33**	**CGL2_10933033**	Putative GTP cyclohydrolase I	149	complement(33626..34072)	44_GENE_32
**10933**	**34**	**CGL2_10933034**	transposase	173	34165..34683	44_GENE_33
10933tRNA		**CGL2_10933R001**	TRNA Val TAC		complement(34822..34897)	
**10933**	**35**	**CGL2_10933035**	4-hydroxybenzoyl-CoA thioesterase	148	complement(34891..35334)	44_GENE_34
10933tRNA		**CGL2_10933R002**	TRNA Asp GTC		35492..35568	
**10933**	**36**	**CGL2_10933036**	Putative MotA/TolQ/ExbB proton channel	223	35891..36559	96_GENE_3

**10933**	**37**	**CGL2_10933037**	Putative biopolymer transport protein ExbD/TolR	148	36550..36993	96_GENE_4
**10933**	**38**	**CGL2_10933038**	Probable TonB family protein	278	37003..37836	96_GENE_5
**10933**	**39**	**CGL2_10933039**	Putative TolB protein	447	37829..39169	96_GENE_6
**10933**	**40**	**CGL2_10933040**	Putative TPR-domain containing protein	274	39214..40035	96_GENE_7
**10933**	**41**	**CGL2_10933041**	DNA mismatch repair protein MutL	634	40102..42003	96_GENE_8
**10933**	**42**	**CGL2_10933042**	tRNA delta(2)-isopentenylpyrophosphate transferase	294	41988..42869	96_GENE_9
**10933**	**43**	**CGL2_10933043**	Methylthioadenosine phosphorylase (EC 2.4.2.28)	300	43012..43911	96_GENE_10
**10933**	**44**	**CGL2_10933044**	Putative carbohydrate kinase, PfkB family	313	43940..44878	96_GENE_11
**10933**	**45**	**CGL2_10933045**	Putative TPR-domain containing protein	254	44890..45651	96_GENE_12
**10933**	**46**	**CGL2_10933046**	Conserved protein of unknown function	335	45707..46711	96_GENE_13
**10933**	**47**	**CGL2_10933047**	Protein of unknown function	335	46757..47761	96_GENE_14
10933tRNA		**CGL2_10933R003**	TRNA Thr CGT		47852..47926	
**10933**	**48**	**CGL2_10933048**	Hypothetical protein	412	complement(47999..49234)	96_GENE_15
		Strain variant path	Strain variant path			Strain variant path
**11276**	**210**	**CGL2_11276210**	Probable Holliday junction resolvase	450	complement(229981..231330)	96_GENE_16
**11276**	**210a**	**CGL2_11276210a**	Hypothetical protein	115	complement(229625..229969)	
**11276**	**209a**	**CGL2_11276209a**	Hypothetical protein	126	complement(229169..229546)	
**11276**	**209**	**CGL2_11276209**	Hypothetical protein	89	complement(228885..229151)	
**11276**	**208**	**CGL2_11276208**	Hypothetical protein	242	complement(228142..228867)	
**11276**	**207**	**CGL2_11276207**	Hypothetical protein	220	complement(227480..228139)	
		Strain variant path	Strain variant path			Strain variant path
**11276**	**206**	**CGL2_11276206**	Conjugal transfer protein, TraA	976	complement(224503..227430)	81_GENE_26
**11276**	**205**	**CGL2_11276205**	Hypothetical protein	134	223999..224400	81_GENE_25
**11276**	**204**	**CGL2_11276204**	Hypothetical protein	129	223451..223837	81_GENE_24
**11276**	**203**	**CGL2_11276203**	Hypothetical protein	132	222865..223260	81_GENE_22
**11276**	**202**	**CGL2_11276202**	Putative heavy metal efflux protein, CzcA	1060	219665..222844	81_GENE_21
**11276**	**201**	**CGL2_11276201**	Putative heavy metal efflux protein, CzcB	419	218409..219665	81_GENE_20
**11276**	**200**	**CGL2_11276200**	Putative heavy metal efflux protein, CzcC	408	217193..218416	81_GENE_19
**11276**	**199**	**CGL2_11276199**	Hypothetical protein	67	216013..216213	81_GENE_17
**11276**	**198**	**CGL2_11276198**	Hypothetical protein	103	215717..216025	81_GENE_16
**11276**	**197**	**CGL2_11276197**	Putative integrase	388	214554..215717	
**10580**	**1**	**CGL2_10580001**	Conserved protein of unknown function	106	423..740	527_GENE_7
**10580**	**2**	**CGL2_10580002**	Conserved protein of unknown function	129	complement(746..1132)	527_GENE_6
**10580**	**3**	**CGL2_10580003**	Hypothetical protein	67	1101..1301	527_GENE_5a
**10580**	**4**	**CGL2_10580004**	Hypothetical protein	81	complement(1295..1537)	527_GENE_4a
**10580**	**5**	**CGL2_10580005**	Putative phage integrase	345	complement(1555..2589)	527_GENE_4
**10580**	**6**	**CGL2_10580006**	transposase	346	3050..4087	527_GENE_3
**10580**	**7**	**CGL2_10580007**	integrase fragment, INT 22C	474	4580..6001	527_GENE_2
**11276**	**196**	**CGL2_11276196**	Conserved protein of unknown function	322	complement(213327..214292)	6_GENE_66

**11276**	**195**	**CGL2_11276195**	Hypothetical protein	49	complement(213095..213241)	6_GENE_65
**11276**	**194**	**CGL2_11276194**	Putative peptidase, M23B family	332	complement(211567..212562)	6_GENE_64
**11276**	**193**	**CGL2_11276193**	Putative ATP-dependent Clp protease, ATPase subun	813	complement(208771..211209)	6_GENE_62
**11276**	**192**	**CGL2_11276192**	O-sialoglycoprotein endopeptidase (EC 3.4.24.57)	345	complement(207711..208745)	6_GENE_61
**11276**	**191**	**CGL2_11276191**	Arginyl-tRNA synthetase (EC 6.1.1.19)	595	complement(205810..207594)	6_GENE_60
**11276**	**190**	**CGL2_11276190**	Queuine tRNA-ribosyltransferase (EC 2.4.2.29)	378	complement(204672..205805)	6_GENE_59
**11276**	**189**	**CGL2_11276189**	Preprotein translocase, YajC subunit	96	complement(204385..204672)	6_GENE_58
**11276**	**188**	**CGL2_11276188**	SecD export membrane protein	524	complement(202762..204333)	6_GENE_57
**11276**	**187**	**CGL2_11276187**	SecF export membrane protein	321	complement(201742..202704)	6_GENE_56
**11276**	**186**	**CGL2_11276186**	Hypothetical protein	37	complement(201613..201723)	6_GENE_55
**11276**	**185**	**CGL2_11276185**	Putative RecJ exonuclease	549	complement(199953..201599)	6_GENE_54
**11276**	**184**	**CGL2_11276184**	GTP pyrophosphokinase (EC 2.7.6.5)	762	complement(197708..199993)	6_GENE_53
**11276**	**183**	**CGL2_11276183**	Probable formate dehydrogenase accessory protein	302	complement(196799..197704)	6_GENE_52
**11276**	**182**	**CGL2_11276182**	Hypothetical protein	83	complement(196515..196763)	6_GENE_51
**11276**	**181**	**CGL2_11276181**	Putative transcriptional regulator, TetR family	231	complement(195720..196412)	6_GENE_50
**11276**	**180**	**CGL2_11276180**	Probable cytochrome c-554	200	complement(195121..195720)	6_GENE_49
**11276**	**179**	**CGL2_11276179**	Putative secretion protein HlyD	317	complement(194171..195121)	6_GENE_48
**11276**	**178**	**CGL2_11276178**	Drug resistance transporter, EmrB/QacA family	527	complement(192588..194168)	6_GENE_47
**11276**	**177**	**CGL2_11276177**	Putative outer membrane efflux protein	464	complement(191197..192588)	6_GENE_46
**11276**	**176**	**CGL2_11276176**	Putative secretion protein HlyD	330	complement(190143..191132)	6_GENE_45
**11276**	**175**	**CGL2_11276175**	Drug resistance transporter, EmrB/QacA family	540	complement(188514..190133)	6_GENE_44
**11276**	**174**	**CGL2_11276174**	Heat-inducible transcription repressor HrcA	352	complement(187240..188295)	6_GENE_43
**11276**	**173**	**CGL2_11276173**	Putative GrpE protein	189	complement(186661..187227)	6_GENE_42
**11276**	**172**	**CGL2_11276172**	Chaperone DnaK	640	complement(184649..186568)	6_GENE_41
**11276**	**171**	**CGL2_11276171**	Chaperone DnaJ	372	complement(183376..184491)	6_GENE_40
**11276**	**170**	**CGL2_11276170**	Conserved protein of unknown function	287	complement(182506..183366)	6_GENE_39
**11276**	**169**	**CGL2_11276169**	Protein of unknown function	148	complement(182058..182501)	6_GENE_38
**11276**	**168**	**CGL2_11276168**	Putative thiopurine S-methyltransferase	201	complement(181422..182024)	6_GENE_37
**11276**	**167**	**CGL2_11276167**	Putative cobalbumin biosynthesis enzyme	179	complement(180872..181408)	6_GENE_36
**11276**	**166**	**CGL2_11276166**	Nicotinate-nucleotide--dimethylbenzimidazole phospl	363	complement(179724..180812)	6_GENE_35
**11276**	**165**	**CGL2_11276165**	Putative cobalamin-5-phosphate synthase (CobS)	260	complement(178945..179724)	6_GENE_34
**11276**	**164**	**CGL2_11276164**	Conserved hypothetical protein	136	178477..178884	6_GENE_33
**11276**	**163**	**CGL2_11276163**	Conserved hypothetical protein	84	178226..178477	6_GENE_32
**11276**	**162**	**CGL2_11276162**	Protein of unknown function	128	177856..178239	6_GENE_31
**11276**	**161**	**CGL2_11276161**	Hypothetical protein	148	177351..177794	6_GENE_30
**11276**	**160**	**CGL2_11276160**	Hypothetical protein	108	177008..177331	6_GENE_29
**11276**	**159**	**CGL2_11276159**	Aldo/keto reductase (EC 1.1.1.65)	337	175692..176702	6_GENE_27
**11276**	**158**	**CGL2_11276158**	Putative phosphoglycerate mutase family protein	222	complement(174799..175464)	6_GENE_26
**11276**	**157**	**CGL2_11276157**	Cobyrinic acid a,c-diamide synthase (CbiA)	471	complement(173391..174803)	6_GENE_25
**11276**	**156**	**CGL2_11276156**	Putative precorrin-8X methylmutase (CbiC)	334	complement(172337..173338)	6_GENE_24
**11276**	**155**	**CGL2_11276155**	Isocitrate dehydrogenase (NAD+) (EC 1.1.1.41)	336	complement(170991..171998)	6_GENE_23

**11276**	**154**	**CGL2_11276154**	Probable isocitrate dehydrogenase (NADP)	116	complement(170597..170944)	6_GENE_22
11276tRNA		**CGL2_11276R003**	TRNA Phe GAA		170086..170161	
**11276**	**153**	**CGL2_11276153**	Putative bacterial surface antigen (D15)	781	complement(167449..169791)	6_GENE_20
**11276**	**152**	**CGL2_11276152**	Putative outer membrane protein (OmpH)	200	complement(166771..167370)	6_GENE_19
**11276**	**151**	**CGL2_11276151**	UDP-3-O-[3-hydroxymyristoyl] glucosamine N-acyltr	350	complement(165722..166771)	6_GENE_18
**11276**	**150**	**CGL2_11276150**	Beta-hydroxyacyl-(Acyl-carrier-protein) dehydratase	173	complement(165235..165753)	6_GENE_17
**11276**	**149**	**CGL2_11276149**	UDP-N-acetylglucosamine acyltransferase (EC 2.3.1.	287	complement(164375..165235)	6_GENE_16
**11276**	**148**	**CGL2_11276148**	Conserved protein of unknown function	296	complement(163495..164382)	6_GENE_15
**11276**	**147**	**CGL2_11276147**	Putative oxidoreductase family protein	321	complement(162539..163501)	6_GENE_14
**11276**	**146**	**CGL2_11276146**	Lipid A disaccharide synthase (LpxB) (EC 2.4.1.182)	405	complement(161314..162528)	6_GENE_13
**11276**	**145**	**CGL2_11276145**	glycosyltransferase	357	complement(160204..161274)	6_GENE_12
**11276**	**144**	**CGL2_11276144**	ABC transporter, ATP-binding protein, MsbA family	600	complement(158351..160150)	6_GENE_11
**11276**	**143**	**CGL2_11276143**	Putative 3-deoxy-D-manno-octulosonic-acid transfer	457	complement(156981..158351)	6_GENE_10
**11276**	**142**	**CGL2_11276142**	Tetraacyldisaccharide-1-P 4'-kinase (EC 2.7.1.130)	357	complement(155911..156981)	6_GENE_9
**11276**	**141**	**CGL2_11276141**	Putative lipid A biosynthesis acyltransferase	309	complement(154985..155911)	6_GENE_8
**11276**	**140**	**CGL2_11276140**	Hypothetical protein	569	complement(153271..154977)	6_GENE_7
**11276**	**139**	**CGL2_11276139**	Putative heptosyltransferase family protein	402	complement(152075..153280)	6_GENE_6
**11276**	**138**	**CGL2_11276138**	Putative heptosyltransferase family protein	371	complement(150967..152079)	6_GENE_5
**11276**	**137**	**CGL2_11276137**	glycosyltransferase	250	complement(150218..150967)	6_GENE_4
**11276**	**136**	**CGL2_11276136**	Conserved protein of unknown function	283	complement(149331..150179)	6_GENE_3
**11276**	**135**	**CGL2_11276135**	transposase	330	complement(148342..149331)	68_GENE_28
**11276**	**134**	**CGL2_11276134**	Conserved hypothetical protein	273	complement(147388..148206)	68_GENE_27
**11276**	**133**	**CGL2_11276133**	Putative glycosyl transferase, family 2	343	complement(146173..147201)	68_GENE_26
**11276**	**132**	**CGL2_11276132**	Lipopolysaccharide heptosyltransferase II	332	complement(145060..146055)	68_GENE_25
**11276**	**131**	**CGL2_11276131**	Histidinol-phosphate phosphatase	199	complement(144460..145056)	68_GENE_24
**11276**	**130**	**CGL2_11276130**	Conserved protein of unknown function	61	complement(144285..144467)	68_GENE_23
**11276**	**129**	**CGL2_11276129**	Probable lipopolysaccharide heptosyltransferase II	344	complement(143227..144258)	68_GENE_22
**11276**	**128**	**CGL2_11276128**	Putative heptosyltransferase family protein	360	complement(142145..143224)	68_GENE_21
**11276**	**127**	**CGL2_11276127**	Putative polysaccharide deacetylase	240	complement(141426..142145)	68_GENE_20
**11276**	**126**	**CGL2_11276126**	Putative glycosyl transferase, family 1	389	complement(140238..141404)	68_GENE_19
**11276**	**125**	**CGL2_11276125**	Protein of unknown function	95	complement(139954..140238)	68_GENE_18
**11276**	**124**	**CGL2_11276124**	Probable diacylglycerol kinase	255	139173..139937	68_GENE_17
**11276**	**123**	**CGL2_11276123**	Protein of unknown function	289	138088..138954	68_GENE_16
**11276**	**122**	**CGL2_11276122**	Adenosylmethionine--8-amino-7-oxononanoate amin	444	136682..138013	68_GENE_15
**11276**	**121**	**CGL2_11276121**	Conserved protein of unknown function	295	complement(135687..136571)	68_GENE_14
**11276**	**120**	**CGL2_11276120**	Putative O-antigen polymerase	453	complement(134251..135609)	68_GENE_12
**11276**	**119**	**CGL2_11276119**	Putative PilT protein-like	351	complement(133199..134251)	68_GENE_11
**11276**	**118**	**CGL2_11276118**	2-C-methyl-D-erythritol 4-phosphate cytidylyltransfe	426	complement(131922..133199)	68_GENE_10
**11276**	**117**	**CGL2_11276117**	Serine O-acetyltransferase (EC 2.3.1.30)	234	complement(131211..131912)	68_GENE_9
**11276**	**116**	**CGL2_11276116**	Putative phosphoesterase, PA-phosphatase related	183	complement(130645..131193)	68_GENE_8
**11276**	**115**	**CGL2_11276115**	Conserved protein of unknown function	546	complement(129008..130645)	68_GENE_7

**11276**	**114**	**CGL2_11276114**	Putative glycosyl transferase, family 9	403	complement(127800..129008)	68_GENE_6
**11276**	**113**	**CGL2_11276113**	Putative glycosyl transferase	270	complement(126978..127787)	68_GENE_5
**11276**	**112**	**CGL2_11276112**	Probable glycosyl transferase, family 39	504	complement(125467..126978)	68_GENE_4
**11276**	**111**	**CGL2_11276111**	Phosphoribosylaminoimidazolecarboxamide formyltra	522	complement(123827..125392)	68_GENE_3
**11276**	**110**	**CGL2_11276110**	Phosphoribosylglycinamide synthetase (EC 6.3.4.13)	425	complement(122527..123801)	68_GENE_2
**11276**	**108**	**CGL2_11276108**	Putative translation factor (SUA5)	204	complement(121916..122527)	68_GENE_1
**11276**	**107**	**CGL2_11276107**	Protein of unknown function	117	complement(121357..121707)	8_GENE_1
**11276**	**106**	**CGL2_11276106**	Putative methyltransferase	189	complement(120732..121298)	8_GENE_2
**11276**	**105**	**CGL2_11276105**	Phosphopantetheine adenylyltransferase (EC 2.7.7.3	166	complement(120235..120732)	8_GENE_3
**11276**	**104**	**CGL2_11276104**	Aspartate aminotransferase (EC 2.6.1.1)	403	complement(119027..120235)	8_GENE_4
**11276**	**103**	**CGL2_11276103**	Conserved protein of unknown function	112	complement(118581..118916)	8_GENE_6
**11276**	**102**	**CGL2_11276102**	Putative ABC transporter, ATP-binding protein	247	117833..118573	8_GENE_7
**11276**	**101**	**CGL2_11276101**	Lipoprotein releasing system, transmembrane protei	412	116598..117833	8_GENE_8
**11276**	**100**	**CGL2_11276100**	Lysyl-tRNA synthetase (EC 6.1.1.6)	507	115078..116598	8_GENE_9
**11276**	**99**	**CGL2_11276099**	Translation initiation factor, aIF-2BI/5-methylthioribo	350	114012..115061	8_GENE_10
**11276**	**98**	**CGL2_11276098**	Putative glycogen debranching enzyme	680	111910..113949	8_GENE_11
**11276**	**97**	**CGL2_11276097**	Ferrochelatase (EC 4.99.1.1)	356	110825..111892	8_GENE_12
**11276**	**96**	**CGL2_11276096**	Putative permease, YjgP/YjgQ family	362	109726..110811	8_GENE_13
**11276**	**95**	**CGL2_11276095**	Putative permease, YjgP/YjgQ family	378	108593..109726	8_GENE_14
**11276**	**94**	**CGL2_11276094**	TRNA-i(6)A37 modification enzyme (MiaB)	468	107156..108559	8_GENE_15
**11276**	**93**	**CGL2_11276093**	Protein of unknown function	134	106759..107160	8_GENE_16
**11276**	**92**	**CGL2_11276092**	Putative SAM-dependent methyltransferases	191	106214..106786	8_GENE_17
**11276**	**91**	**CGL2_11276091**	Protein of unknown function	208	105494..106117	8_GENE_18
**11276**	**90**	**CGL2_11276090**	Putative cytochrome c, class I	133	complement(104820..105218)	8_GENE_20
**11276**	**89**	**CGL2_11276089**	transposase	446	complement(103323..104660)	8_GENE_21
**11276**	**88**	**CGL2_11276088**	Aspartate aminotransferase (EC 2.6.1.17)	399	complement(101673..102869)	8_GENE_22
**11276**	**87**	**CGL2_11276087**	Protein of unknown function	215	complement(101029..101673)	8_GENE_23
**11276**	**86**	**CGL2_11276086**	Homoserine dehydrogenase (EC 1.1.1.3)	443	complement(99579..100907)	8_GENE_24
**11276**	**85**	**CGL2_11276085**	Threonine synthase (EC 4.2.3.1)	353	complement(98521..99579)	8_GENE_25
**11276**	**84**	**CGL2_11276084**	Probable phosphoglycerate mutase	400	complement(97297..98496)	8_GENE_26
**11276**	**83**	**CGL2_11276083**	Aspartate kinase (EC 2.7.2.4)	412	complement(96068..97303)	8_GENE_27
**11276**	**82**	**CGL2_11276082**	2-isopropylmalate synthase (EC 2.3.3.13)	544	complement(94444..96075)	8_GENE_28
**11276**	**81**	**CGL2_11276081**	Acyl-(Acyl-carrier-protein)--UDP-N-acetylglucosamin	270	complement(93639..94448)	8_GENE_29
**11276**	**80**	**CGL2_11276080**	Putative integration host factor, alpha subunit HimA	98	complement(93318..93611)	8_GENE_30
**11276**	**79**	**CGL2_11276079**	Putative transcriptional regulator, MerR family	113	complement(92878..93216)	8_GENE_31
11276tRNA		**CGL2_11276R002**	TRNA Pro GGG		complement(92808..92884)	
**11276**	**78**	**CGL2_11276078**	Acid phosphatase (SurE) (EC 3.1.3.2)	266	complement(91906..92703)	8_GENE_32
**11276**	**77**	**CGL2_11276077**	Protein of unknown function	189	complement(91329..91895)	8_GENE_33
**11276**	**76**	**CGL2_11276076**	Cysteinyl-tRNA synthetase, class Ia (EC 6.1.1.16)	490	complement(89870..91339)	8_GENE_34
**11276**	**75**	**CGL2_11276075**	Putative TRNA/rRNA methyltransferase (SpoU)	192	complement(89139..89714)	8_GENE_35
**11276**	**74**	**CGL2_11276074**	Putative thioredoxin family protein	214	complement(88357..88998)	8_GENE_36

**11276**	**73**	**CGL2_11276073**	NAD+ synthase (EC 6.3.5.1)	592	complement(86582..88357)	8_GENE_37
**11276**	**72**	**CGL2_11276072**	3,4-dihydroxy-2-butanone 4-phosphate synthase/GT	400	complement(85328..86527)	8_GENE_38
**11276**	**71**	**CGL2_11276071**	6,7-dimethyl-8-ribityllumazine synthase (EC 2.5.1.9)	161	complement(84822..85304)	8_GENE_39
**11276**	**70**	**CGL2_11276070**	Transcription antitermination factor (NusB)	168	complement(84319..84822)	8_GENE_40
**11276**	**69**	**CGL2_11276069**	Protein of unknown function	166	complement(83822..84319)	8_GENE_41
**11276**	**68**	**CGL2_11276068**	Adenylosuccinate lyase (EC 4.3.2.2)	437	complement(82418..83728)	8_GENE_42
**11276**	**67**	**CGL2_11276067**	Phosphoribosylaminoimidazole-succinocarboxamide s	239	complement(81644..82360)	8_GENE_43
**11276**	**66**	**CGL2_11276066**	Phosphoribosylformylglycinamidine synthetase (PurS	98	complement(81364..81657)	8_GENE_43a
**11276**	**65**	**CGL2_11276065**	Protein of unknown function	446	complement(80024..81361)	8_GENE_45
**11276**	**64**	**CGL2_11276064**	Putative asparaginase	308	complement(79087..80010)	8_GENE_46
**11276**	**63**	**CGL2_11276063**	Acetate kinase (EC 2.7.2.1)	374	77809..78930	8_GENE_47
**11276**	**62**	**CGL2_11276062**	Putative methyltransferase	224	complement(77008..77679)	8_GENE_48
**11276**	**61**	**CGL2_11276061**	Hypothetical protein	970	74052..76961	8_GENE_49
**11276**	**60**	**CGL2_11276060**	TonB protein	222	73316..73981	8_GENE_50
**11276**	**59**	**CGL2_11276059**	TonB system transport protein, ExbD	137	72884..73294	8_GENE_51
**11276**	**58**	**CGL2_11276058**	biopolymer transport protein, ExbB	230	72116..72805	8_GENE_52
**11276**	**57**	**CGL2_11276057**	Putative ABC transporter permease protein	372	70783..71898	8_GENE_53
**11276**	**56**	**CGL2_11276056**	Putative ABC transporter permease protein	378	69650..70783	8_GENE_54
**11276**	**55**	**CGL2_11276055**	BC transporter ATP-binding protein (EC 3.6.3.25)	592	67875..69650	8_GENE_55
**11276**	**54**	**CGL2_11276054**	Secretion protein (HlyD)	301	66983..67885	8_GENE_56
**11276**	**53**	**CGL2_11276053**	Putative outer membrane efflux protein	483	65508..66956	8_GENE_57
**11276**	**52**	**CGL2_11276052**	Putative cytochrome c, class I	110	65093..65422	8_GENE_58
**11276**	**51**	**CGL2_11276051**	Fructose-6-phosphate phosphoketolase (EC 4.1.2.22	788	complement(62484..64847)	8_GENE_59
**11276**	**50**	**CGL2_11276050**	Fructose-bisphosphate aldolase (EC 4.1.2.13)	305	complement(61517..62431)	8_GENE_60
**11276**	**49**	**CGL2_11276049**	Dihydrolipoyl dehydrogenase (EC 1.8.1.4)	461	complement(60057..61439)	8_GENE_61
**11276**	**48**	**CGL2_11276048**	Conserved protein of unknown function	304	59095..60006	8_GENE_62
**11276**	**47**	**CGL2_11276047**	Glycogen phosphorylase (EC 2.4.1.1)	862	56395..58980	8_GENE_63
**11276**	**46**	**CGL2_11276046**	Phosphoglucomutase, alpha-D-glucose phosphate-sp	547	54729..56369	8_GENE_64
**11276**	**45**	**CGL2_11276045**	Putative transposase	362	complement(53117..54202)	8_GENE_65
**11276**	**44**	**CGL2_11276044**	Probable cytochrome c oxidase, subunit I (cbb-type)	460	complement(51308..52687)	8_GENE_66
**11276**	**43**	**CGL2_11276043**	Putative major facilitator superfamily protein	510	complement(49355..50884)	8_GENE_67
**11276**	**42**	**CGL2_11276042**	Hypothetical protein	110	48898..49227	8_GENE_68
**11276**	**41**	**CGL2_11276041**	ABC transporter, ATP-binding protein	555	47215..48879	8_GENE_69
**11276**	**40**	**CGL2_11276040**	Conserved protein of unknown function	95	complement(46239..46523)	8_GENE_70
**11276**	**39**	**CGL2_11276039**	Conserved protein of unknown function	132	complement(45752..46147)	141_GENE_2
**11276**	**38**	**CGL2_11276038**	Putative OmpA family protein	265	complement(44871..45665)	141_GENE_3
**11276**	**37**	**CGL2_11276037**	Conserved hypothetical protein	227	43720..44400	141_GENE_5
**11276**	**36**	**CGL2_11276036**	Putative metallophosphoesterase	251	complement(42818..43570)	141_GENE_6
**11276**	**35**	**CGL2_11276035**	Conserved hypothetical protein	1175	39220..42744	141_GENE_7
**11276**	**34**	**CGL2_11276034**	DNA repair exonuclease	410	37991..39220	141_GENE_8
**11276**	**33**	**CGL2_11276033**	Conserved hypothetical protein	195	37000..37584	141_GENE_10

**11276**	**32**	**CGL2_11276032**	Conserved hypothetical protein	238	36287..37000	141_GENE_11
**11276**	**31**	**CGL2_11276031**	Conserved hypothetical protein	390	35111..36280	141_GENE_12
**11276**	**30**	**CGL2_11276030**	Conserved hypothetical protein	571	33372..35084	141_GENE_13
**11276**	**29**	**CGL2_11276029**	serine protease	326	32301..33278	141_GENE_14
**11276**	**28**	**CGL2_11276028**	Protein of unknown function	195	31682..32266	69_GENE_29
**11276**	**27**	**CGL2_11276027**	Hypothetical protein	406	30404..31621	69_GENE_28
**11276**	**26**	**CGL2_11276026**	Hypothetical protein	217	29711..30361	69_GENE_26
**11276**	**25**	**CGL2_11276025**	Protein of unknown function	313	complement(28269..29207)	69_GENE_25
**11276**	**24**	**CGL2_11276024**	Conserved hypothetical protein	176	complement(27599..28126)	69_GENE_24
**11276**	**23**	**CGL2_11276023**	Conserved hypothetical protein	267	complement(26722..27522)	69_GENE_21
**11276**	**22**	**CGL2_11276022**	transposase	316	complement(25796..26743)	69_GENE_20
**11276**	**21**	**CGL2_11276021**	transposase	138	complement(25402..25815)	69_GENE_19
**11276**	**20**	**CGL2_11276020**	Conserved hypothetical protein	373	complement(24041..25159)	69_GENE_18
**11276**	**19**	**CGL2_11276019**	Hypothetical protein	120	complement(23424..23783)	69_GENE_17
**11276**	**18**	**CGL2_11276018**	Conserved hypothetical protein	59	complement(22360..22536)	69_GENE_15
**11276**	**17**	**CGL2_11276017**	Conserved hypothetical protein	103	21919..22227	69_GENE_14
**11276**	**16**	**CGL2_11276016**	universal stress protein	287	21011..21871	69_GENE_13
**11276**	**15**	**CGL2_11276015**	sulfate permease	491	19521..20993	69_GENE_12
**11276**	**14**	**CGL2_11276014**	Hypothetical protein	217	complement(18013..18663)	69_GENE_11
		Strain variant path	Strain variant path			Strain variant path
**11276**	**13a**	**CGL2_11276013a**	Probable transposase	130	complement(17227..17616)	
11276tRNA		**CGL2_11276R001**	TRNA Arg CCT		17023..17099	
**11276**	**13**	**CGL2_11276013**	Hypothetical protein	56	16792..16959	167_GENE_11
**11276**	**11**	**CGL2_11276011**	N-acetylmuramoyl-L-alanine amidase (EC 3.5.1.28)	437	15391..16701	167_GENE_10
**11276**	**11**	**CGL2_11276011**	N-acetylmuramoyl-L-alanine amidase (EC 3.5.1.28)	437	15391..16701	167_GENE_10
**11276**	**10**	**CGL2_11276010**	Conserved protein of unknown function	132	complement(14672..15067)	167_GENE_8
**11276**	**9**	**CGL2_11276009**	Protein of unknown function	714	complement(12502..14643)	167_GENE_7
**11276**	**8**	**CGL2_11276008**	Putative peptidase M50	243	complement(11699..12427)	167_GENE_6
**11276**	**7**	**CGL2_11276007**	Tryptophanyl-tRNA synthetase (EC 6.1.1.2)	355	complement(10577..11641)	167_GENE_5
**11276**	**6**	**CGL2_11276006**	Acetyl-coenzyme A carboxylase carboxyl transferase	296	complement(9579..10466)	167_GENE_4
**11276**	**5**	**CGL2_11276005**	DNA polymerase III, alpha subunit (EC 2.7.7.7)	1201	5955..9557	167_GENE_3
**11276**	**4**	**CGL2_11276004**	Putative UvrD/REP helicase	649	3953..5899	167_GENE_2
**11276**	**3**	**CGL2_11276003**	Conserved hypothetical protein	288	complement(2946..3809)	824_GENE_4
**11276**	**2**	**CGL2_11276002**	Methyl accepting chemotaxis transducer	714	complement(547..2688)	824_GENE_2
**11276**	**1**	**CGL2_11276001**	probable transposase	128	complement(40..422)	824_GENE_1

Strain variant contigs:

			**Strain variant linked to 11277 at ~142,000**			
**8975**	**2**	**CGL2_08975002**	Protein of unknown function	213	complement(1682..2320)	865_GENE_2

**8975**	**1 (=Vaı**	**CGL2_08975001**	Protein of unknown function (cyt 572, full on 7508)	409	46..1272	865_GENE_1
**cyt572**	**A**	**CGL2_11277153A**	Cyt572 variant			
**cyt572**	**B**	**CGL2_11277153B**	Cyt572 variant			
**cyt572**	**C**	**CGL2_11277153C**	Cyt572 variant			
**cyt572**	**D**	**CGL2_11277153D**	Cyt572 variant			
**cyt572**	**E**	**CGL2_11277153E**	Cyt572 variant			
			**Low depth, links via 7252 to 10391 = 11386 at ~16,000**			
**7252**	**1**	**CGL2_07252001**	Hypothetical protein	126	434..811	
**7252**	**2**	**CGL2_07252002**	Hypothetical protein	110	811..1140	
**7252**	**3**	**CGL2_07252003**	Hypothetical protein	69	1140..1346	
**7252**	**4**	**CGL2_07252004**	Hypothetical protein	195	1524..2108	
**7252**	**5**	**CGL2_07252005**	Hypothetical protein	70	2068..2278	
**8492**	**1**	**CGL2_08492001**	Protein of unknown function	366	complement(403..1500)	402_GENE_5
**9769**	**3**	**CGL2_09769003**	DNA primase	1133	complement(1720..5118)	402_GENE_4
**9769**	**2**	**CGL2_09769002**	ATPase involved in ABC transporters	221	complement(585..1247)	402_GENE_2
**9769**	**1**	**CGL2_09769001**	Hypothetical protein	165	complement(58..552)	402_GENE_1
			**Strain variant linked to 11386-11389**			
**11389**	**59a**	**CGL2_11389059a**	Hypothetical protein	161	61743..62225	
**11389**	**58a**	**CGL2_11389058a**	Hypothetical protein	239	61019..61735	
**11389**	**57a**	**CGL2_11389057a**	putative type III restriction-modification system, Res	939	58210..61026	
**11389**	**56a**	**CGL2_11389056a**	putative type III restriction-modification system, me	856	55640..58207	
**11389**	**55a**	**CGL2_11389055a**	Hypothetical protein	241	54708..55430	
**11389**	**54a**	**CGL2_11389054a**	Hypothetical protein	128	54230..54613	
**11389**	**53a**	**CGL2_11389053a**	Hypothetical protein	674	52196..54217	
**11389**	**52a**	**CGL2_11389052a**	Hypothetical protein	301	51267..52169	
**11389**	**51a**	**CGL2_11389051a**	Hypothetical protein	204	50342..50953	
**11389**	**50a**	**CGL2_11389050a**	Putative integrase	404	49123..50334	
**11389**	**49**	**CGL2_11389049**	SsrA-binding protein	163	48031..48519	26_GENE_24
**11389**	**48**	**CGL2_11389048**	Leucyl aminopeptidase (EC 3.4.11.1)	533	46367..47965	26_GENE_25
**11389**	**47**	**CGL2_11389047**	ExsB protein (succinoglycan synthase?)	241	45526..46248	26_GENE_26
**11389**	**46**	**CGL2_11389046**	Putative radical activating enzyme	212	44885..45520	26_GENE_27
**11389**	**45**	**CGL2_11389045**	Protein of unknown function	231	44144..44836	26_GENE_28
**11389**	**44**	**CGL2_11389044**	DNA gyrase, A subunit	816	41593..44040	26_GENE_29
**11389**	**43**	**CGL2_11389043**	DNA gyrase, B subunit (EC 5.99.1.3)	818	39081..41534	26_GENE_30
**11389**	**42**	**CGL2_11389042**	DNA polymerase III, beta chain (EC 2.7.7.7)	387	37847..39007	26_GENE_31
**11389**	**41**	**CGL2_11389041**	Chromosomal replication initiator protein (DnaA) ORI	465	36431..37825	26_GENE_32
			**Strain variant linked to 10851 ~20 kb or 11346 ~21 kb**			
**11185**	**1**	**CGL2_11185001**	Probable Bacterial Conjugative Coupling Protein, Trw	128	complement(311..694)	529 GENE 5
**11185**	**2**	**CGL2_11185002**	Hypothetical protein	258	complement(1186..1959)	490_GENE_1
**11185**	**3**	**CGL2_11185003**	Hypothetical protein	75	complement(2248..2472)	490_GENE_2
**11185**	**4**	**CGL2_11185004**	Hypothetical protein	116	complement(2610..2957)	490_GENE_3

**11185**	**5**	**CGL2_11185005**	Hypothetical protein	112	3294..3629	490_GENE_5
**11185**	**6**	**CGL2_11185006**	Transposase	326	complement(3801..4778)	490_GENE_6
**11185**	**7**	**CGL2_11185007**	Hypothetical protein	46	4924..5061	490_GENE_7
**11185**	**8**	**CGL2_11185008**	Transposase	267	complement(5328..6128)	
**11185**	**9**	**CGL2_11185009**	Putative DNA helicase	455	complement(6166..7530)	
**11185**	**10**	**CGL2_11185010**	Hypothetical protein	107	complement(7530..7850)	
			**Strain variant linked to 11068 at ~130,000**			
**9240**	**5**	**CGL2_09240005**	Integrase	316	complement(2150..3097)	435_GENE_1
**9240**	**4**	**CGL2_09240004**	**Hypothetical protein**	69	complement(1944..2150)	435_GENE_2
**9240**	**3**	**CGL2_09240003**	**Hypothetical protein**	40	complement(1841..1960)	435_GENE_3
**9240**	**2**	**CGL2_09240002**	**transcriptional activator**	274	complement(527..1348)	435_GENE_4
**9240**	**1**	**CGL2_09240001**	**transposase**	59	308..484	435_GENE_5
**11402**	**1**	**CGL2_11402001**	transcriptional activator	97	427..717	435_GENE_6
**6010**	**2**	**CGL2_06010002**	autoinducer biosynthetic protein/LuxI-type quorum s	234	complement(403..1104)	435_GENE_7
**9250**	**1**	**CGL2_09250001**	Conserved hypothetical protein	354	935..1996	435_GENE_8
			**Strain variant linked to 9240**			
**10101**	**1**	**CGL2_10101001**	transposase	164	complement(24..515)	234_GENE_9
**10101**	**2**	**CGL2_10101002**	pyoverdine chromophore precursor synthetase	1125	993..4367	234_GENE_8
**10101**	**3**	**CGL2_10101003**	transposase (single)	248	complement(4394..5137)	234_GENE_7
**10065**	**6**	**CGL2_10065006**	Hypothetical protein	180	complement(5248..5787)	234_GENE_6
**10065**	**5**	**CGL2_10065005**	acetolactate synthase, large subunit	587	complement(3286..5046)	234_GENE_5
**10065**	**4**	**CGL2_10065004**	acetolactate synthase small subunit	183	complement(2738..3286)	234_GENE_4
**10065**	**3**	**CGL2_10065003**	Conserved hypothetical protein	137	complement(1862..2272)	234_GENE_3
**10065**	**2**	**CGL2_10065002**	biotin synthesis protein/methyltransferase	228	complement(1179..1862)	234_GENE_2
**10065**	**1**	**CGL2_10065001**	DNA binding protein	165	complement(602..1096)	234_GENE_1
			**Strain variant linked to 10933 end (alternative to link to end of 11276)**			
**10608**	**11**	**CGL2_10608011**	Hypothetical protein	412	12940..14175	96_GENE_15
**10608**	**10**	**CGL2_10608010**	Probable Holliday junction resolvase	450	complement(11153..12502)	96_GENE_16
**10608**	**9**	**CGL2_10608009**	Hypothetical protein	79	complement(10900..11136)	96_GENE_17
**10608**	**8**	**CGL2_10608008**	Protein of unknown function	127	complement(10524..10904)	96_GENE_18
**10608**	**7**	**CGL2_10608007**	Probable mobilization protein, MobD	242	complement(9786..10511)	96_GENE_19
**10608**	**6**	**CGL2_10608006**	Hypothetical protein	215	complement(9138..9782)	637_GENE_2
**10608**	**5**	**CGL2_10608005**	Conjugal transfer protein, TraA	976	complement(6161..9088)	81_GENE_26
**10608**	**4**	**CGL2_10608004**	Hypothetical protein	135	5654..6058	81_GENE_25
**10608**	**3**	**CGL2_10608003**	Hypothetical protein	129	5106..5492	81_GENE_24
**10608**	**2**	**CGL2_10608002**	Hypothetical protein	132	4520..4915	81_GENE_22
**10608**	**1**	**CGL2_10608001**	Putative heavy metal efflux protein, CzcA	1060	1320..4499	81_GENE_21
			**Unclear how linked except for common (divergent) region to 11276/11608**			
**11216**	**30**	**CGL2_11216030**	ABC transporter, ATP-binding protein (EC 3.6.3.25)	138	31763..32176	115_GENE_22
**11216**	**29**	**CGL2_11216029**	Hypothetical protein	98	31507..31800	115_GENE_21
**11216**	**28**	**CGL2_11216028**	Putative resolvase	198	30554..31147	115_GENE_20

**11216**	**27**	**CGL2_11216027**	transposase	451	complement(29065..30417)	115_GENE_19
**11216**	**26**	**CGL2_11216026**	Hypothetical protein	108	28621..28944	115_GENE_18
**11216**	**25**	**CGL2_11216025**	Hypothetical protein	104	28121..28432	115_GENE_17
**11216**	**24**	**CGL2_11216024**	Hypothetical protein	94	27844..28125	115_GENE_16
**11216**	**23**	**CGL2_11216023**	Hypothetical protein	57	27647..27817	115_GENE_15
**11216**	**22**	**CGL2_11216022**	GGDEF/EAL domain-containing protein	404	26334..27545	115_GENE_13
**11216**	**21**	**CGL2_11216021**	Hypothetical protein	385	25087..26241	115_GENE_11
**11216**	**20**	**CGL2_11216020**	Hypothetical protein	104	complement(24472..24783)	115_GENE_10
**11216**	**19**	**CGL2_11216019**	DNA primase	1121	complement(21107..24469)	115_GENE_9
**11216**	**18**	**CGL2_11216018**	Putative transcriptional regulator	102	20592..20897	115_GENE_8
**11216**	**17**	**CGL2_11216017**	Hypothetical protein	75	20378..20602	115_GENE_7
**11216**	**16**	**CGL2_11216016**	Hypothetical protein	135	complement(19888..20292)	115_GENE_6
**11216**	**15a**	**CGL2_11216015a**	Hypothetical protein	189	complement(19280..19846)	
**11216**	**15**	**CGL2_11216015**	mobilization protein, MobD	253	complement(18550..19308)	115_GENE_4
**11216**	**14**	**CGL2_11216014**	Hypothetical protein	237	complement(17780..18490)	115_GENE_3
**11216**	**13a**	**CGL2_11216013a**	Hypothetical protein	85	complement(17202..17456)	
**11216**	**13**	**CGL2_11216013**	Conjugal transfer protein, TraB	701	complement(15097..17199)	115_GENE_1
**11216**	**12**	**CGL2_11216012**	Conjugal transfer protein, TraA	976	complement(12167..15094)	81_GENE_26
**11216**	**11**	**CGL2_11216011**	Hypothetical protein	135	11660..12064	81_GENE_25
**11216**	**10**	**CGL2_11216010**	Hypothetical protein	129	11112..11498	81_GENE_24
**11216**	**9**	**CGL2_11216009**	Hypothetical protein	132	10526..10921	81_GENE_22
**11216**	**8**	**CGL2_11216008**	Putative heavy metal efflux protein, CzcA	1060	7326..10505	81_GENE_21
**11216**	**7**	**CGL2_11216007**	Putative heavy metal efflux protein, CzcB	419	6070..7326	81_GENE_20
**11216**	**6**	**CGL2_11216006**	Putative heavy metal efflux protein, CzcC	408	4854..6077	81_GENE_19
**11216**	**5**	**CGL2_11216005**	Hypothetical protein	67	3674..3874	81_GENE_17
**11216**	**4**	**CGL2_11216004**	Hypothetical protein	103	3378..3686	81_GENE_16
**11216**	**3**	**CGL2_11216003**	Recombinase	373	2260..3378	81_GENE_15
**11216**	**2**	**CGL2_11216002**	Putative integrase	248	complement(1269..2012)	
**11216**	**1**	**CGL2_11216001**	Hypothetical protein	86	complement(673..930)	
		**Strain variant linked loosely internally to 11276 at ~18,000**				
**10638**	**10**	**CGL2_10638010**	Conserved hypothetical protein	168	complement(19400..19903)	69_GENE_10
**10638**	**9**	**CGL2_10638009**	transposase	242	complement(18602..19327)	69_GENE_9
**10638**	**8**	**CGL2_10638008**	restriction endonuclease	285	complement(17519..18373)	69_GENE_8
**10638**	**7**	**CGL2_10638007**	RNA helicase	1717	complement(12316..17466)	69_GENE_7
**10638**	**6**	**CGL2_10638006**	Hypothetical protein	59	complement(11945..12121)	69_GENE_5
**10638**	**5**	**CGL2_10638005**	transposase	254	complement(10886..11647)	69_GENE_4
**10638**	**4**	**CGL2_10638004**	Hypothetical protein	142	complement(10461..10886)	69_GENE_3
**10638**	**3**	**CGL2_10638003**	Conserved hypothetical protein	67	complement(9940..10140)	69_GENE_2
**10638**	**2**	**CGL2_10638002**	Superfamily II DNA/RNA helicases	957	complement(7079..9949)	69_GENE_1
**10638**	**1**	**CGL2_10638001**	Superfamily II DNA/RNA helicases	1309	complement(3153..7079)	

Small, unplaced genome fragments, likely to be *Leptospirillum* group II:

**8298**	**6**	**CGL3_08298006**	Hypothetical protein	148	complement(2927..3370)	898_GENE_1
**8298**	**5**	**CGL3_08298005**	mobilization protein, MobC	146	complement(2155..2592)	898_GENE_2
**8298**	**4**	**CGL3_08298004**	mobilization protein, MobD	220	complement(1468..2127)	898_GENE_3
**8298**	**3**	**CGL3_08298003**	mobilization protein MobE	207	complement(848..1468)	898_GENE_5
**8298**	**2**	**CGL3_08298002**	Hypothetical protein	110	complement(516..845)	898_GENE_6
**8298**	**1**	**CGL3_08298001**	DNA helicase/TrwC	179	complement(29..565)	898_GENE_7
**6909**	**5**	**CGU_06909005**	Conserved hypothetical protein	106	complement(1386..1703)	888_GENE_5
**6909**	**4**	**CGU_06909004**	Hypothetical protein	110	complement(1057..1386)	888_GENE_4
**6909**	**3**	**CGU_06909003**	Hypothetical protein	157	complement(549..1019)	888_GENE_3
**6909**	**1**	**CGU_06909001**	Hypothetical protein	153	complement(31..488)	
**9588**	**1**	**CGLU_09588001**	helicase	844	25..2556	830_GENE_5
**9588**	**2**	**CGLU_09588002**	Protein of unknown function	136	2691..3098	830_GENE_4
**9588**	**3**	**CGLU_09588003**	Hypothetical protein	164	complement(3290..3781)	830_GENE_3
**9588**	**4**	**CGLU_09588004**	Conserved hypothetical protein	383	complement(3808..4956)	830_GENE_2
**9588**	**5**	**CGLU_09588005**	Hypothetical protein	128	complement(4972..5355)	830_GENE_1
**9588**	**6**	**CGLU_09588006**	Hypothetical protein	120	complement(5349..5708)	
**9588**	**7**	**CGLU_09588007**	Hypothetical protein	166	complement(5736..6233)	
**9144**	**1**	**CGLU_09144001**	RNA helicase	294	complement(78..959)	759_GENE_5
**9144**	**2**	**CGLU_09144002**	Hypothetical protein	399	complement(1003..2199)	759_GENE_4
**9144**	**3**	**CGLU_09144003**	Hypothetical protein	87	complement(2306..2566)	759_GENE_2
**9144**	**4**	**CGLU_09144004**	ATP-dependent protease La	381	complement(2566..3708)	759_GENE_1
**9074**	**7**	**CGLU_09074007**	Hypothetical protein	84	3258..3508	
**9074**	**6**	**CGLU_09074006**	thiol-disulfide interchange protein, DsbG	328	1996..2979	728_GENE_6
**9074**	**5**	**CGLU_09074005**	Hypothetical protein	89	1730..1996	728_GENE_5
**9074**	**4**	**CGLU_09074004**	Hypothetical protein	99	1327..1623	728_GENE_4
**9074**	**3**	**CGLU_09074003**	Hypothetical protein	67	1055..1255	728_GENE_3
**9074**	**2**	**CGLU_09074002**	Conserved hypothetical protein	209	396..1022	728_GENE_2
**9074**	**1**	**CGLU_09074001**	Conserved hypothetical protein	55	248..412	728_GENE_1
**9375**	**1**	**CGLU_09375001**	Putative phosphoribulokinase	987	246..3206	745_GENE_2
**9375**	**2**	**CGLU_09375002**	Hypothetical protein	87	complement(3314..3574)	745_GENE_3
**9375**	**3**	**CGLU_09375003**	Hypothetical protein	477	complement(3613..5043)	745_GENE_4
**8925**	**1**	**CGL2_08925001**	Probable plasmid stabilization protein, StbA	332	complement(28..1023)	

**8925**	**2**	**CGL2_08925002**	DotB (type II secretion)	367	complement(1300..2400)	569_GENE_5
**8925**	**3**	**CGL2_08925003**	TraI	275	complement(2400..3224)	569_GENE_4
**8758**	**4**	**CGL2_08758001**	sensory box/GGDEF domain/EAL domain protein	346	558..1595	569_GENE_3
**8758**	**5**	**CGL2_08758002**	Protein of unknown function	235	1901..2605	569_GENE_2
**8758**	**6**	**CGL2_08758003**	Putative chemotaxis protein MotB	244	2665..3396	569_GENE_1
**9428**	**4**	**CGLU_09428004**	Hypothetical protein	300	complement(3206..4105)	
**9428**	**3**	**CGLU_09428003**	Hypothetical protein	126	complement(2568..2945)	
**9428**	**2**	**CGLU_09428002**	Putative DNA primase	511	complement(1036..2568)	673_GENE_1
**9428**	**1**	**CGLU_09428001**	Hypothetical protein	231	complement(1..693)	673_GENE_3
**8706**	**4**	**CGLU_08706004**	Hypothetical protein	66	complement(3127..3324)	673_GENE_4
**8706**	**3**	**CGLU_08706003**	Hypothetical protein	100	complement(2377..2676)	673_GENE_5
**8706**	**3**	**CGLU_08706002**	Putative mobilization relaxase, MobA	272	complement(1562..2377)	673_GENE_6
**8706**	**1**	**CGLU_08706001**	Putative type IV secretory pathway VirD4	449	complement(161..1507)	
**9446**	**1**	**CGLU_09446001**	Hypothetical protein	151	complement(70..522)	
**9446**	**2**	**CGLU_09446002**	Conserved hypothetical protein	114	complement(909..1250)	
**9446**	**3**	**CGLU_09446003**	Putative hydrolase	281	complement(1373..2215)	
**9446**	**4**	**CGLU_09446004**	Putative SAM-dependent methyltransferase	227	complement(2251..2931)	
**9446**	**5**	**CGLU_09446005**	Hypothetical protein	111	complement(2990..3322)	
**9446**	**6**	**CGLU_09446006**	Hypothetical protein	103	complement(3513..3821)	592_GENE_1
**9446**	**7**	**CGLU_09446007**	Hypothetical protein	110	complement(4005..4334)	592_GENE_2
**9446**	**8**	**CGLU_09446008**	Putative N-acetyl transferase	144	complement(4429..4860)	592_GENE_3
**8858**	**4**	**CGLU_08858004**	Hypothetical protein	170	1298..1807	592_GENE_4
**8858**	**3**	**CGLU_08858003**	Putative hydrolase	95	949..1233	592_GENE_5
**8858**	**2**	**CGLU_08858002**	Hypothetical protein	54	677..838	592_GENE_6
**8858**	**1**	**CGLU_08858001**	Putative N-acetyl transferase	162	192..677	592_GENE_7
**9713**	**5**	**CGL3_09713005**	Conserved hypothetical protein	517	complement(4991..6541)	
**9713**	**4**	**CGL3_09713004**	Probable DNA primase	302	complement(3611..4516)	
**9713**	**3**	**CGL3_09713003**	Hypothetical protein	105	complement(2736..3050)	811_GENE_1
**9713**	**2**	**CGL3_09713002**	Putative mobilization relaxase, MobA	292	complement(1861..2736)	811_GENE_2
**9713**	**1**	**CGL3_09713001**	Putative Type IV secretion system, VirD4	599	complement(69..1865)	811_GENE_3
**9378**	**1**	**CGL2_09378001**	Hypothetical protein	398	complement(502..1695)	684_GENE_1
**9378**	**2**	**CGL2_09378002**	Hypothetical protein	425	complement(1695..2969)	684_GENE_2
**9378**	**3**	**CGL2_09378003**	Conserved protein of unknown function	238	complement(2969..3682)	684_GENE_3
**9378**	**4**	**CGL2_09378004**	Conserved protein of unknown function	161	complement(3682..4164)	684_GENE_4
**9378**	**5**	**CGL2_09378005**	Conserved protein of unknown function	136	complement(4221..4628)	684_GENE_5
**9378**	**6**	**CGL2_09378006**	Conserved protein of unknown function	195	complement(4638..5222)	684_GENE_6

**9792**	**8**	**CGLU_09792008**	Conserved hypothetical protein	71	complement(6197..6409)	478_GENE_8
**9792**	**7**	**CGLU_09792007**	Hypothetical protein	76	complement(5983..6210)	478_GENE_7
**9792**	**6**	**CGLU_09792006**	Protein of unknown function	97	complement(5181..5471)	478_GENE_6
**9792**	**5**	**CGLU_09792005**	Hypothetical protein	93	4893..5171	478_GENE_5
**9792**	**4**	**CGLU_09792004**	Putative plasmid helicase TrwC	1153	1435..4893	478_GENE_4
**9792**	**3**	**CGLU_09792003**	Hypothetical protein	64	1239..1430	478_GENE_3
**9792**	**2**	**CGLU_09792002**	Hypothetical protein	58	1051..1224	478_GENE_2
**9792**	**1**	**CGLU_09792001**	Hypothetical protein	287	191..1051	478_GENE_1
