# Supplementary material for: Population Genomic Analysis of Strain Variation in Leptospirillum Group II Bacteria Involved in Acid Mine Drainage Formation
Source: PLoS Biol. 2008 Jul 22;6(7):e177. doi: 10.1371/journal.pbio.0060177 (PMC2475542; doi:10.1371/journal.pbio.0060177)
Supplement: Supplementary file 5 [file pbio.0060177.st002.pdf]

	Phred quality score cutoff				
	20	*25*	*30*	*40*	*50*
Total synonymous SNPs	786	734	707	640	547
Total replacement SNPs	701	644	606	551	461
Total intergenic SNPs (nt + indels)	534	392	325	247	207
Total indels in genes	1058	612	452	259	177
Total indels in intergenic regions	304	188	127	71	60
Replicated synonymous SNPs	458	440	427	387	333
Replicated replacement SNPs	335	325	313	282	228
Replicated intergenic SNPs (nt + indels)	119	111	101	83	73
Replicated indels in coding regions	65	57	42	37	24
Replicated indels in intergenic regions	22	20	17	10	6
Total SNPs	3079	2382	2090	1697	1392
Total replicated SNPs	977	933	883	789	658
Total sites in assembly	2671936	2669185	2665839	2422942	2402552
Overall SNP density (%)	0.12	0.09	0.08	0.07	0.06
Replicated SNP density (%)	0.04	0.03	0.03	0.03	0.03
% of all SNPs that are replicated	32	39	42	46	47
Ratio of replacement/synonymous SNPs	0.89	0.88	0.86	0.86	0.84
Density of synonymous SNPs (%)	0.029	0.027	0.027	0.026	0.023
Density of replacement SNPs (%)	0.026	0.024	0.023	0.023	0.019
Synonymous % of total	25.5	30.8	33.8	37.7	39.3
Replacement % of total	22.8	27.0	29.0	32.5	33.1
Intergenic % of total	17.3	16.5	15.6	14.6	14.9
Indels in coding regions % of total	34.4	25.7	21.6	15.3	12.7

Table S2: Classification of all SNPs observed in the 5-way CG *Leptospirillum* group II assembly. Increasing Phred quality score cutoffs were used to examine the effect of sequencing error on the distribution of SNPs between site classes.
