# Supplementary material for: Population Genomic Analysis of Strain Variation in Leptospirillum Group II Bacteria Involved in Acid Mine Drainage Formation
Source: PLoS Biol. 2008 Jul 22;6(7):e177. doi: 10.1371/journal.pbio.0060177 (PMC2475542; doi:10.1371/journal.pbio.0060177)
Supplement: Supplementary file 6 [file pbio.0060177.st003.pdf]

Table S3

List of single nucleotide base pair positions on contigs that have been scaffolded into an ~700 Kb region that span the origin of replication. Yellow and pink highlights indicate SNPs that are linked on at least one read or by mate pair information. Blue highlights indicate the locations of significantly discrepant reads, most of which have ~100% sequence identity to reads of the UBA-type *Leptospirillum* group II ("L. rubarum"). Grey highlights indicate SNPs present in only a single read.
Jazz (2004) indicates positions on the scaffolds reported previously by Tyson et al. (2004).
Phrap (2006) indicates positions on the scaffolds reported here and shown in Fig. 2. Red bars separate contigs.

Contig/Position	Jazz (2004)	Phrap (2006)	Functional annotation	Base 1	Base 2	Base 3	#1	#2	#3	Subst.	Notes	Strain	
**Contig 11277**													
7167	42_11	5wayCG_LeptoII_Contig11277_GENE_11	Chaperone protein HscA	G	*		14	1			shift		
11012	42_17	5wayCG_LeptoII_Contig11277_GENE_16	Protein of Unknown Function	G	A		15	1		T/A			
11270	42_18	5wayCG_LeptoII_Contig11277_GENE_17	Pseudouridylate synthase (4.2.1.70)	T	*		13	1			frameshift		
12764	42_20	5wayCG_LeptoII_Contig11277_GENE_19	Glycerol 3-phosphate dehydrogenase (NADP)	G	A		15	2		Y/H	split		
13836	42_21	5wayCG_LeptoII_Contig11277_GENE_20	Probable phosphoribosyltransferase	G	*		10	1			synonymous		
13843	42_21	5wayCG_LeptoII_Contig11277_GENE_20	Probable phosphoribosyltransferase	G	*		10	1			synonymous		
14211	42_21	5wayCG_LeptoII_Contig11277_GENE_20	Probable phosphoribosyltransferase	G	*		12	1			split		
15250	42_22	5wayCG_LeptoII_Contig11277_GENE_21	Protein of unknown function	C	*		13	1			shift		
18675	42_24	5wayCG_LeptoII_Contig11277_GENE_23	Putative phosphatidylethanolamine binding protein	C	*		14	1			frameshift		
18759	42_24	5wayCG_LeptoII_Contig11277_GENE_23	Putative phosphatidylethanolamine binding protein	G	*		13	1			split		
20600	42_26	5wayCG_LeptoII_Contig11277_GENE_25	Protein of unknown function	C	A		10	2		P/H			
26922	42_33	5wayCG_LeptoII_Contig11277_GENE_32	Putative peptidase M16	C	*		6	1			split/shift		
27338	42_34	5wayCG_LeptoII_Contig11277_GENE_33	Putative peptidase M16	C	*		10	1			split		
34153			intergenic	G	A		2	1			intergenic		
35202	42_41	5wayCG_LeptoII_Contig11277_GENE_40	Hypothetical protein	C	A		6	2		A/S			
40087_40090	21_5	5wayCG_LeptoII_Contig11277_GENE_46	Hypothetical protein	CGGG	*GG*		10	1			split		
41999			intergenic	A	C		21	1			intergenic		
42599	21_8	5wayCG_LeptoII_Contig11277_GENE_48	Leucyl-tRNA synthetase (6.1.1.4)	C	T		17	5		P/L			
50,720	pre 21_16		intergenic	T	G		23	2			intergenic	strain 1	strain 1
50737	pre 21_16		intergenic	T	C		22	2			intergenic	strain 1	strain 1
51075	21_17	5wayCG_LeptoII_Contig11277_GENE_57	Probable ATPase, PP-loop superfamily	C	*		16	1		L/S	split		
54816			intergenic	T	*		7	1			intergenic		
55955-55958	21_21	5wayCG_LeptoII_Contig11277_GENE_62	Anthranilate synthases component I (EC 4.1.3.27)	****	AGTG		8	1			split		
57427	21_22	5wayCG_LeptoII_Contig11277_GENE_64	Put. branched chain amino acid aminotransferase	*	C		10	1			split		
62690	21_25	5wayCG_LeptoII_Contig11277_GENE_67	Protein of unknown function	A	*		11	1			shift/split		
63469-63471	21_26	5wayCG_LeptoII_Contig11277_GENE_68	Protein of unknown function	TTC	***		12	1			synonymous		
67132			intergenic	G	C		10	2			intergenic		
71020	21_34	5wayCG_LeptoII_Contig11277_GENE_73	L-2,4-diaminobutyric acid acetyltransferase	C	T		12	1			synonymous		
72369	21_36	5wayCG_LeptoII_Contig11277_GENE_75	Ectoine synthase	T	*		11	1		L/M	split		
75164	21_41	5wayCG_LeptoII_Contig11277_GENE_80	Prob. sodium/calcium exchanger membrane region	C	T		6	1			synonymous		
75976	21_42	5wayCG_LeptoII_Contig11277_GENE_81	Probable transporter protein	C	*		6	1			shift/split		
78812	21_44	5wayCG_LeptoII_Contig11277_GENE_83	Putative proteasome component	G	A		5	1			synonymous		
79125	21_44	5wayCG_LeptoII_Contig11277_GENE_83	Putative proteasome component	A	*		7	1			shift		
81868	21_48	5wayCG_LeptoII_Contig11277_GENE_87	Putative proteasome component	G	A		2	4			synonymous	strain 2	strain 2
81976	21_48	5wayCG_LeptoII_Contig11277_GENE_87	Putative proteasome component	C	A		3	2			synonymous	strain 2	strain 2
85125	21_52	5wayCG_LeptoII_Contig11277_GENE_90	Putative band 7 family protein	T	C		11	1			synonymous		
85936	21_53	5wayCG_LeptoII_Contig11277_GENE_91	Protein of unknown function	C	T		7	3			synonymous	strain 3	strain 3
86170	21_53	5wayCG_LeptoII_Contig11277_GENE_91	Protein of unknown function	C	A		9	2			synonymous	strain 3	strain 3
86254	21_53	5wayCG_LeptoII_Contig11277_GENE_91	Protein of unknown function	T	C		10	2			synonymous	strain 3	strain 3
87381	21_56	5wayCG_LeptoII_Contig11277_GENE_93	Protein of unknown function	G	A		14	1		G/R			
87757	21_57	5wayCG_LeptoII_Contig11277_GENE_94	Alcohol dehydrogenase (EC 1.1.1.1)	C	T		9	1			synonymous		
88418	21_57	5wayCG_LeptoII_Contig11277_GENE_94	Alcohol dehydrogenase (EC 1.1.1.1)	G	T		12	2		T/N		strain 4	strain 4
88794	(22_25)		intergenic	C	A		13	5			intergenic	strain 4	strain 4
91102	21_59	5wayCG_LeptoII_Contig11277_GENE_96	Putative acriflavin resistance protein	T	C		9	1			synonymous	strain 4	strain 4
91148	21_59	5wayCG_LeptoII_Contig11277_GENE_96	Putative acriflavin resistance protein	T	C		12	1		I/ V		strain 4	strain 4
111776			Protein of unknown Function								XYG69022.b1		
112111	32_36	5wayCG_LeptoII_Contig11277_GENE_114	Protein of unknown function	C	T		7	1		R/G			
113249			intergenic	A	G		15	1			intergenic		
114540			intergenic	G	C		10	1			intergenic		
116279	32_33	5wayCG_LeptoII_Contig11277_GENE_117	Protein of unknown function	T	G		13	1			synonymous		
116471			intergenic	G	C		12	1			intergenic		
118656	32_28	5wayCG_LeptoII_Contig11277_GENE_121	Protein of unknown function	A	C		14	1			synonymous		
119686	32_26	5wayCG_LeptoII_Contig11277_GENE_123	Protein of unknown function	G	A		9	1			synonymous		
119692	32_26	5wayCG_LeptoII_Contig11277_GENE_123	Protein of unknown function	G	A		8	1			synonymous		
121277	32_7	5wayCG_LeptoII_Contig11277_GENE_144	Probable thiamine biosynthesis protein	A	*		2	2			split		
125885	32_21	5wayCG_LeptoII_Contig11277_GENE_130	Put. periplasmic signal transduction histidine kinase	C	*		7	1			frameshift		
127655	32_20	5wayCG_LeptoII_Contig11277_GENE_131	Putative response regulator receiver protein	T	*		8	1			split		
129887	32_18	5wayCG_LeptoII_Contig11277_GENE_133	Prob. crossover junction endodeoxyribonuclease	G	T		11	1		G/*			
131217	32_16	5wayCG_LeptoII_Contig11277_GENE_135	Holliday junction DNA helicase (RuvB)	G	A		16	1		G/E			
132431	32_14	5wayCG_LeptoII_Contig11277_GENE_137	Protein of unknown function	G	A		7	1					
135036	21_21	5wayCG_LeptoII_Contig11277_GENE_62	intergenic	C	T		12	3			intergenic	strain 5	strain 5
135155	32_9	5wayCG_LeptoII_Contig11277_GENE_142	Protein of unknown function	A	G		15	3		T/A		strain 5	strain 5
135379	32_9	5wayCG_LeptoII_Contig11277_GENE_142	Protein of unknown function	G	A		13	1		M/I		strain 6	strain 6
135973			intergenic	T	C		12	1			intergenic	strain 6	strain 6
136596	32_7	5wayCG_LeptoII_Contig11277_GENE_144	Probable thiamine biosynthesis protein	C	T		21	1			synonymous	strain 5	strain 5
136962	32_7	5wayCG_LeptoII_Contig11277_GENE_144	Probable thiamine biosynthesis protein	T	A		14	5			synonymous	strain 5,7	strain 5,7

136993	32_7	5wayCG_Leptoll_Contig11277_GENE_144	Probable thiamine biosynthesis protein	G	A		14	5		N/D		strain 5,7	strain 5,7
137088	32_7	5wayCG_Leptoll_Contig11277_GENE_144	Probable thiamine biosynthesis protein	C	G		3	12			synonymous	strain 5,7	strain 5,7
137136	32_7	5wayCG_Leptoll_Contig11277_GENE_144	Probable thiamine biosynthesis protein	C	T		2	13			synonymous	strain 5,7	strain 5,7
137178	32_7	5wayCG_Leptoll_Contig11277_GENE_144	Probable thiamine biosynthesis protein	G	A		2	12			synonymous	strain 5,7	strain 5,7
137233	32_7	5wayCG_Leptoll_Contig11277_GENE_144	Probable thiamine biosynthesis protein	T	C		3	11		H/Y+		strain 5,7	strain 5,7
137484	32-6	5wayCG_Leptoll_Contig11277_GENE_145	Probable radical SAM family protein	C	T		13	1		P/S		strain 5	strain 5
137633	32_6	5wayCG_Leptoll_Contig11277_GENE_145	Probable radical SAM family protein	T	C		6	4			synonymous	strain 5	strain 5
137892	32_6	5wayCG_Leptoll_Contig11277_GENE_145	Probable radical SAM family protein	T	C		11	4		W/R		strain 5,6	strain 5,6
138247	32_6	5wayCG_Leptoll_Contig11277_GENE_145	Probable radical SAM family protein	C	A		7	3		T/N		strain 6	strain 6
138458			intergenic	T	C		9	4			intergenic	strain 6,5	strain 6,5
138679	32_5	5wayCG_Leptoll_Contig11277_GENE_146	Conserved protein of unknown function	G	T		6	6			synonymous	strain 6,5	strain 6,5
138738	32_5	5wayCG_Leptoll_Contig11277_GENE_146	Conserved protein of unknown function	A	G		5	6		L/S		strain 6,5	strain 6,5
138860	32_5	5wayCG_Leptoll_Contig11277_GENE_146	Conserved protein of unknown function	T	C		7	7			synonymous	strain 6,5	strain 6,5
138898	32_5	5wayCG_Leptoll_Contig11277_GENE_146	Conserved protein of unknown function	A	C		8	6		A/S		strain 6,5	strain 6,5
138917	32_5	5wayCG_Leptoll_Contig11277_GENE_146	Conserved protein of unknown function	G	T		10	4			synonymous	strain 6,5	strain 6,5
139022	32_5	5wayCG_Leptoll_Contig11277_GENE_146	Conserved protein of unknown function	G	A		11	8			synonymous	strain 6,5	strain 6,5
139026			intergenic	A	T		9	7			intergenic	strain 6,5	strain 6,5
139117-139789	32_4, 32_3	5wayCG_Leptoll_Contig11277_GENES_147-149	Probable oxidoreductase FAD/NAD(P)-binding protein and heterosulfide reductase								Rec strain 6		**Recombinant, but not UBA type**
139602	32_3	5wayCG_Leptoll_Contig11277_GENE_148	heterodisulfide reductase	G	A		14	2		C/R	strain 6		
142360	630_3	5wayCG_Leptoll_Contig11277_GENE_150	Protein of unknown function	C	T		7	1		H/Y			
143212			intergenic	*	A		7	1			synonymous		
143228	630_4	5wayCG_Leptoll_Contig11277_GENE_151	Transposase	C	*		7	2			synonymous	strain 8	strain 8
143663	630_4	5wayCG_Leptoll_Contig11277_GENE_151	Transposase	A	C		5	2			synonymous	strain 8	strain 8
143697	630_4	5wayCG_Leptoll_Contig11277_GENE_151	Transposase	T	G		6	2		S/A		strain 8	strain 8
143771	630_4	5wayCG_Leptoll_Contig11277_GENE_151	Transposase	C	T		4	2			synonymous	strain 8	strain 8
143870	630_4	5wayCG_Leptoll_Contig11277_GENE_151	Transposase	C	G		3	4			synonymous	strain 8	strain 8
144109	630_4	5wayCG_Leptoll_Contig11277_GENE_151	Transposase	G	A		4	2		G/D		strain 8	strain 8
144648	630_5	5wayCG_Leptoll_Contig11277_GENE_152	Protein of unknown function	C	A		7	1		A/D		strain 9	strain 9
144738-144745	865_1/630_6	5wayCG_Leptoll_Contig11277_GENE_153	Protein of unknown function	gttgttttgcttg<br>aataactacc	********g*tt*<br>**t***t***		1	8			inserted block in single read		
144962	630_6	5wayCG_Leptoll_Contig11277_GENE_153	Newly described cytochrome	T	C		9	2			synonymous	strain 9	strain 9
145398	630_6	5wayCG_Leptoll_Contig11277_GENE_153	Newly described cytochrome	C	T		11	1		G/E		strain 8	strain 8
145423	630_6	5wayCG_Leptoll_Contig11277_GENE_153	Newly described cytochrome	C	G	T	8	2	1	G/R/R		strain 9,8	strain 9,8
146000	630_6	5wayCG_Leptoll_Contig11277_GENE_153	Newly described cytochrome	G	T		2	1			synonymous	strain 9	strain 9
146005-146006	630_6	5wayCG_Leptoll_Contig11277_GENE_153	Newly described cytochrome	GT	TA		2	1		NKK/KQN		strain 9	strain 9
153030	600_1	5wayCG_Leptoll_Contig11277_GENE_158	DNA helicase	T	G		2	1		T/P			
158619			intergenic	T	C		3	1			intergenic		
158808-158809	185_4	5wayCG_Leptoll_Contig11277_GENE_166	hypothetical protein	GA	CC		3	3		G/S		strain 10	strain 10
159439	185_6	5wayCG_Leptoll_Contig11277_GENE_168	hypothetical protein	C	A		9	1		G/V			strain 10
161133-161157			intergenic	agcctttctagg<br>cggttttgatggg	*g********a**<br>***t***g**gg*		2	2			intergenic		strain 10
166469	185_19	5wayCG_Leptoll_Contig11277_GENE_180	hypothetical protein	A	G		4	1		F/S			
166529	185_19	5wayCG_Leptoll_Contig11277_GENE_180	hypothetical protein	A	C		5	1		G/V			
167006	185_19	5wayCG_Leptoll_Contig11277_GENE_180	hypothetical protein	A	T		6	2		F/Y			
168152	185_21	5wayCG_Leptoll_Contig11277_GENE_181	hypothetical protein	G	A		8	1		S/L			
168698			intergenic	A	G		2	2			intergenic		
168767			intergenic	A	G		2	1			intergenic		
168959			intergenic	A	G		1	1			intergenic		
169007			intergenic	A	G		1	1			intergenic		
169510	265_1	??	Protein of Unknown Function	C	T		3	1		E/K			
172327-172334			transposase	ttatgat<br>aaaa	w/ *******<br>*aaa	w/ *******<br>*aa*	2	2	5		synonymous		
178581	173_9	5wayCG_Leptoll_Contig11277_GENE_190	Conserved protein of unknown function	T	A		11	1		K/N			
179187	173_7	5wayCG_Leptoll_Contig11277_GENE_191	hypothetical protein	***	CCC		7	1			synonymous		
188278	374_7	5wayCG_Leptoll_Contig11277_GENE_203	Protein of unknown function	T	C		13	1			synonymous		
189975	374_9	5wayCG_Leptoll_Contig11277_GENE_205	Conserved hypothetical protein	G	T		6	1		P/Q			
197543	4_93	5wayCG_Leptoll_Contig11277_GENE_214	Heat shock protein HslU	C	T		16	1		G/D		strain 11	strain 11
199000	4_91	5wayCG_Leptoll_Contig11277_GENE_216	Putative phage integrase family protein	*	G		7	1			split		
200273	4_90	5wayCG_Leptoll_Contig11277_GENE_218	Putaive glucose-inhibited division protein A	C	T		7	2		V/M		strain 12	strain 12
200696	4_90	5wayCG_Leptoll_Contig11277_GENE_218	Putaive glucose-inhibited division protein A	A	G		4	4		S/R		strain 12	strain 12
201058	4_90	5wayCG_Leptoll_Contig11277_GENE_218	Putaive glucose-inhibited division protein A	C	T		6	4			synonymous	strain 12	strain 12
201075	4_89	5wayCG_Leptoll_Contig11277_GENE_219	DNA topoisomerase (EC 5.99.1.2)	T	C		6	5		I/V		strain 12	strain 12
201260	4_89	5wayCG_Leptoll_Contig11277_GENE_219	DNA topoisomerase (EC 5.99.1.2)	A	T		6	3		H/L		strain 12	strain 12
201523	4_89	5wayCG_Leptoll_Contig11277_GENE_219	DNA topoisomerase (EC 5.99.1.2)	A	G		8	4			synonymous	strain 12	strain 12
201526	4_89	5wayCG_Leptoll_Contig11277_GENE_219	DNA topoisomerase (EC 5.99.1.2)	T	C		7	4			synonymous	strain 12	strain 12
202370	4_89	5wayCG_Leptoll_Contig11277_GENE_219	DNA topoisomerase (EC 5.99.1.2)	G	C		7	1		R/T		strain 12	strain 12
202775	4_89	5wayCG_Leptoll_Contig11277_GENE_219	DNA topoisomerase (EC 5.99.1.2)	G	A		6	6		L/P		strain 12	strain 12
203485	4_89	5wayCG_Leptoll_Contig11277_GENE_219	DNA topoisomerase (EC 5.99.1.2)	G	C		8	3			synonymous	strain 12	strain 12
203491	4_89	5wayCG_Leptoll_Contig11277_GENE_219	DNA topoisomerase (EC 5.99.1.2)	A	G		5	3			synonymous	strain 12	strain 12
203774	4_88	5wayCG_Leptoll_Contig11277_GENE_220	DNA processing protein DprA	G	A		5	9			synonymous	strain 12	strain 12
204475	4_88	5wayCG_Leptoll_Contig11277_GENE_220	DNA processing protein DprA	G	A		8	6		Y/H		strain 12	strain 12
204634	4_87	5wayCG_Leptoll_Contig11277_GENE_221	Protein of unknown function	C	T		8	6			synonymous	strain 12	strain 12
205018	4_86	5wayCG_Leptoll_Contig11277_GENE_222	Putative ABC transporter ATP-binding protein	A	C		7	4			synonymous	strain 12	strain 12
205158	4_86	5wayCG_Leptoll_Contig11277_GENE_222	Putative ABC transporter ATP-binding protein	A	G		9	5			synonymous	strain 12	strain 12
205246-205247	4_86	5wayCG_Leptoll_Contig11277_GENE_222	Putative ABC transporter ATP-binding protein	GG	**		13	1			synonymous	strain 12	strain 12
205365			intergenic	C	A		7	6			intergenic	strain 12	strain 12
205411	4_85	5wayCG_Leptoll_Contig11277_GENE_223	Sulfate-transporting ATPase (EC 3.6.3.25)	T	A		12	3		T/S		strain 12,13	strain 12,13
205838	4_85	5wayCG_Leptoll_Contig11277_GENE_223	Sulfate-transporting ATPase (EC 3.6.3.25)	C	A		3	14			synonymous	strain 12	strain 12

205877	4_85	5wayCG_Leptoll_Contig11277_GENE_223	Sulfate-transporting ATPase (EC 3.6.3.25)	G	A	3	14		synonymous	strain 12	strain 12	
206084	4_85	5wayCG_Leptoll_Contig11277_GENE_223	Sulfate-transporting ATPase (EC 3.6.3.25)	T	C	11	1		synonymous	strain 12	strain 12	
206355	4_84	5wayCG_Leptoll_Contig11277_GENE_224	Putative transmembrane transporter	G	T	3	10		synonymous	strain 12	strain 12	
206385	4_84	5wayCG_Leptoll_Contig11277_GENE_224	Putative transmembrane transporter	T	C	4	10		synonymous	strain 12	strain 12	
206839	4_84	5wayCG_Leptoll_Contig11277_GENE_224	Putative transmembrane transporter	G	C	7	8		synonymous	strain 12	strain 12	
206848	4_84	5wayCG_Leptoll_Contig11277_GENE_224	Putative transmembrane transporter	C	G	7	8		synonymous	strain 12	strain 12	
207009-207011	4_84	5wayCG_Leptoll_Contig11277_GENE_224	Putative transmembrane transporter	GCT	CGG	6	5		synonymous	strain 12	strain 12	
207015	4_84	5wayCG_Leptoll_Contig11277_GENE_224	Putative transmembrane transporter	C	G	6	5		synonymous	strain 12	strain 12	
207026	4_84	5wayCG_Leptoll_Contig11277_GENE_224	Putative transmembrane transporter	T	C	6	7	N/D		strain 12	strain 12	
207244	4_84	5wayCG_Leptoll_Contig11277_GENE_224	Putative transmembrane transporter	T	C	3	4	Q/R				
210026	4_80	5wayCG_Leptoll_Contig11277_GENE_228	Hypothetical	C	T	8	1	V/M				
210609	4_79	5wayCG_Leptoll_Contig11277_GENE_229	Putative A/G-specific DNA glycosylase	G	A	8	5	A/V		strain 13	strain 13	
210767	4_79	5wayCG_Leptoll_Contig11277_GENE_229	Putative A/G-specific DNA glycosylase	G	A	8	5		synonymous	strain 13	strain 13	
211010	4_79	5wayCG_Leptoll_Contig11277_GENE_229	Putative A/G-specific DNA glycosylase	C	T	8	5		synonymous	strain 13	strain 13	
211052	4_79	5wayCG_Leptoll_Contig11277_GENE_229	Putative A/G-specific DNA glycosylase	T	C	7	5		synonymous	strain 13	strain 13	
211587	4_79	5wayCG_Leptoll_Contig11277_GENE_229	Putative A/G-specific DNA glycosylase	T	C	9	14	G/D		strain 13	strain 13	
211796	4_78	5wayCG_Leptoll_Contig11277_GENE_230	Hypothetical	A	G	9	16		synonymous	strain 13	strain 13	
212007	4_78	5wayCG_Leptoll_Contig11277_GENE_230	Hypothetical	A	G	12	7		synonymous	strain 13	strain 13	
212022	4_78	5wayCG_Leptoll_Contig11277_GENE_230	Hypothetical	G	C	13	6		synonymous	strain 13	strain 13	
212403	4_77	5wayCG_Leptoll_Contig11277_GENE_231	Put. 2-amino-4-hydroxy-6-hydroxymethyl-	A	C	8	4	M/R		strain 13	strain 13	
212624	4_77	5wayCG_Leptoll_Contig11277_GENE_231	dihydropteridine diphosphokinase	C	A	9	6		synonymous	strain 13	strain 13	
212786	4_77	5wayCG_Leptoll_Contig11277_GENE_231	Putative 2-amino-4-hydroxy-6-hydroxymethyldihydrop	G	A	10	6		synonymous	strain 13	strain 13	
213271	4_76	5wayCG_Leptoll_Contig11277_GENE_232	Aspartate aminotransferase (EC 2.6.1.1)	G	A	12	7		synonymous	strain 13	strain 13	
213349	4_76	5wayCG_Leptoll_Contig11277_GENE_232	Aspartate aminotransferase (EC 2.6.1.1)	A	C	12	7		synonymous	strain 13	strain 13	
213382	4_76	5wayCG_Leptoll_Contig11277_GENE_232	Aspartate aminotransferase (EC 2.6.1.1)	A	G	13	8		synonymous	strain 13	strain 13	
213534	4_76	5wayCG_Leptoll_Contig11277_GENE_232	Aspartate aminotransferase (EC 2.6.1.1)	C	T	6	10	N/D		strain 13	strain 13	
214730	4_75	5wayCG_Leptoll_Contig11277_GENE_233	Hypothetical	A	G	13	5		synonymous	strain 13	strain 13	
214829	4_75	5wayCG_Leptoll_Contig11277_GENE_233	Hypothetical	G	T	14	5	P/Q		strain 13	strain 13	
214902	4_75	5wayCG_Leptoll_Contig11277_GENE_233	Hypothetical	G	A	15	4	L/F		strain 13	strain 13	
215143	4_74	5wayCG_Leptoll_Contig11277_GENE_234	Hypothetical	T	C	10	5		synonymous	strain 13	strain 13	
215389	4_74	5wayCG_Leptoll_Contig11277_GENE_234	Hypothetical	T	C	8	6		synonymous	strain 13	strain 13	
215596	4_74	5wayCG_Leptoll_Contig11277_GENE_234	Hypothetical	A	G	5	8		synonymous	strain 13	strain 13	
216315	4_73	5wayCG_Leptoll_Contig11277_GENE_235	Hypothetical	A	G	14	1		synonymous	strain 13	strain 13	
217811			intergenic	C	A	9	5		intergenic	strain14	strain14	
217999	4_70	5wayCG_Leptoll_Contig11277_GENE_238	3-oxoacyl-[acyl-carrier-protein] reductase (1.1.1.100)	A	G	10	5	I/V		strain14	strain14	
218010	4_70	5wayCG_Leptoll_Contig11277_GENE_238	3-oxoacyl-[acyl-carrier-protein] reductase (1.1.1.100)	A	T	9	6		synonymous	strain14	strain14	
219619	4_68	5wayCG_Leptoll_Contig11277_GENE_240	Hypothetical	A	G	6	2		synonymous			
220454	4_66	5wayCG_Leptoll_Contig11277_GENE_242	Hypothetical	G	T	6	1		synonymous			
220651		#N/A	intergenic	T	G	8	1		intergenic			
223689	4_62	5wayCG_Leptoll_Contig11277_GENE_246	Put. methyl-accepting chemotaxis sensory transducer	A	G	10	1		synonymous			
223744	4_62	5wayCG_Leptoll_Contig11277_GENE_246	Put. methyl-accepting chemotaxis sensory transducer	C	T	10	1	G/E				
226532	4_60	5wayCG_Leptoll_Contig11277_GENE_248	Putative signal transduction histidine kinase (CheA)	C	*	15	1		split			
227443	4_60	5wayCG_Leptoll_Contig11277_GENE_248	Putative signal transduction histidine kinase (CheA)	A	G	15	1		synonymous			
230033	4_56	5wayCG_Leptoll_Contig11277_GENE_252	Protein of unknown function	G	A	4	3	R/C				
234517	4_53	5wayCG_Leptoll_Contig11277_GENE_255	Flagellar biosynthesis protein	G	A	8	1		synonymous			
234588	4_53	5wayCG_Leptoll_Contig11277_GENE_255	Flagellar biosynthesis protein	A	G	8	1	F/L				
235604	4_52	5wayCG_Leptoll_Contig11277_GENE_256	Flagellar biosynthesis protein	C	*	10	1		split			
236069			intergenic	G	A	13	1		intergenic			
236401	4_50	5wayCG_Leptoll_Contig11277_GENE_258	Flagellar biosynthesis protein FliP	T	C	9	1		synonymous			
238023	4_47	5wayCG_Leptoll_Contig11277_GENE_261	Putative flagellar basal body-associated protein FliL	T	*	15	1		split			
241642	4_44	5wayCG_Leptoll_Contig11277_GENE_264	Conserved protein of unknown function	G	A	6	2		synonymous			
244834	4_40	5wayCG_Leptoll_Contig11277_GENE_268	Protein of unknown function	C	*	10	1		shift change			
246423	4_38	5wayCG_Leptoll_Contig11277_GENE_270	Flagellar M-ring protein FliF	C	*	10	1		split			
248665	4_36	5wayCG_Leptoll_Contig11277_GENE_272	Flagellar basal-body rod protein FlgC	G	A	11	1		synonymous			
250960	4_33	5wayCG_Leptoll_Contig11277_GENE_275	Put. PAS/PAC sensor signal transduction histidine kinas	C	T	15	1	V/I				
259087	4_25	5wayCG_Leptoll_Contig11277_GENE_283	Glycine dehydrogenase (Decarboxylating) subunit 2	G	*	20	1		split			
259186	4_25	5wayCG_Leptoll_Contig11277_GENE_283	Glycine dehydrogenase (Decarboxylating) subunit 2		unit 2				XYG62706.g1			**Recombinant read, but not UBA type**
259385	4_25	5wayCG_Leptoll_Contig11277_GENE_283	Glycine dehydrogenase (Decarboxylating)subunit 2	C	T	14	1	G/R	split			
262017	4_23	5wayCG_Leptoll_Contig11277_GENE_285	Glycine cleavage system H protein	CAGG	**G*	12	1	L/*				
263856			intergenic	C	T	24	2		intergenic			
265110	4_20	5wayCG_Leptoll_Contig11277_GENE_288	Put. methyl-accepting chemotaxis sensory transducer	G	A	10	1	S/L				
266067	4_20	5wayCG_Leptoll_Contig11277_GENE_288	Put. methyl-accepting chemotaxis sensory transducer	G	A	10	3	A/ V				
266298	4_19	5wayCG_Leptoll_Contig11277_GENE_289	Put. methyl-accepting chemotaxis sensory transducer	G	A	19	1		synonymous			
266878	4_18	5wayCG_Leptoll_Contig11277_GENE_290	Protein of unknown function	G	A	12	3	A/ V				
**Contig 11184**												
2985	4_10	5wayCG_Leptoll_Contig11184_GENE_3	Protein of unknown function	G	*	12	1		split			
3725			intergenic	C	A	15	1		intergenic			
6136	4_6	5wayCG_Leptoll_Contig11184_GENE_7	Probable short-chain dehydrogenase/reductase SDR	T	C	10	1	R/G				
12919	59_29	5wayCG_Leptoll_Contig11184_GENE_12	Probable outer membrane efflux protein	C	A	9	1	E/D				
14399	59_28	5wayCG_Leptoll_Contig11184_GENE_13	Protein of unknown function	A	T	14	2		synonymous			
19314-193115	59_21	5wayCG_Leptoll_Contig11184_GENE_20	DNA polymerase III subunit gamma/tau (EC 2.7.7.7)	AA	**	15	1		split			
23171	59_17	5wayCG_Leptoll_Contig11184_GENE_24	Putative mechanosensitive ion channel	C	*	12	1	K/Q	split			
29609	59_11	5wayCG_Leptoll_Contig11184_GENE_30	Probable glycosyl transferase family protein	G	*	16	1		split			
55894	3_11	5wayCG_Leptoll_Contig11184_GENE_54	Dephospho-CoA kinase (EC 2.7.1.24)	T	C	6	7	M/I				
57986	3_14	5wayCG_Leptoll_Contig11184_GENE_57	DNA polymerase I (EC 2.7.7.7)	T	*	16	1		split			
71864	3_27	5wayCG_Leptoll_Contig11184_GENE_69	Glutamate 5-kinase (EC 2.7.2.11)	C	T	9	8		synonymous			
**Contig 11181**												
1177	3_34	5wayCG_Leptoll_Contig11181_GENE_1	Protein of unknown function	G	A	13	2	S/L				

8245	3_41	5wayCG_Leptoll_Contig11181_GENE_7	Ribosomal protein L20	C	A	10	2	A/S				
8744	3_43	5wayCG_Leptoll_Contig11181_GENE_9	Translation initiation factor 3 (IF-3)	G	A	9	2	P/L				
14113	3_45	5wayCG_Leptoll_Contig11181_GENE_11	Putative diguanylate cyclase/phosphodiesterase	A	C	13	3	W/G				
15310	3_45	5wayCG_Leptoll_Contig11181_GENE_11	Putative diguanylate cyclase/phosphodiesterase	G	A	13	4	R/W		Strain 14		Strain 14
15468	3_45	5wayCG_Leptoll_Contig11181_GENE_11	Putative diguanylate cyclase/phosphodiesterase	G	A	13	1	T/M				
15491	3_45	5wayCG_Leptoll_Contig11181_GENE_11	Putative diguanylate cyclase/phosphodiesterase	G	*	12	2		split			
18957	3_49	5wayCG_Leptoll_Contig11181_GENE_15	Thiazole biosynthesis protein (ThiG)	*	C	13	2		split	Strain 14		Strain 14
22115	3_51	5wayCG_Leptoll_Contig11181_GENE_18	Putative glyoxalase (GloA)	C	A	17	1		synonymous			
25763	3_54	5wayCG_Leptoll_Contig11181_GENE_21	Putative metallophosphoesterase	G	A	11	5	V/I				
25777-25778	3_55	5wayCG_Leptoll_Contig11181_GENE_22	Probable major facilitator superfamily transporter	TG	AT	17	1	H/I				
27498	3_56	5wayCG_Leptoll_Contig11181_GENE_23	Carboxymethylenebutenolidase (EC 3.1.1.45)	C	*	11	1		split			
29458	3_57	5wayCG_Leptoll_Contig11181_GENE_24	Putative outer membrane protein	T	G	12	2	Q/P				
29986	3_58	5wayCG_Leptoll_Contig11181_GENE_25	Protein of unknown function	G	A	19	1		synonymous			
31469	3_59	5wayCG_Leptoll_Contig11181_GENE_26	Put. periplasmic signal transduction histidine kinase	G	A	16	1	T/I				
36920	3_65	5wayCG_Leptoll_Contig11181_GENE_32	ATP synthase F1, beta subunit (EC 3.6.3.15)	C	T	13	2	E/K				
38628	3_67	5wayCG_Leptoll_Contig11181_GENE_34	ATP synthase F1, alpha subunit (EC 3.6.3.15)	A	G	20	1	I/T				
43822	3_75	5wayCG_Leptoll_Contig11181_GENE_42	Putative oligopeptide ABC transporter, permease	G	A	14	7		synonymous			
44365	3_75	5wayCG_Leptoll_Contig11181_GENE_42	Putative oligopeptide ABC transporter, permease	T	G	20	1	M/L				
45272	3_76	5wayCG_Leptoll_Contig11181_GENE_43	Putative oligopeptide ABC transporter, permease	C	G	22	1	R/T				
48356	3_79	5wayCG_Leptoll_Contig11181_GENE_46	Triosephosphate isomerase (EC 5.3.1.1)	G	C	20	1		synonymous			
49219	3_80	5wayCG_Leptoll_Contig11181_GENE_47	Phosphoglycerate kinase (EC 2.7.2.3)	A	C	16	1	V/G				
53181-53192	3_82	5wayCG_Leptoll_Contig11181_GENE_49	Peptidoglycan glycosyltransferase (EC 2.4.1.129)	gagaaca aggag	******* *****				split			
60428	3_93	5wayCG_Leptoll_Contig11181_GENE_57	Multi-sensor signal transduction histidine kinase	*	C	10	1		split			
63961	3_95	5wayCG_Leptoll_Contig11181_GENE_59	Protein of unknown function	G	C	11	1	A/G				
68064	3_100	5wayCG_Leptoll_Contig11181_GENE_64	Putative diguanylate phosphodiesterase	G	*	15	1	R/Q	split			
69931	3_103	5wayCG_Leptoll_Contig11181_GENE_67	Putative sulfide-quinone reductase	G	T	16	1	L/I				
75332	147_11	5wayCG_Leptoll_Contig11181_GENE_72	Probable helicase	C	G	15	1		synonymous			
79100-79101	147_9	5wayCG_Leptoll_Contig11181_GENE_74	Putative metal-dependent phosphohydrolase	CC	**	11	1		split			
79922	147_9	5wayCG_Leptoll_Contig11181_GENE_74	Putative metal-dependent phosphohydrolase	C	T	11	1		split			
**Contig 11386**												
31346	26_8	5wayCG_Leptoll_Contig11386_GENE_24	CRISPR-ssociated protein, Cas1	T	A	17	1					
24829	26_13	5wayCG_Leptoll_Contig11386_GENE_20	Hypothetical protein	T	*	4	4		split			
22385-22386	26_15	5wayCG_Leptoll_Contig11386_GENE_18	DNA methyltransferase/helicase	CC	**	5	1		split			
21734	26_15	5wayCG_Leptoll_Contig11386_GENE_18	DNA methyltransferase/helicase	C	A	3	2	R/M				
21116-21224			Helicase						XYG6763.b2		**Recombinant, but not UBA type**	
18541	26_19	5wayCG_Leptoll_Contig11386_GENE_14	Conserved hypothetical protein	A	G	12	2	S/G		Strain 15		Strain 15
18427	26_19	5wayCG_Leptoll_Contig11386_GENE_14	Conserved hypothetical protein	T	C	10	2		synonymous	Strain 15		Strain 15
18412	26-19	5wayCG_Leptoll_Contig11386_GENE_14	Conserved hypothetical protein	A	G	13	2	I/V		Strain 15		Strain 15
18400	26_19	5wayCG_Leptoll_Contig11386_GENE_14	Conserved hypothetical protein	G	A	12	2	V/I		Strain 15		Strain 15
18263	26_19	5wayCG_Leptoll_Contig11386_GENE_14	Conserved hypothetical protein	A	G	12	3	E/G		Strain 15		Strain 15
17671	26_19	5wayCG_Leptoll_Contig11386_GENE_14	Conserved hypothetical protein	A	G	15	1	T/A				
17216-17218	26_19	5wayCG_Leptoll_Contig11386_GENE_14	Conserved hypothetical protein	AGT	TGC	12	1	QF/LL				
16045-16047	26_20	5wayCG_Leptoll_Contig11386_GENE_13	Conserved hypothetical, possibly virulence associate	GGC	***	7	2		split			
15342	26_22	5wayCG_Leptoll_Contig11386_GENE_11	Hypothetical protein	C	T	6	1		synonymous			
13133			intergenic	A	G	6	5		intergenic	Strain 16		Strain 16
13066			intergenic	C	T	6	6		intergenic	Strain 16		Strain 16
13015			intergenic	A	G	4	4		intergenic	Strain 16		Strain 16
13010			intergenic	C	T	4	5		intergenic	Strain 16		Strain 16
12787	26_24	5wayCG_Leptoll_Contig11386_GENE_9	SsrA-binding protein	A	G	5	3		synonymous	Strain 16		Strain 16
12718	26_24	5wayCG_Leptoll_Contig11386_GENE_9	SsrA-binding protein	G	A	4	3		synonymous	Strain 16		Strain 16
12691	26_24	5wayCG_Leptoll_Contig11386_GENE_9	SsrA-binding protein	T	C	4	3		synonymous	Strain 16		Strain 16
12628-12673	26_24	5wayCG_Leptoll_Contig11386_GENE_9	SsrA-binding protein	snips at end of strain (several reads)						Strain 16		Strain 16
11749	183_9	5wayCG_Leptoll_Contig11386_GENE_8	Leucyl aminopeptidase (EC 3.4.11.1)	C	A	8	1		synonymous			
10313	183_9	5wayCG_Leptoll_Contig11386_GENE_8	Leucyl aminopeptidase (EC 3.4.11.1)	C	A	2	2	T/P				
9707	183_7	5wayCG_Leptoll_Contig11386_GENE_6	Putative radical activating enzyme	T	C	6	1		synonymous			
9471	183_7/26_27	5wayCG_Leptoll_Contig11386_GENE_6	Putative radical activating enzyme	T	C	6	1	S/P:E/Q				
8722	26_28/183_6	5wayCG_Leptoll_Contig11386_GENE_5	Protein of unknown function	T	G	7	1		synonymous			
8720	26_28/183_6	5wayCG_Leptoll_Contig11386_GENE_5	Protein of unknown function	C	T	7	1		synonymous			
5506	26_30/183_4	5wayCG_Leptoll_Contig11386_GENE_3	DNA gyrase, B subunit (EC 5.99.1.3)	A	G	7	7	I/V:K/R		Strain 17		Strain 17
4760	26_30/183_4	5wayCG_Leptoll_Contig11386_GENE_3	DNA gyrase, B subunit (EC 5.99.1.3)	T	G	12	1		synonymous			
4757	26_30/!83_4	5wayCG_Leptoll_Contig11386_GENE_3	DNA gyrase, B subunit (EC 5.99.1.3)	A	G	11	1		synonymous			
4568	26_30/!83_4	5wayCG_Leptoll_Contig11386_GENE_3	DNA gyrase, B subunit (EC 5.99.1.3)	C	G	5	5		synonymous	Strain 17		Strain 17
4352	26_30/!83_5	5wayCG_Leptoll_Contig11386_GENE_4	DNA gyrase, A subunit	T	C	4	3		synonymous	Strain 18		Strain 18
4334	26_30/183_6	5wayCG_Leptoll_Contig11386_GENE_5	Protein of unknown function	C	A	5	3		synonymous	Strain 18		Strain 18
3938	26_30/!83_6	5wayCG_Leptoll_Contig11386_GENE_5	Protein of unknown function	G	A	7	3		synonymous	Strain 17		Strain 17
3827	26_30/183_7	5wayCG_Leptoll_Contig11386_GENE_6	Putative radical activating enzyme	G	C	8	4		synonymous	Strain 17		Strain 17
3467	26_30/!83_7	5wayCG_Leptoll_Contig11386_GENE_6	Putative radical activating enzyme	C	G	5	7		synonymous	Strain 17		Strain 17
1779	183_2	5wayCG_Leptoll_Contig11386_GENE_1	Chromosomal replication initiator protein (DnaA) (Ori)	C	T	8	7		synonymous	Strain 17		Strain 17
1718	183_2	5wayCG_Leptoll_Contig11386_GENE_1	Chromosomal replication initiator protein (DnaA)	G	A	7	6		synonymous	Strain 17		Strain 17
1703	183_2	5wayCG_Leptoll_Contig11386_GENE_1	Chromosomal replication initiator protein (DnaA)	A	C	10	3		synonymous	Strain 17		Strain 17
1586	183_2	5wayCG_Leptoll_Contig11386_GENE_1	Chromosomal replication initiator protein (DnaA)	A	G	8	4		synonymous	Strain 17		Strain 17
1553	183_2	5wayCG_Leptoll_Contig11386_GENE_1	Chromosomal replication initiator protein (DnaA)	C	A	6	4		synonymous	Strain 17		Strain 17
1517	183_2	5wayCG_Leptoll_Contig11386_GENE_1	Chromosomal replication initiator protein (DnaA)	T	C	6	4		synonymous	Strain 17		Strain 17
1361	183_2	5wayCG_Leptoll_Contig11386_GENE_1	Chromosomal replication initiator protein (DnaA)	C	A	2	2		synonymous	Strain 17		Strain 17
1178	183_2	5wayCG_Leptoll_Contig11386_GENE_1	Chromosomal replication initiator protein (DnaA)	T	C	4	2		synonymous	Strain 17		Strain 17
1118	183_2	5wayCG_Leptoll_Contig11386_GENE_1	Chromosomal replication initiator protein (DnaA)	C	T	4	2		synonymous	Strain 17		Strain 17
**Contig 11389**												

56017			intergenic	G	A	3	1		intergenic		
38092-38093	26_31-26-33	5wayCG_LeptoII_Contig11389_GENE_40-42	Genes spanning DnaA (origin)						Recomb (Fig) to strain 19		**UBA-5wayCG recombinant**
34887	26_33	5wayCG_LeptoII_Contig11389_GENE_40	Acetolactate synthase, large subunit (EC 2.2.1.6)	T	C	13	5		synonymous	Strain 19	Strain 19
34686	26_33	5wayCG_LeptoII_Contig11389_GENE_40	Acetolactate synthase, large subunit (EC 2.2.1.6)	A	G	11	7		synonymous	Strain 19	Strain 19
34461	26_33	5wayCG_LeptoII_Contig11389_GENE_40	Acetolactate synthase, large subunit (EC 2.2.1.6)	C	T	9	5		synonymous	Strain 19	Strain 19
34437	26_33	5wayCG_LeptoII_Contig11389_GENE_40	Acetolactate synthase, large subunit (EC 2.2.1.6)	G	A	8	7		synonymous	Strain 19	Strain 19
34297	26_33	5wayCG_LeptoII_Contig11389_GENE_40	Acetolactate synthase, large subunit (EC 2.2.1.6)	G	A	10	3		synonymous	Strain 19	Strain 19
33988	26_34	5wayCG_LeptoII_Contig11389_GENE_39	Acetolactate synthase, small subunit	A	G	4	2		synonymous	Strain 19	Strain 19
33769	26_34	5wayCG_LeptoII_Contig11389_GENE_39	Acetolactate synthase, small subunit	C	T	4	4		synonymous	Strain 19	Strain 19
33351	26_35	5wayCG_LeptoII_Contig11389_GENE_38	Acetohydroxy acid isomeroreductase (EC 1.1.1.86)	G	*	13	1		shift/split		
33236	26_35	5wayCG_LeptoII_Contig11389_GENE_38	Acetohydroxy acid isomeroreductase (EC 1.1.1.86)	G	A	12	4		synonymous	Strain 19	Strain 19
33194	26_35	5wayCG_LeptoII_Contig11389_GENE_38	Acetohydroxy acid isomeroreductase (EC 1.1.1.86)	G	A	11	4		synonymous	Strain 19	Strain 19
33093	26_35	5wayCG_LeptoII_Contig11389_GENE_38	Acetohydroxy acid isomeroreductase (EC 1.1.1.86)	C	T	12	5	G/E		Strain 19	Strain 19
32462	26_36	5wayCG_LeptoII_Contig11389_GENE_37	Phosphatidylserine decarboxylase (EC 4.1.1.65)	A	G	12	8		synonymous	Strain 19	Strain 19
32375	26_36	5wayCG_LeptoII_Contig11389_GENE_37	Phosphatidylserine decarboxylase (EC 4.1.1.65)	C	T	18	1		synonymous		
30772	26_38	5wayCG_LeptoII_Contig11389_GENE_35	2-isopropylmalate synthase (EC 2.3.3.13) AA and pyru	A	G	9	13		synonymous	Strain 19	Strain 19
29101	26_39	5wayCG_LeptoII_Contig11389_GENE_34	3-isopropylmalate dehydrogenase (EC 1.1.1.85) AA bi	A	C	16	11		synonymous	Strain 19	Strain 19
28478	26_40	5wayCG_LeptoII_Contig11389_GENE_33	Aspartate-semialdehyde dehydrogenase (EC 1.2.1.11	A	G	15	1		synonymous		
26174			intergenic	C	A	15	1		intergenic		
25924	26_43	5wayCG_LeptoII_Contig11389_GENE_30	Protein of unknown function	G	A	13	1	R/Q			
20508	80_25	5wayCG_LeptoII_Contig11389_GENE_23	Cobalamin B12-binding/Radical SAM family protein	G	T	11	1	V/F			
18176	80_24	5wayCG_LeptoII_Contig11389_GENE_22	Ribulose-bisphosphate carboxylase (EC 4.1.1.39)	G	A	24	1	R/W			
12105	80_17	5wayCG_LeptoII_Contig11389_GENE_15	Putative ribosomal protein L11 methyltransferase	A	G	13	2		synonymous		
7373	80_11	5wayCG_LeptoII_Contig11389_GENE_9	Precorrin-6B methyltransferase	A	*	17	1		split		
5503	80_9	5wayCG_LeptoII_Contig11389_GENE_7	Cobalamin biosynthesis protein (CbiD)	C	*	11	1		split		
6285-6286	214_4	5wayCG_LeptoII_Contig10706_GENE_6	DNA helicase	CA	**	20	1	L/I	split		
**Contig 11172**											
1391			intergenic	T	A	3	9		intergenic		
2727	150_13	5wayCG_LeptoII_Contig11172_GENE_5	Putative inner-membrane protein	T	G	12	4		synonymous		
5885	150_11	5wayCG_LeptoII_Contig11172_GENE_7	Glucose-inhibited division protein A	G	C	12	1	V/L			
8666	150_8	5wayCG_LeptoII_Contig11172_GENE_10	ParB-like partition protein	C	T	6	3		synonymous		
16295	6_88a	5wayCG_LeptoII_Contig11172_GENE_18	Protein of unknown function	T	C	6	1	V/A			
27837-27839	6_77	5wayCG_LeptoII_Contig11172_GENE_31	Pseudouridylate synthases, 23S RNA-specific	TCC	*C*	1	21		split		
29815	6_76	5wayCG_LeptoII_Contig11172_GENE_32	Putative TPR-domain containing protein	C	A	18	1	R/L			
31290	6_74	5wayCG_LeptoII_Contig11172_GENE_34	Isoleucyl-tRNA synthetase (EC 6.1.1.5)	T	C	16	1	I/V			
40618	48_36	5wayCG_LeptoII_Contig11172_GENE_42	Putative aminoglycoside phosphotransferase	G	A	18	1	E/K			
40661	48_36	5wayCG_LeptoII_Contig11172_GENE_42	Putative aminoglycoside phosphotransferase	C	T	16	2	T/I			
42451	48_34	5wayCG_LeptoII_Contig11172_GENE_44	Protein of unknown function	T	*	20	4		split		
43685	48_32	5wayCG_LeptoII_Contig11172_GENE_46	Putative peptidyl-prolyl cis-trans isomerase	C	T	18	1	D/N			
47690			intergenic	A	C	12	1		intergenic		
48901	48_29	5wayCG_LeptoII_Contig11172_GENE_49	Transcription-repair coupling factor	C	T	20	2	E/K			
53009			intergenic	G	T	10	7		intergenic		
53894			intergenic	G	*	9	1		intergenic		
58537	48_16	5wayCG_LeptoII_Contig11172_GENE_61	Pyruvate synthase alpha chain (EC 1.2.7.1)	T	C	16	1		synonymous		
58922	48_16	5wayCG_LeptoII_Contig11172_GENE_61	Pyruvate synthase alpha chain (EC 1.2.7.1)	C	*	14	1		split		
65013			intergenic	G	A	6	1		intergenic		
67338	48_5	5wayCG_LeptoII_Contig11172_GENE_71	Putative heat shock protein DnaJ, N-terminal	G	A	4	2		synonymous	Strain 20	Strain 20
69832	48_3	5wayCG_LeptoII_Contig11172_GENE_73	Chaperone clpB	G	C	8	3		synonymous	Strain 20	Strain 20
70180	48_3	5wayCG_LeptoII_Contig11172_GENE_73	Chaperone clpB	T	C	7	4		synonymous	Strain 20	Strain 20
71044	48_2	5wayCG_LeptoII_Contig11172_GENE_74	Probable bacterial regulatory protein, Fis family	T	G	2	1	S/A			
71081	48_2	5wayCG_LeptoII_Contig11172_GENE_74	Probable bacterial regulatory protein, Fis family	G	A	2	1	R/H			
**Contig 11111**											
117007	43_1	5wayCG_LeptoII_Contig11111_GENE_114	Putative heavy metal efflux pump, CzcA family	A	g	10	5		synonymous		
115373	43_3	5wayCG_LeptoII_Contig11111_GENE_112	Protein of unknown function	T	A	8	6	D/E		Strain 21	Strain 21
115282	43_3	5wayCG_LeptoII_Contig11111_GENE_112	Protein of unknown function	C	T	8	7	A/ V		Strain 21	Strain 21
114006	43_4	5wayCG_LeptoII_Contig11111_GENE_111	Putative Xaa-Pro aminopeptidase	T	C	13	1	F/L			
113650	43_5	5wayCG_LeptoII_Contig11111_GENE_110	Probable O-methyltransferase family protein	T	C	11	6		synonymous	strain 22	strain 22
112841			intergenic	G	A	13	1		synonymous		
111117	43_8	5wayCG_LeptoII_Contig11111_GENE_107	Glucose-1-phosphate adenylyltransferase (EC 2.7.7.27	A	G	18	3		synonymous	strain 22	strain 22
109424	43_9	5wayCG_LeptoII_Contig11111_GENE_106	Putative glycoside hydrolase, family 57	A	G	18	5	V/A		strain 22	strain 22
109023	43_9	5wayCG_LeptoII_Contig11111_GENE_106	Putative glycoside hydrolase, family 57	G	*	17	1		split		
108814	43_9	5wayCG_LeptoII_Contig11111_GENE_106	Putative glycoside hydrolase, family 57	C	G	17	8		synonymous	strain 22	strain 22
107510	43_10	5wayCG_LeptoII_Contig11111_GENE_105	4-alpha-glucanotransferase (EC 2.4.1.25)	C	A	17	5	A/S		strain 22	strain 22
104687	43_12	5wayCG_LeptoII_Contig11111_GENE_103	Probable aldolase	C	T	14	7		synonymous	strain 22	strain 22
104685	43_12	5wayCG_LeptoII_Contig11111_GENE_103	Probable aldolase	T	G	12	6	D/A		strain 22	strain 22
103629	43_13	5wayCG_LeptoII_Contig11111_GENE_102	Putative glycoside hydrolase, family 57	C	T	4	14		synonymous	strain 22	strain 22
103359	43_13	5wayCG_LeptoII_Contig11111_GENE_102	Putative glycoside hydrolase, family 57	G	A	5	19		synonymous	strain 22	strain 22
103326	43_13	5wayCG_LeptoII_Contig11111_GENE_102	Putative glycoside hydrolase, family 57	C	T	5	20		synonymous	strain 22	strain 22
103317	43_13	5wayCG_LeptoII_Contig11111_GENE_102	Putative glycoside hydrolase, family 57	G	T	5	20		synonymous	strain 22	strain 22
103287	43_13	5wayCG_LeptoII_Contig11111_GENE_102	Putative glycoside hydrolase, family 57	C	T	5	20		synonymous	strain 22	strain 22
102717	43_13	5wayCG_LeptoII_Contig11111_GENE_102	Putative glycoside hydrolase, family 57	C	A	3	15		synonymous	strain 22	strain 22
92995	43_19	5wayCG_LeptoII_Contig11111_GENE_96	Putative outer membrane efflux protein	C	T	11	1		synonymous		
91118	43_20	5wayCG_LeptoII_Contig11111_GENE_95	Thioredoxin reductase (EC 1.8.1.9)	A	G	3	12		synonymous		
90073			intergenic	A	C	11	4		intergenic		
89140	43_22	5wayCG_LeptoII_Contig11111_GENE_93	Protein of unknown function	C	T	12	1		synonymous		
88574	43_22	5wayCG_LeptoII_Contig11111_GENE_93	Protein of unknown function	C	*	15	1		split		
87086	43_23	5wayCG_LeptoII_Contig11111_GENE_92	Protein of unknown function	G	T	26	2	P/T			
83658	43_28	5wayCG_LeptoII_Contig11111_GENE_87	Putative peptidase, M48 family	C	T	17	1	M/I			

82929	43_29	5wayCG_LeptoII_Contig11111_GENE_86	Ribulose-phosphate 3-epimerase (EC 5.1.3.1)	T	*	13	1		split		
81426	277_2	5wayCG_LeptoII_Contig11111_GENE_84	Putative cell shape determining protein (MreB/Mrl)	*	C	16	1		split		
80905-80906	277_2	5wayCG_LeptoII_Contig11111_GENE_84	Putative cell shape determining protein (MreB/Mrl)	GG	**	15	1	SI/YS			
78085	277_5	5wayCG_LeptoII_Contig11111_GENE_81	Peptidoglycan glycosyltransferase (EC 2.4.1.129)	G	A	20	1	P/L			
77739	277_5	5wayCG_LeptoII_Contig11111_GENE_81	Peptidoglycan glycosyltransferase (EC 2.4.1.129)	C	T	20	1		synonymous		
74950	277_7	5wayCG_LeptoII_Contig11111_GENE_79	Ribonucleases G and E	T	G	3	6		synonymous		
73603	277_9	5wayCG_LeptoII_Contig11111_GENE_77	Putative dihydroneopterin aldolase	T	C	9	2		synonymous		
73169			intergenic	T	C	4	2		intergenic		
70336	426_3	5wayCG_LeptoII_Contig11111_GENE_72	Ribosomal protein L13	C	T	2	5		synonymous		
69479	426_5	5wayCG_LeptoII_Contig11111_GENE_70	N-acetyl-gamma-glutamyl-phosphate reductase	A	G	19	11		synonymous	strain 23	strain 23
69020	426_5	5wayCG_LeptoII_Contig11111_GENE_70	N-acetyl-gamma-glutamyl-phosphate reductase	G	T	18	1		synonymous		
68147	426_6	5wayCG_LeptoII_Contig11111_GENE_69	Arginine biosynthesis protein (ArgJ) (EC 2.3.1.35)	C	*	4	1		split		
67993	426_6	5wayCG_LeptoII_Contig11111_GENE_69	Arginine biosynthesis protein (ArgJ) (EC 2.3.1.35)	C	T	6	2	S/N		strain 23	strain 23
67902	426_6	5wayCG_LeptoII_Contig11111_GENE_69	Arginine biosynthesis protein (ArgJ) (EC 2.3.1.35)	A	G	9	3		synonymous	strain 23	strain 23
66507	13_68	5wayCG_LeptoII_Contig11111_GENE_68	Ribosomal protein S2	C	*	7	1		split/shift		
65702	13_67	5wayCG_LeptoII_Contig11111_GENE_67	Elongation factor Ts (EF-Ts)	A	T	6	5	F/L		strain 24	strain 24
65645	13_67	5wayCG_LeptoII_Contig11111_GENE_67	Elongation factor Ts (EF-Ts)	G	A	6	4		synonymous	strain 24	strain 24
63443	13_64	5wayCG_LeptoII_Contig11111_GENE_64	Alanine racemase (EC 5.1.1.1)	G	T	4	13	K/L		strain 24	strain 24
63350	13_64	5wayCG_LeptoII_Contig11111_GENE_64	Alanine racemase (EC 5.1.1.1)	T	C	6	13		synonymous	strain 24	strain 24
63144	13_64	5wayCG_LeptoII_Contig11111_GENE_64	Alanine racemase (EC 5.1.1.1)	G	A	5	14	L/S		strain 24	strain 24
62979			intergenic	C	T	5	12		intergenic	strain 24	strain 24
62661	13_63	5wayCG_LeptoII_Contig11111_GENE_63	Putative integral membrane protein	C	T	17	2		synonymous		
62448	13_63	5wayCG_LeptoII_Contig11111_GENE_63	Putative integral membrane protein	A	G	6	11		synonymous	strain 24	strain 24
61442	13_61	5wayCG_LeptoII_Contig11111_GENE_61	Putative ABC transporter	G	A	10	4		synonymous	strain 24	strain 24
61271	13_61	5wayCG_LeptoII_Contig11111_GENE_61	Putative ABC transporter	A	G	11	6		synonymous	strain 24	strain 24
60536	13_61	5wayCG_LeptoII_Contig11111_GENE_61	Putative ABC transporter	A	G	15	2		synonymous	strain 24	strain 24
60185	13_61	5wayCG_LeptoII_Contig11111_GENE_61	Putative ABC transporter	T	C	13	2		synonymous	strain 24	strain 24
60110	13_61	5wayCG_LeptoII_Contig11111_GENE_61	Putative ABC transporter	A	G	12	1		synonymous	strain 24	strain 24
59471	13_60	5wayCG_LeptoII_Contig11111_GENE_60	ResB-like family protein (poss cyt biosyn)	G	A	3	3		synonymous	strain 24	strain 24
59150	13_60	5wayCG_LeptoII_Contig11111_GENE_60	ResB-like family protein (poss cyt biosyn)	A	G	11	4		synonymous	strain 24	strain 24
58089			intergenic	C	A	12	3		intergenic	strain 24	strain 24
57393	13_59	5wayCG_LeptoII_Contig11111_GENE_59	Putative cytochrome c biogenesis protein	G	A	3	14		split	strain 24	strain 24
52335	13_53	5wayCG_LeptoII_Contig11111_GENE_53	Primosomal protein N' (PriA)	G	*	23	1		split/shift		
50948			intergenic	C	*	15	1		intergenic		
49044			intergenic	C	T	19	1		intergenic		
45370	13_46	5wayCG_LeptoII_Contig11111_GENE_46	Putative Ppx/GppA phosphatase family protein	T	C	4	12		synonymous	strain 25	strain 25
44819			intergenic	T	G	23	2		intergenic		
44748	13_45	5wayCG_LeptoII_Contig11111_GENE_45	Protein of unknown function	C	A	19	7		synonymous	strain 25	strain 25
44664	13_45	5wayCG_LeptoII_Contig11111_GENE_45	Protein of unknown function	G	A	17	7		synonymous	strain 25	strain 25
44486	13_45	5wayCG_LeptoII_Contig11111_GENE_45	Protein of unknown function	T	*	23	2		split/shift		
44298	13_45	5wayCG_LeptoII_Contig11111_GENE_45	Protein of unknown function	T	C	6	20		synonymous	strain 25	strain 25
42974-42975	13_44	5wayCG_LeptoII_Contig11111_GENE_44	Ubiquinone/menaquinone biosyn. methyltransferase	AT	**	19	1				
41908	13_43	5wayCG_LeptoII_Contig11111_GENE_43	Putative radical SAM family protein	A	G	19	2		synonymous	strain 25	strain 25
40971	13_42	5wayCG_LeptoII_Contig11111_GENE_42	Protein of unknown function	C	T	7	9	V/I		strain 25	strain 25
40384	13_41	5wayCG_LeptoII_Contig11111_GENE_41	Putative 4-hydroxybenzoate polyprenyl transferase	C	G	15	6	V/L		strain 25	strain 25
40220	13_35	5wayCG_LeptoII_Contig11111_GENE_36	Acetylornithine aminotransferase (EC 2.6.1.11)	T	*	15	1		shift		
36264	13_35	5wayCG_LeptoII_Contig11111_GENE_36	Acetylornithine aminotransferase (EC 2.6.1.11)	A	G	19	1		synonymous		
36225-36226	13_35	5wayCG_LeptoII_Contig11111_GENE_36	Acetylornithine aminotransferase (EC 2.6.1.11)						XYD6958.b1		**UBA-5wayCG recombinant**
35946	13_35	5wayCG_LeptoII_Contig11111_GENE_36	Acetylornithine aminotransferase (EC 2.6.1.11)	C	T	15	1		synonymous		
35128	13_34	5wayCG_LeptoII_Contig11111_GENE_35	Ornithine carbamoyltransferase (EC 2.1.3.3)	G	A	12	9		synonymous	Strain 26	Strain 26
34948	13_34	5wayCG_LeptoII_Contig11111_GENE_35	Ornithine carbamoyltransferase (EC 2.1.3.3)	C	A	9	8		synonymous	Strain 26	Strain 26
34765	13_34	5wayCG_LeptoII_Contig11111_GENE_35	Ornithine carbamoyltransferase (EC 2.1.3.3)	T	A	11	11		synonymous	Strain 26	Strain 26
34201	13_33	5wayCG_LeptoII_Contig11111_GENE_34	Argininosuccinate synthase (EC 6.3.4.5)	G	A	12	12		synonymous	Strain 26	Strain 26
33722	13_33	5wayCG_LeptoII_Contig11111_GENE_34	Argininosuccinate synthase (EC 6.3.4.5)	G	T	19	2	S/Y		Strain 26	Strain 26
33670-33275	13_33	5wayCG_LeptoII_Contig11111_GENE_34	Argininosuccinate synthase (EC 6.3.4.5)						XYD24967.x1		**UBA-5wayCG recombinant**
33043	13_32	5wayCG_LeptoII_Contig11111_GENE_33	Argininosuccinate lyase (EC 4.3.2.1)	G	A	26	1	R/W			
32986	13_32	5wayCG_LeptoII_Contig11111_GENE_33	Argininosuccinate lyase (EC 4.3.2.1)	T	*	25	1		split/shift		
32477	13_32	5wayCG_LeptoII_Contig11111_GENE_33	Argininosuccinate lyase (EC 4.3.2.1)	T	A	11	16		synonymous	Strain 27	Strain 27
32399	13_32	5wayCG_LeptoII_Contig11111_GENE_33	Argininosuccinate lyase (EC 4.3.2.1)	G	A	10	17		synonymous	Strain 27	Strain 27
31074	13_31	5wayCG_LeptoII_Contig11111_GENE_32	Diaminopimelate decarboxylase (EC 4.1.1.20)	C	T	13	1		synonymous	Strain 27	Strain 27
29972	13_30	5wayCG_LeptoII_Contig11111_GENE_31	Protein of unknown function	T	G	8	3		synonymous	Strain 27	Strain 27
29054	13_29	5wayCG_LeptoII_Contig11111_GENE_30	Dihydrodipicolinate synthase (EC 4.2.1.52)	G	A	17	1	A/ V		Strain 28	Strain 28
28088	13_27	5wayCG_LeptoII_Contig11111_GENE_28	5-carboxymethyl-2-hydroxymuconate delta-isomerase	T	*	10	2		synonymous	Strain 28	Strain 28
26646	13_26	5wayCG_LeptoII_Contig11111_GENE_27	Putative flagellar basal body rod protein	G	C	10	3		synonymous	Strain 28	Strain 28
23985	13_22	5wayCG_LeptoII_Contig11111_GENE_23	Putative flagellar P-ring protein	A	C	5	10		synonymous	Strain 28	Strain 28
23973	13_22	5wayCG_LeptoII_Contig11111_GENE_23	Putative flagellar P-ring protein	C	T	5	10		synonymous	Strain 28	Strain 28
23086	13_22	5wayCG_LeptoII_Contig11111_GENE_23	Putative flagellar P-ring protein	T	C	8	13	S/N		Strain 28	Strain 28
23063	13_21	5wayCG_LeptoII_Contig11111_GENE_22	Conserved hypothetical protein	A	G	6	5	F/S		Strain 28	Strain 28
22801	13_21	5wayCG_LeptoII_Contig11111_GENE_22	Conserved hypothetical protein	G	T	4	4	N/K			
22115	13_20	5wayCG_LeptoII_Contig11111_GENE_21	Putative flagellin	G	C	4	10		synonymous		
22004	13_20	5wayCG_LeptoII_Contig11111_GENE_21	Putative flagellin	G	A	13	1		synonymous		
20265	13_17	5wayCG_LeptoII_Contig11111_GENE_18	Flagellar hook-associated protein (FlgK)	C	T	5	2		synonymous		
19708	13_17	5wayCG_LeptoII_Contig11111_GENE_18	Flagellar hook-associated protein (FlgK)	A	G	6	8	A/ V			
19699	13_17	5wayCG_LeptoII_Contig11111_GENE_18	Flagellar hook-associated protein (FlgK)	C	T	5	8	N/S			
19673	13_17	5wayCG_LeptoII_Contig11111_GENE_18	Flagellar hook-associated protein (FlgK)	*	C	11	1		split		
16960	13_13	5wayCG_LeptoII_Contig11111_GENE_14	Putative flagellin	G	A	13	9		synonymous	Strain 28	Strain 28
16765	13_13	5wayCG_LeptoII_Contig11111_GENE_14	Putative flagellin	A	C	12	4	S/A		Strain 28	Strain 28
16412			intergenic	A	C	12	2		intergenic	Strain 28	Strain 28

15960	13_12	5wayCG_LeptoII_Contig11111_GENE_13	Putative flagellar hook-associated protein (FliD)	C	*	13	1		split		
15556	13_12	5wayCG_LeptoII_Contig11111_GENE_13	Putative flagellar hook-associated protein (FliD)	A	G	14	6	F/S		Strain 28	Strain 28
14321	13_10	5wayCG_LeptoII_Contig11111_GENE_11	Hypothetical	G	A	5	15		synonymous	Strain 28	Strain 28
14318	13_10	5wayCG_LeptoII_Contig11111_GENE_11	Hypothetical	C	T	5	15		synonymous	Strain 28	Strain 28
13951			intergenic	A	C	4	17		intergenic	Strain 28	Strain 28
13610	13_9	5wayCG_LeptoII_Contig11111_GENE_10	Conserved protein of unknown function	T	A	5	12	F/Y		Strain 28	Strain 28
11967	13_8	5wayCG_LeptoII_Contig11111_GENE_9	Putative SAM-dependent methyltransferases	G	A	13	7		synonymous	Strain 28	Strain 28
11675	13_8	5wayCG_LeptoII_Contig11111_GENE_9	Putative SAM-dependent methyltransferases	C	T	13	7	I/ V		Strain 28	Strain 28
11546	13_8	5wayCG_LeptoII_Contig11111_GENE_9	Putative SAM-dependent methyltransferases	C	*	18	1		split		
11235	13_8	5wayCG_LeptoII_Contig11111_GENE_9	Putative SAM-dependent methyltransferases	A	G	5	4		synonymous	Strain 28	Strain 28
11166	13_8	5wayCG_LeptoII_Contig11111_GENE_9	Putative SAM-dependent methyltransferases	C	A	5	10		synonymous	Strain 28	Strain 28
11004	13_8	5wayCG_LeptoII_Contig11111_GENE_9	Putative SAM-dependent methyltransferases	A	C	5	10		synonymous	Strain 28	Strain 28
10141	13_7	5wayCG_LeptoII_Contig11111_GENE_8	Putative heptosyltransferase family protein	C	T	9	5		synonymous	Strain 28	Strain 28
10064	13_7	5wayCG_LeptoII_Contig11111_GENE_8	Putative heptosyltransferase family protein	T	G	7	4	E/A		Strain 28	Strain 28
9906	13_7	5wayCG_LeptoII_Contig11111_GENE_8	Putative heptosyltransferase family protein	A	G	5	3		synonymous	Strain 28	Strain 28
9832	13_7	5wayCG_LeptoII_Contig11111_GENE_8	Putative heptosyltransferase family protein	*	C	8	1		split		
8910	13_6	5wayCG_LeptoII_Contig11111_GENE_6	Conserved hypothetical protein	C	G	12	6		synonymous	Strain 28	Strain 28
8804-8815	13_6	5wayCG_LeptoII_Contig11111_GENE_6	Conserved hypothetical protein	ctatcctatcct	ctatcct***** **a***tatcct	1	9	5 S/I	shift	Strain 28	Strain 28
8787	13_6	5wayCG_LeptoII_Contig11111_GENE_6	Conserved hypothetical protein	C	G	10	5	A/P		Strain 28	Strain 28
8499	13_6	5wayCG_LeptoII_Contig11111_GENE_6	Conserved hypothetical protein	A	T	8	3	W/R		Strain 28	Strain 28
8196	13_6	5wayCG_LeptoII_Contig11111_GENE_6	Conserved hypothetical protein	C	A	10	5	A/S		Strain 28	Strain 28
8026	13_6	5wayCG_LeptoII_Contig11111_GENE_6	Conserved hypothetical protein	C	A	9	3		synonymous	Strain 28	Strain 28
6936	13_5	5wayCG_LeptoII_Contig11111_GENE_5	Probable heptosyltransferase family protein	C	A	8	1	R/L			
6630	13_5	5wayCG_LeptoII_Contig11111_GENE_5	Probable heptosyltransferase family protein	T	*	13	1		split		
4032	13_3	5wayCG_LeptoII_Contig11111_GENE_2	Hypothetical	T	C	13	3		synonymous	Strain 29	Strain 29
3125	13_3	5wayCG_LeptoII_Contig11111_GENE_2	Hypothetical	C	T	11	5	R/H		Strain 29	Strain 29
