# Supplementary material for: Population Genomic Analysis of Strain Variation in Leptospirillum Group II Bacteria Involved in Acid Mine Drainage Formation
Source: PLoS Biol. 2008 Jul 22;6(7):e177. doi: 10.1371/journal.pbio.0060177 (PMC2475542; doi:10.1371/journal.pbio.0060177)
Supplement: Supplementary file 7 [file pbio.0060177.st004.pdf]

Quality cutoff	P(error)	Segrep/ Segsil	Fixrep/ Fixsil	Segrep	Segsil	Total sites	Exp Segrep/Segsil*	pN/pS	dN/dS
0	1	3.14	0.59	1950	622	2572		1.09	0.21
10	1.0E-01	2.43	0.56	630	259	889	13.6	0.85	0.20
20	1.0E-02	1.12	0.53	130	116	246	4.51	0.39	0.18
25	3.2E-03	1.12	0.52	118	105	223	1.06	0.39	0.18
30	1.0E-03	1.03	0.52	104	101	205	1.07	0.36	0.18
40	1.0E-04	1.03	0.53	96	93	189	0.98	0.36	0.18
50	1.0E-05	1.16	0.52	78	67	145	0.85	0.41	0.18
25, replicated only, all depths	3.2E-03	1.54	0.53	40	26	66		0.54	0.18
25, depth > 3	3.2E-03	1.31	0.55	106	81	187		0.46	0.19
25, depth > 4	3.2E-03	1.28	0.49	95	74	169		0.45	0.19

Table S4: The results of MK tests for Phred quality score cutoffs of 20 and higher are nearly identical, as indicated here by the similarity in ratios of segregating replacement to silent sites and fixed replacement to fixed silent sites for cutoff scores higher than 20. The use of replicated SNPs only or sites at higher coverage levels only also does not affect the outcome. *, Expected ratio of segregating replacement to segregating sites if 2/3 of masked sites are replacement and 1/3 are silent. The effect of increasing the minimum required coverage depth for a quality score cutoff of 25 is shown in the bottom three rows.
